# Treatment with L-type amino acid transporter 1 inhibitor JPH203 enhances protein synthesis in C2C12 myotubes

**DOI:** 10.1038/s41598-025-24534-2

**Published:** 2025-11-19

**Authors:** Junya Takegaki, Takaoki Saneyasu, Kazuhisa Honda

**Affiliations:** Graduate School of Agricultural Science, Kobe University, 1-1 Rokkodai-cho, Nada-ku, Kobe, Hyogo 657-8501 Japan

**Keywords:** L-type amino acid transporter 1, Leucine, Mechanistic target of rapamycin, Muscle protein synthesis, Myotubes, Biochemistry, Cell biology, Physiology

## Abstract

Excessive muscle protein synthesis causes skeletal muscle hypertrophy. Essential amino acids are substrates for muscle proteins and stimulate muscle protein synthesis. Several essential amino acids are taken up into muscle cells through L-type amino acid transporter 1 (LAT1). However, LAT1 may influence protein synthesis in an amino acid uptake-independent manner. Here, we investigated the effects of LAT1 inhibition on protein synthesis in C2C12 myotubes and the associated mechanisms. JPH203 (50 μM), a selective inhibitor of LAT1, stimulated protein synthesis without changing expression of phosphorylated p70S6K (T389) and 4EBP1 (T37/46), an indicator of mTORC1 activity. Culturing in amino acid-free media did not suppress JPH203-induced protein synthesis. The mTORC1 inhibitor rapamycin (100 nM) did not suppress JPH203-induced protein synthesis. ATP-competitive mTOR inhibitor AZD8055 (1 μM) suppressed JPH203-induced protein synthesis. JPH203 treatment increased intracellular glutamine concentration. These results suggest that inhibition of LAT1 function augments muscle protein synthesis, possibly through the activation of rapamycin-insensitive mTOR signaling; elevated intracellular glutamine levels may contribute to the enhancement of muscle protein synthesis induced by LAT1 inhibition.

**Supplementary Information:**

The online version contains supplementary material available at 10.1038/s41598-025-24534-2.

## Introduction

Skeletal muscle plays a crucial role not only in body movement but also in whole-body metabolism, and maintaining its mass is essential for improving performance and promoting health^1,2^. The mass of skeletal muscle is increased through the accumulation of muscle protein, and numerous nutritional interventions have been developed to activate muscle protein synthesis and accelerate muscle hypertrophy^3,4^. Essential amino acids (EAAs) are a typical example of such interventions and are known to exert a strong effect in small quantities^5,6^.

EAAs have the potential to activate muscle protein synthesis, and the potency of leucine, one of the branched-chain amino acids (BCAAs), is known to be extremely high^5,6^. This high potency is attributed to the capacity of leucine to activate mTORC1, which plays a role in muscle protein synthesis in response to resistance exercise and EAAs intake^7–10^. EAAs and BCAAs, especially those enriched in leucine, can stimulate the anabolic response of skeletal muscle with a smaller amount than other nutrients (e.g., whey protein, etc.), and are widely used for applications such as accelerating the response associated with resistance exercise^11–13^.

Leucine, along with neutral amino acids such as other BCAAs, is transported into skeletal muscle via the L-type amino acid transporter 1 (solute carrier family 7 member 5, LAT1)^14^. Although other LAT family members (LAT2-4) can also transport leucine, LAT1 plays a primary role in the acute uptake of leucine and has attracted attention as a key transporter involved in amino acid transport and muscle protein synthesis in skeletal muscle^15–20^. Several studies have reported that LAT1 mRNA levels increase transiently following the intake of EAAs or resistance exercise, typically peaking at 3–6 h^21–23^. In these reports, increase in LAT1 protein expression up to 3 h or 24 h after EAAs intake or single bout of resistance exercise were observed primarily in young individuals^21,22^, although the data beyond these time points lacked and some previous studies reported unchanged^20,23–25^. Prolonged resistance training has been reported to increase the expression of LAT1 in skeletal muscle^26^. While LAT1 expression can change in response to exercise and nutrition, the role of LAT1 in regulating muscle protein synthesis has not been fully elucidated.

A previous study reported that muscle-specific LAT1 knockout reduced p70S6K phosphorylation (a marker of mTORC1 activity) in response to leucine injection and a high-protein diet, but did not change muscle mass in mice. This finding was suggested to be due to compensatory expression of LAT2^15^. There are also some in vitro studies on the contribution of LAT1 to the regulation of skeletal muscle mass. LAT1 inhibition by 2-aminobicyclo-(2,2,1)-heptane-2-carboxylic acid and knockdown interfered with myotube differentiation in C2C12 myoblasts^27^. Overexpression of LAT1 decreases protein synthesis in C2C12 myoblasts^26^. Knockdown of neuronal precursor cell-expressed developmentally downregulated gene 4, a regulator of ubiquitin-dependent breakdown of LAT1, attenuated the decrease of LAT1 expression and protein synthesis caused by chemotherapy drug cocktail administration in L6 myotubes^28^. Despite this accumulating evidence, the mechanism by which LAT1 directly regulates muscle protein synthesis under physiological conditions remains unclear.

The use of a pharmacological inhibitor and differentiated myotubes enables the evaluation of effects without compensatory changes in transporter expression, allowing for a more physiological assessment. Therefore, in the present study, we investigated the effect of administering the LAT1-selective inhibitor JPH203 on protein synthesis and the possible mechanisms underlying the changes in C2C12 myotubes. We hypothesized that JPH203 treatment would reduce intracellular leucine concentration and attenuate protein synthesis, but the results were somewhat unpredictable.

## Materials and methods

### Cell culture

The C2C12 murine myoblast cell line was purchased from DS Pharma Biomedical Co. Ltd. (Osaka, Japan) and cultured in Dulbecco’s Modified Eagle Medium (DMEM, pH = 7.0–7.5, 08458-45, Nacalai Tesque, Kyoto, Japan) supplemented with 10% Fetal Clone III (Cytiva, Tokyo, Japan), penicillin (100 U/mL) and streptomycin (100 μg/mL) at 37 °C in 5% CO_2_/95% air. When myoblasts reached 90% confluency, the medium was changed to DMEM supplemented with 2% horse serum, and the cells were cultured for 4–6 days to induce differentiation into myotubes.

**Fig. 1:**
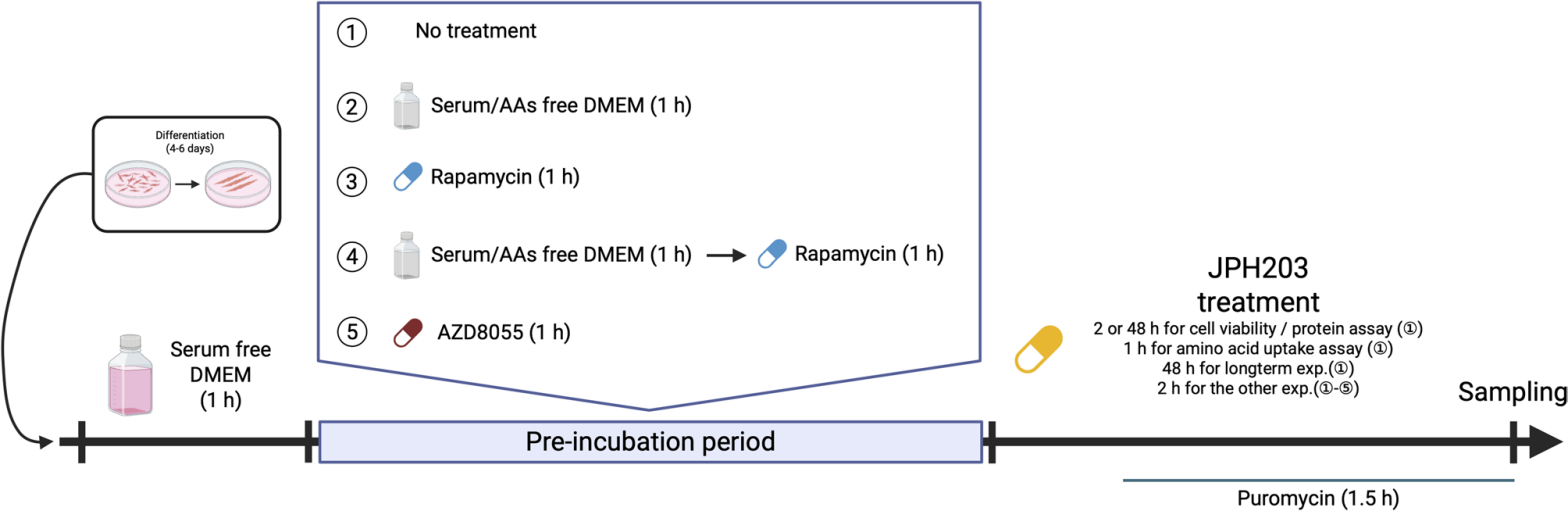
A schematic overview of the experimental time course for each intervention.

For experimental treatments, myotubes were first cultured in serum-free DMEM for 1 h. In some experiments, cells were subsequently incubated for an additional hour in DMEM lacking both serum and amino acids for 1 h, as deprivation of serum and amino acids is commonly performed for 1–4 h^29,30^. For amino acid uptake assay, myotubes were treated with or without JPH203 (dihydrochloride, 36053, Cayman Chemical, MI, USA) alone for 1 h. In other short-term assays, treatment lasted 2 h, since the inhibitory effect on LAT1 has been reported to persist at least 2 h^31^. Long-term experiments were performed with 48-h treatment. Concentration-dependent assays were performed using 1–100 μM JPH203, a range widely adopted in previous studies on other cell types^32,33^. In some experiments, JPH203 treatment was combined with either rapamycin (R124000, Toronto Research Chemicals, Toronto, Canada) or AZD8055 (A10114, AdooQ Bioscience, CA, USA) with a 1-h preincubation at concentrations of 100 nM and 1 μM, respectively, as used in previous reports^34,35^. The time course of each intervention is summarized in Fig. 1. All compounds were dissolved in dimethyl sulfoxide (DMSO), and the final concentration of DMSO in the culture media was ≤ 0.1%. For the measurement of muscle protein synthesis, puromycin was administered in the last 90 min following previous research^36^.

### Cell viability assay

Cell viability after 2 or 48 h of JPH203 treatment, a cell viability assay was performed using a Cell Counting Kit-8 (Dojindo Laboratories, Kumamoto, Japan) according to the manufacturer’s protocol with slight modifications. Briefly, C2C12 myoblasts were seeded in 96-well culture plates and differentiated into myotubes as described above. The myotubes were then pre-incubated with serum-free DMEM for 1 h, and treated with JPH203 for 2 h or 48 h. Finally, CCK-8 solution was added to each well and 3-h color reaction was performed.

### Amino acid uptake assay

The amino acid uptake capacity of C2C12 myotubes was assessed using the amino acid uptake assay kit (342-09893, Dojindo, Kumamoto, Japan) following the manufacturer’s protocol. Briefly, the myotubes were cultured in 12-well plates, and JPH203 was added at a final concentration of 50 μM after culturing in serum-free DMEM for 1 h. The medium was then removed and the myotubes were washed with Hank’s Balanced Salt Solution (HBSS pH = 7.0–7.6, 37 °C, 17459-55, Nacalai Tesque) for 3 times, and the myotubes were incubated with HBSS which does not contain JPH203 or vehicle for 5 min at 37 °C. Then, the medium was replaced with a boronophenylalanine uptake solution, which was prepared using HBSS as the base buffer, and incubated for 5 min. It was then changed to a working solution which contains fluorescent probe and prepared using HBSS as the base buffer after 3 times of washing with HBSS, and incubated for an additional 5 min at 37 °C. Finally, observations were made using a fluorescence microscope (BZ-9000 microscope, Keyence Co., Osaka, Japan), and the fluorescence intensity was measured using ImageJ.

### Amino acid analysis

For the analysis of amino acids in the culture medium, 40 μL of the media was collected from each well, and nor-leucine was added for standardization. An equal amount of 20% sulfosalicylic acid was added and mixed well. The mixture was then centrifuged at 7000*g* for 10 min at 4 °C, and the supernatant was collected as the amino acid extract from the media.

For the analysis of intracellular amino acids, after rinsing the myotubes with phosphate-buffered saline (PBS), they were gently harvested in ice-cold PBS using a cell lifter to minimize mechanical damage and immediately centrifuged at 1000*g* for 5 min at 4 °C. The supernatant was discarded, and 250 μL of fresh PBS containing nor-leucine for standardization was added to each tube before bead crashing at 4000 rpm for 30 s. Twenty-five μL of crude lysate was mixed with an equal amount of 2× RIPA buffer and further homogenized. After centrifugation at 10,000*g* for 10 min at 4 °C, the supernatant was collected, and the protein concentration was measured to correct the final intracellular amino acid concentration. Two hundred μL of crude lysate was mixed with a 4× volume of 99.5% ethanol, centrifuged at 1000*g* for 10 min at 4 °C, and the supernatant was collected to remove protein and stored as an amino acid extract from the cells.

A portion of each amino acid extract was dried and dissolved in 20 μL of ethanol/water/triethylamine (2:2:1) solution. After drying, the pellet was dissolved in 20 μL of an ethanol/water/triethylamine/phenyl isothiocyanate (7:1:1:1) solution and incubated at room temperature for 30 min. After drying, the pellet was dissolved in PTC-Amino Acid mobile phase A (FUJIFILM Wako Pure Chemical Co., Osaka, Japan) and filtered. Standard samples (amino acid mixture standard solution type H, l-tryptophan, and l-glutamine, FUJIFILM Wako Pure Chemical Co.) were processed using the same method. The samples were analyzed using HPLC system (Elite LaChrom, (two L-2130 pumps, L-2455 diode array detector, L-2350 column oven, and L-2200 autosampler, D-2000 Elite software; Hitachi High-Tech Co., Tokyo, Japan) and column (Wakopak Wakosil-PTC, 4 mm × 250 mm, FUJIFILM Wako Pure Chemical Co.). The temperature of the column was maintained at 40 °C. Amino acid mobile phase A and B (FUJIFILM Wako Pure Chemical Co.) were used at a flow rate of 1.0 mL/min for the following gradient method: 0–20 min, B = 0–70%; followed by 10 min 100% B and 40 min 100% A for column equilibration.

### RNA extraction and real-time PCR

Total RNA was extracted from the myotubes using Sepasol-RNA I Super G (Nacalai Tesque), and the RNA concentration was measured using a NanoDrop Lite (Thermo Fisher Scientific). Using ReverTra Ace qPCR RT Master Mix with gDNA Remover (TOYOBO Co., LTD., Osaka, Japan), 0.5 μg of total RNA was reverse transcribed into cDNA. Gene expression levels were quantified using TB Green Premix Ex Taq II (Takara Bio Inc., Shiga, Japan) with QuantStudio 1 real-time PCR system (Thermo Fisher Scientific). The gene expressions were quantified using the calibration curve method, and *rps17* was used as a control housekeeping gene. Housekeeping gene *rps17* expression was not differ significantly among the experimental groups. Table 1 shows the sequences of the primers used in this study.

**Table 1:** Primer sequences for qPCR.

Gene	Forward primer (5′–3′)	Reverse primer (5′–3′)
*Lat1*	ATGACGCTGATGTACGCCTT	AGGCTTCTTGAATCGGAGCC
*Lat2*	GAACCACCCGGGTTCTGAC	CGCTGACCAATCCGATCTCT
*Lat3*	GGAGATCCGGAGTGTGGAAG	GACTCTGTCCCCGACAAACTT
*Lat4*	CGCTGTGTTGGAAAACCTCC	CTCTGGCTTCGTACACAGGT
*Snat1*	CATGTACTTCCTGACGGCCA	ATGAGAATGTCGCCTGTGCT
*Snat2*	TTACGGACACGTGGAATCGG	ACGGAACTCCGGATAGGGAA
*Snat3*	CGACGTGCTATCCAGCAGAT	GTTGGGGGCGAAGATAACCA
*Snat4*	CTGGTCATCCTCGTGCCTAC	TGAGATAAAACGCAGCCGGA
*Snat6*	AACTCAGCCGAGAGGACTTC	ACTGAAGCTTCCGACCACAG
*Snat7*	TGGCGCTCTTTATCCCTGAC	CTTGAATGAGGCACAGCCCT
*Myh7*	CAATGAGACGGTGGTGGGTT	CTTGCCTTTGCCTTTGTCCG
*Myh2*	GTAGTGGTGGAGCTGCCAAG	AATGAGGATGGGTGCTCCTG
*Myh1*	GTGGGGCTGTACCAGAAGTC	TTTCTTTCCACCACCGCCAC
*Myh4*	TTTAAAGCCGGCCTGTTGGG	ATCAGGTACCCTCTGCACAC
*Myh3*	CGGAGGAGCTGTTAGCTACG	CATCACAGCCCCTGTCAGTT
*Rps17*	CCGGGTCATCATCGAGAAGT	GCGCTTGTTGGTGTGGAAGT

### Immunofluorescence staining

The myotubes were fixed with ice-cold methanol for 15 min at 4 °C and then blocked with 5% goat serum for 60 min at room temperature. They were subsequently incubated overnight at 4 °C with an anti-MYH3 antibody (1:200, ab124205, Abcam, Cambridge, UK) diluted in 0.1% BSA/0.3% Triton X-100. After washing, the myotubes were incubated with goat anti-rabbit fluorescent-conjugated secondary antibody at room temperature. Finally, the myotubes were mounted with ProLong Gold Antifade Mountant with DNA Stains DAPI (P36941, Thermo Fisher Scientific, MA, USA) and observed as previously described. Five images were randomly taken from each well, and the diameter of at least 70 randomly selected myotubes within the images was measured using Image J software (National Institutes of Health, Bethesda, MD, USA).

### Western blotting

After each intervention, myotubes were homogenized in RIPA buffer containing cOmplete Mini protease inhibitor cocktail and PhosSTOP phosphatase inhibitor cocktail (11836153001 and 4906845001, respectively, Sigma-Aldrich, St. Louis, MO, USA) and analyzed as previously described with slight modifications^37,38^. Briefly, the homogenates were centrifuged at 10,000*g* for 10 min at 4 °C, and the protein concentration of the supernatants was determined using a BCA protein assay kit (23225, Thermo Fisher Scientific). Samples were diluted in 3× Red Loading Buffer and boiled at 95 °C for 5 min. Equal amounts of proteins were then separated on m-PAGEL (2332240, ATTO, Tokyo, Japan). The separated proteins were transferred to polyvinylidene difluoride membranes, and the membranes were blocked with Bullet Blocking One for Western Blotting (13779-01, Nacalai Tesque) for 5 min at room temperature. The membranes were then incubated overnight at 4 °C with following primary antibodies: puromycin (1:3000, MABE343, Merck Millipore, Darmstadt, Germany), phospho-p70S6K (1:1000, Thr389, #9205, Cell Signaling Technology (CST), Danvers, MA, USA), total-p70S6K (1:1000, #2708, CST), phsopho-rpS6 (1:3000, Ser240/244, #5364, CST), total-rpS6 (1:3000, #2217, CST), phospho-4EBP1 (1:1000, Thr37/46, #2855, CST), total-4EBP1 (1:1000, #9466, CST), phospho-Akt (1:1000, Ser473, #9271, CST), total-Akt (1:1000, #9272, CST), Atrogin-1 (1:3000, ab168372, Abcam, Cambridge, UK), MuRF-1 (1:1000, sc-398608, Santa Cruz Biotechnology, TX, USA), K48 linkage-specific polyubiquitin (1:1000, #8081, CST), phospho-ULK1 (1:1000. Ser757, #14202, CST; 1:1000, Ser555, #5869, CST), total-ULK1 (1:1000, #8054, CST), p62/SQSTM (1:3000, PM045, Medical & Biological Laboratory, Nagoya, Japan), or LC3 (1:2000, #2775, CST). After washing, the membranes were incubated with the appropriate secondary antibodies (115-035-206, Jackson ImmunoResearch Inc., PA, USA, or #7074, CST) for 1 h at room temperature. Bands were visualized using Immobilon Forte Western HRP Substrate (WBLUF0500, Merck Millipore) and detected using the ChemiDoc Touch Imaging System (Bio-Rad, Hercules, CA, USA). Band intensities were quantified using Image J software. Coomassie Brilliant Blue staining was used to verify equal loading between lanes and to normalize the results.

### Statistical analysis

In experiments examining concentration-dependent effects, one-way ANOVA and Dunnett’s multiple-comparison testing were performed. In experiments examining the single effects of JPH203, unpaired *t*-test was performed. In experiments that combined JPH203 treatment with other interventions, a two-way ANOVA was performed. If an interaction was observed, Šidák multiple-comparison testing was performed. The analysis was conducted in GraphPad Prism 10 (San Diego, CA, USA). For all experiments, n indicates the number of wells analyzed, with each well treated as an independent biological replicate. No additional averaging of technical replicates was applied. All values were expressed as mean ± SEM with individual plots. Statistical significance of differences was defined as *P* < 0.05.

## Results

### JPH203 treatment increases protein synthesis in C2C12 myotubes

**Fig. 2:**
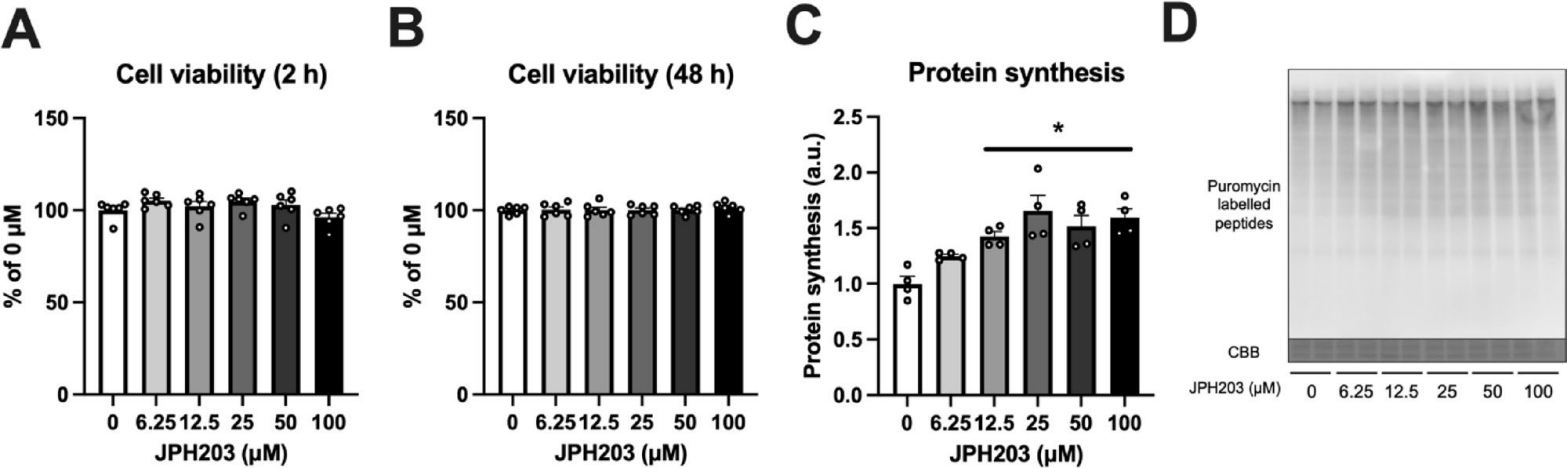
JPH203 treatment augments protein synthesis in C2C12 myotubes. Myotubes were exposed to 0–100 μM JPH203. Cell viability at 2 (**A**) and 48 h (**B**) after exposure to JPH203. Protein synthesis in myotubes (**C**) and representative bands (**D**) 2 h after exposure to JPH203. Data are expressed as mean ± SE. *P < 0.05 versus 0 μM JPH203 (control).

We first investigated the dose responses of JPH203 on cell viability and protein synthesis in C2C12 myotubes. Since previous research has shown that JPH203 inhibits amino acid uptake immediately after exposure, and that changes in muscle protein synthesis associated with amino acid exposure occur within a few hours, protein synthesis was measured after 2 h^38,39^. JPH203 treatment at concentrations of 0–100 μM did not influence cell viability after 2 h (Fig. 2A). The effects of long-term exposure (48 h) also examined, but no change was observed (Fig. 2B). On the other hand, a concentration-dependent increase in protein synthesis in C2C12 myotubes was observed, and there was a significant increase at 12.5–100 μM (Fig. 2C,D). These results suggest that JPH203 treatment increases protein synthesis without influencing cell viability in C2C12 myotubes.

### JPH203 treatment reduces amino acid uptake and increases the mRNAs of some amino acid transporters

**Fig. 3:**
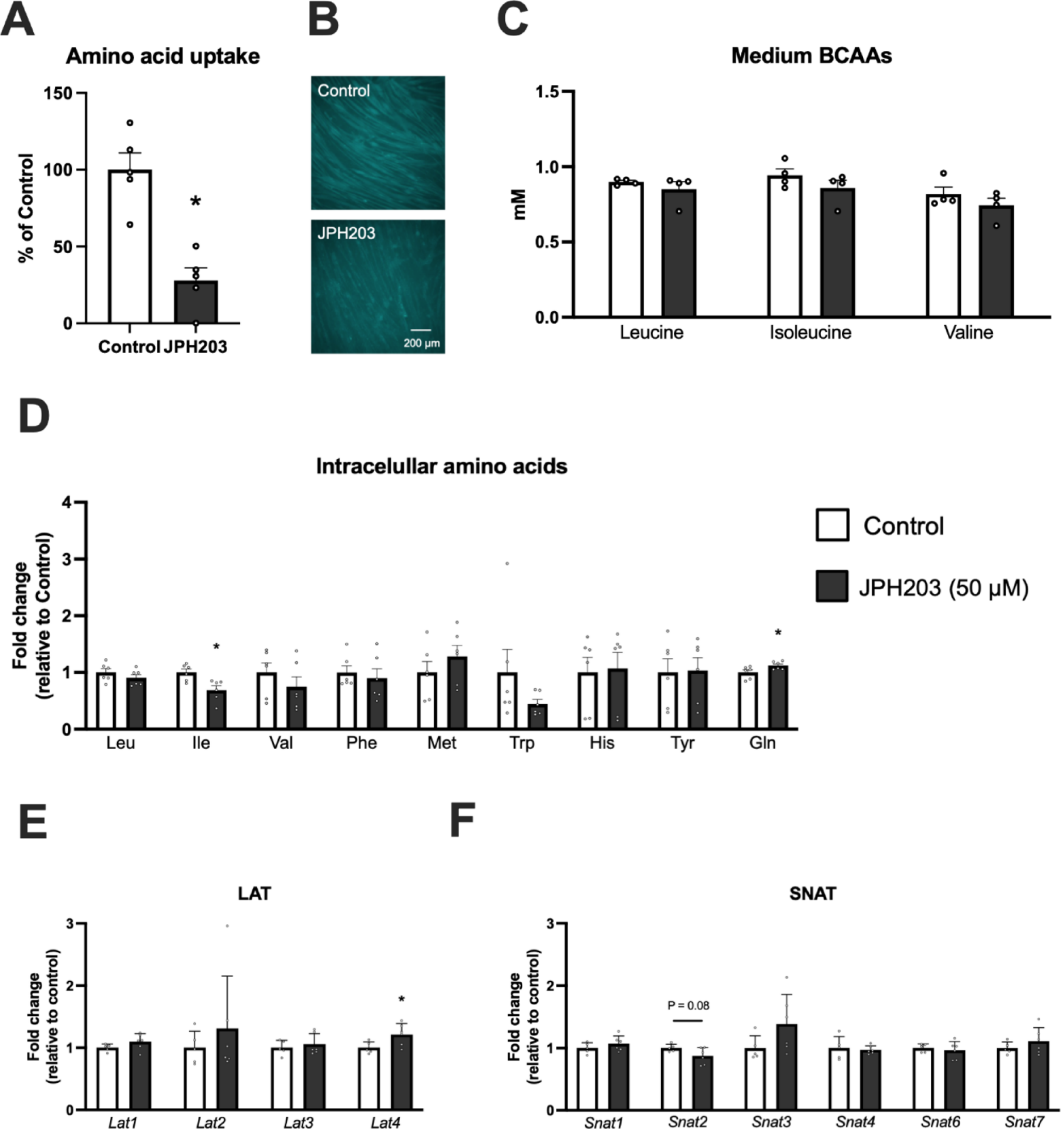
Changes in amino acid uptake, extra- and intracellular amino acid concentration, and gene expression of LAT and SNAT families following JPH203 treatment in C2C12 myotubes. Myotubes were exposed to 50 μM JPH203. Amino acid uptake 5 min after exposure to JPH203 (**A**) and its representative image (**B**, scale bar = 200 μm). BCAA concentrations in culture media 2 h after exposure to JPH203 (**C**). Intracellular amino acids transported by LAT1 2 h after exposure to JPH203 (**D**). Gene expression of LAT and SNAT family members 2 h after exposure to JPH203 (**E**,**F**). Data are expressed as mean ± SE. *P < 0.05 versus 0 μM JPH203 (control).

Based on the above results, we decided to use 50 μM JPH203 as the intervention, as the mean response appeared to plateau around this concentration, which affected protein synthesis without compromising cell viability. Although JPH203 treatment did not affect the concentration of BCAAs in the culture medium after 2 h, it did reduce the uptake of amino acids immediately (5 min) after treatment (Fig. 3A–C). The changes in intracellular amino acid were then examined and we found that intracellular isoleucine was decreased, and glutamine was increased (Fig. 3D). Among the LAT and SNAT family members that have been confirmed to be expressed in mice (except for SNAT8, which expression was not able to detect), only LAT4 mRNA showed a significant increase after JPH203 treatment (Fig. 3E,F). These results suggest that JPH203 was effective in reducing amino acid uptake, altering intracellular amino acid concentrations, which may be accompanied by compensatory increase in LAT4 expression.

### Changes in myotube diameter and related factors by JPH203 treatment

**Fig. 4:**
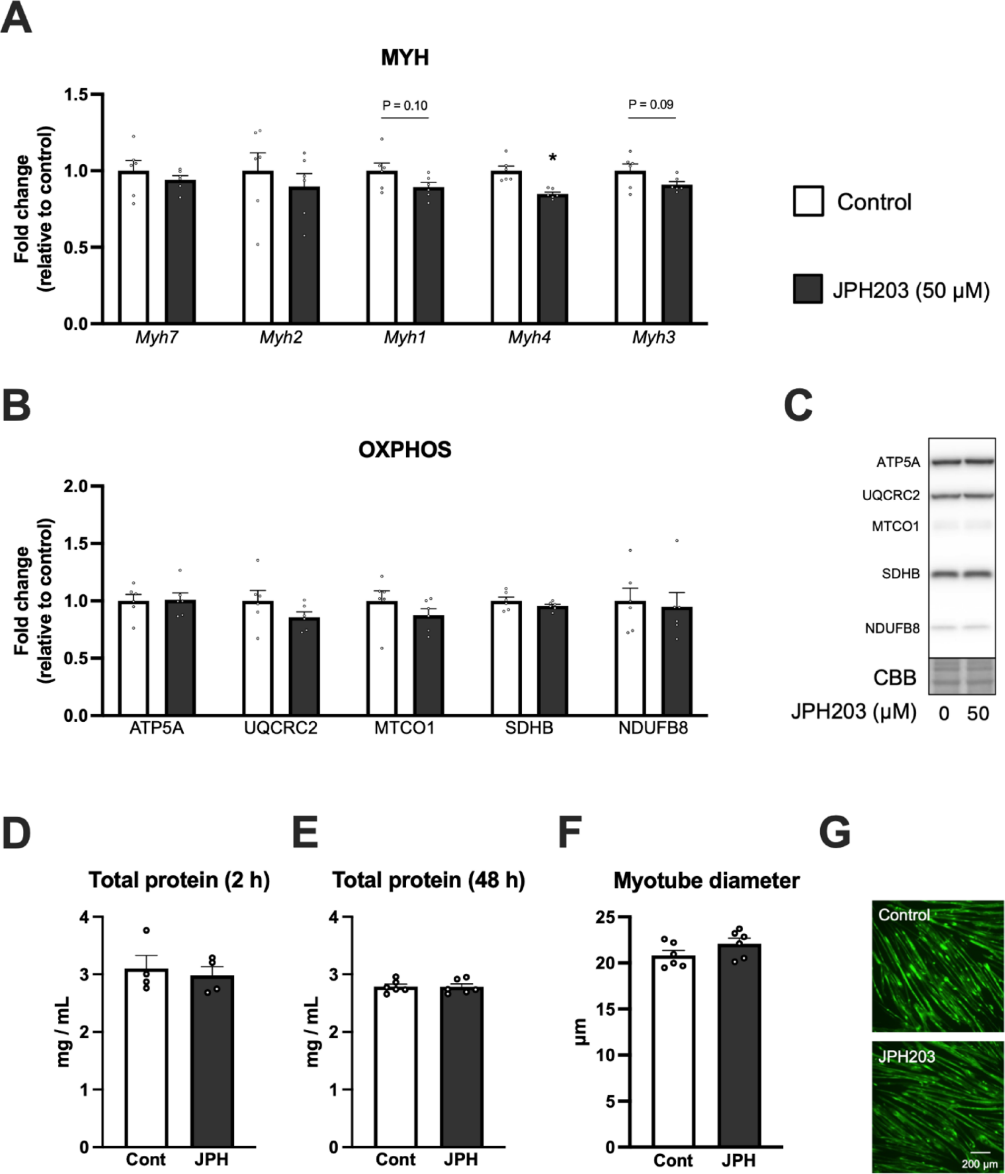
Changes in myosin heavy chain mRNA levels, OXPHOS protein expression, total protein concentration, and myotube diameter caused by JPH203 treatment in C2C12 myotubes. Myotubes were exposed to 50 μM JPH203 for 2 or 48 h. Myosin heavy chain mRNA levels after 2 h JPH203 treatment (**A**), OXPHOS protein expression levels (**B**) and representative bands (**C**) after 48 h JPH203 treatment. Total protein concentration (**D**,**E**) after 2 or 48 h JPH203 treatment. Myotube diameter (**F**) and its representative image (**G**) after 48 h JPH203 treatment. Data are expressed as mean ± SE. *P < 0.05 versus 0 μM JPH203 (control).

We next investigated whether JPH203-induced activation of protein synthesis contributes to muscle growth by examining changes in myotube diameter and related factors. As shown in Fig. 4A, Myh 4 gene expression decreased after 2 h of JPH203 treatment, while other myosin heavy chain genes showed either no change (Myh7 and Myh2) or decreasing trends (Myh1 and Myh3). OXPHOS protein levels were unchanged after 48 h JPH203 treatment (Fig. 4B; Fig. 4C indicates representative blot data). Total protein concentration was not altered at either 2 h or 48 h (Fig. 4D,E). Myotube diameter also remained unchanged after 48 h of JPH203 treatment (Fig. 4F; 4G indicates representative staining image for MYH3). These results indicate that LAT1 inhibition does not promote myotube growth, suggesting that the observed increase in protein synthesis may not contribute to muscle growth.

### Effects on muscle anabolic and catabolic systems by JPH203 treatment

**Fig. 5:**
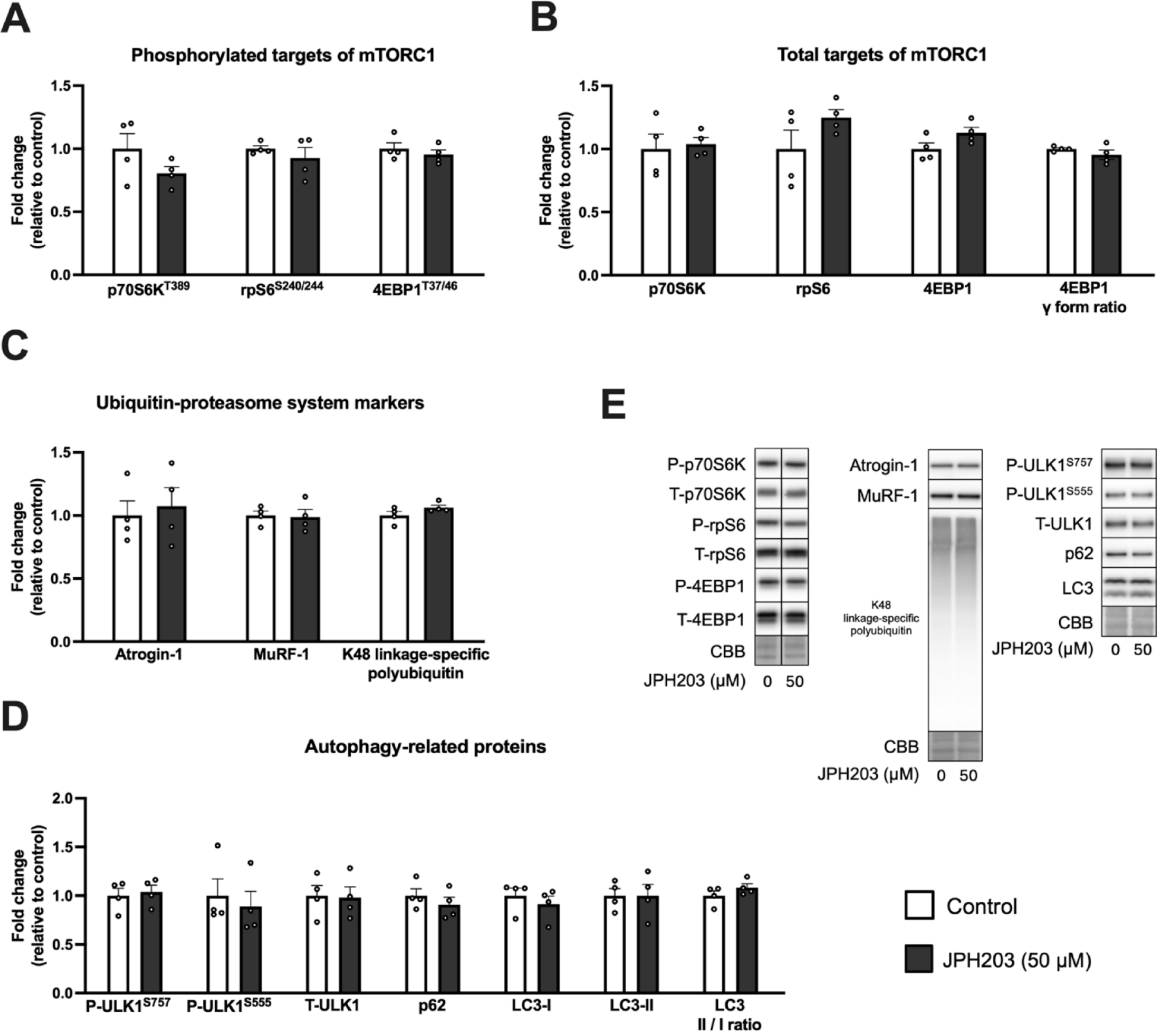
Protein expression of factors involved in mTORC1, ubiquitin–proteasome system, and autophagy. Myotubes were exposed to 50 μM JPH203 for 2 h. Phosphorylated (**A**) and total protein (**B**) expression of mTORC1 signaling factors. Protein expression of ubiquitin–proteasome system- (**C**) and autophagy-related factors (**D**). Representative bands (**E**). Data are expressed as mean ± SE.

To investigate the mechanisms by which JPH203 treatment increases protein synthesis, changes in muscle anabolic and catabolic systems were examined. JPH203 treatment did not affect phosphorylated and total protein expression of the downstream targets of mTORC1, p70S6K (and its downstream target rpS6), and 4EBP1 (Fig. 5A,B; Fig. 5E shows representative bands). Muscle-specific ubiquitin ligases Atrogin-1 and MuRF-1, and K48 linkage-specific polyubiquitin, an indicator of ubiquitinated proteins targeted by the proteasome, were also unchanged (Fig. 5C). Phosphorylated (Ser757 and Ser555) and total protein expression of ULK1, a regulator of the initiation of isolation membrane, were not changed by JPH203 treatment. There were no changes in p62 and LC3-II, a cargo receptor for ubiquitinated proteins that are degraded by autophagy and a marker for autophagosome formation, respectively, and LC3-I and LC3-II/I ratio were also unchanged by JPH203 treatment (Fig. 5D). These results suggest that mTORC1 signaling and major catabolic systems were not activated by JPH203 treatment.

### JPH203 also stimulates protein synthesis in amino acid-free medium

**Fig. 6:**
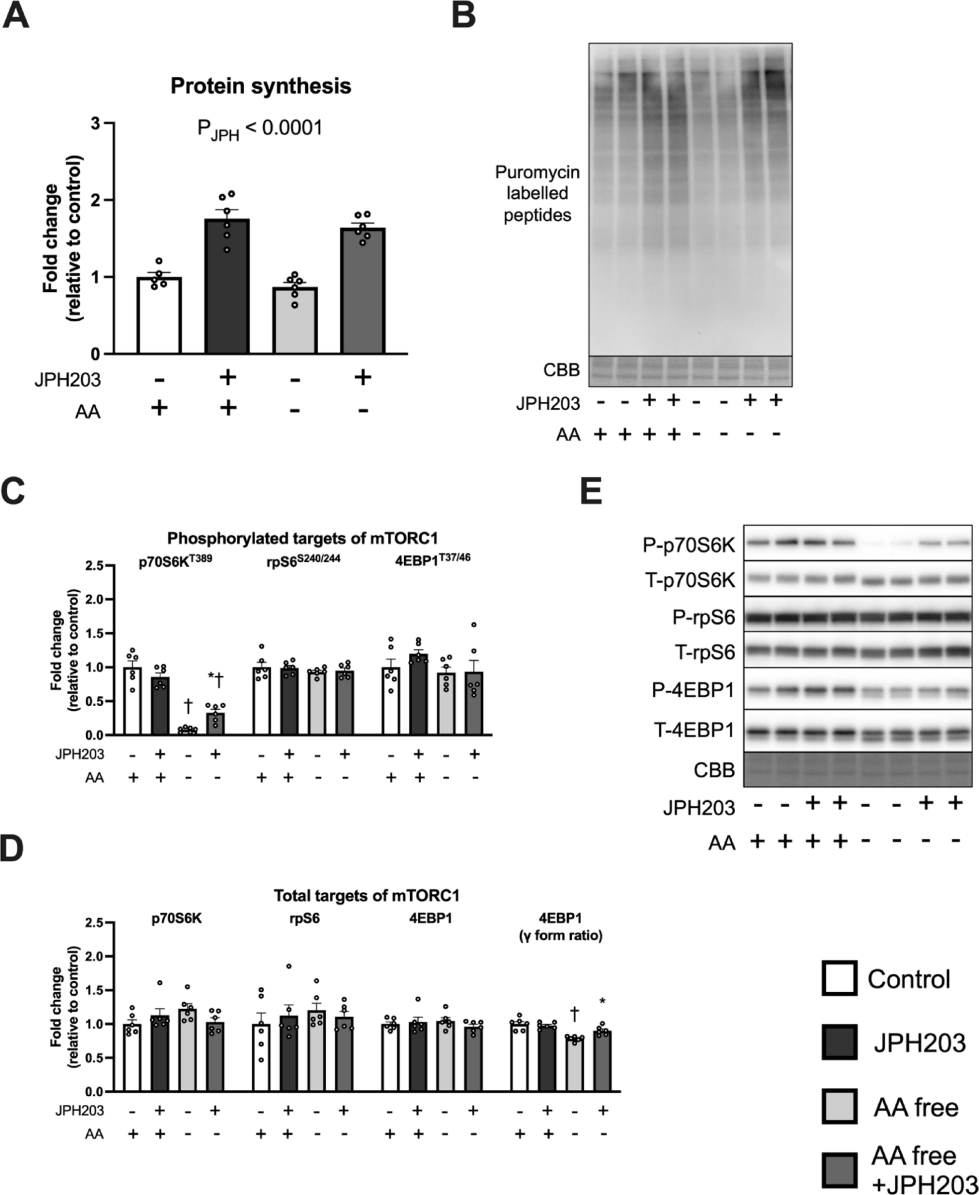
Incubation with amino acid-free media does not suppress JPH203-induced augmentation of protein synthesis in C2C12 myotubes. Myotubes were cultured in amino acid-free medium for 1 h and then exposed to JPH203 for 2 h. Protein synthesis in myotubes (**A**) and representative bands (**B**). Phosphorylated (**C**) and total protein (**D**) expression and representative bands (**E**) of mTORC1 signaling factors. Data are expressed as mean ± SE. Significant interactions were observed in Phospho-p70S6K (T389) and 4EBP1 (γ form ratio). *P < 0.05 versus JPH203 (–) within same media condition. ^†^*P* < 0.05 versus amino acid (–) within same JPH203 treatment condition.

We then examined whether the increase in protein synthesis by JPH203 was due to changes in amino acid supply from culture media. The myotubes were cultured in serum- and amino acid-free medium for 1 h and then treated with JPH203 for 2 h. JPH203 increased protein synthesis in both media (main effect of JPH, Fig. 6A,B). Upon exposure to JPH203 in amino acid-free medium, some activation of mTORC1, indicated by an increase in phosphorylated p70S6K expression and γ-form ratio of total 4EBP1, was observed (Fig. 6C–E). These results suggest that stimulation of protein synthesis by JPH203 treatment is not attributable to the amino acids initially present in the culture media.

### JPH203-induced protein synthesis is not inhibited by rapamycin

**Fig. 7:**
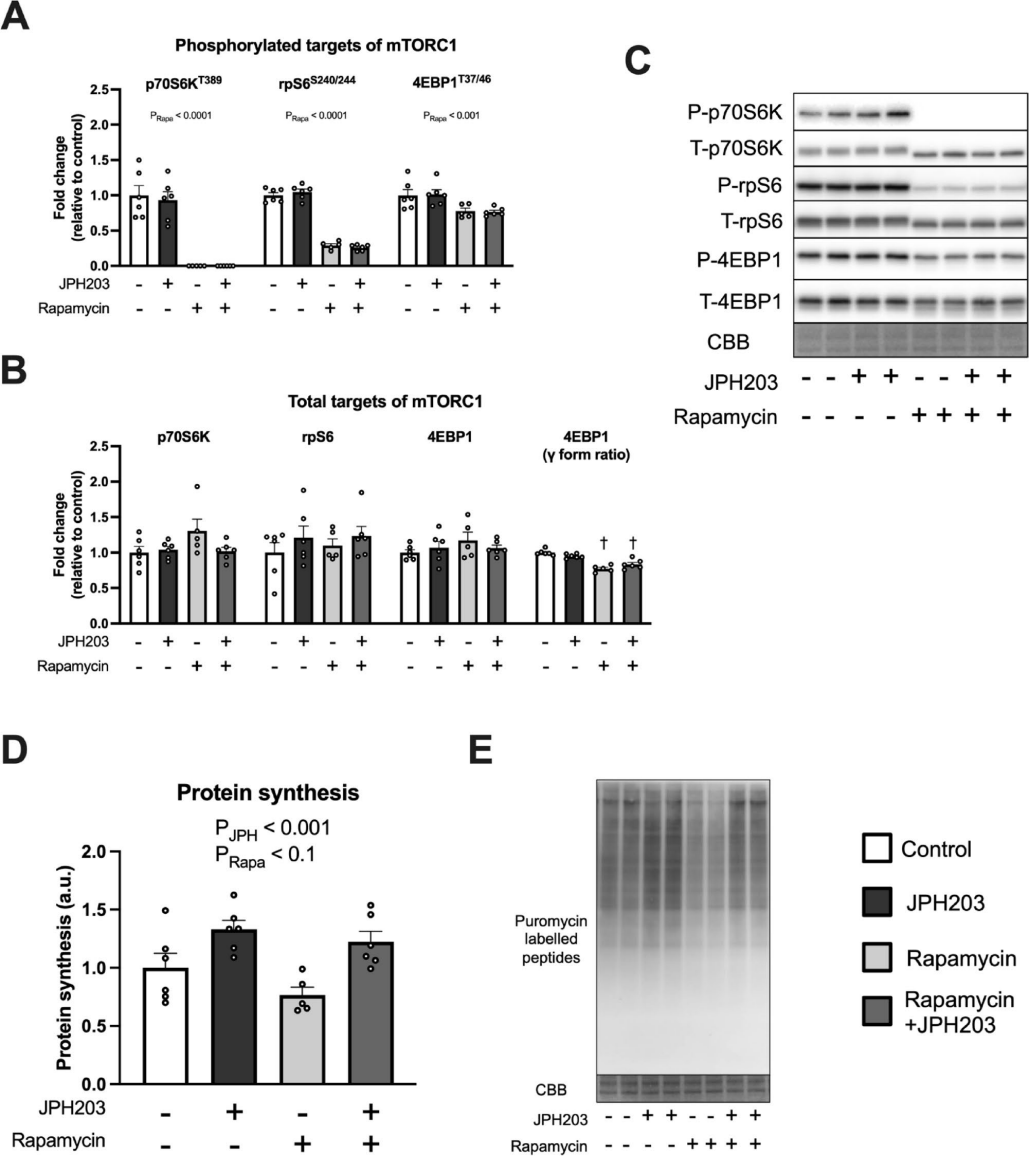
Rapamycin administration does not suppress JPH203-induced augmentation of protein synthesis in C2C12 myotubes. Myotubes were exposed to 100 nM rapamycin for 1 h and then exposed to 50 μM JPH203 for 2 h. Phosphorylated (**A**) and total protein (**B**) expression and representative bands (**C**) of mTORC1 signaling factors. Protein synthesis in myotubes (**D**) and representative bands (**E**). Data are expressed as mean ± SE. A significant interaction was observed in 4EBP1 (γ form ratio). ^†^*P* < 0.05 versus rapamycin (–) within same JPH203 treatment condition.

**Fig. 8:**
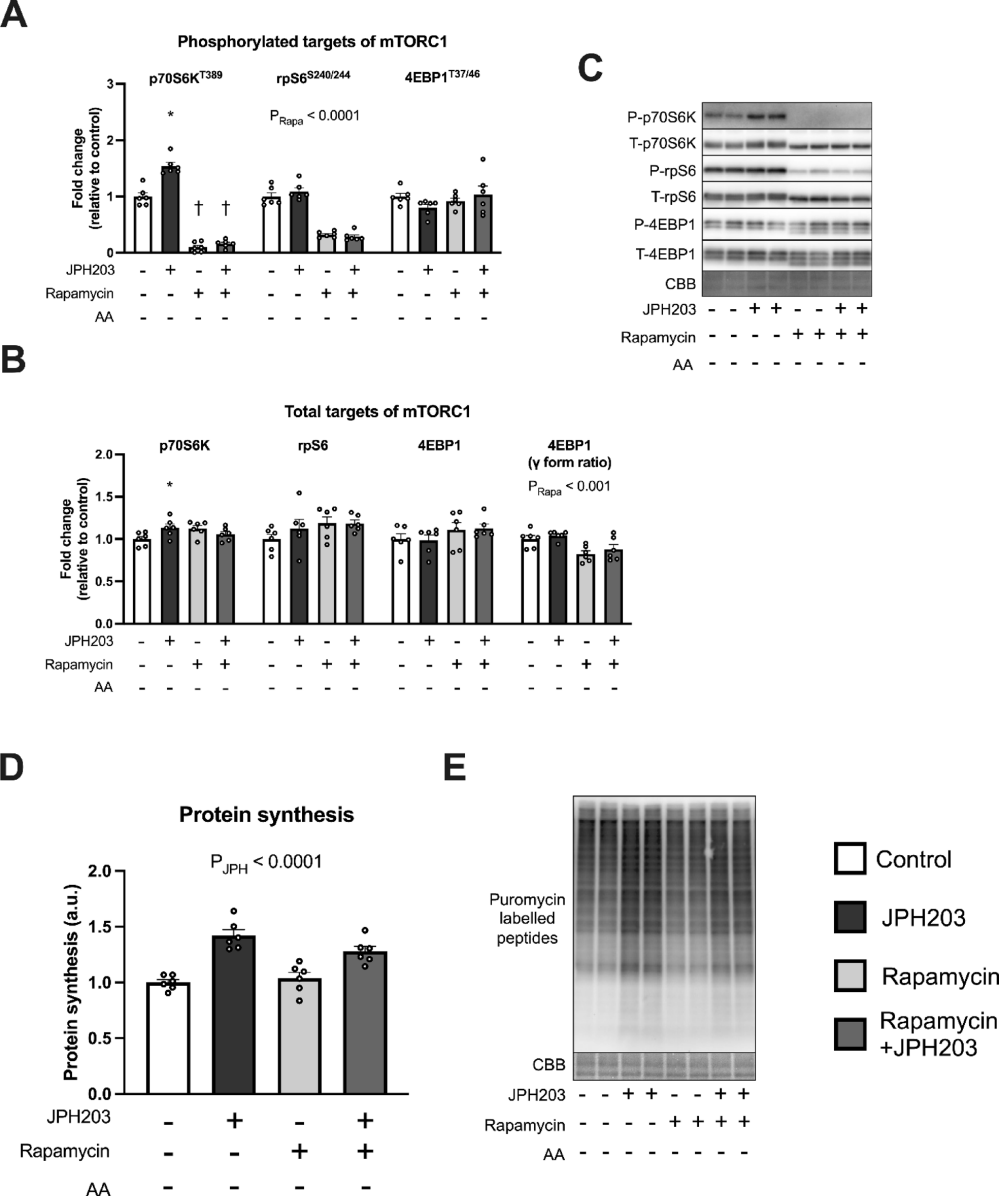
Rapamycin administration does not suppress JPH203-induced augmentation of protein synthesis in C2C12 myotubes cultured in amino acid-free media. Myotubes were cultured in amino acid-free medium for 1 h, then exposed to rapamycin for 1 h, followed by JPH203 for 2 h. Phosphorylated (**A**) and total protein (**B**) expression and representative bands (**C**) of mTORC1 signaling factors. Protein synthesis in myotubes (**D**) and representative bands (**E**). Data are expressed as mean ± SE. *P < 0.05 versus JPH203 (–) within same rapamycin treatment condition. ^†^*P* < 0.05 vs. rapamycin (–) within same JPH203 treatment condition.

Although mTORC1 activation was not detected at 2 h after the JPH203 exposure under amino acid-containing conditions, the observation that JPH203 activates mTORC1 in C2C12 myotubes cultured in amino acid-free medium raises the possibility that mTORC1 may contribute to JPH203-induced protein synthesis even in the presence of amino acids. To examine this possibility, we further tested whether this response could be suppressed by rapamycin, one of the inhibitors of mTORC1. Pre-exposure of rapamycin for 1 h clearly reduced phosphorylated protein expression of p70S6K, rpS6, and 4EBP1 and γ-form ratio of total 4EBP1, suggesting that rapamycin worked properly (Fig. 7A–C). On the other hand, protein synthesis was slightly reduced by rapamycin administration (main effect of Rapa, Fig. 7D,E), but the increase of protein synthesis by JPH203 was not inhibited (main effect of JPH). We also conducted experiments in which rapamycin was added while the myotubes were cultured in amino acid-free medium. Rapamycin administration decreased phosphorylated p70S6K and rpS6 expression and γ-form ratio of total 4EBP1 (Fig. 8A–C). Moreover, rapamycin administration counteracted the increase associated with the JPH203 treatment (Fig. 8A). Although the significant interaction in γ-form ratio of total 4EBP1 could not be detected (Fig. 8B), the inhibition of mTORC1 by rapamycin was sufficient. However, protein synthesis was only increased by JPH203 (main effect of JPH, Fig. 8D,E), suggesting that rapamycin-sensitive mTORC1 is not responsible for the increase of protein synthesis in C2C12 myotubes, regardless of the presence or absence of amino acids in the culture media.

### JPH203-induced protein synthesis is inhibited by AZD8055

**Fig. 9:**
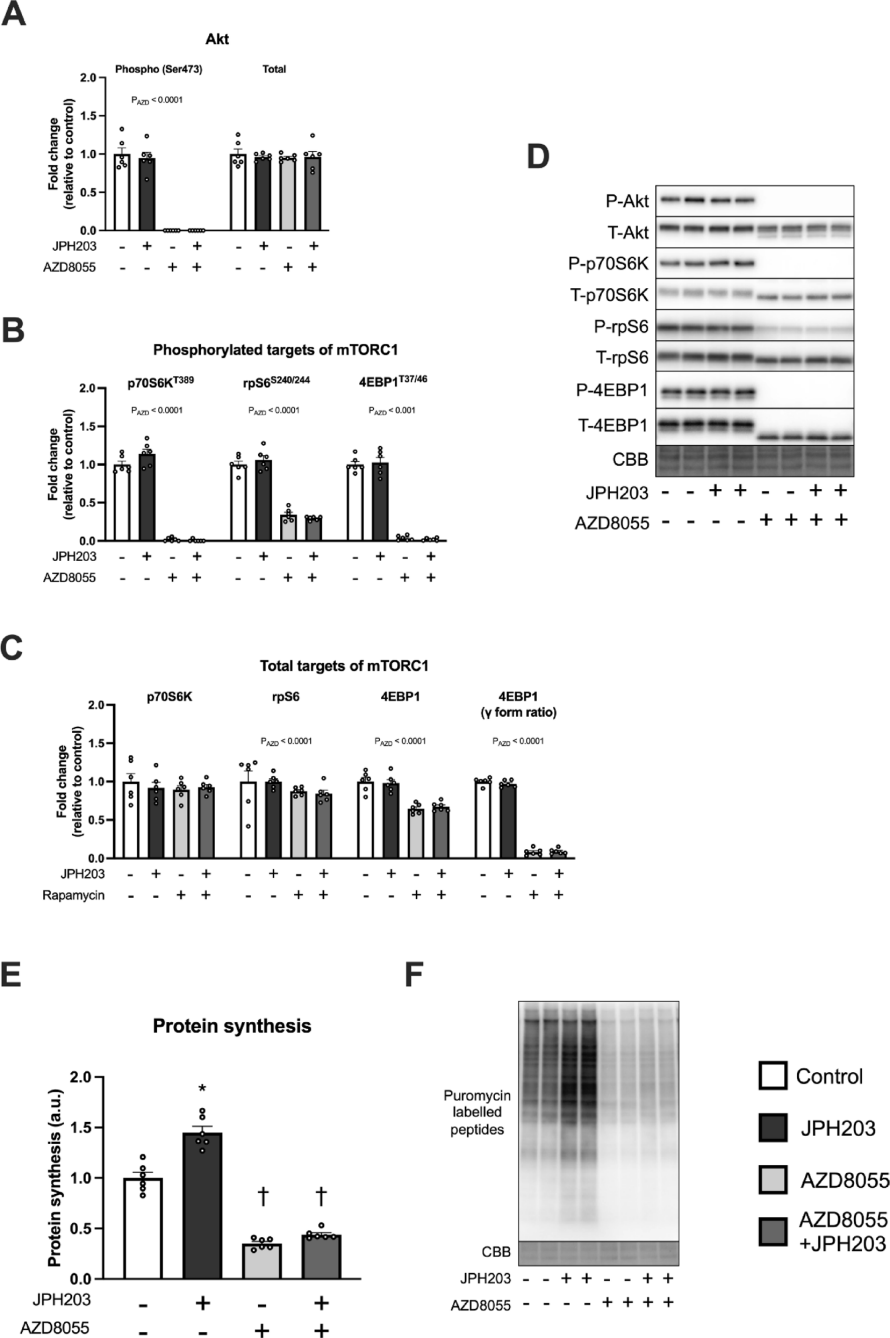
AZD8055 administration suppresses JPH203-induced augmentation of protein synthesis in C2C12 myotubes. Myotubes were exposed to 1 μM AZD8055 for 1 h and then exposed to 50 μM JPH203 for 2 h. Phosphorylated and total Akt expression (**A**), phosphorylated (**B**) and total protein (**C**) expression of factors involved in mTORC1 signaling, and representative bands (**D**). Protein synthesis in myotubes (**E**) and representative bands (**F**). Data are expressed as mean ± SE. A significant interaction was observed in protein synthesis. *P < 0.05 versus JPH203 (–) within same AZD8055 treatment condition. ^†^*P* < 0.05 versus AZD8055 (–) within same JPH203 treatment condition.

To clarify whether the activation of protein synthesis by JPH203 is mTOR-independent or mTOR-dependent other than rapamycin-sensitive mTORC1, we used AZD8055, an ATP-competitive mTOR inhibitor and reported to inhibit both mTORC1 and mTORC2^40^. Pre-exposure of AZD8055 for 1 h clearly reduced both phosphorylated protein expression of Akt (Ser473), a downstream target of mTORC2, and phosphorylated protein expression of p70S6K, rpS6, and 4EBP1 and γ-form ratio of total 4EBP1, suggesting that AZD8055 inhibited both mTORC1 and mTORC2 properly (Fig. 9A–D). Protein synthesis was significantly increased by JPH203 treatment under normal conditions. Although AZD8055 alone decreased protein synthesis, co-treatment with JPH203 abolished the stimulatory effect (Fig. 9E,F). These results, along with the different effects of rapamycin and AZD8055, suggest that the increase in protein synthesis by JPH203 treatment is dependent on rapamycin-insensitive mTOR signaling dependent.

## Discussion

We investigated the effect of JPH203 treatment on protein synthesis and the mechanisms underlying these changes, as well as related factors, in C2C12 myotubes. The main findings are as follows: (1) JPH203 treatment increased protein synthesis, (2) the increase of protein synthesis by JPH203 was not diminished by amino acid deprivation in the medium, and (3) the increase of protein synthesis was diminished not by rapamycin, but by AZD8055. These results suggest that inhibition of LAT1 increased muscle protein synthesis even under amino acid-deprived conditions, and that under amino acid-replete conditions, the effect involves rapamycin-insensitive mTOR signaling.

Inhibition of LAT1 increased protein synthesis in C2C12 myotubes without decreasing cell viability in the present study. This result is reasonable, as a previous study reported that the overexpression of LAT1 decreased protein synthesis in C2C12 myoblasts^26^. We have also obtained consistent results using differentiated cells. As mentioned above, resistance exercise and training, as well as the intake of amino acids, are reported to increase the expression of LAT1 in skeletal muscle^20–25^. The present results raise the possibility that increased LAT1 expression may not necessarily have a purely positive impact on muscle protein synthesis. To clarify the role of LAT1 in regulating skeletal muscle protein synthesis, further studies using in vivo models that avoid compensatory transporter expression are needed.

Myofibrillar proteins are proteins that are responsible for the contraction of skeletal muscle, and usual skeletal muscle hypertrophy is accompanied by an increase in myofibrillar proteins^41,42^. In the present study, we did not directly measure the protein synthesis of the myofibrillar fraction. However, we assessed the gene expression of myosin heavy chains and observed a significant decrease in Myh4 mRNA, along with decreasing trends in Myh1 and Myh3 mRNAs following JPH203 treatment. Longer exposure of JPH203 did not increase protein concentration and myotube diameter. These facts suggest that increase of protein synthesis caused by LAT1 inhibition may not induce muscle hypertrophy. A previous study reported that 24-h exposure to 1 and 2 μM JPH203 treatment increased mitochondrial content in C2C12 myotubes^43^. However, in the present study, OXPHOS proteins were not changed by JPH203 treatment, suggesting that the observed increase in protein synthesis did not involve mitochondrial proteins. This may be due to the difference in the dose of JPH203 used. In a previous proteomics study in several cancer cell lines, JPH203 was reported to activate pathways such as cell cycle and tRNA charging^44^. These pathways may also contribute to the observed increase in protein synthesis, and further studies are needed to identify which proteins are synthesized upon JPH203 treatment and to examine the in vivo responses.

The inhibition of LAT1 increased protein synthesis, but did not alter mTORC1 activity or the major muscle protein-catabolic systems. As discussed in several studies and reviews (for example^45^), leucine has the potency not only to stimulate muscle protein synthesis through the activation of mTORC1, but also to downregulate protein degradation. Thus, the effects observed following JPH203 treatment markedly differ from those typically attributed to leucine. Furthermore, considering that the intracellular leucine concentration was not affected by JPH203 treatment in the present study, it is unlikely that the changes induced by JPH203 treatment are mediated solely by alterations in leucine uptake. mTOR signaling is a central regulator of muscle protein synthesis and is typically sensitive to rapamycin; however, some mTOR-dependent processes are known to be resistant to rapamycin. Resistance exercise-induced protein synthesis is reported to be fully inhibited by AZD8055, but not fully inhibited by rapamycin, even under muscle-specific rictor knockout conditions (i.e., mTORC2 inhibited condition) in rats or mice^46–48^. Therefore, the present results of experiments using two mTOR inhibitors indicate that the augmentation of protein synthesis by LAT1 inhibition is rapamycin-insensitive mTOR-dependent in C2C12 myotubes. In humans, the activation of muscle protein synthesis induced by the intake of EAAs is reported to be rapamycin-sensitive mTOR-dependent^8^. LAT1 is known to transport not only amino acids that can activate mTORC1, such as leucine, methionine, and phenylalanine, but also amino acids that lack this potency^7,18^. At least, these facts suggest that amino acids like leucine, which have the potential to activate mTORC1, are not involved in the increase of protein synthesis observed in C2C12 myotubes in the present study.

Incubation with amino acid-free media did not suppress the augmentation of protein synthesis by JPH203 treatment in C2C12 myotubes in the present study. The detailed mechanisms are currently unclear; however, there are two possible explanations. First, the release of amino acids produced by protein-catabolic systems and their reuptake may be involved. Numerous studies have demonstrated that autophagy, a major protein-catabolic system in skeletal muscle, is activated, and intracellular amino acids are generated through the degradation of proteins in cells cultured in amino acid-free media^49–52^. Some transporters transport amino acids in both directions, and in the LAT family, LAT3 and LAT4 are known to transport leucine by facilitated diffusion^16,52^. Although the amount may be small, amino acid reuptake may have contributed to the JPH203-induced stimulation of protein synthesis, as observed in the presence of amino acids. Another possibility is the function of LAT1 beyond its amino acid transport activity, as demonstrated by a recent study^53^. In that study, knockdown of LAT1 promoted cell mitosis, and no effect was observed with JPH203 in HeLa S3 cells. However, there may be some as-yet-undiscovered effects associated with the substrate competition of JPH203 other than amino acid transport activity. Further investigations are necessary to inhibit protein-catabolic systems and knock down LAT1 in myotubes to clarify these points.

JPH203 treatment increased glutamine in C2C12 myotubes. Since LAT1 is known to transport neutral amino acids into the cell using glutamine as an exchange substrate, the increase of intracellular glutamine concentration as a result of LAT1 inhibition would be reasonable^54^. On the other hand, JPH203 treatment decreased intracellular isoleucine in C2C12 myotubes. A previous study reported that the relative contribution of each amino acid transporter to the uptake of specific amino acids varies depending on the cell type^55^. Therefore, isoleucine may have fewer alternative transport routes than other large neutral amino acids in C2C12 myotubes, potentially explaining the observed decrease in its intracellular concentration. Glutamine is reported not to stimulate mTORC1 in C2C12 myotubes; however, a report is available to help understand the regulation of glutamine on muscle protein synthesis^7^. A previous study reported that glutamine perfusion increased muscle protein synthesis, and a positive relationship was found between protein synthetic rate and intramuscular glutamine concentration under insulin stimulation in rats^56^. Thus, glutamine appears to enhance the activation of muscle protein synthesis induced by other synthetic stimuli. In this study, the concentration of leucine in C2C12 myotubes was maintained even after exposure to JPH203. Although the increase was modest, protein synthesis may have been stimulated by the elevated intracellular glutamine concentration. In addition, the present study found that JPH203 treatment did not affect LAT1 mRNA levels, but did affect LAT4 mRNA levels, which may be due to a change in intracellular amino acid concentration at a later time point. A previous study reported that 10 μM JPH203 treatment increased LAT1 mRNA level in HT29 human colon adenocarcinoma cells^38^. The detailed mechanism for the differential response is unclear, but it may be due to the distinct signaling capacities of each cell line. Further studies are needed to determine the actual contribution of intracellular glutamine concentration to protein synthesis and long-term adaptation.

The present study has some potential limitations. First, we have not examined the detailed changes or involvement of individual LAT and SNAT family members. The uptake of amino acids such as leucine and other BCAAs is not solely regulated by the LAT and SNAT families. To provide a complete picture, a comprehensive analysis is needed, which is beyond the scope of this study focusing on LAT1. Nevertheless, clarifying this point would help elucidate how LAT1 controls muscle protein synthesis, including its complementary relationship with other LAT family members and SNAT. Second, the variability in the concentrations of some amino acids was relatively large, which may be partly due to the low concentrations of these amino acids in the samples. Limitations in analytical sensitivity and the pre-column derivatization method, which has been reported to cause variation in certain amino acids^57^, may have also contributed to this variability. Other measurement methods, such as post-column derivatization or using liquid chromatography-mass spectrometry, may yield more accurate data. Finally, the present results may include off-target effects of JPH203, as has been suggested in previous reports given the relatively high dose used^44^. However, the precise molecular basis of such effects has not been fully elucidated, and our findings should be interpreted with this limitation in mind.

In conclusion, inhibition of LAT1 augmented protein synthesis through rapamycin-insensitive mTOR in C2C12 myotubes. On the other hand, LAT1 inhibition did not increase protein concentration and myotube diameter, suggesting that augmenting protein synthesis through LAT1 inhibition may not lead muscle growth. Our findings contribute to a deeper understanding of the role of LAT1 in regulating skeletal muscle protein synthesis.

## Supplementary Information

Below is the link to the electronic supplementary material.

Supplementary Material 1

## Data Availability

The datasets used and analyzed during the current study are available from the corresponding author on reasonable request.
