# Supplementary material for: Treatment with L-type amino acid transporter 1 inhibitor JPH203 enhances protein synthesis in C2C12 myotubes
Source: Sci Rep. 2025 Nov 19;15:40805. doi: 10.1038/s41598-025-24534-2 (PMC12630651; doi:10.1038/s41598-025-24534-2)

Supplemental information 1

Uncropped band images used for western blot analysis. Loaded samples are noted above the image. The cropped area is indicated by the dotted line. N: not included in the analysis, and M: marker.

Band images in Fig. 1.

Puromycin

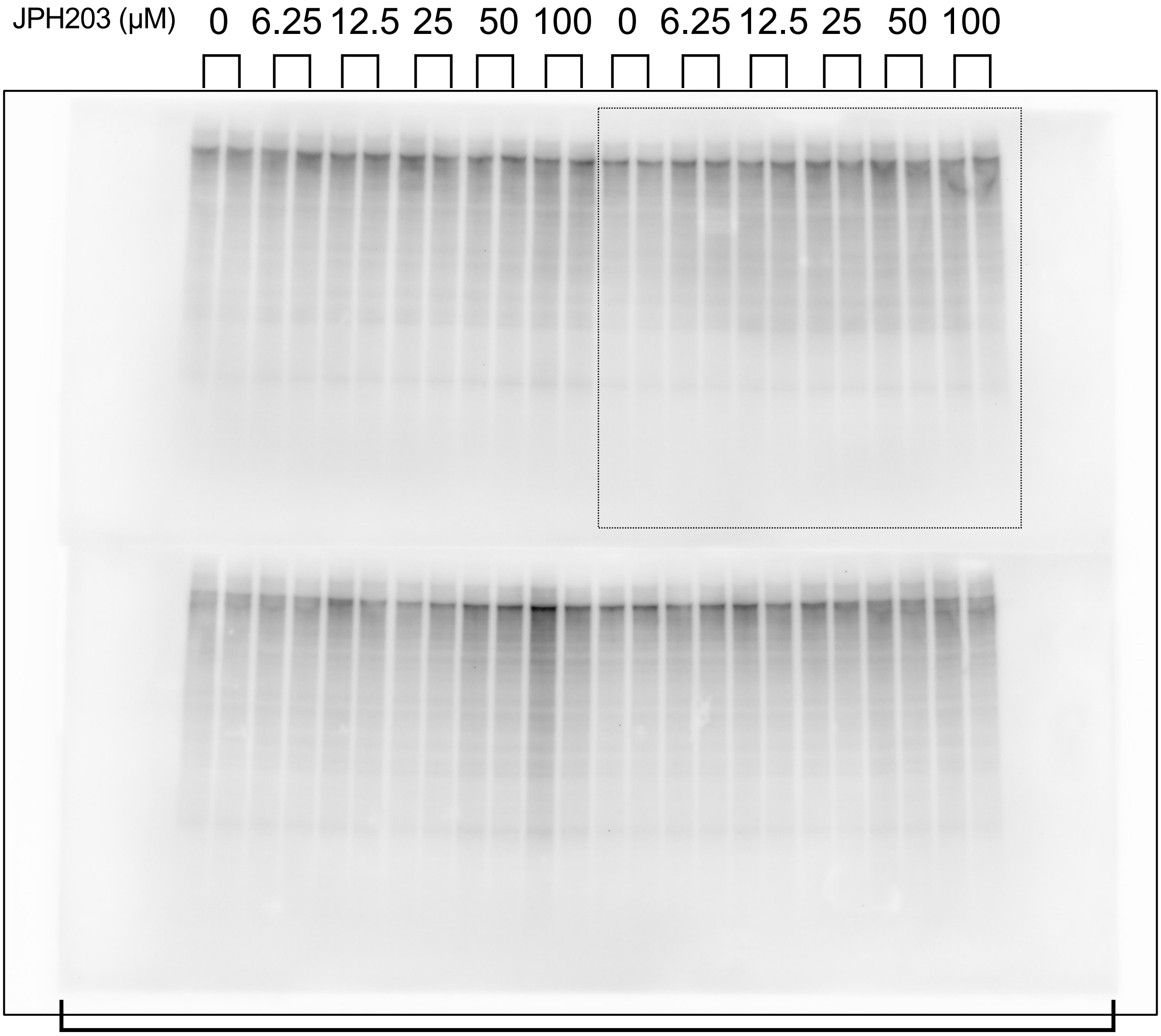

The lower membrane is not included in the analysis.

Band images in Fig. 1 (continued).

Bright field

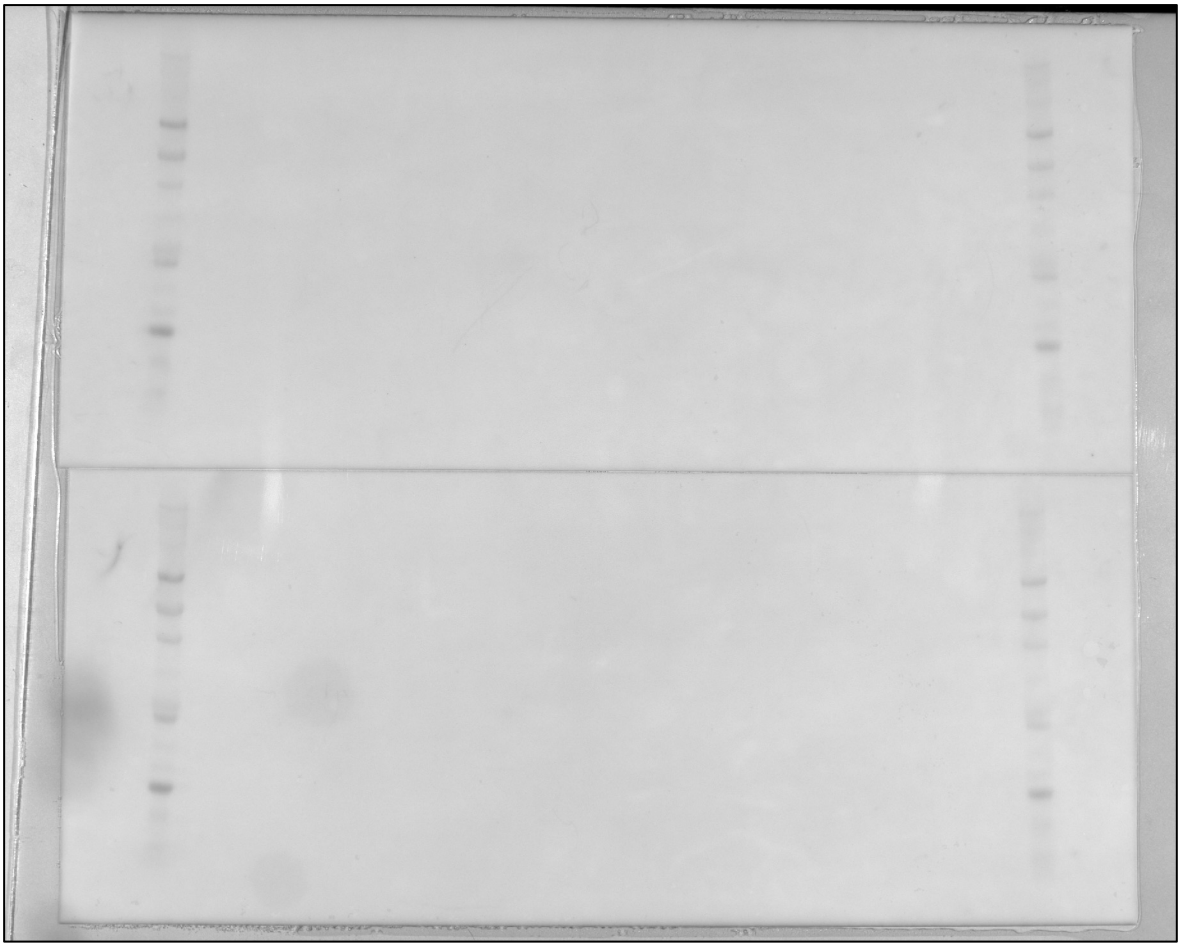

CBB

For
puromycin

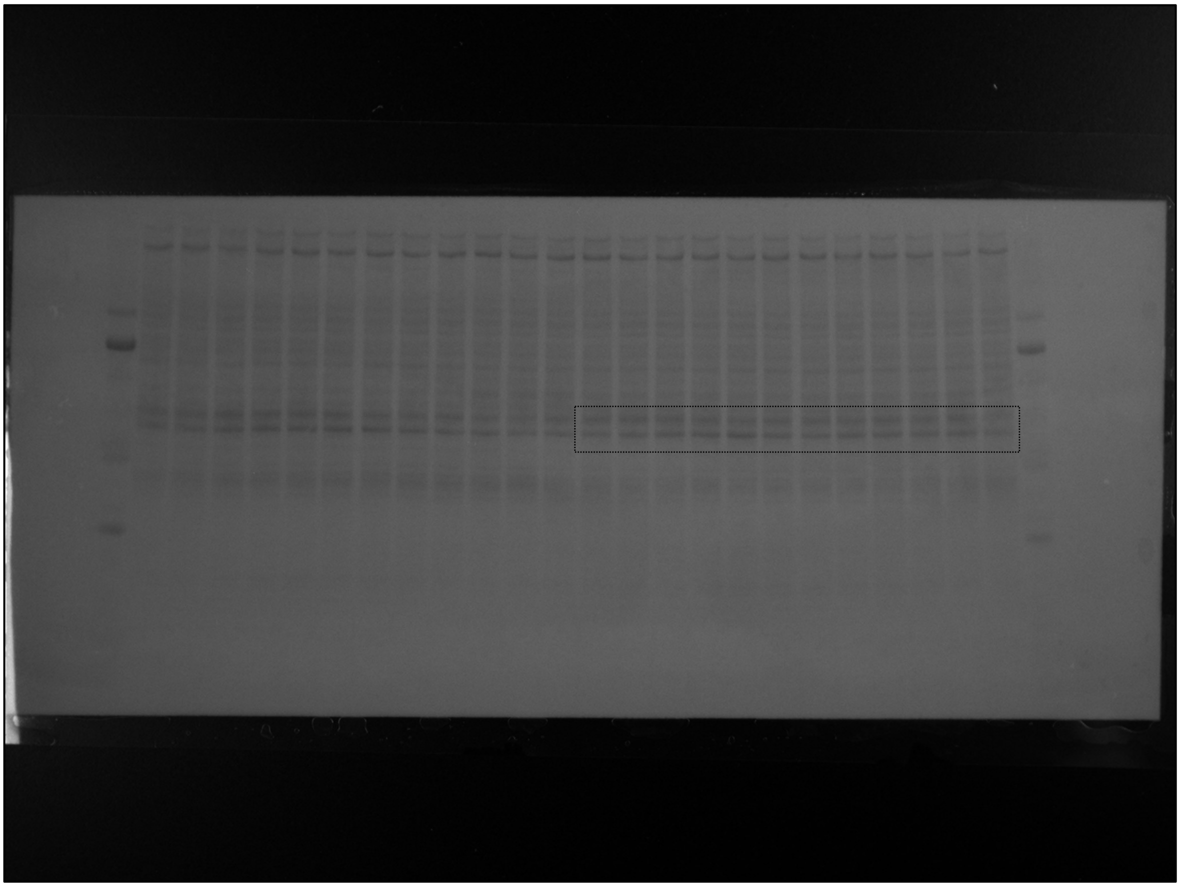

Band images in Fig. 3.

OXPHOS

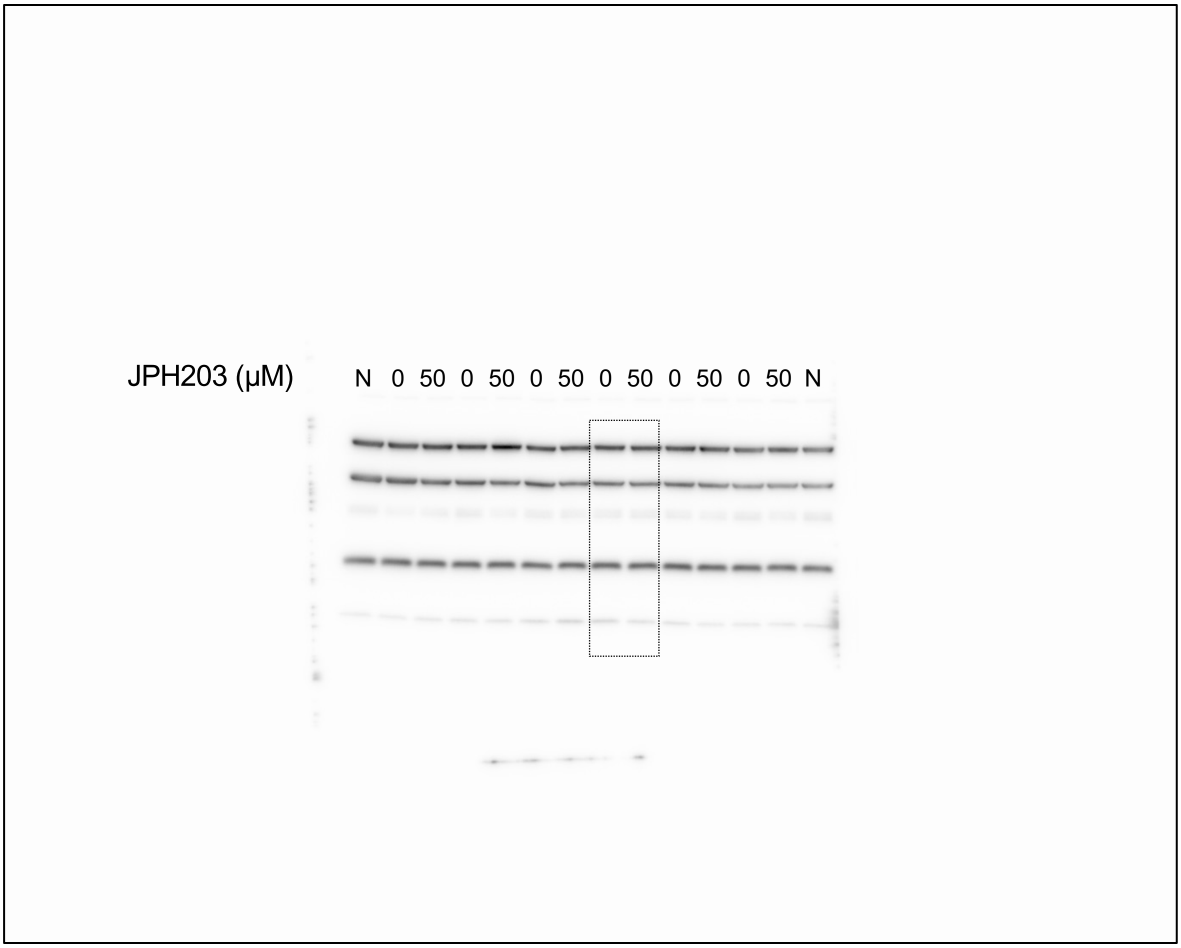

Bright field

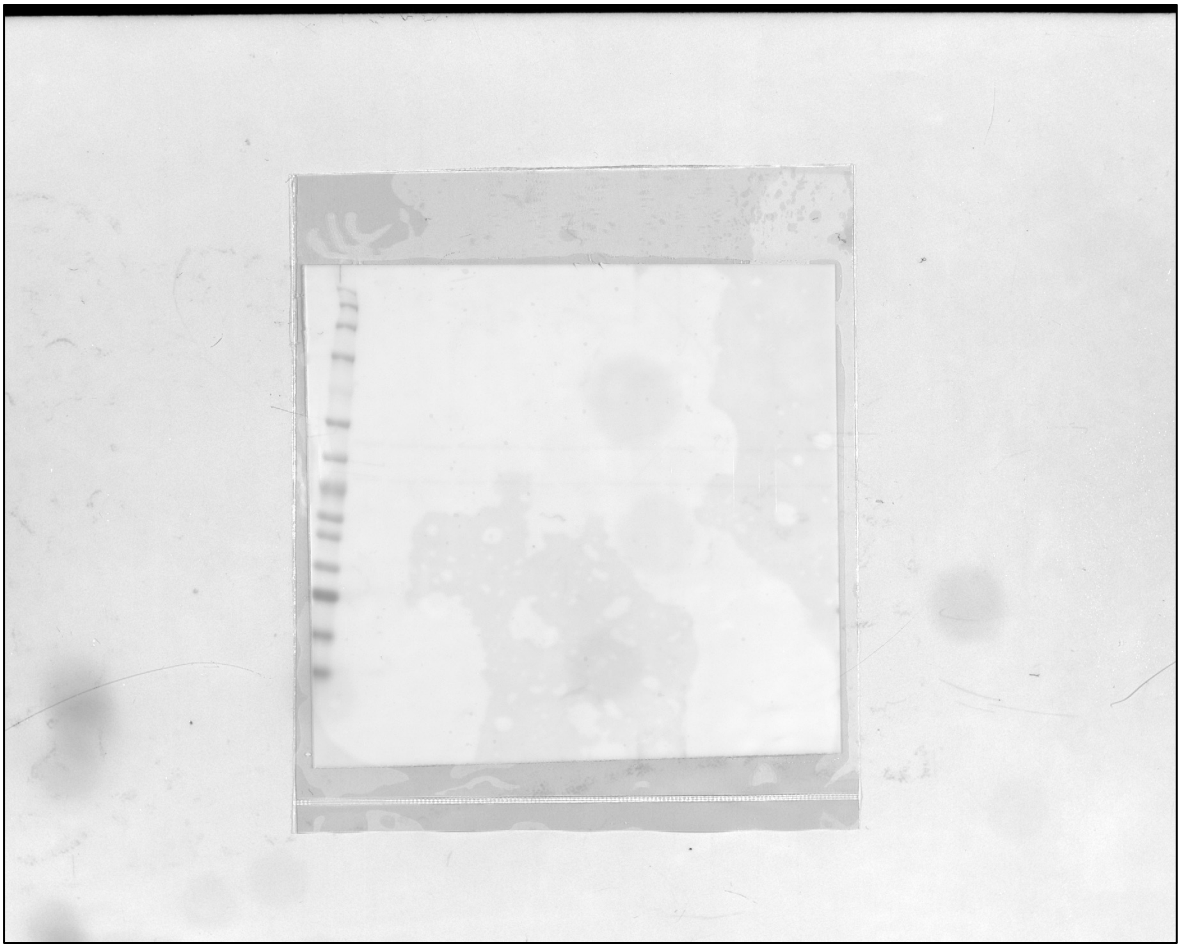

Band images in Fig. 3.

CBB
for
OXPHOS

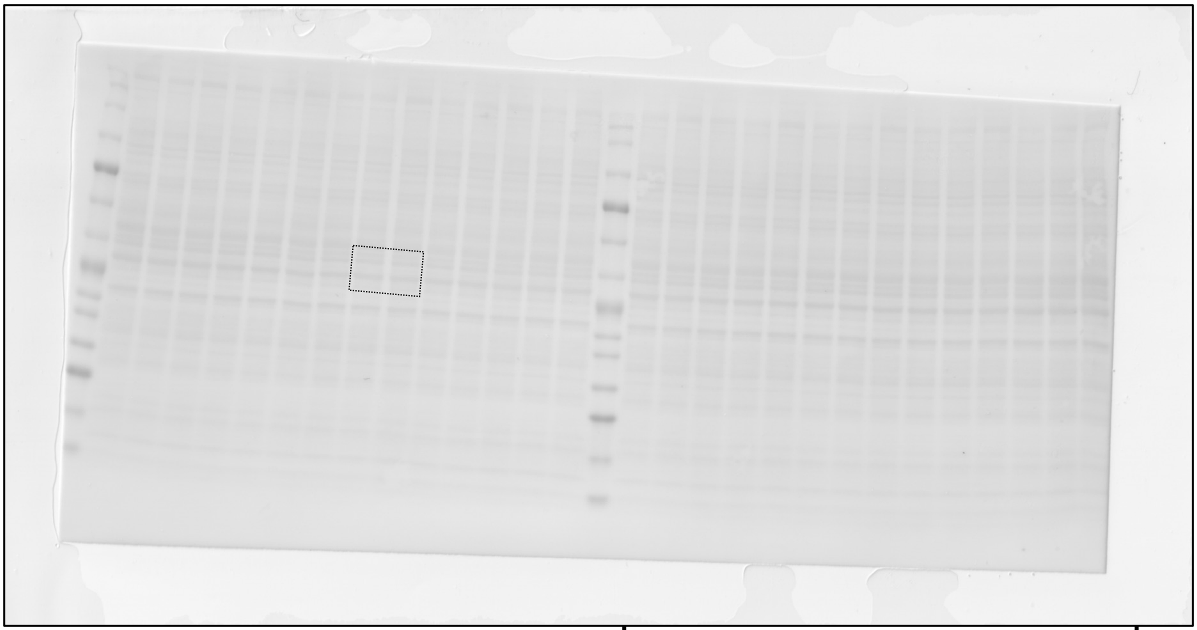

The right side is not included in the analysis.

Band images in Fig. 4.

P-p70S6K

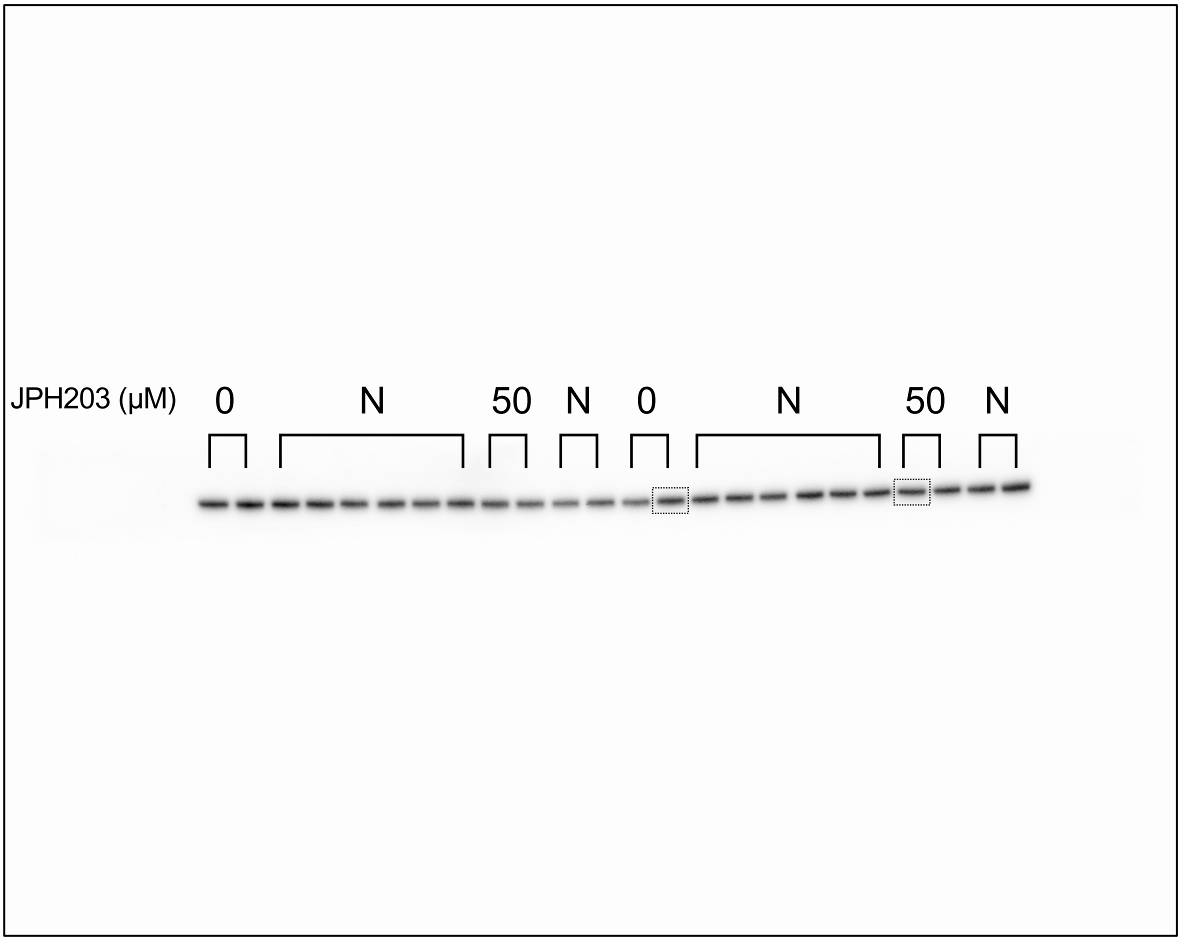

Bright field

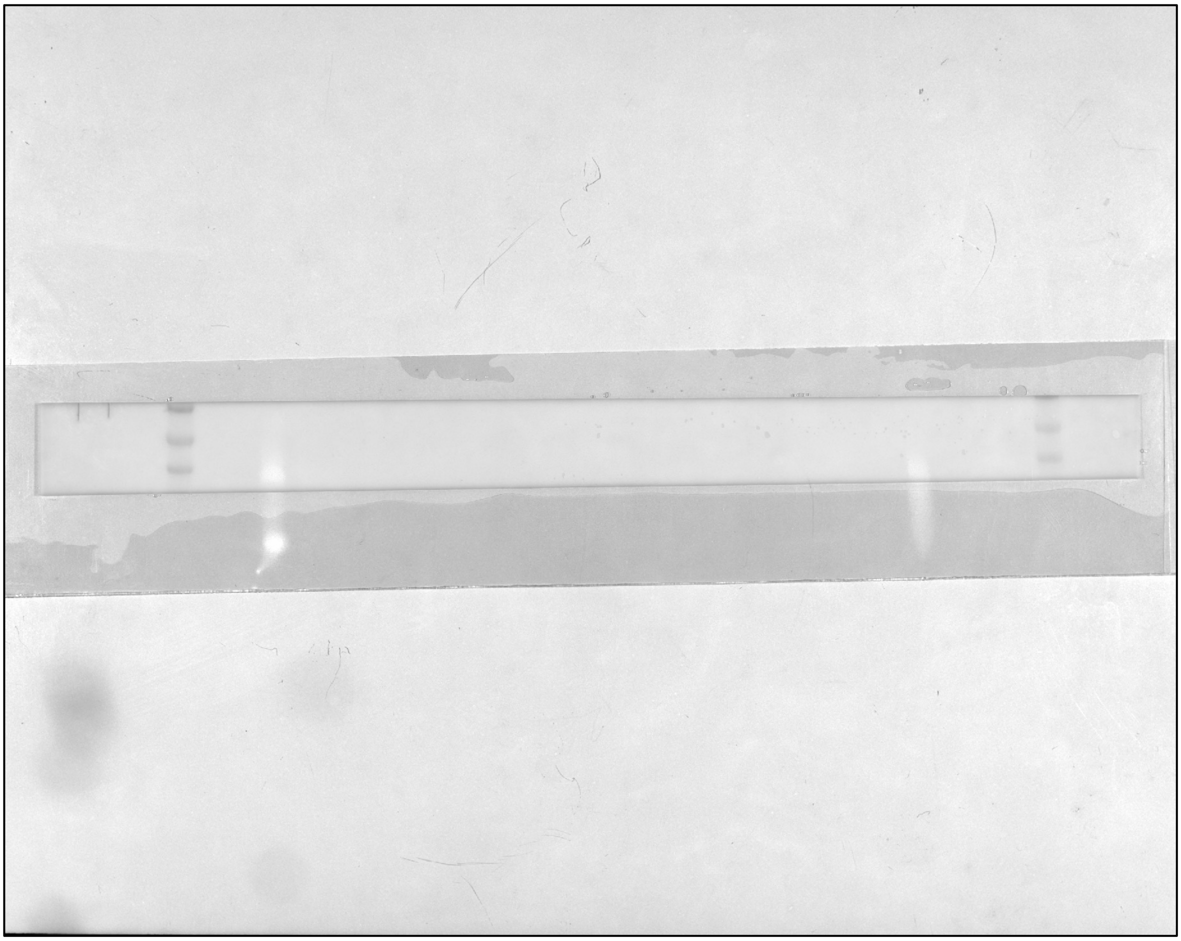

Band images in Fig. 4 (continued).

P-rpS6

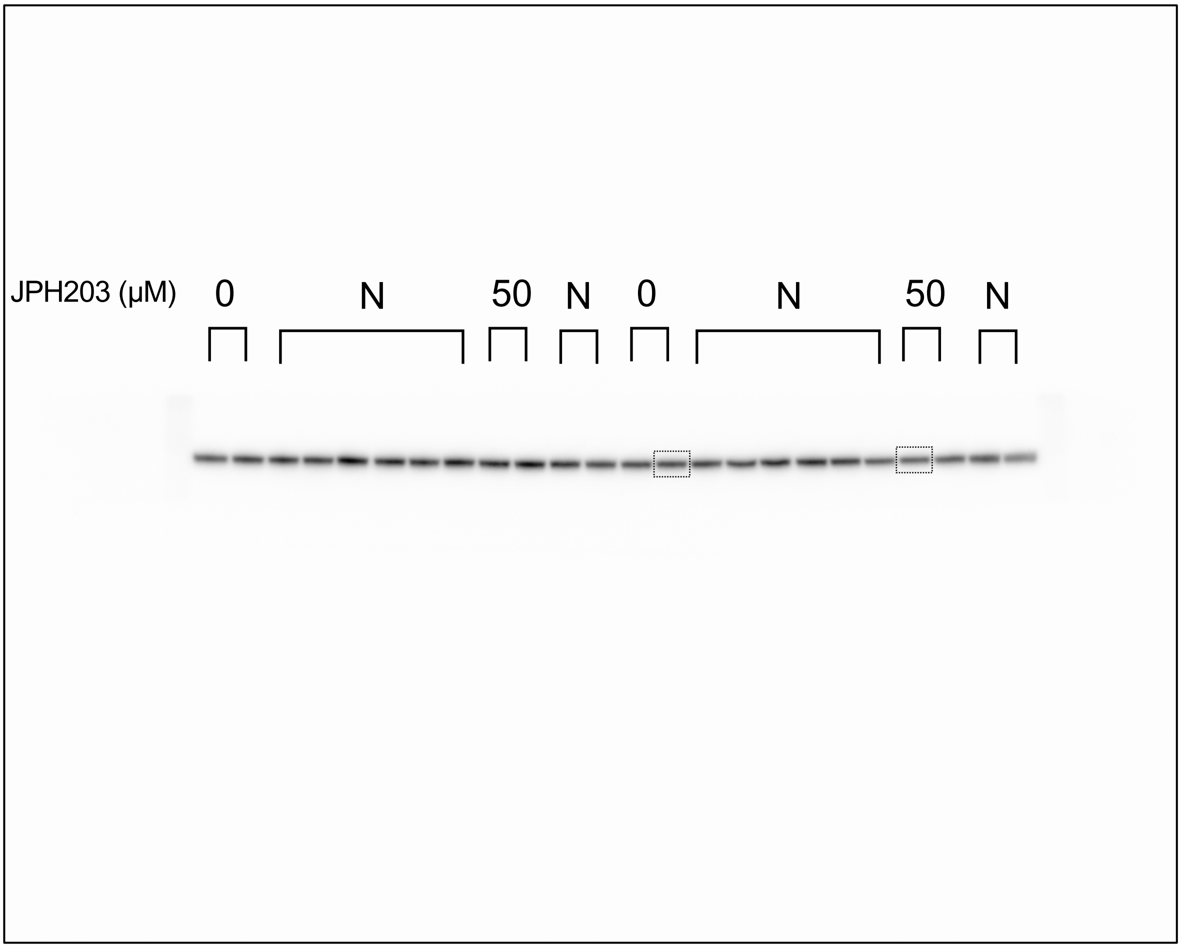

Bright field

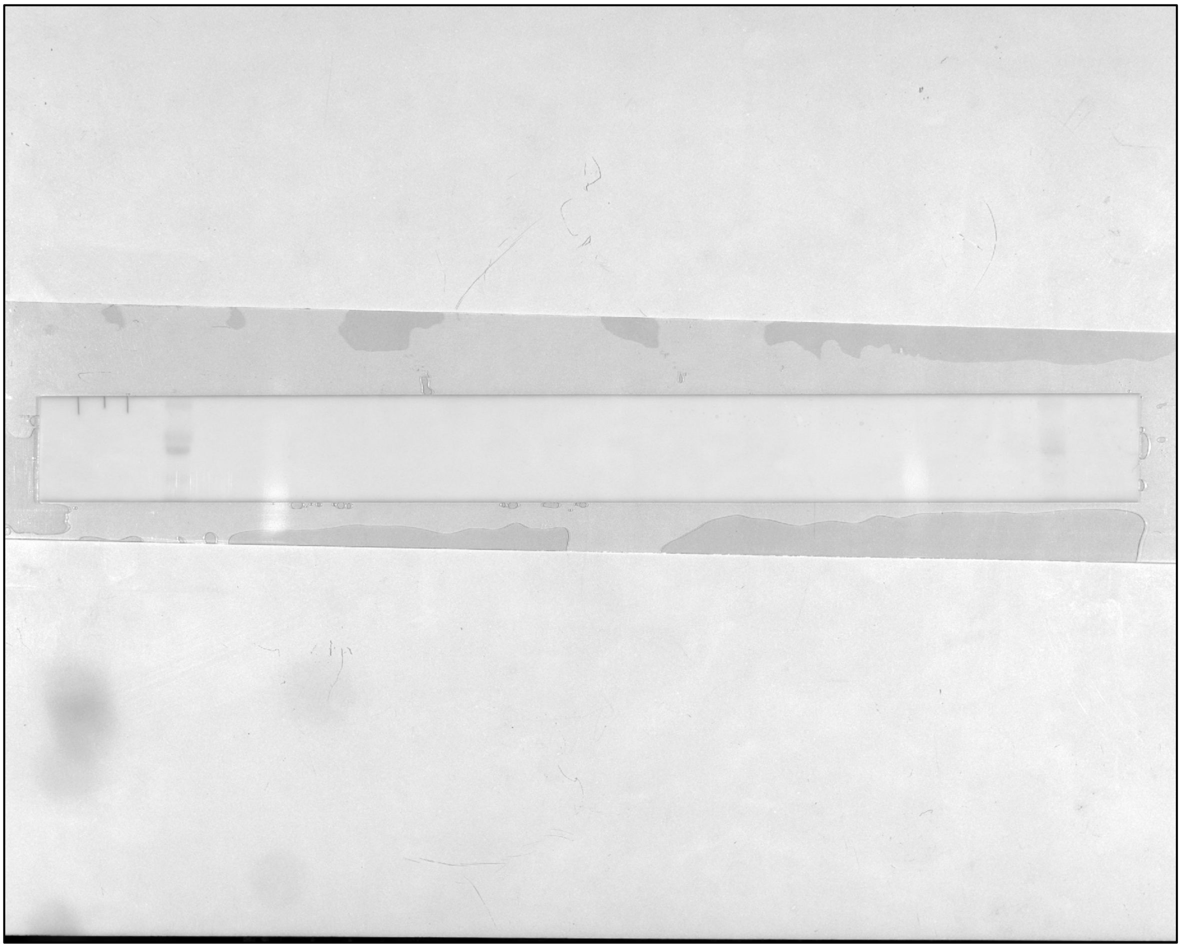

Band images in Fig. 4 (continued).

P-4EBP1

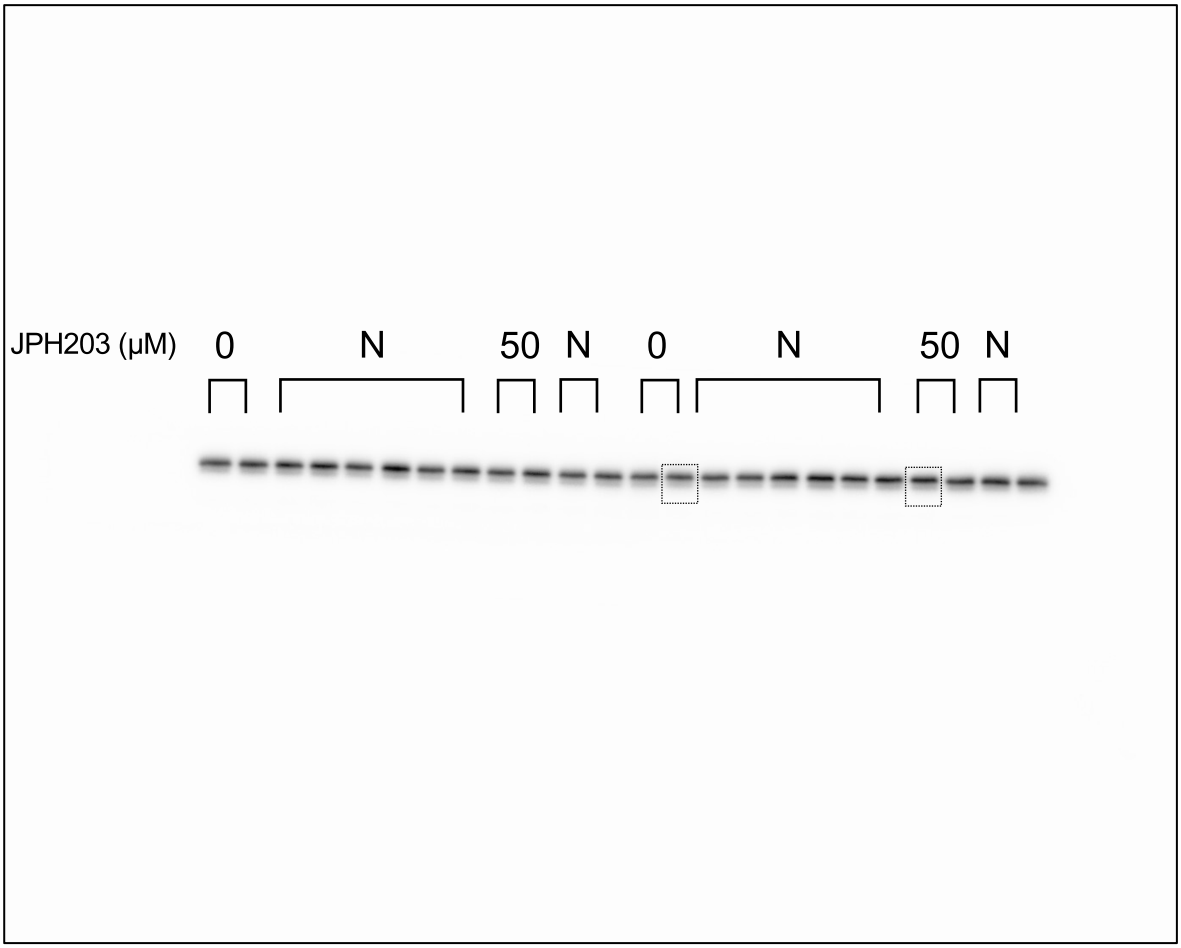

Bright field

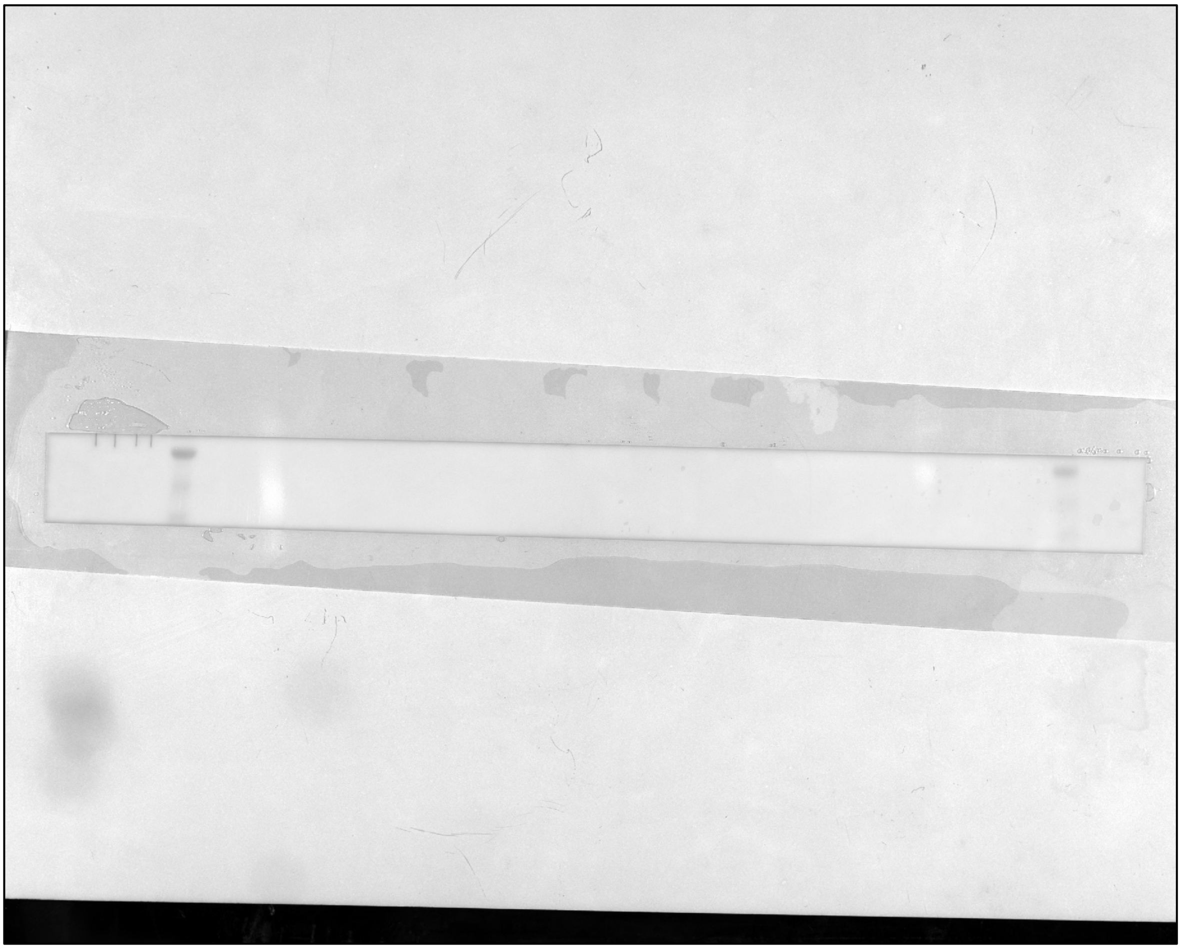

Band images in Fig. 4 (continued).

T-p70S6K

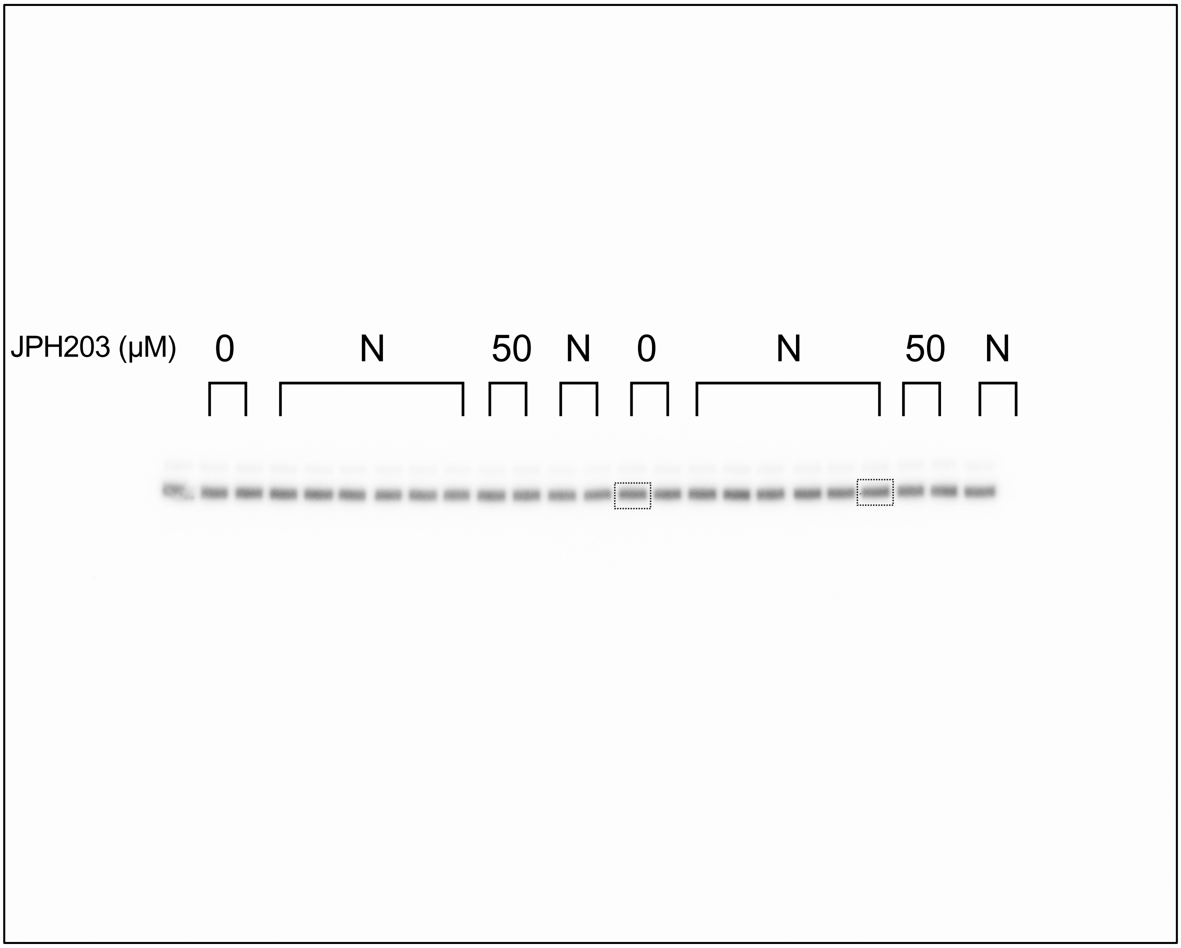

Bright field

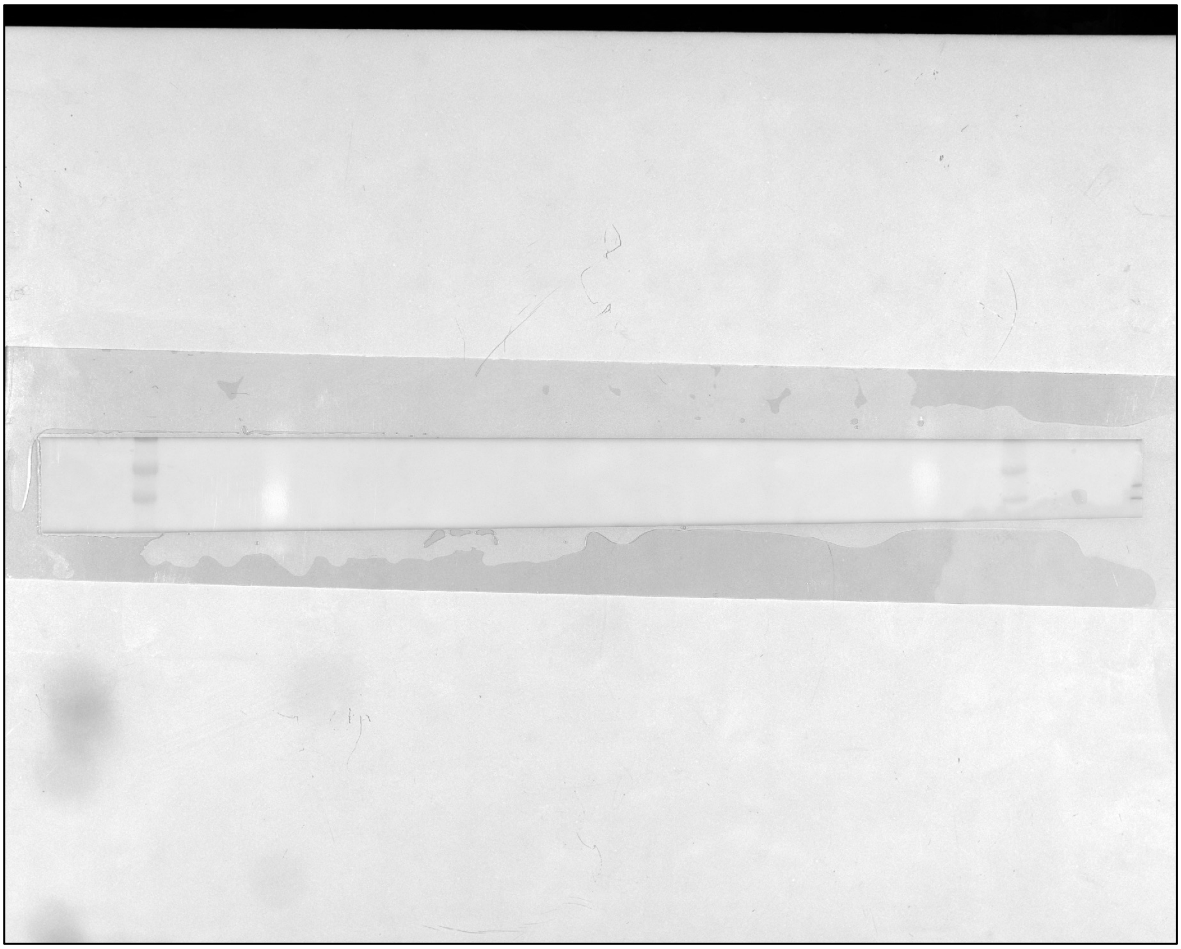

Band images in Fig. 4 (continued).

T-rpS6

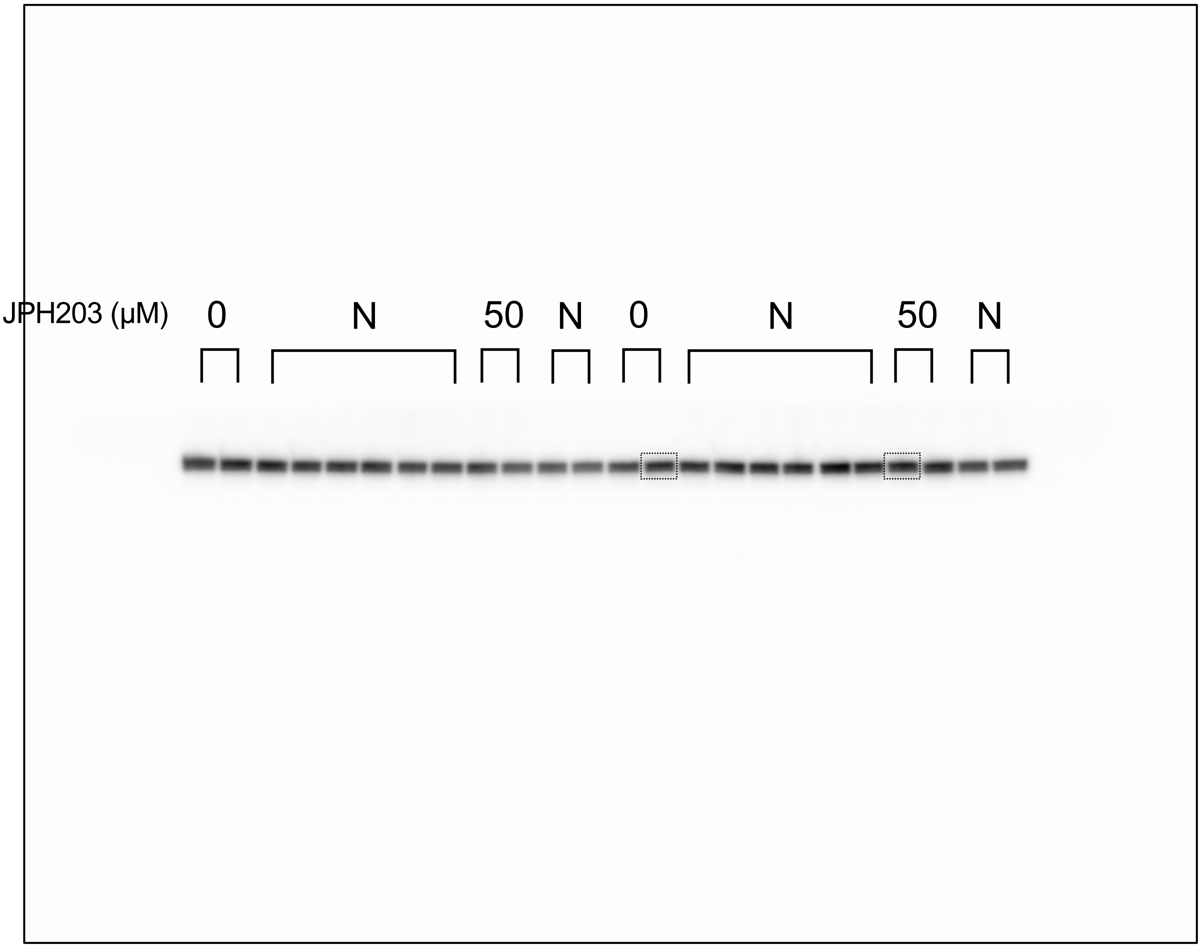

Bright field

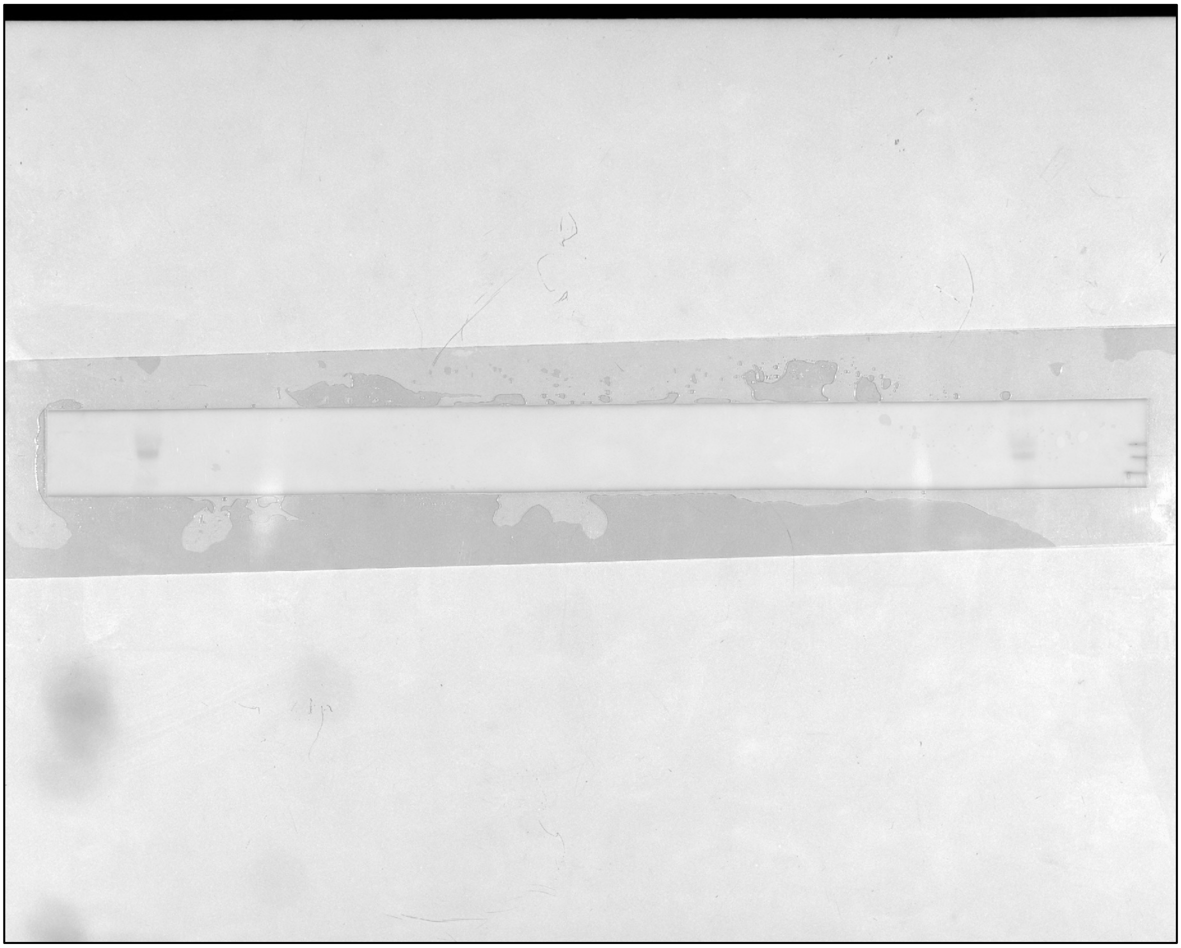

Band images in Fig. 4 (continued).

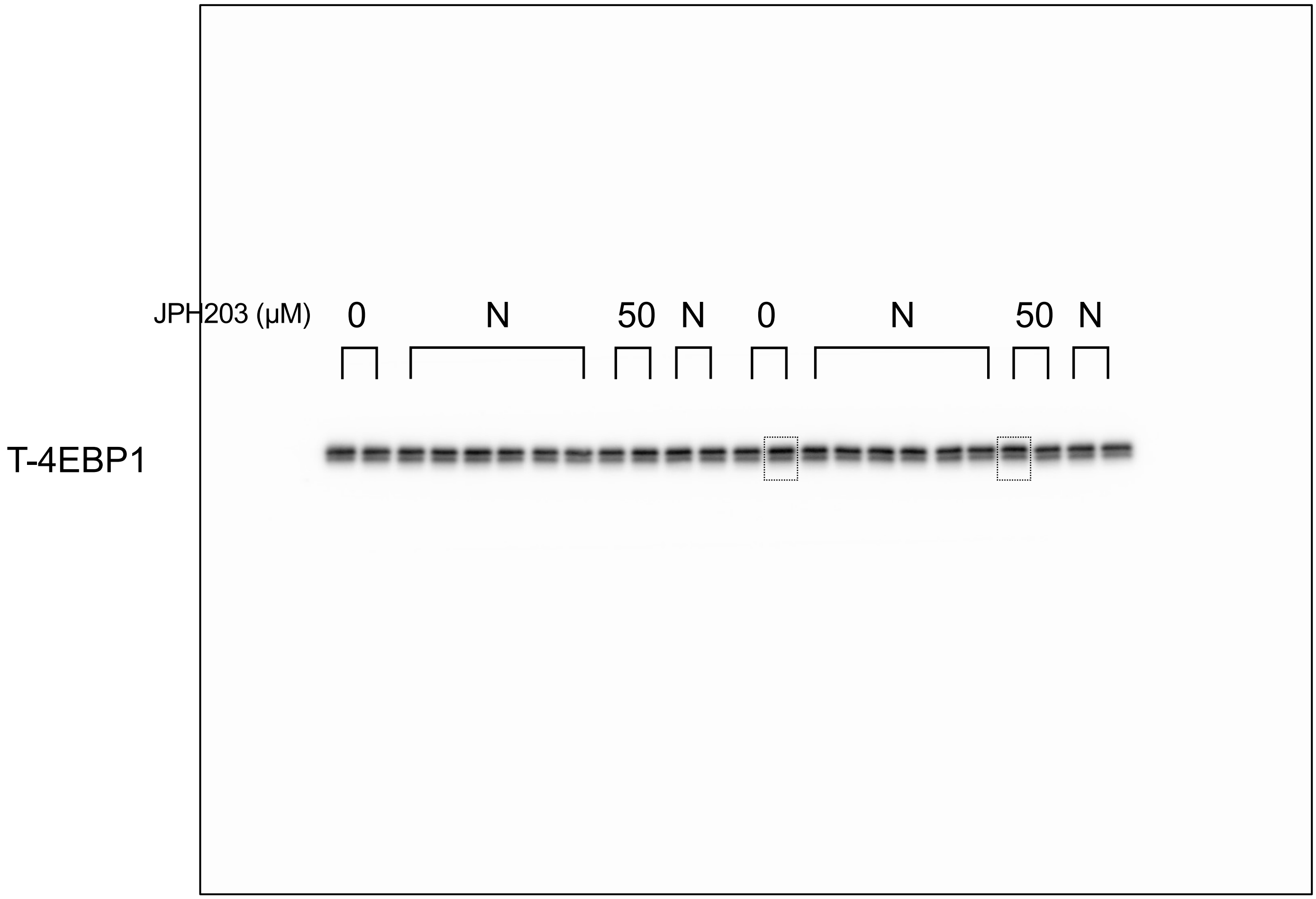

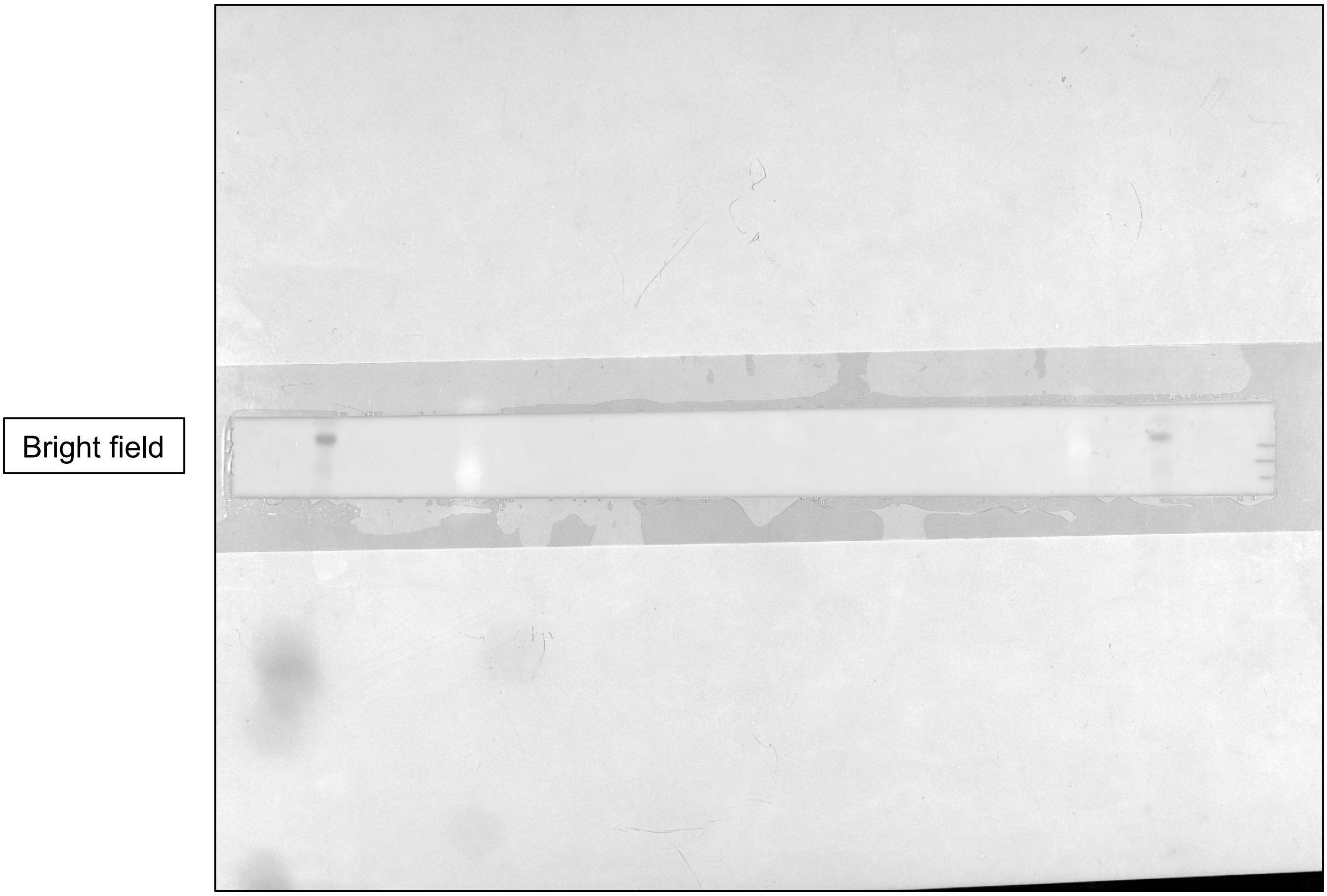

Band images in Fig. 4 (continued).

CBB

For P-p70S6K, P-rpS6, and P-4EBP1.

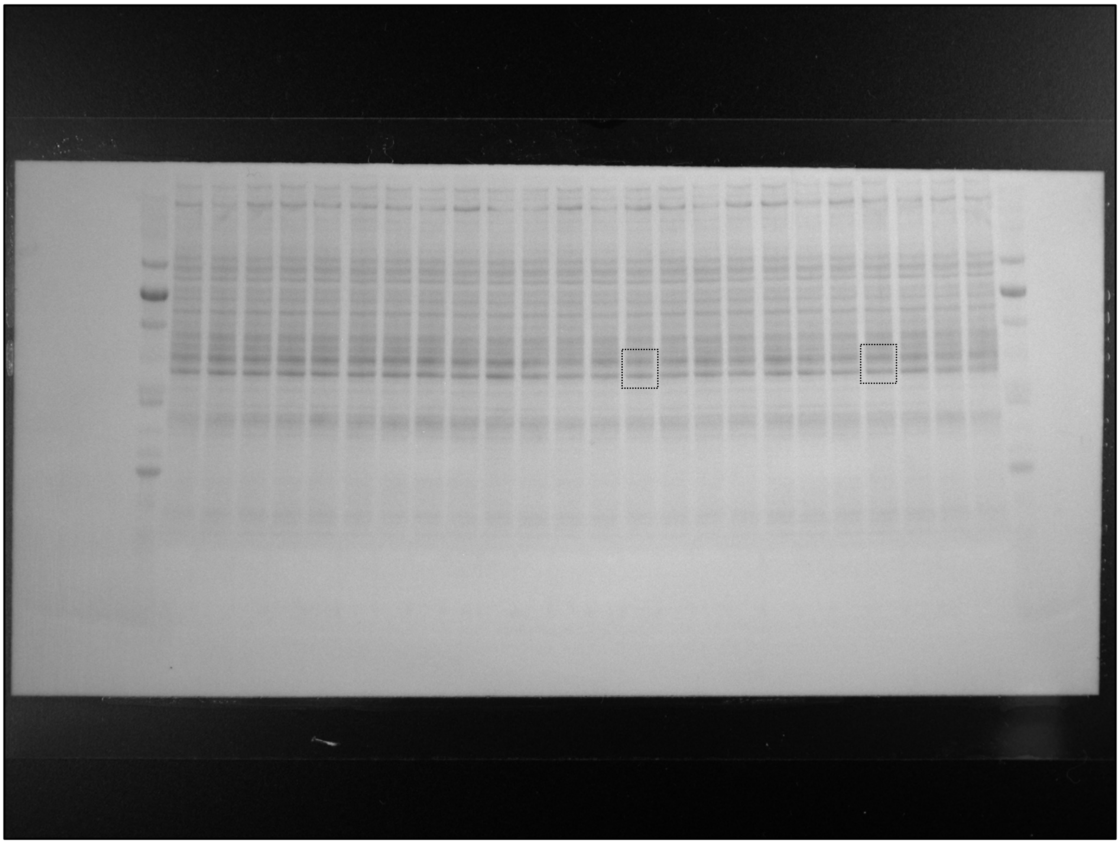

CBB

For T-p70S6K, T-rpS6, and T-4EBP1.

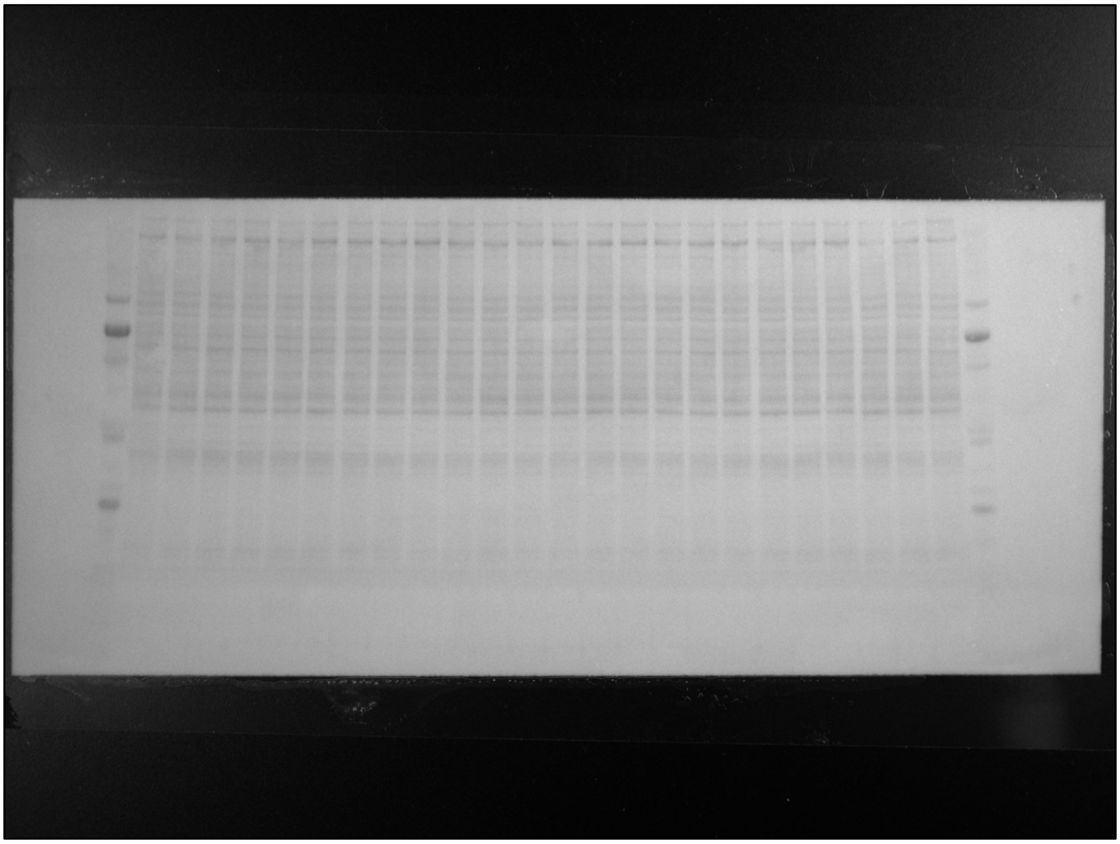

Band images in Fig. 4 (continued).

Atrogin-1

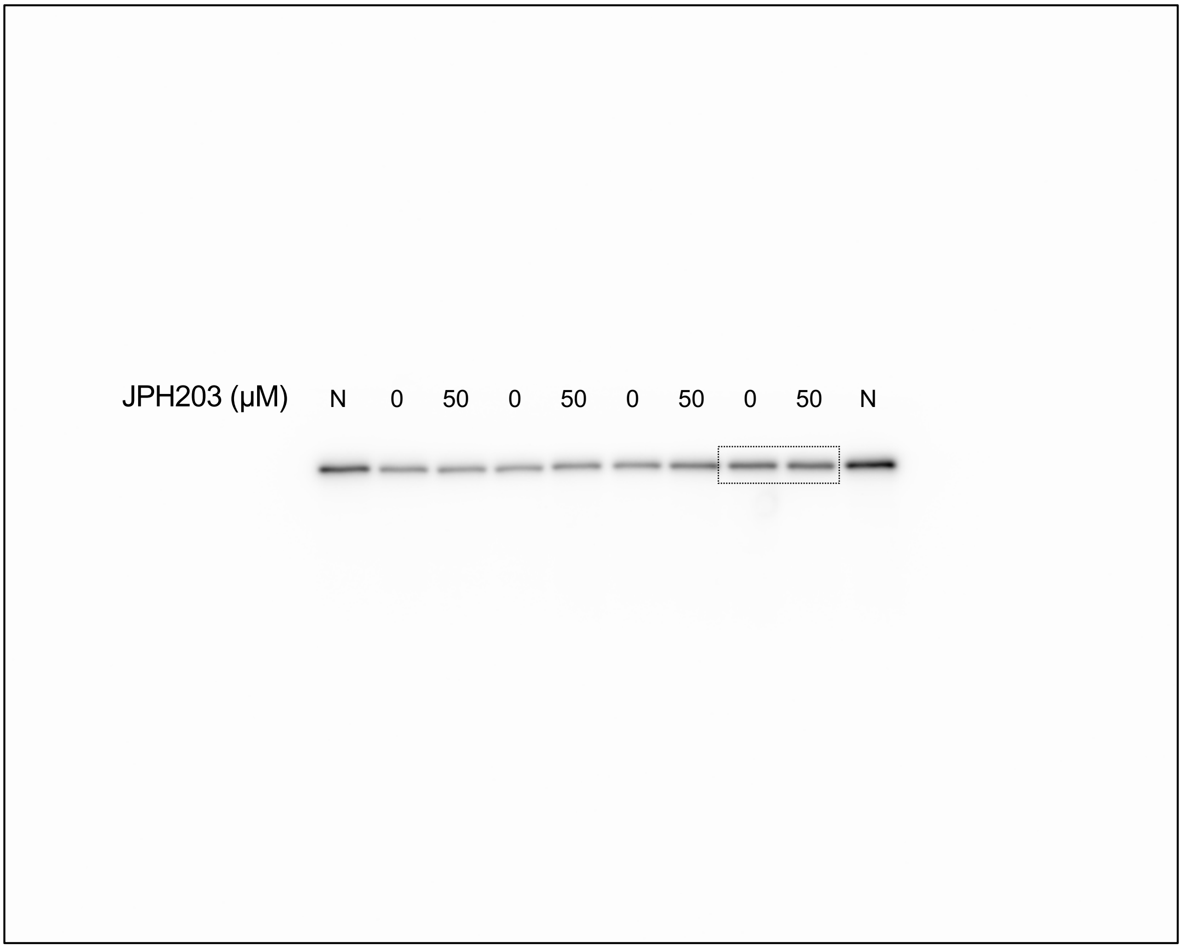

Bright field

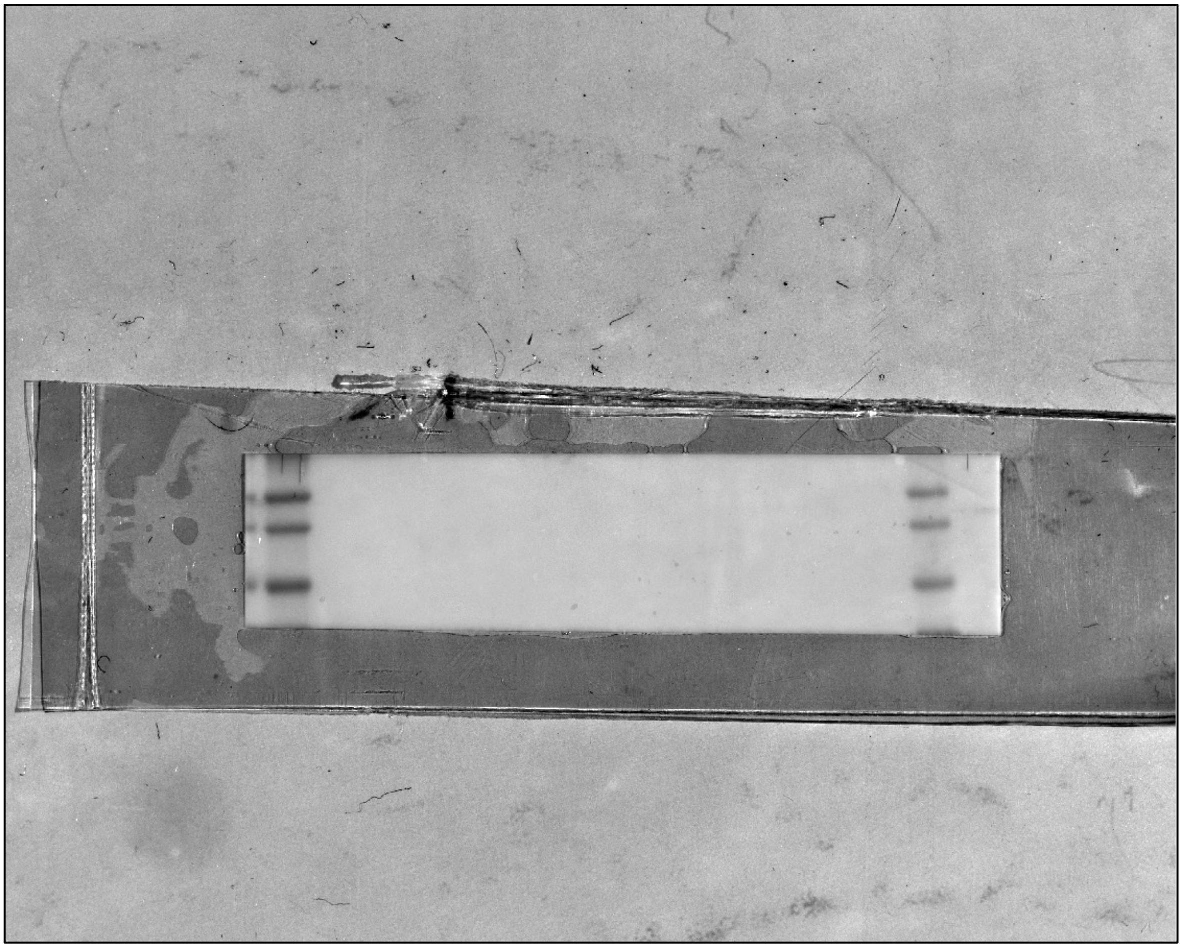

Band images in Fig. 4 (continued).

MuRF-1

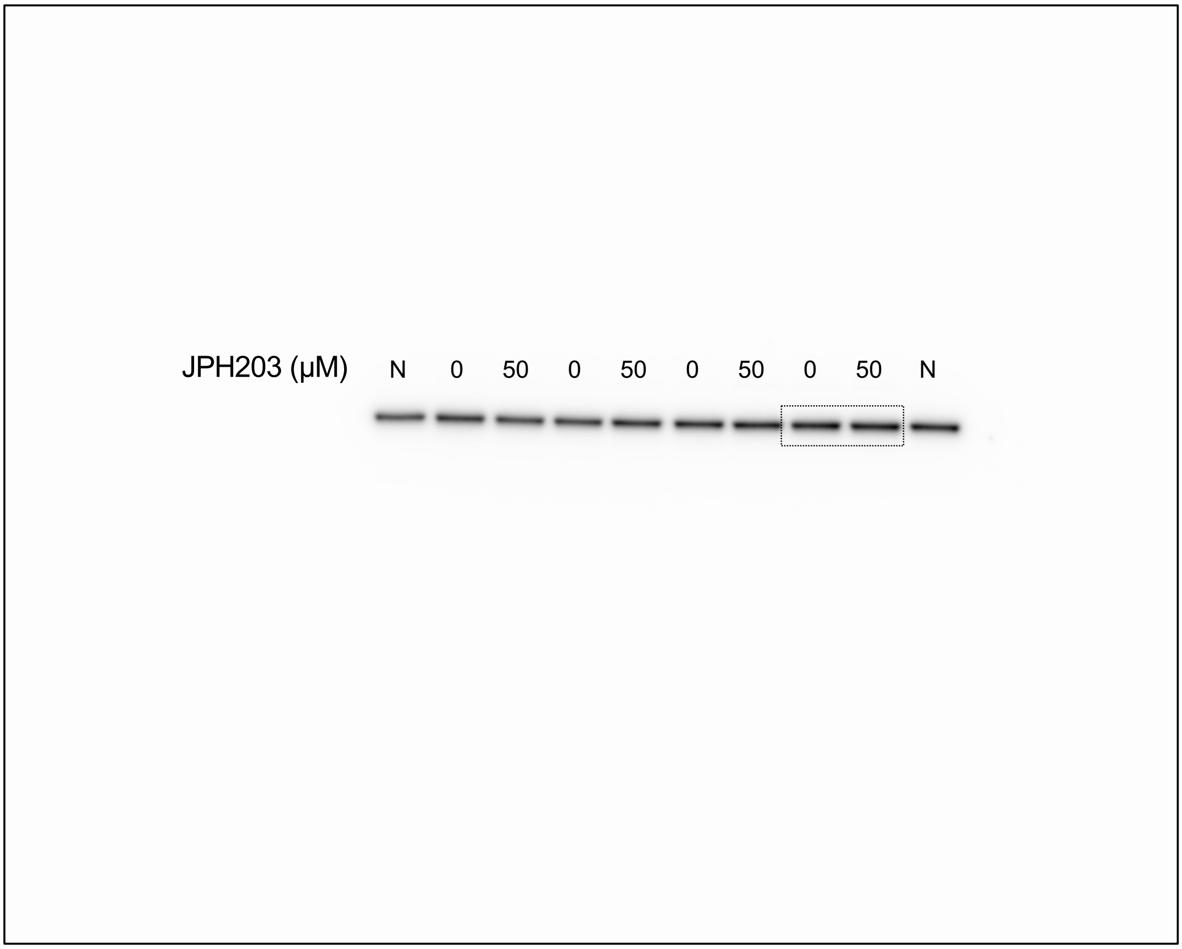

Bright field

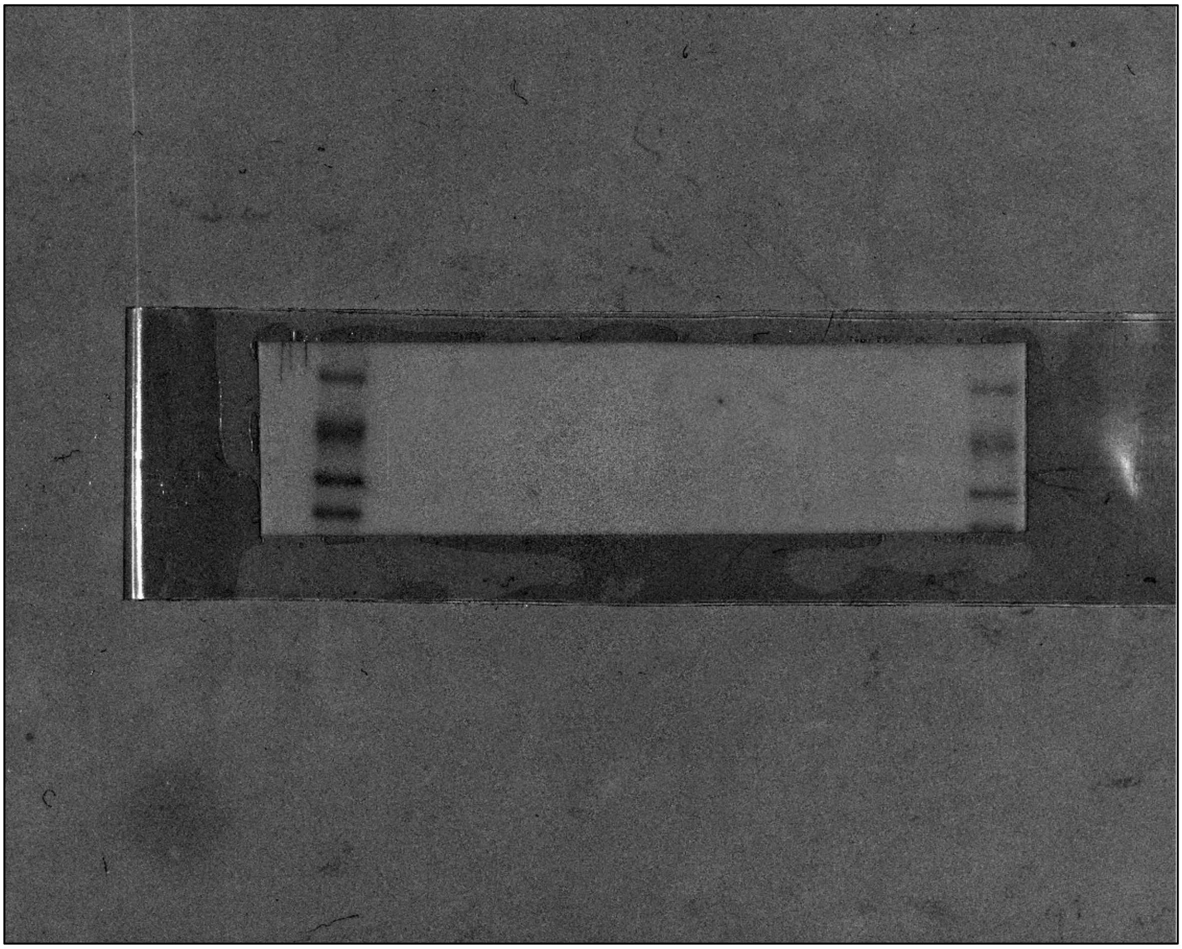

Band images in Fig. 4 (continued).

K48 linkage-specific polyubiquitin

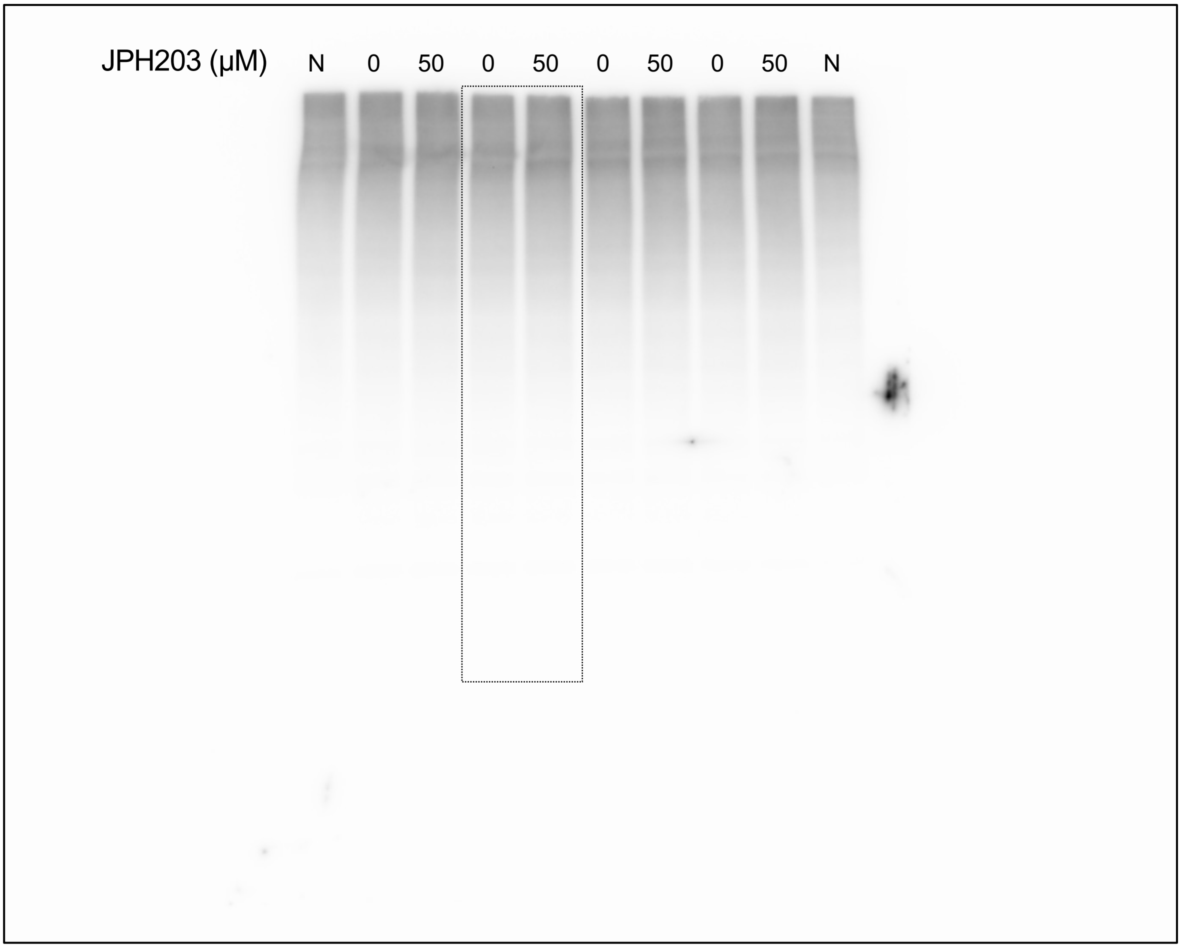

Bright field

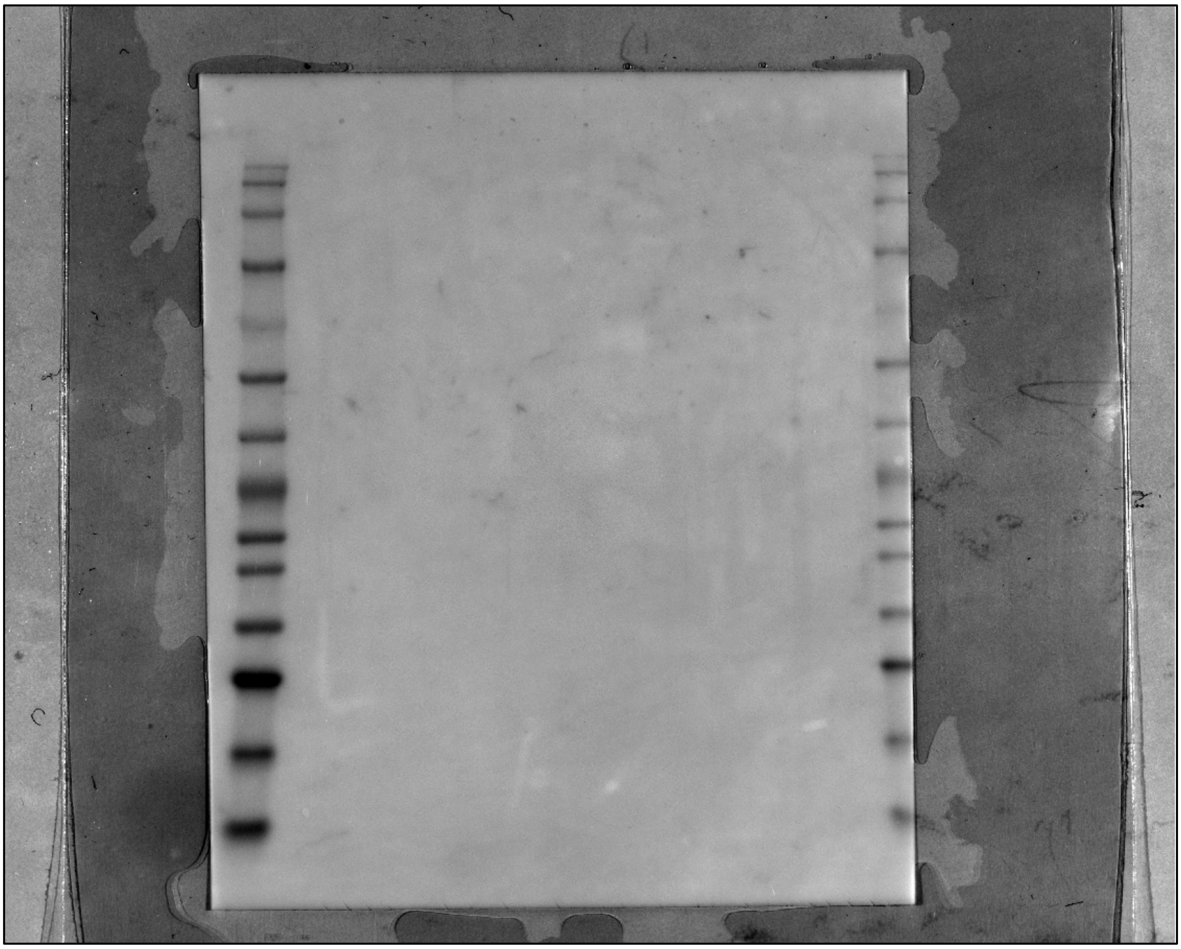

Band images in Fig. 4 (continued).

P-ULK1 (S757)

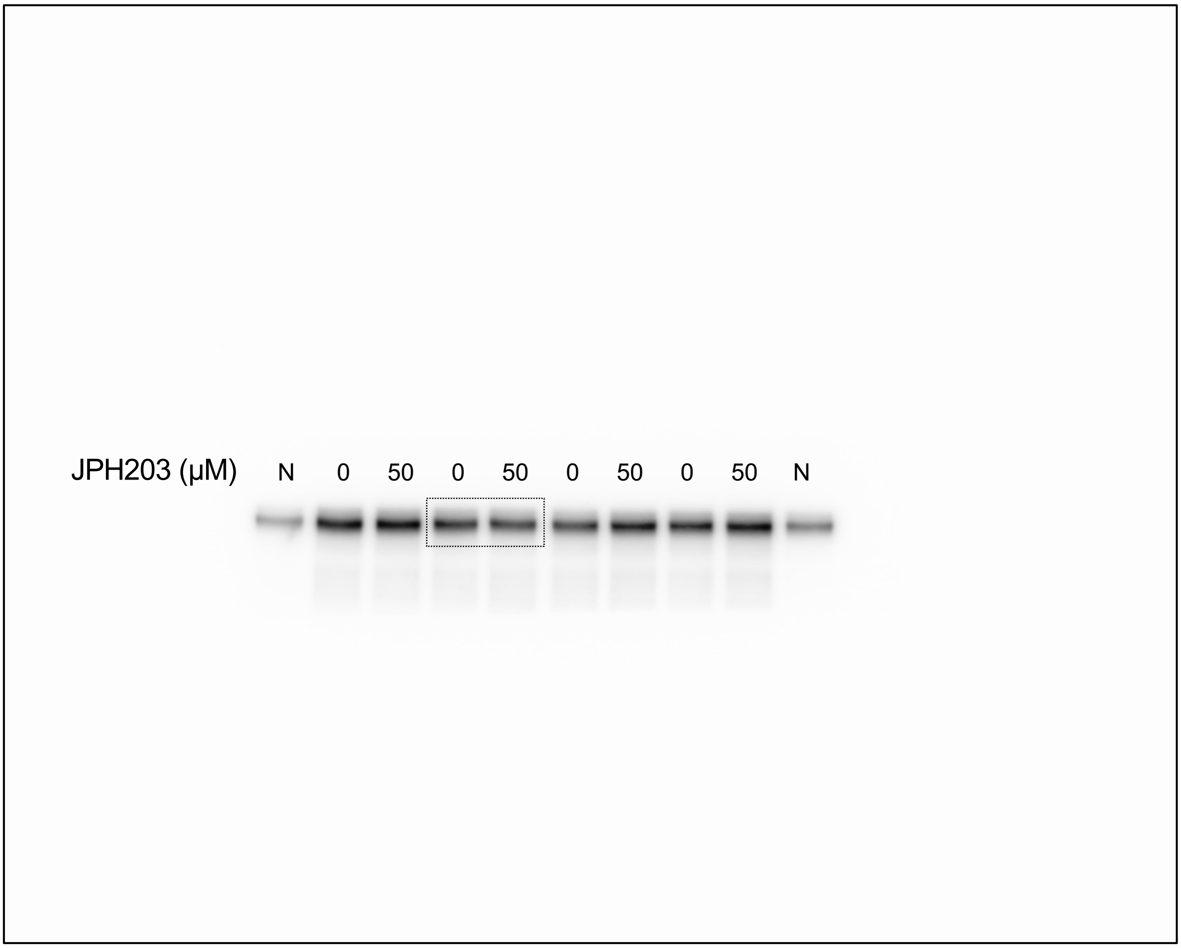

Bright field

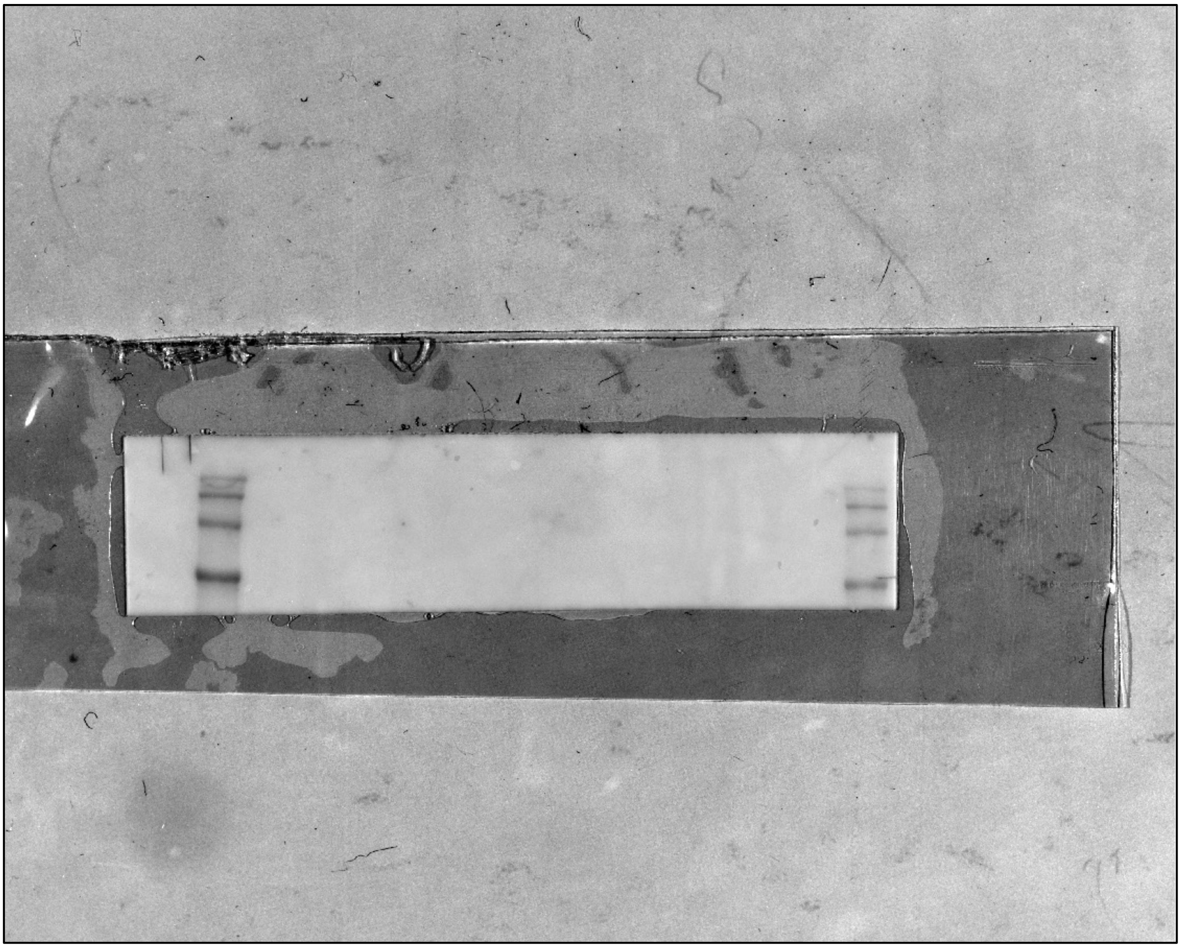

Band images in Fig. 4 (continued).

P-ULK1
(S555)

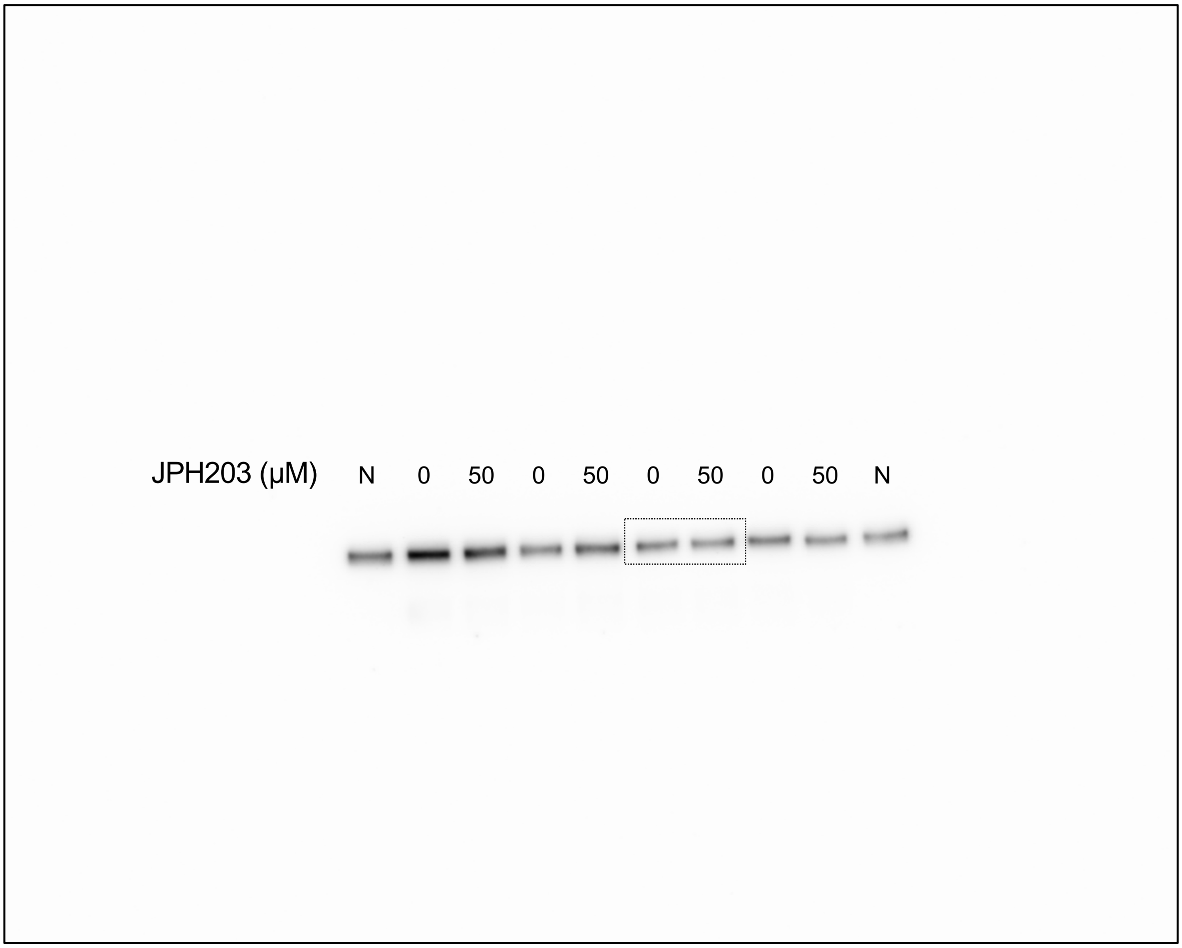

Bright field

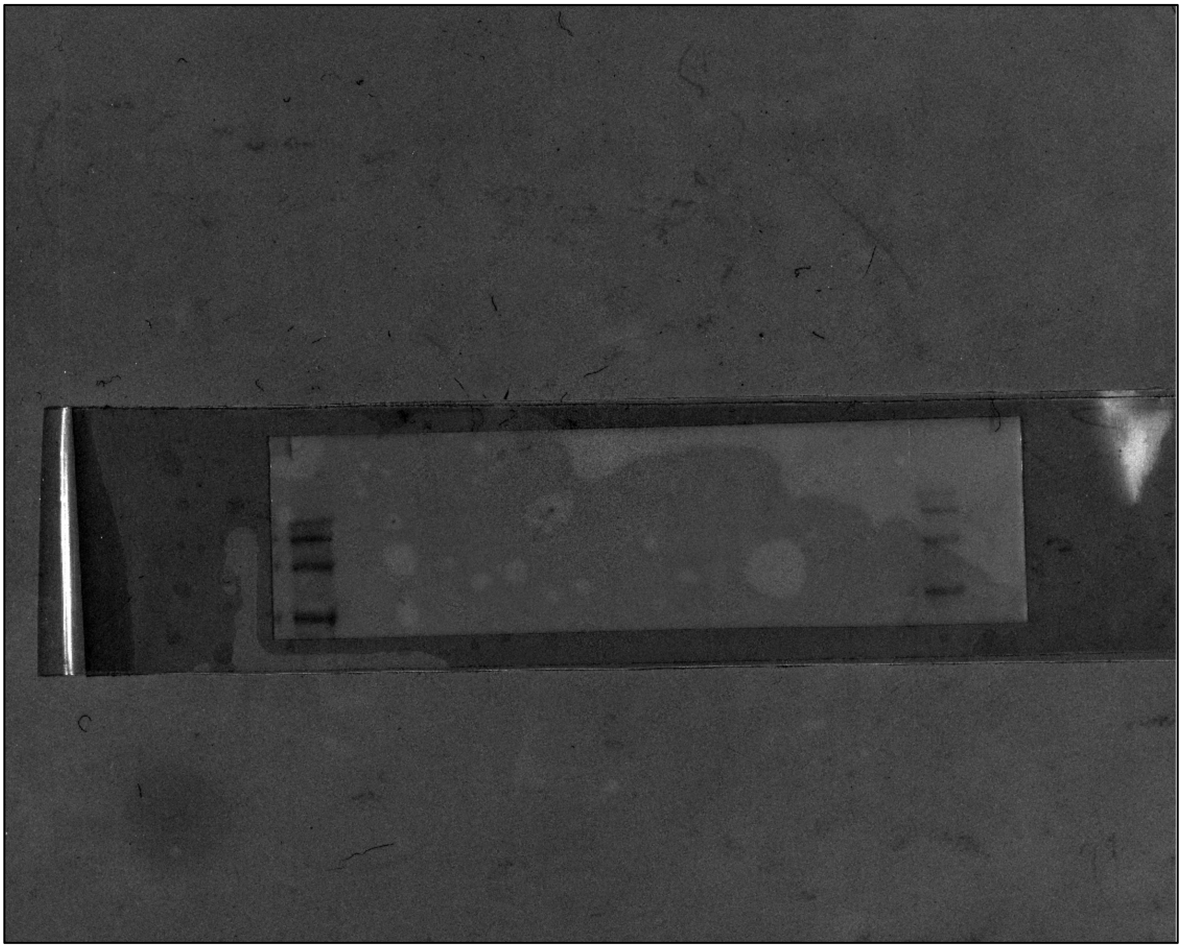

Band images in Fig. 4 (continued).

T-ULK1

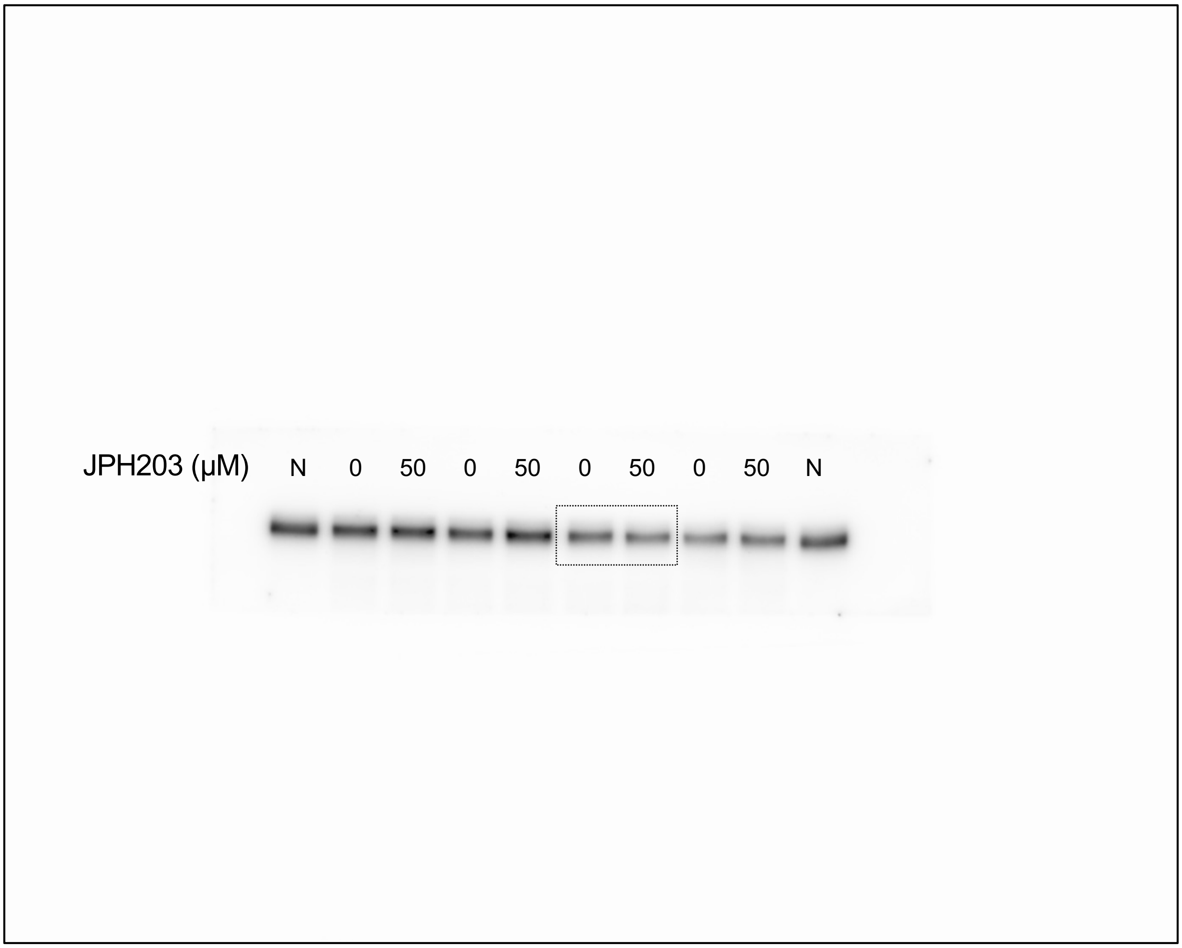

Bright field

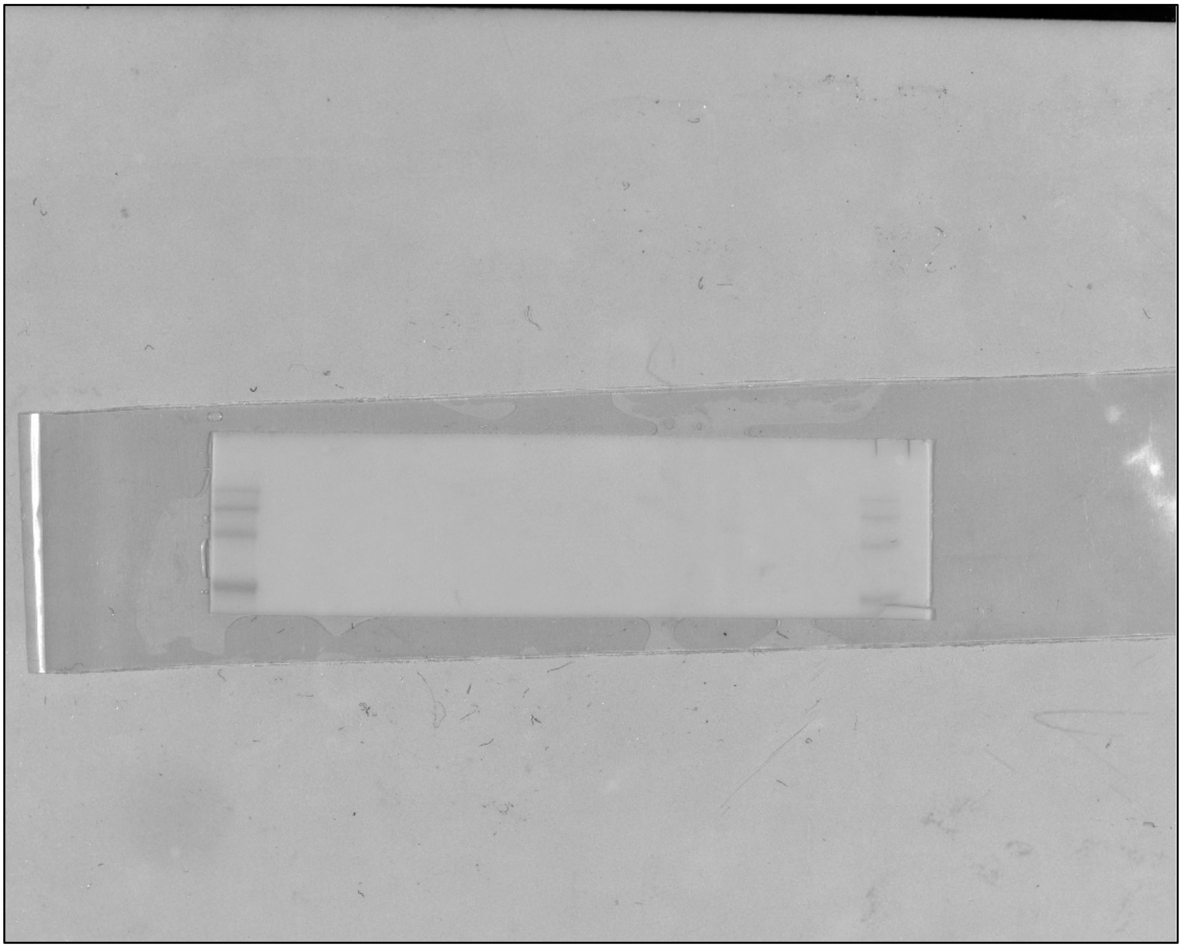

Band images in Fig. 4 (continued).

p62

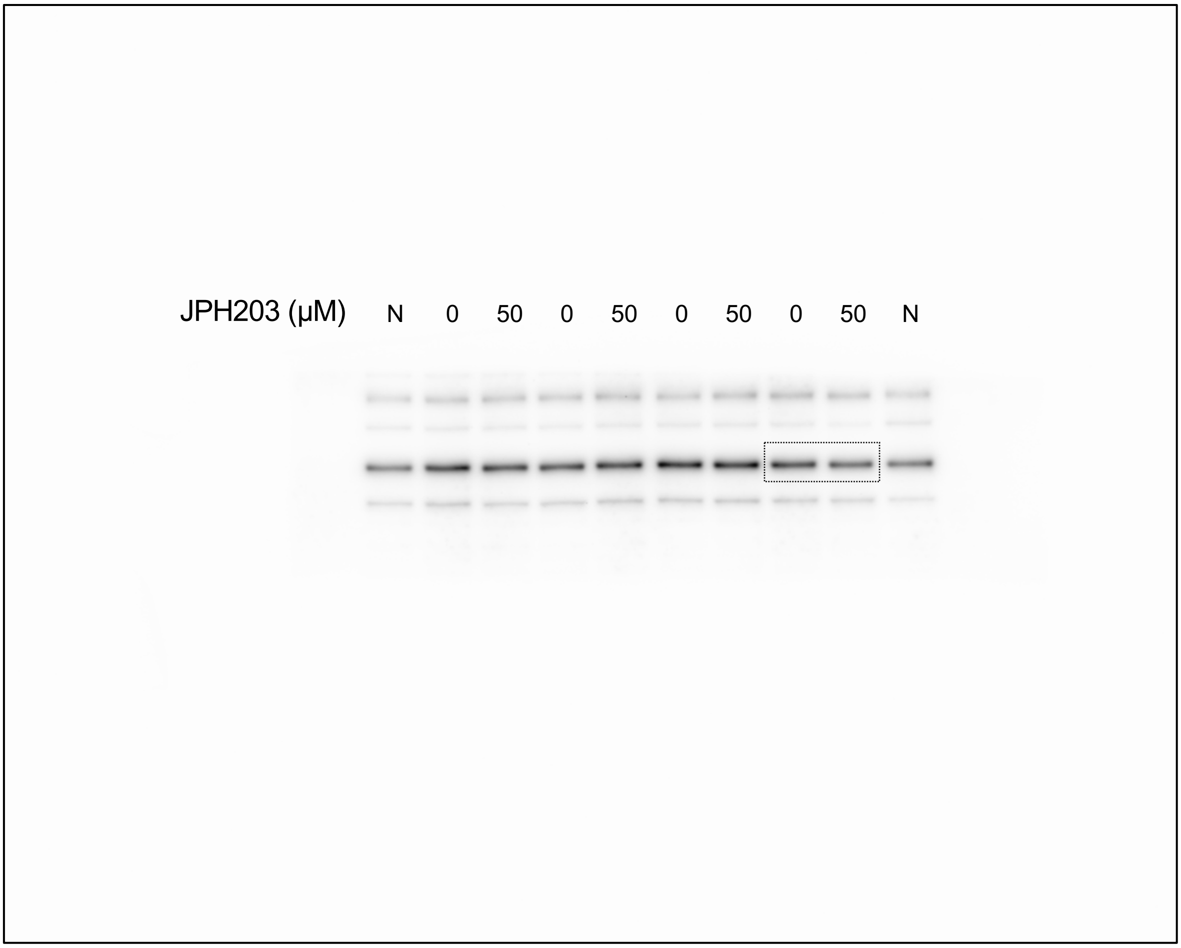

Bright field

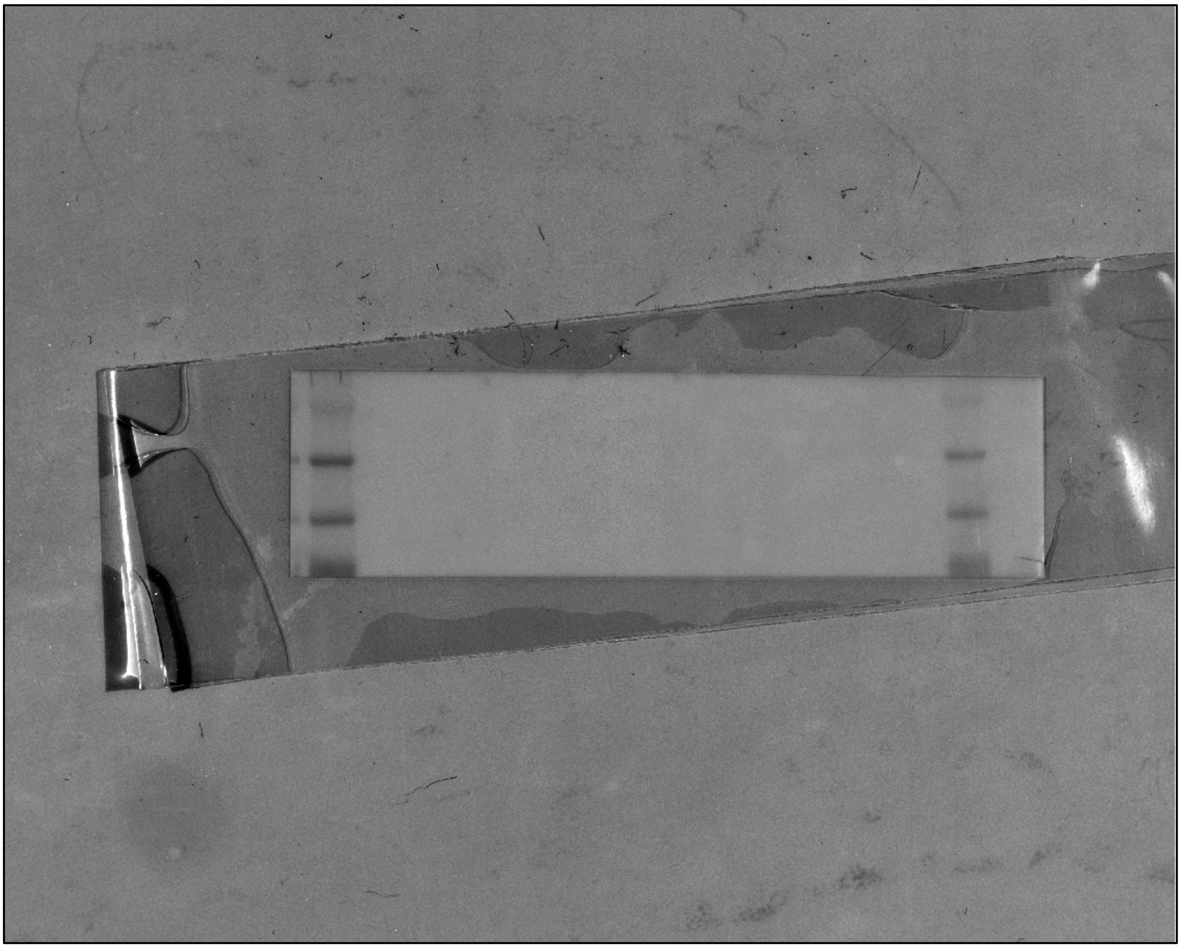

Band images in Fig. 4 (continued).

LC3

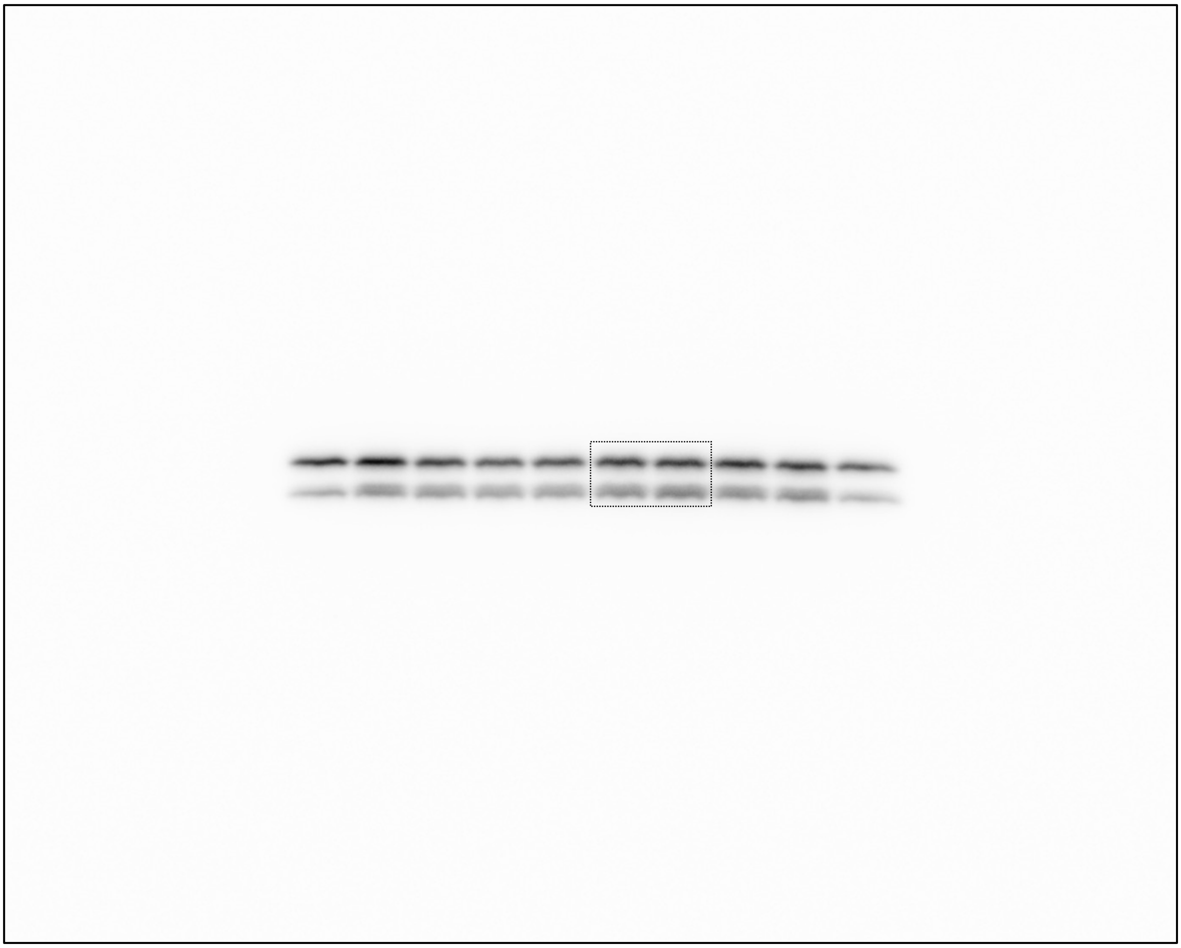

Bright field

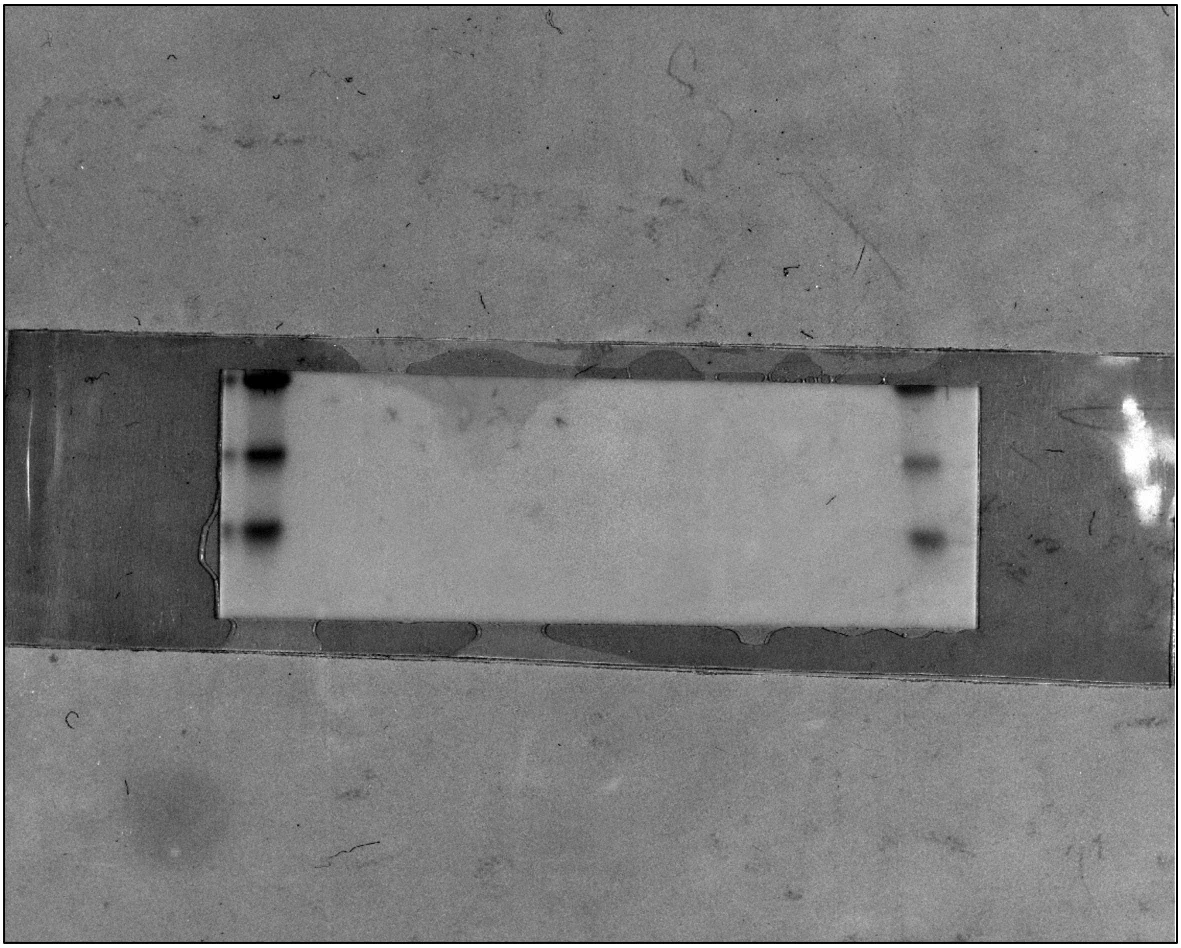

Band images in Fig. 4 (continued).

CBB

For Atrogin-1, K48 linkage-specific polyubiquitin, T-ULK1, p62, and LC3.

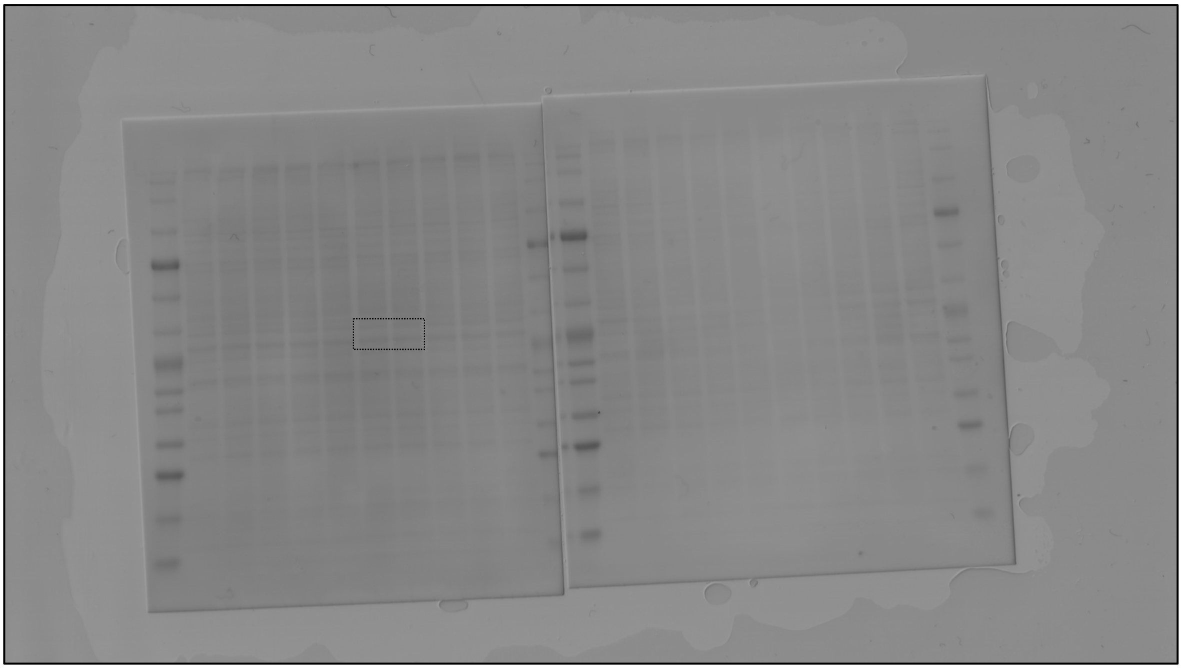

CBB

For MuRF-1, P-ULK1 (S757), and P-ULK1 (S555).

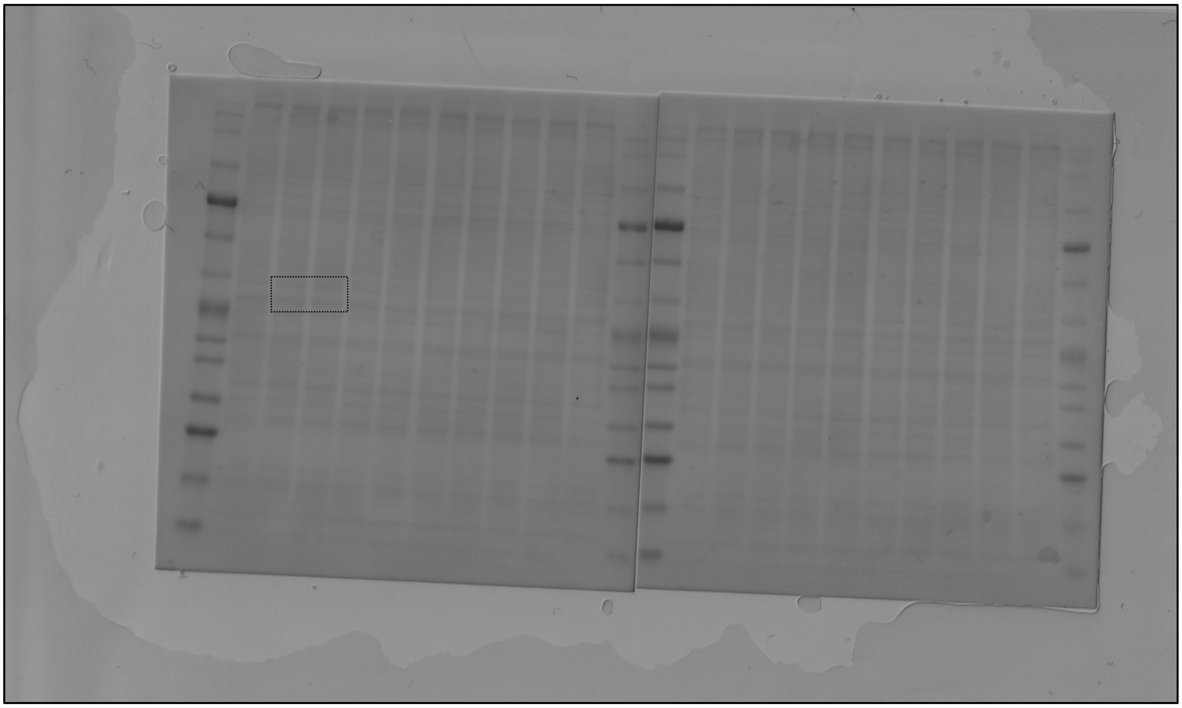

Band images in Fig. 5.

Puromycin

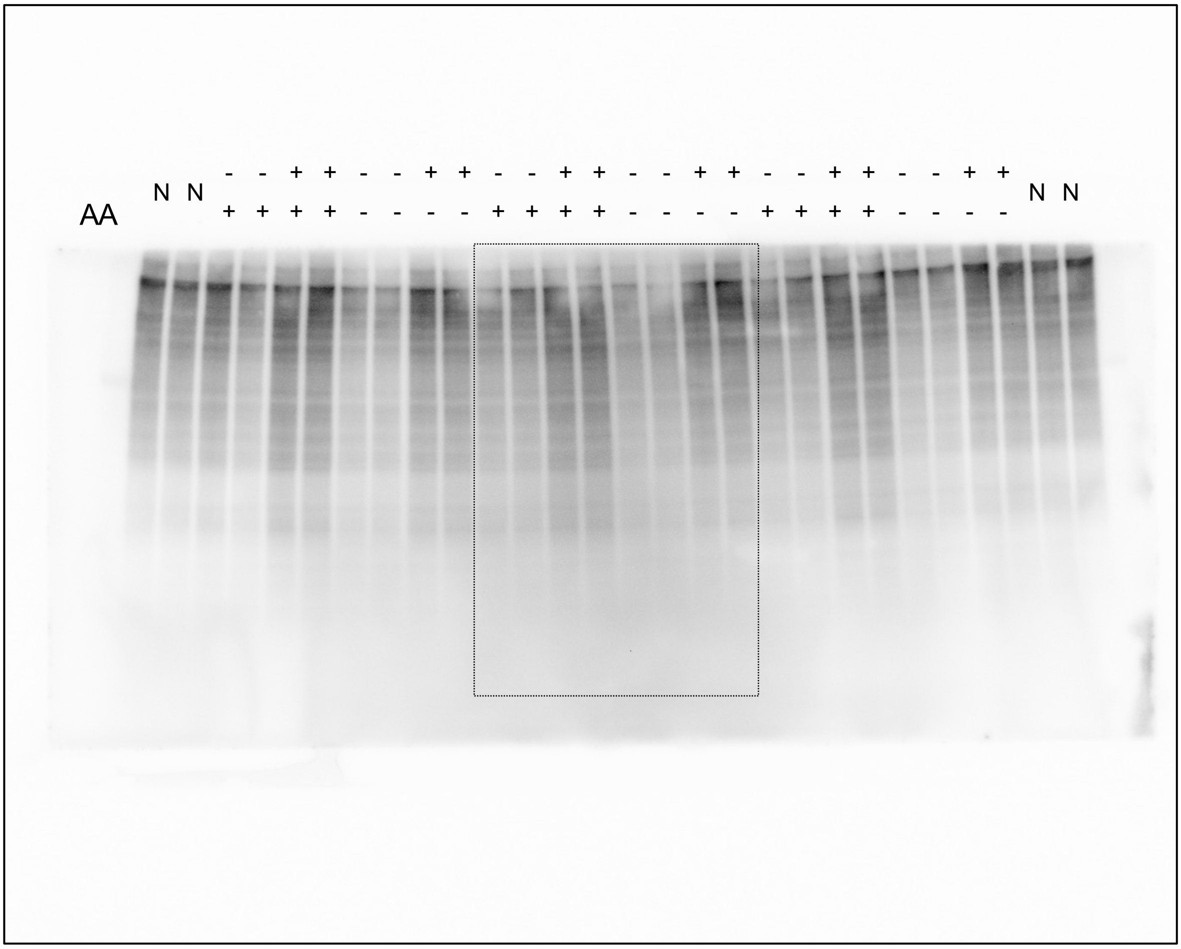

Bright field

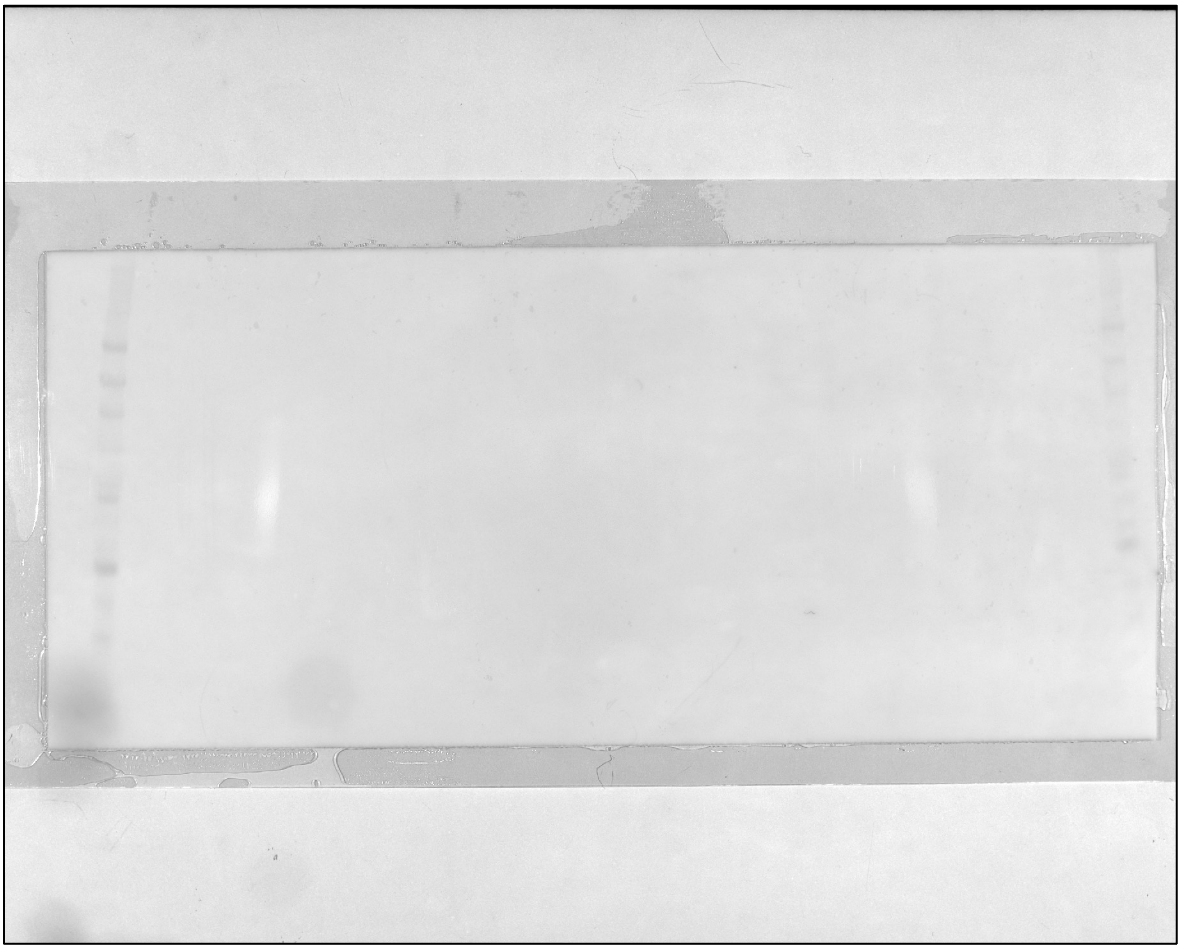

Band images in Fig. 5 (continued).

CBB

For puromycin

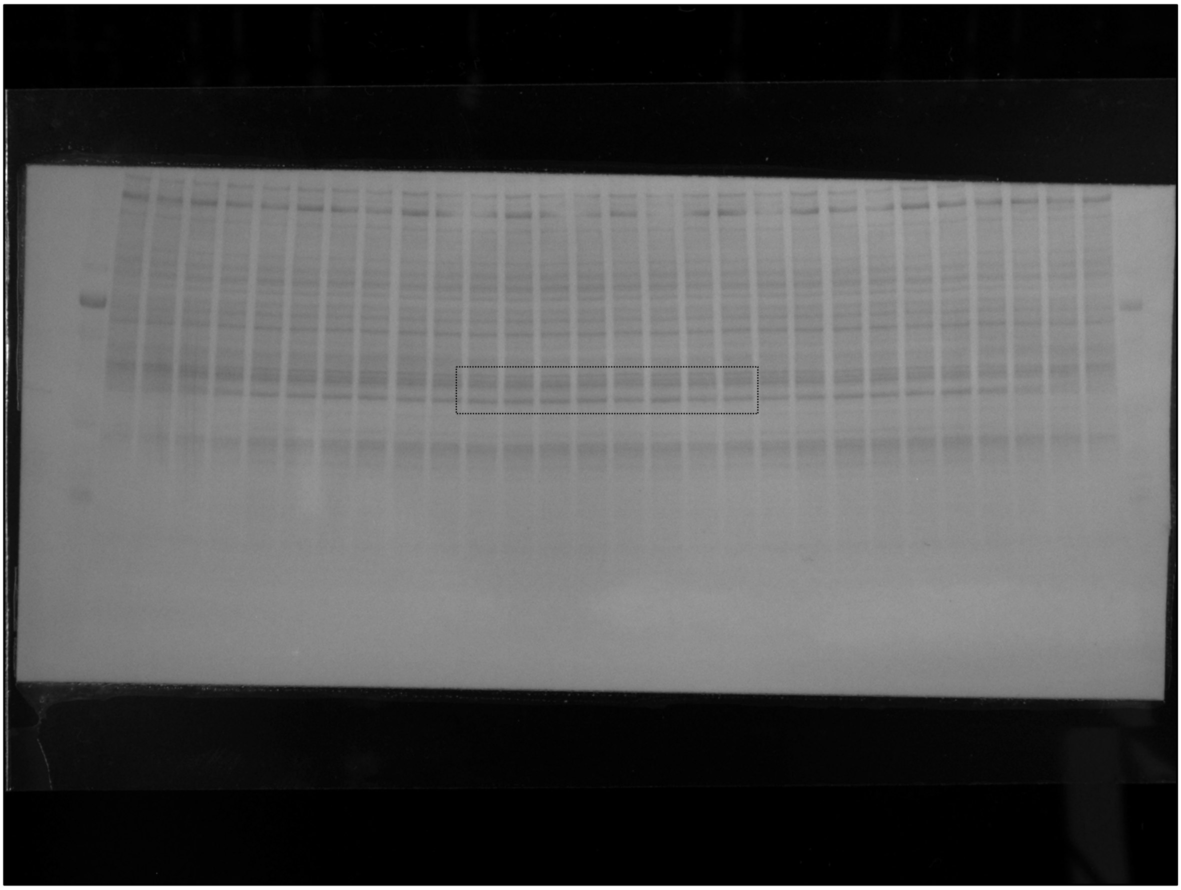

Band images in Fig. 5 (continued).

P-p70S6K

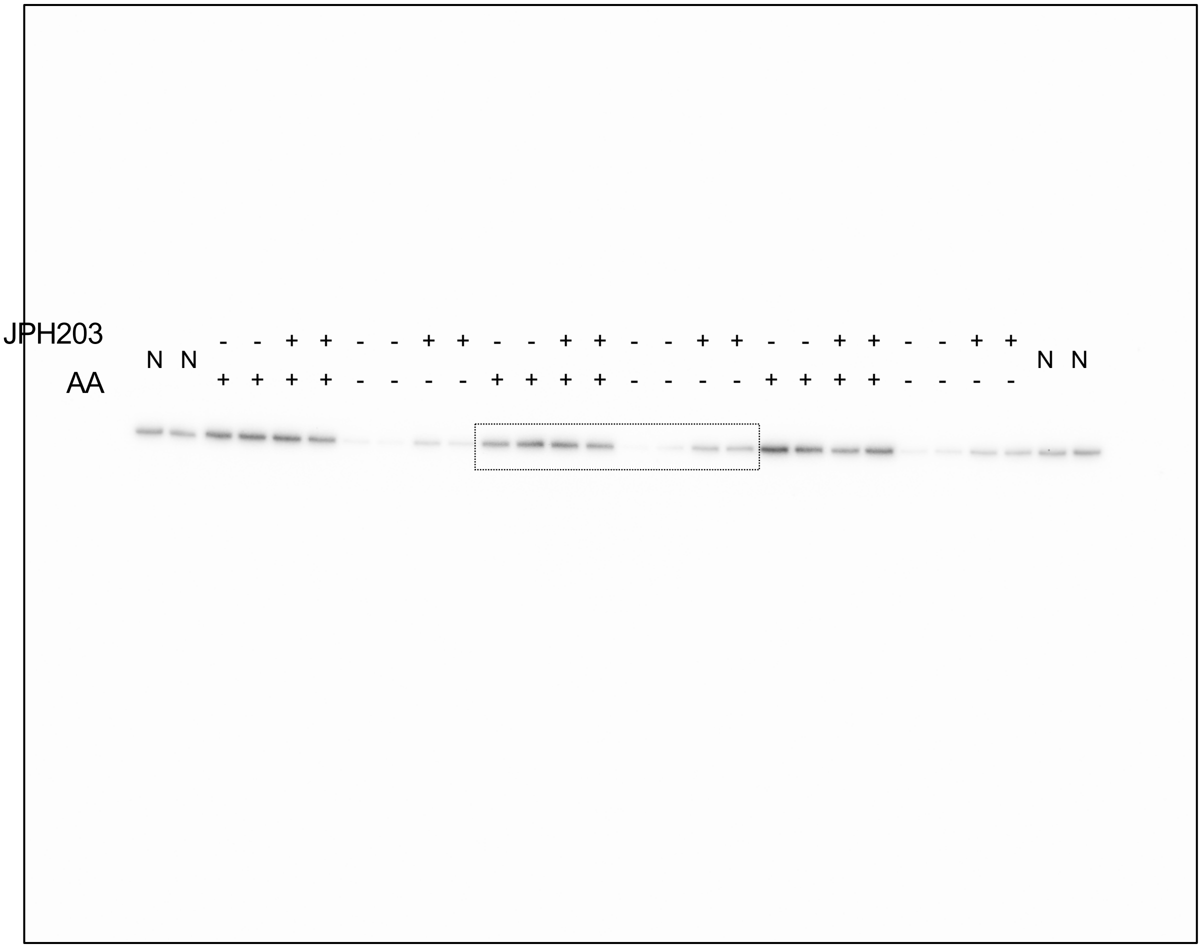

Bright field

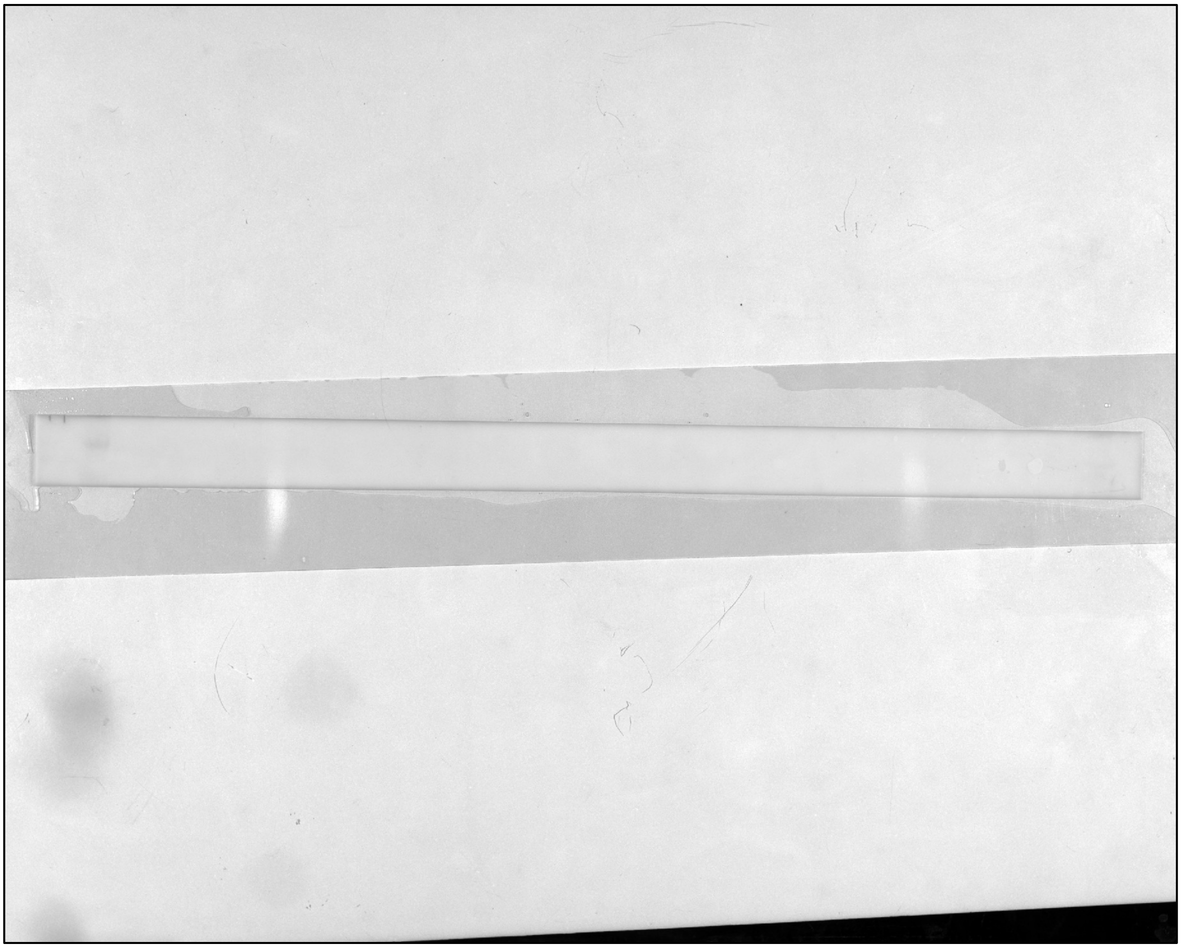

Band images in Fig. 5 (continued).

P-rpS6

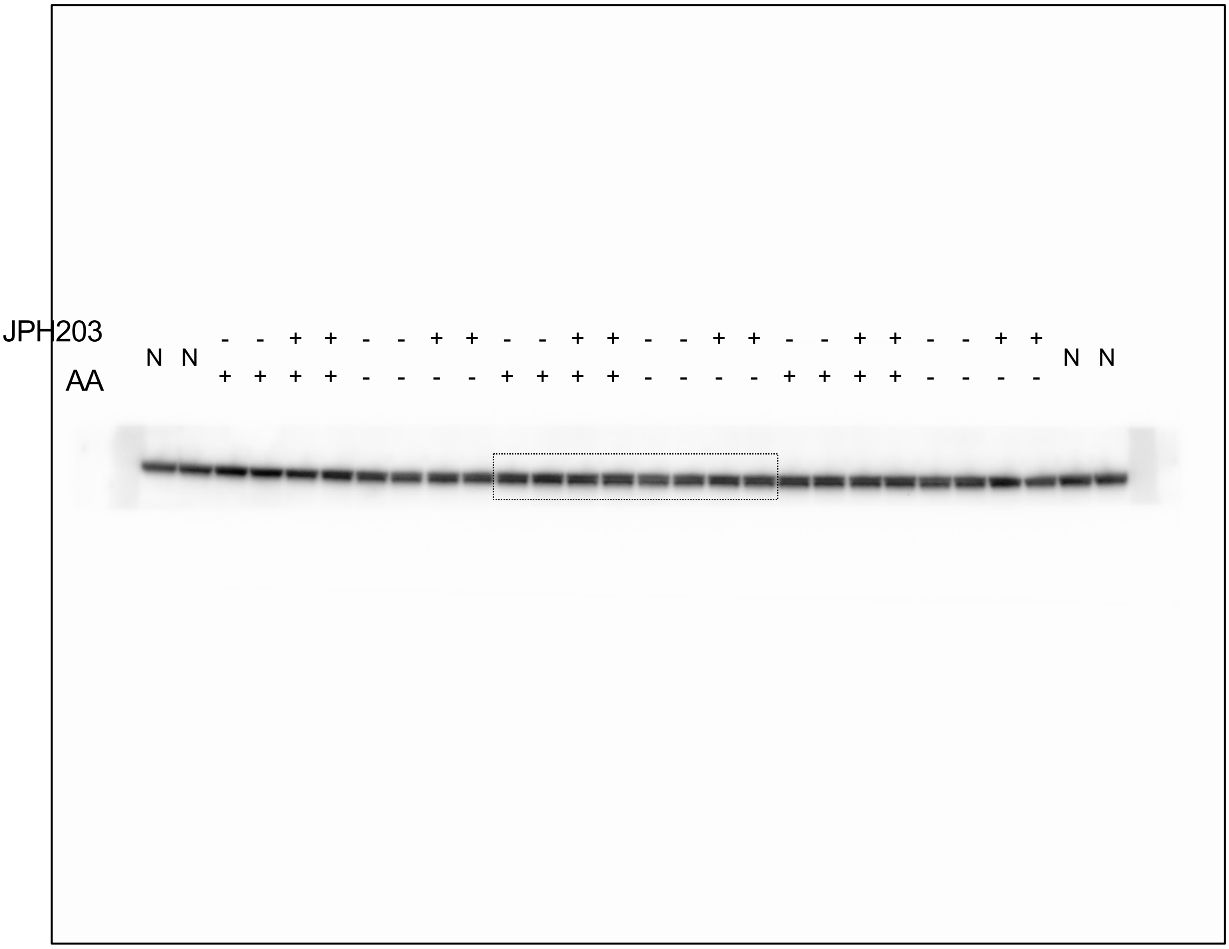

Bright field

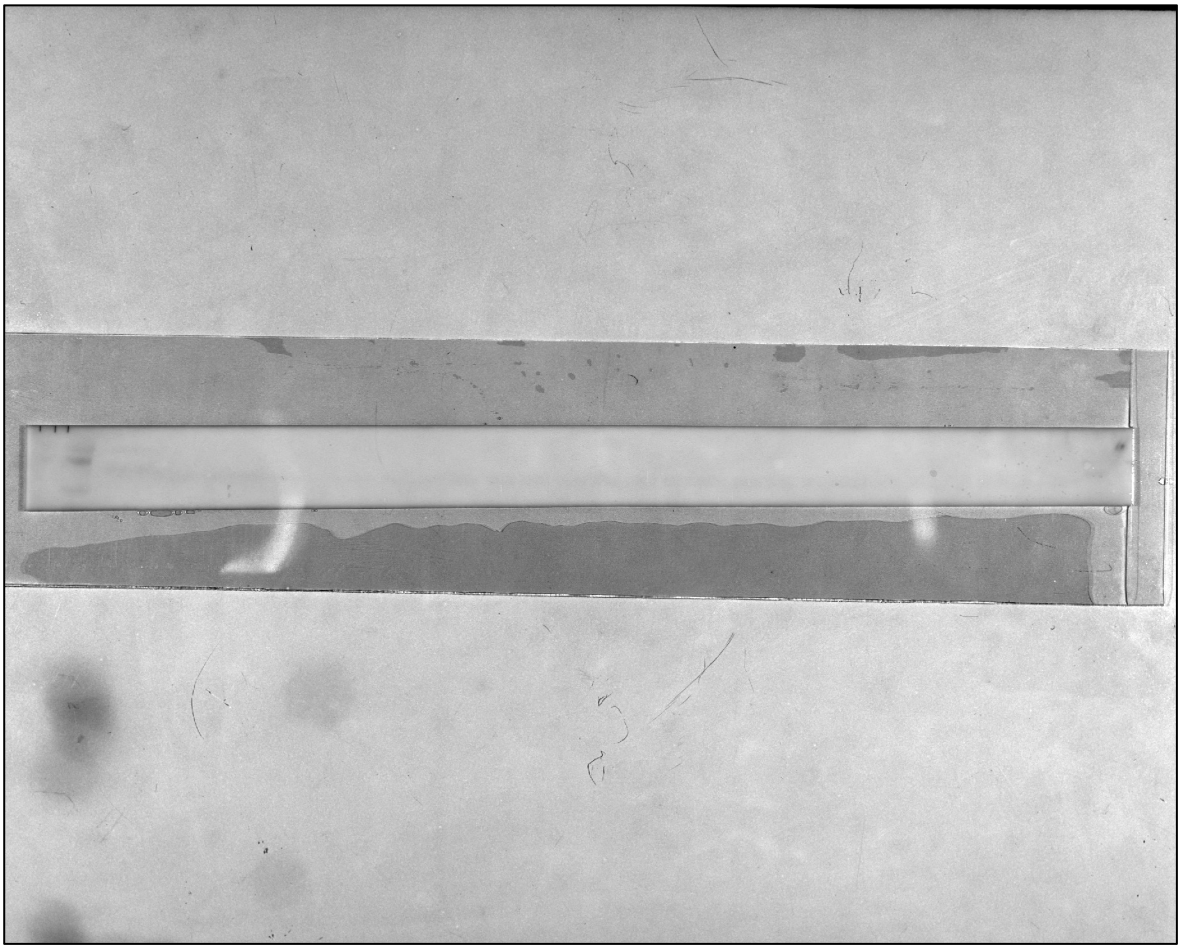

Band images in Fig. 5 (continued).

P-4EBP1

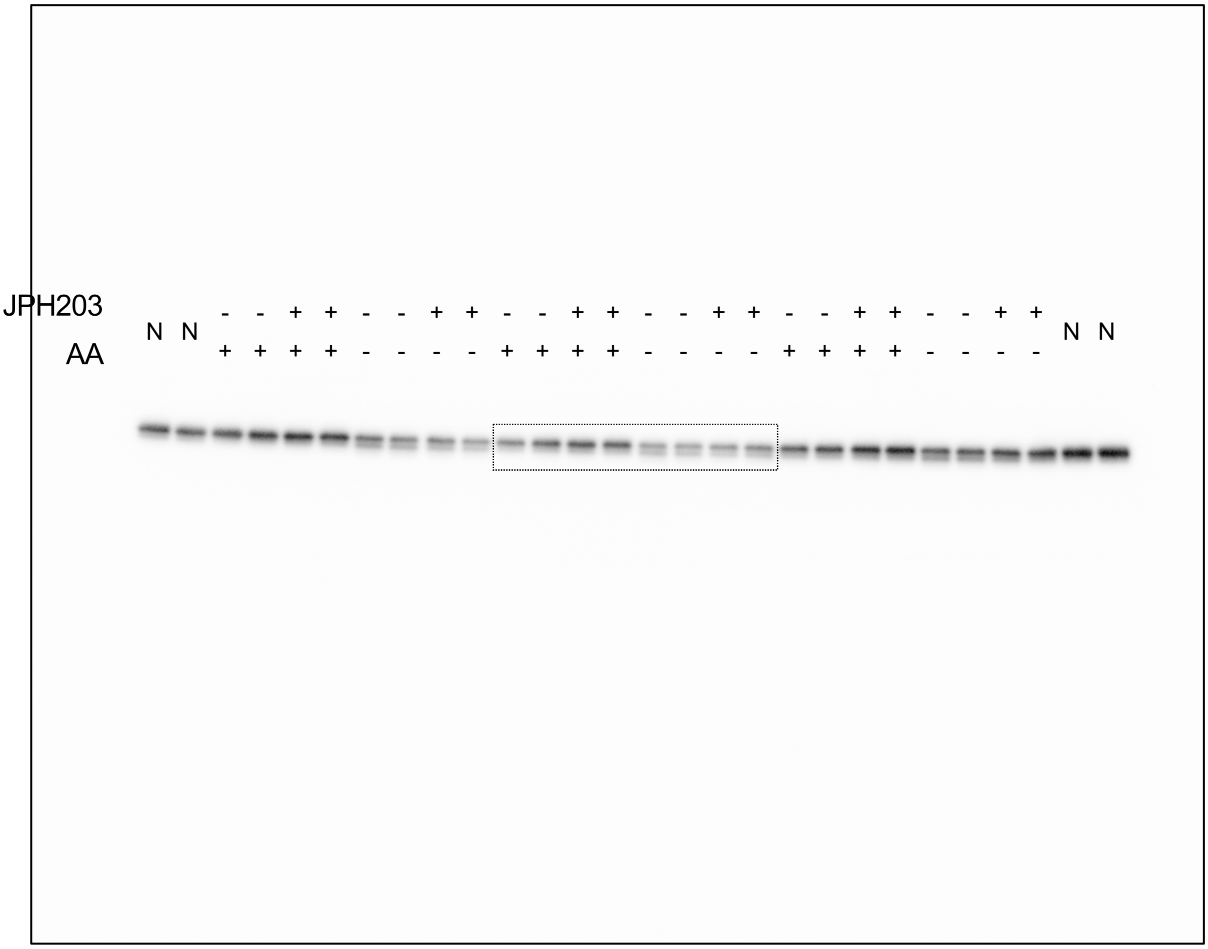

Bright field

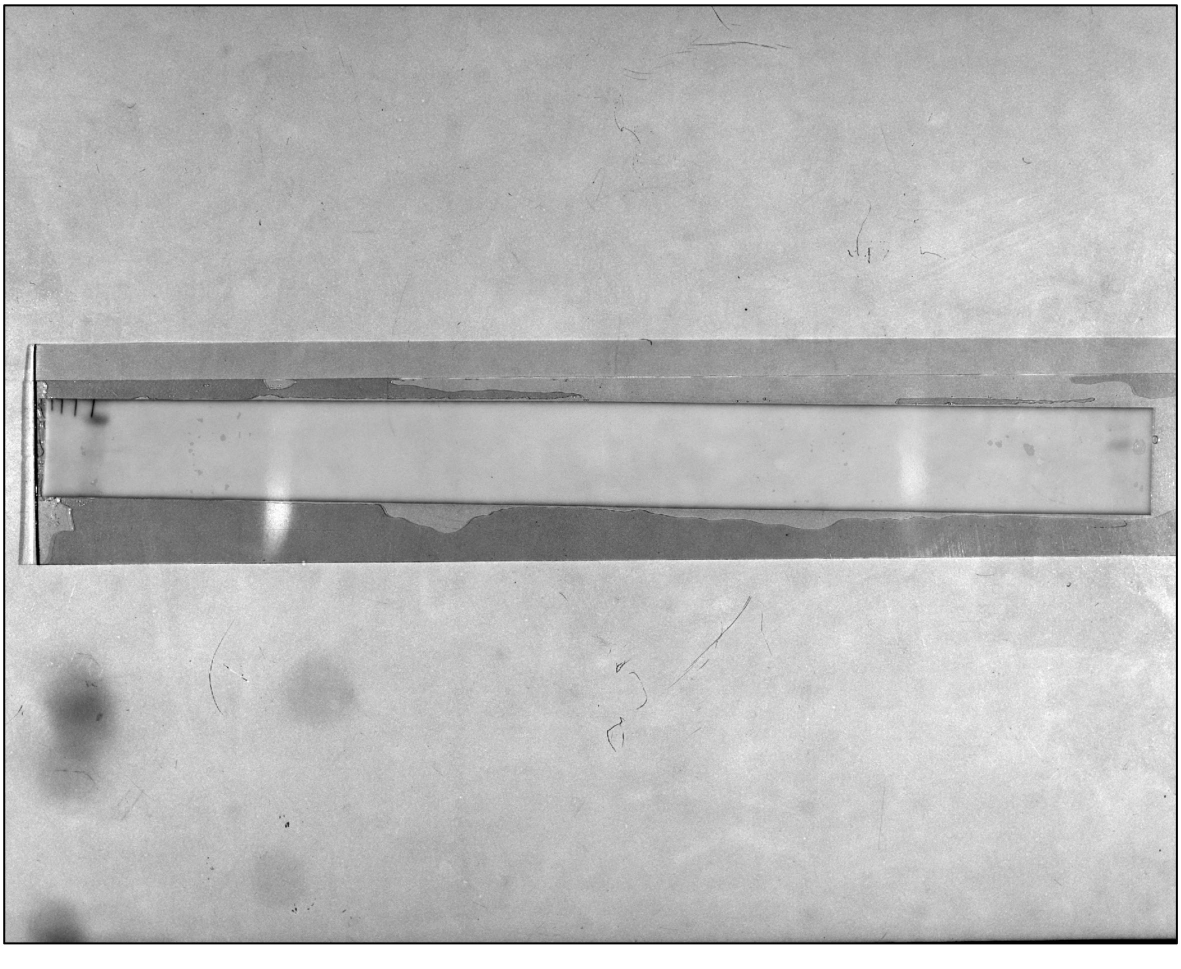

Band images in Fig. 5 (continued).

T-p70S6K

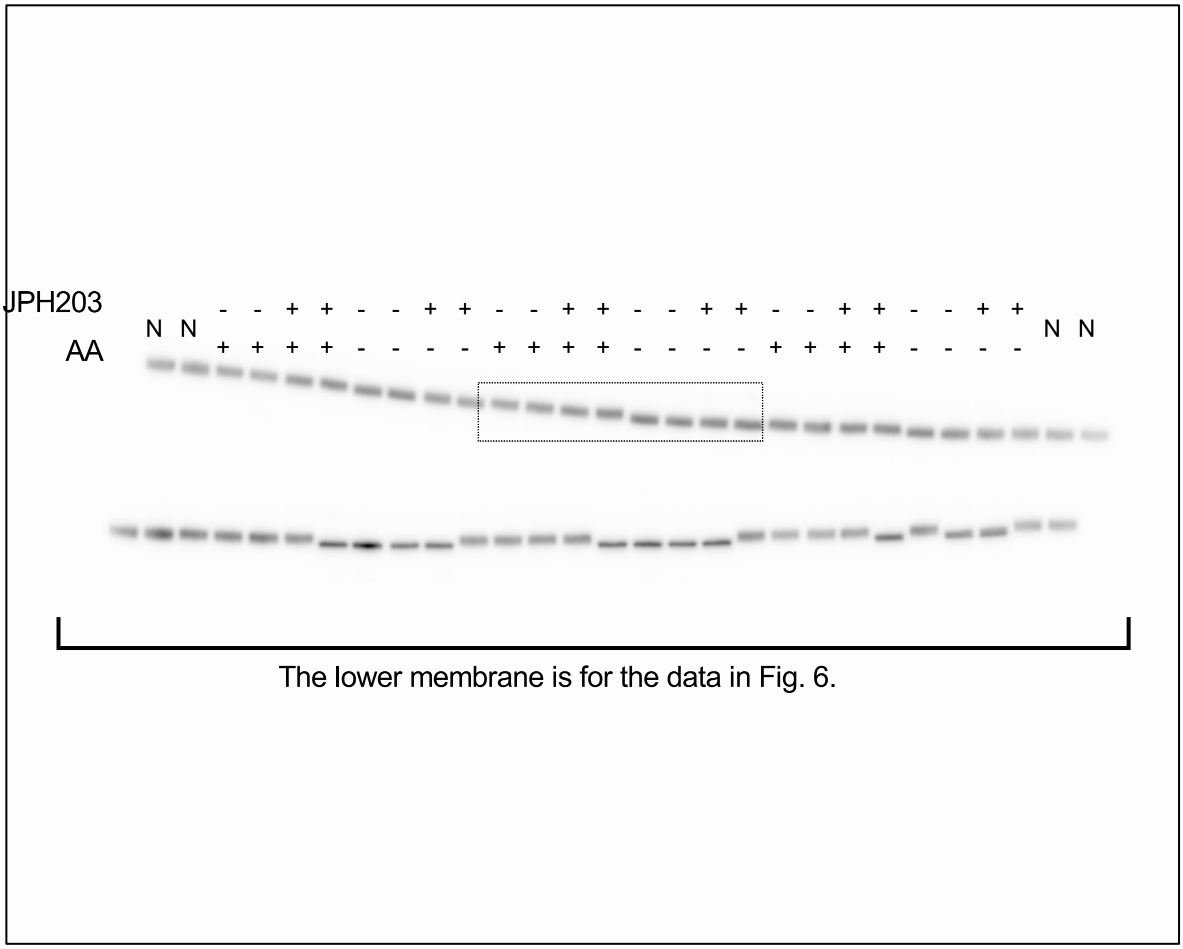

Bright field

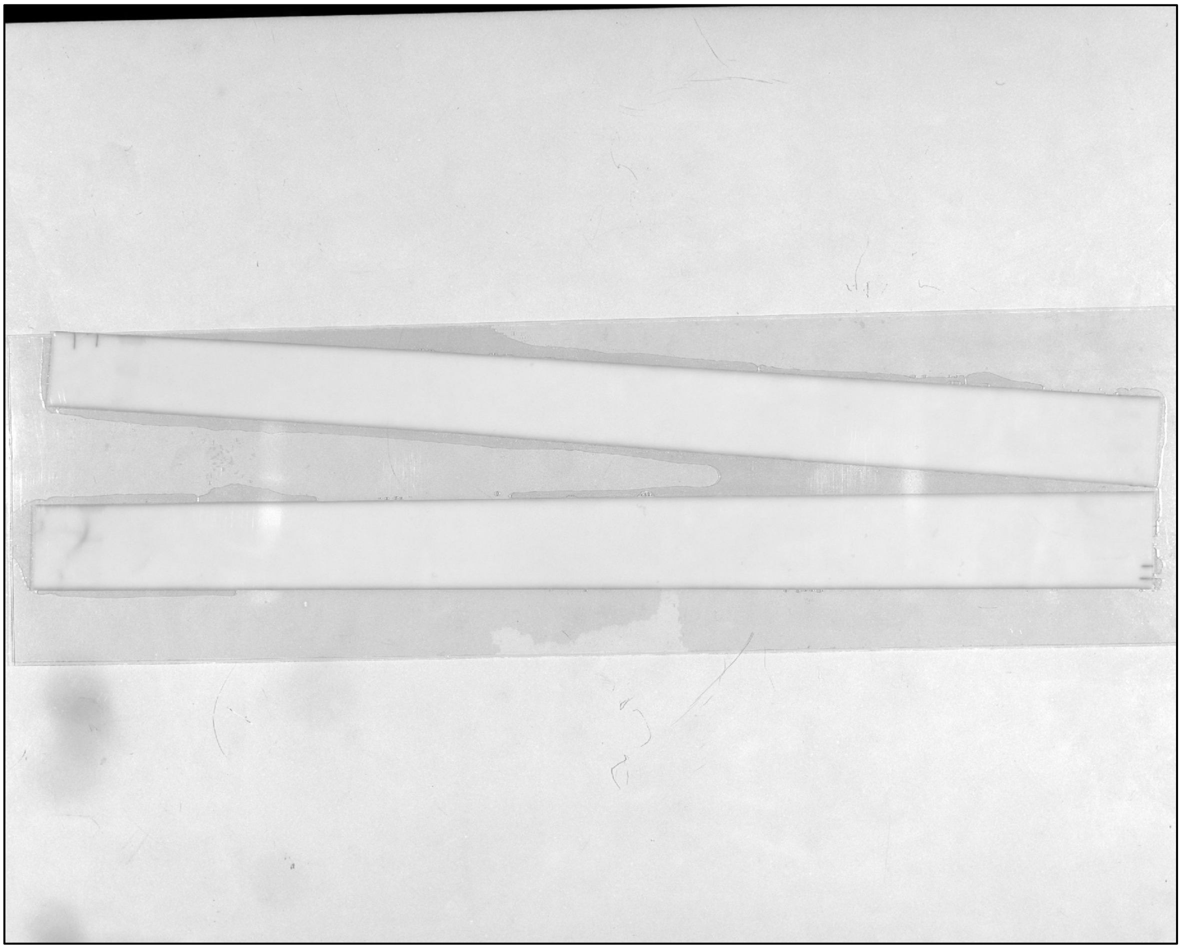

Band images in Fig. 5 (continued).

T-rpS6

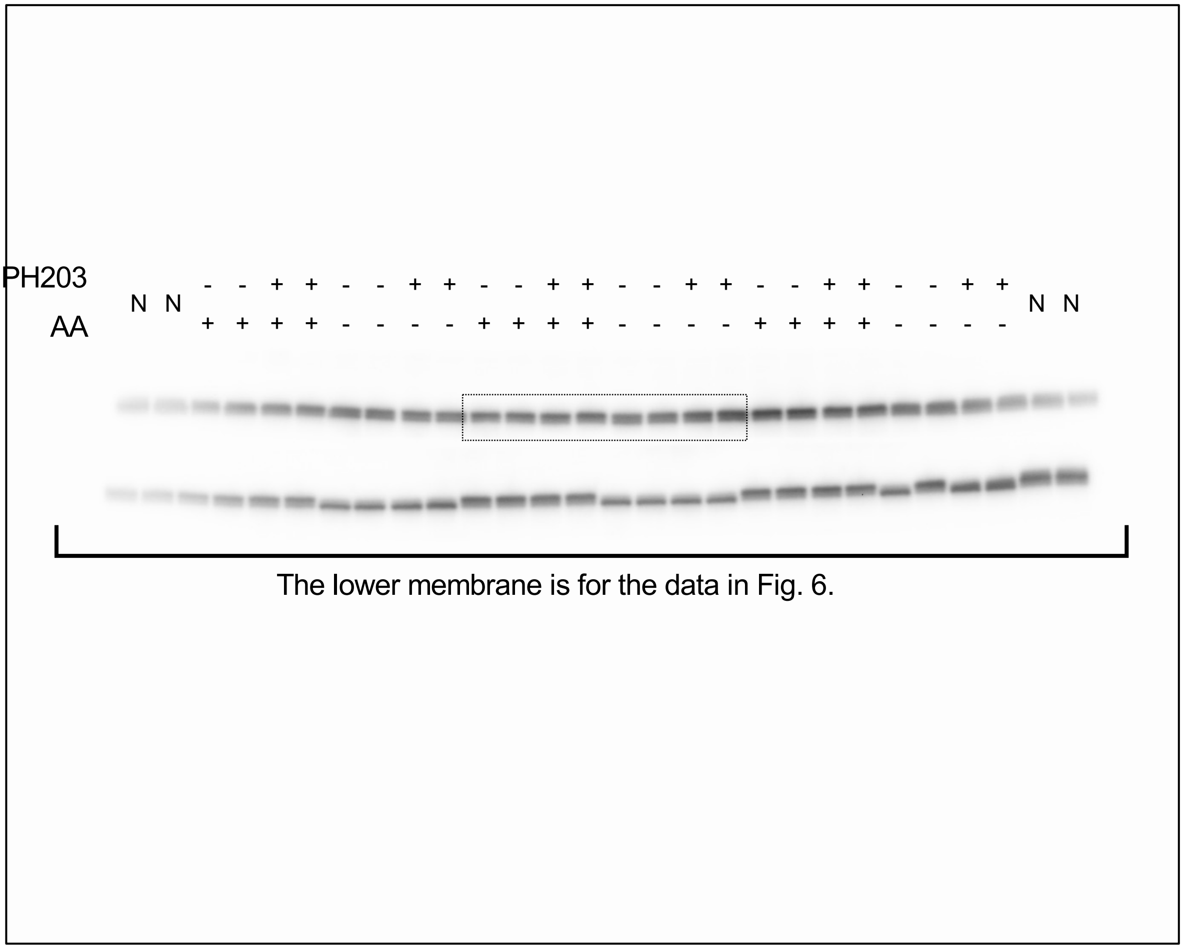

Bright field

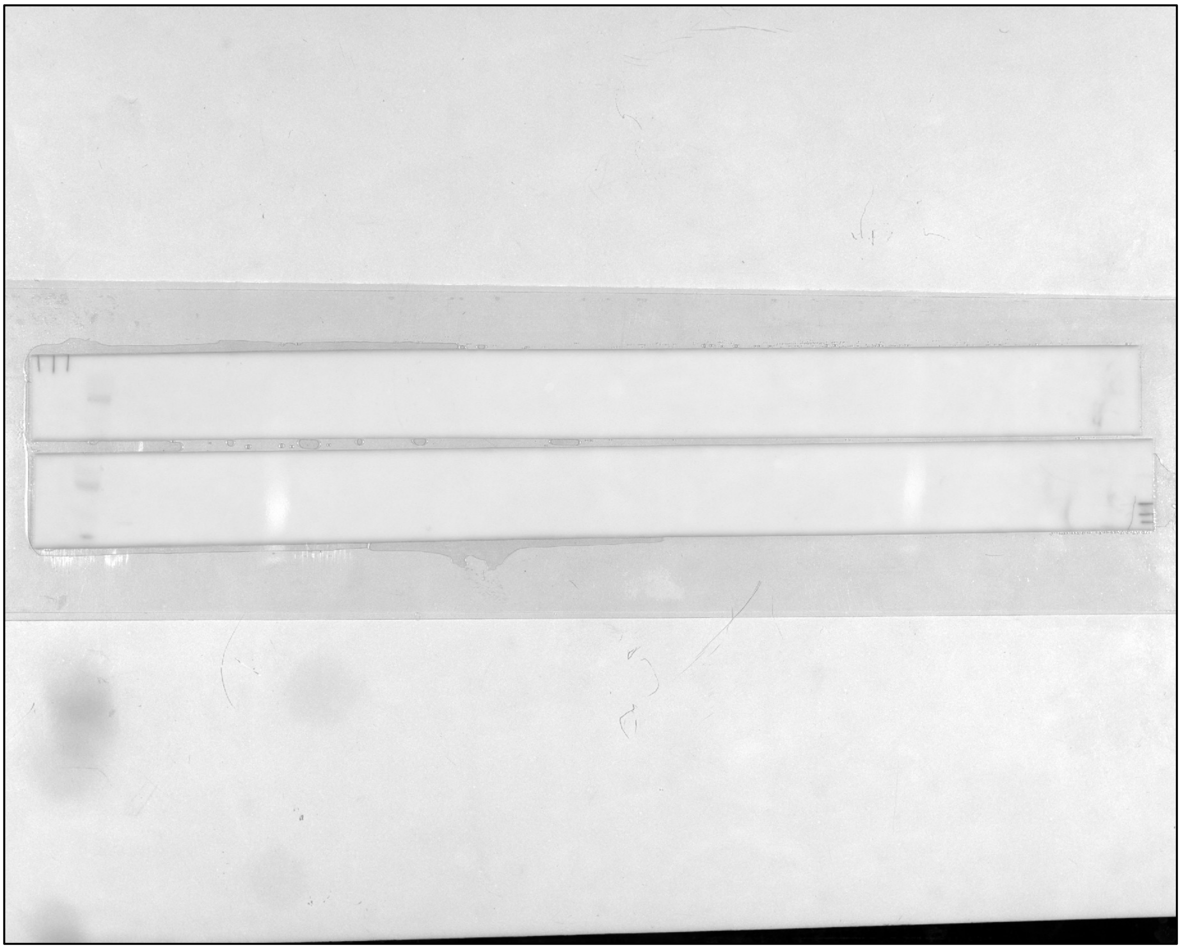

Band images in Fig. 5 (continued).

T-4EBP1

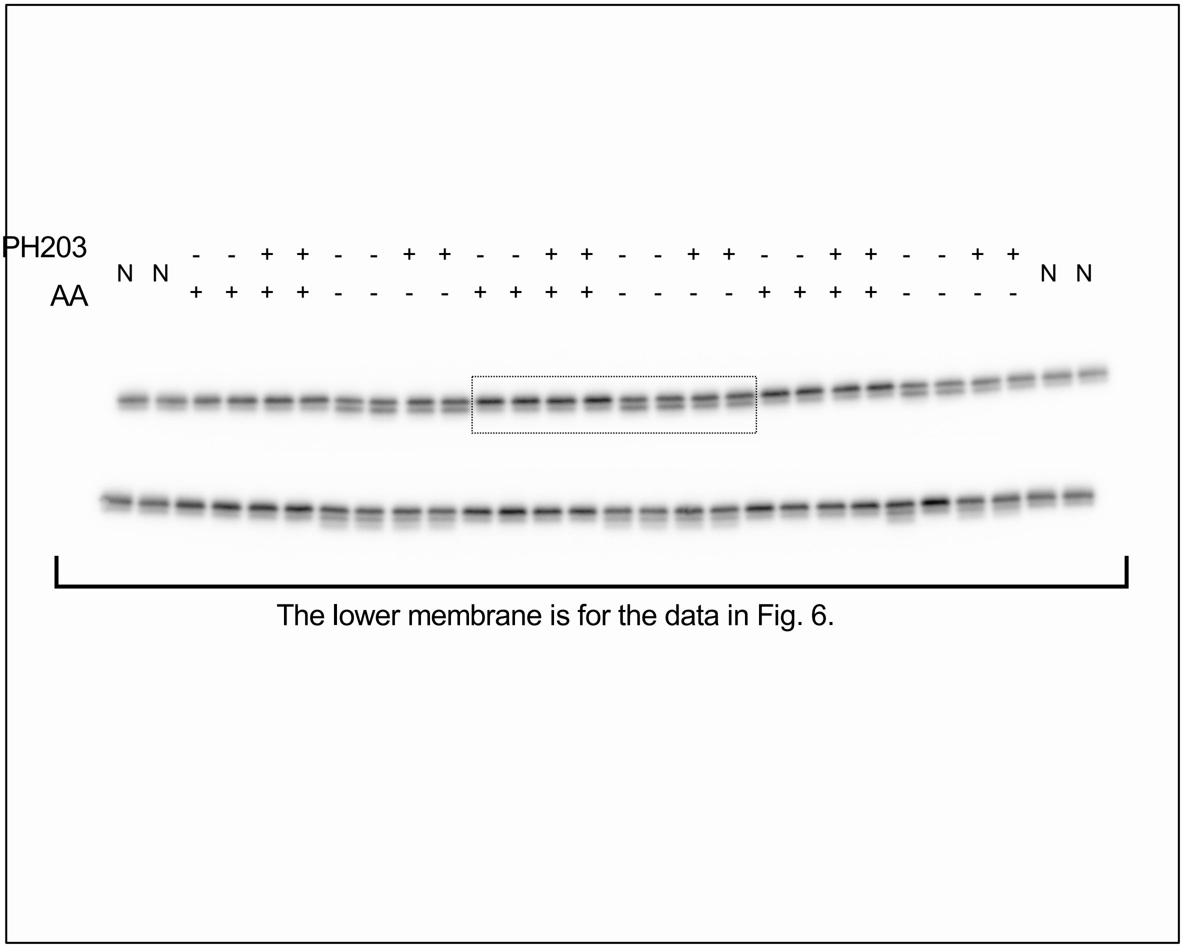

Bright field

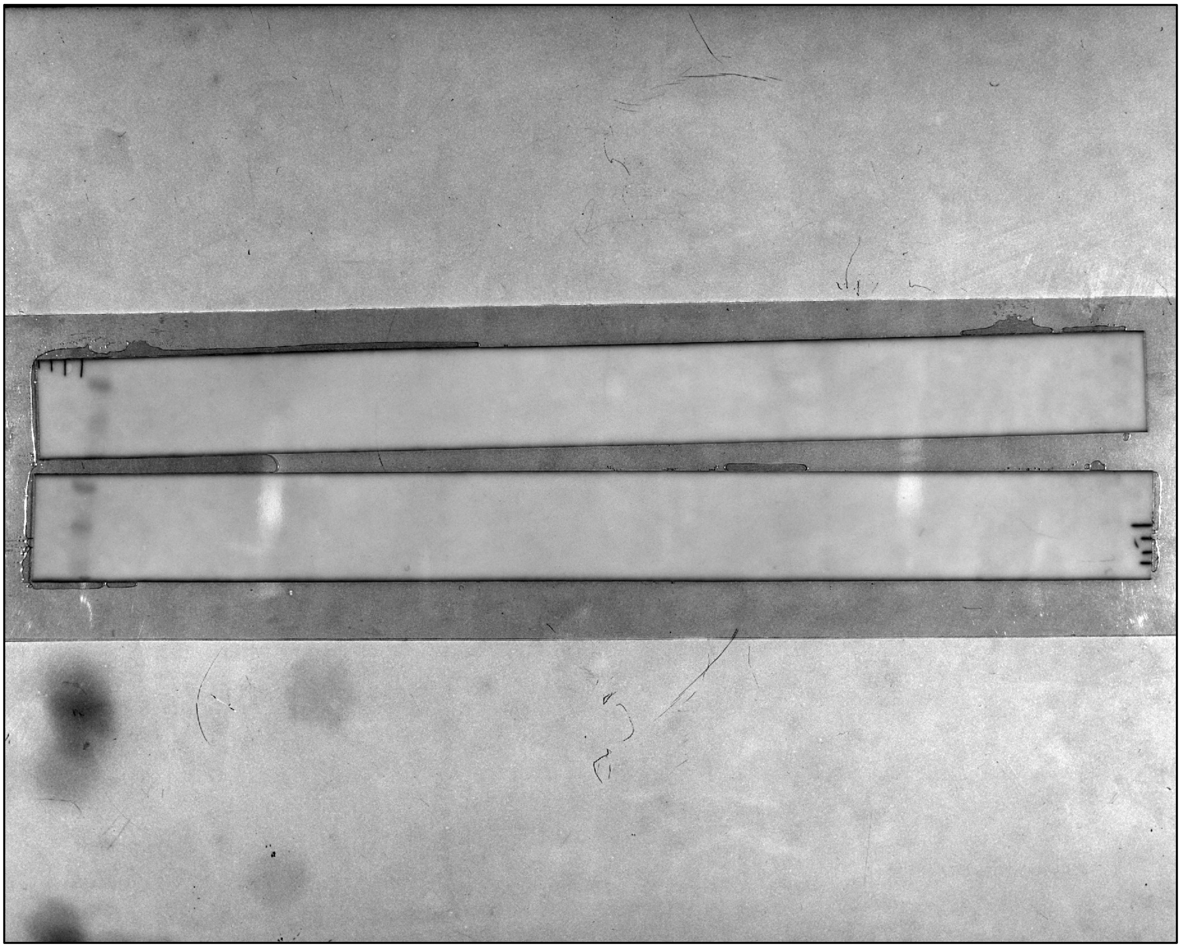

Band images in Fig. 5 (continued).

CBB

For P-p70S6K, P-rpS6, and P-4EBP1.

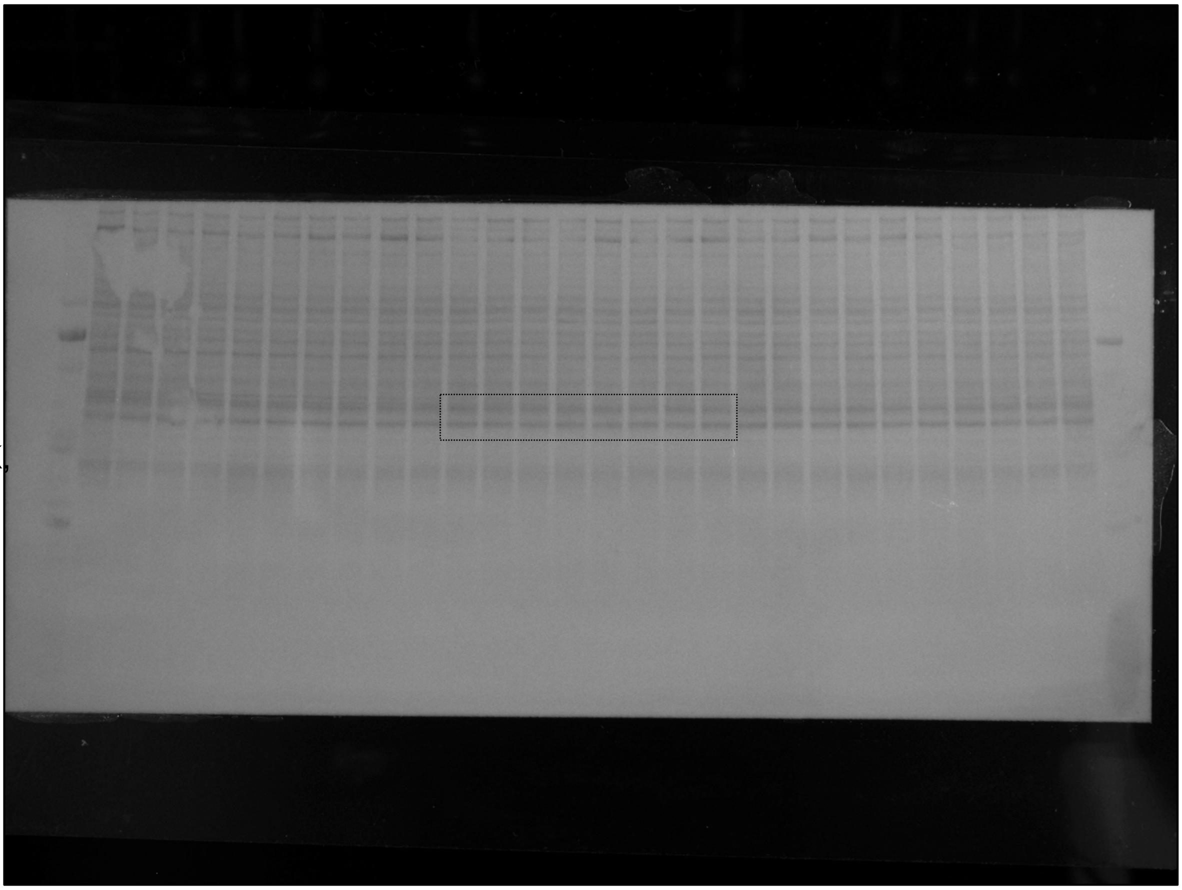

CBB

For T-p70S6K, T-rpS6, and T-4EBP1.

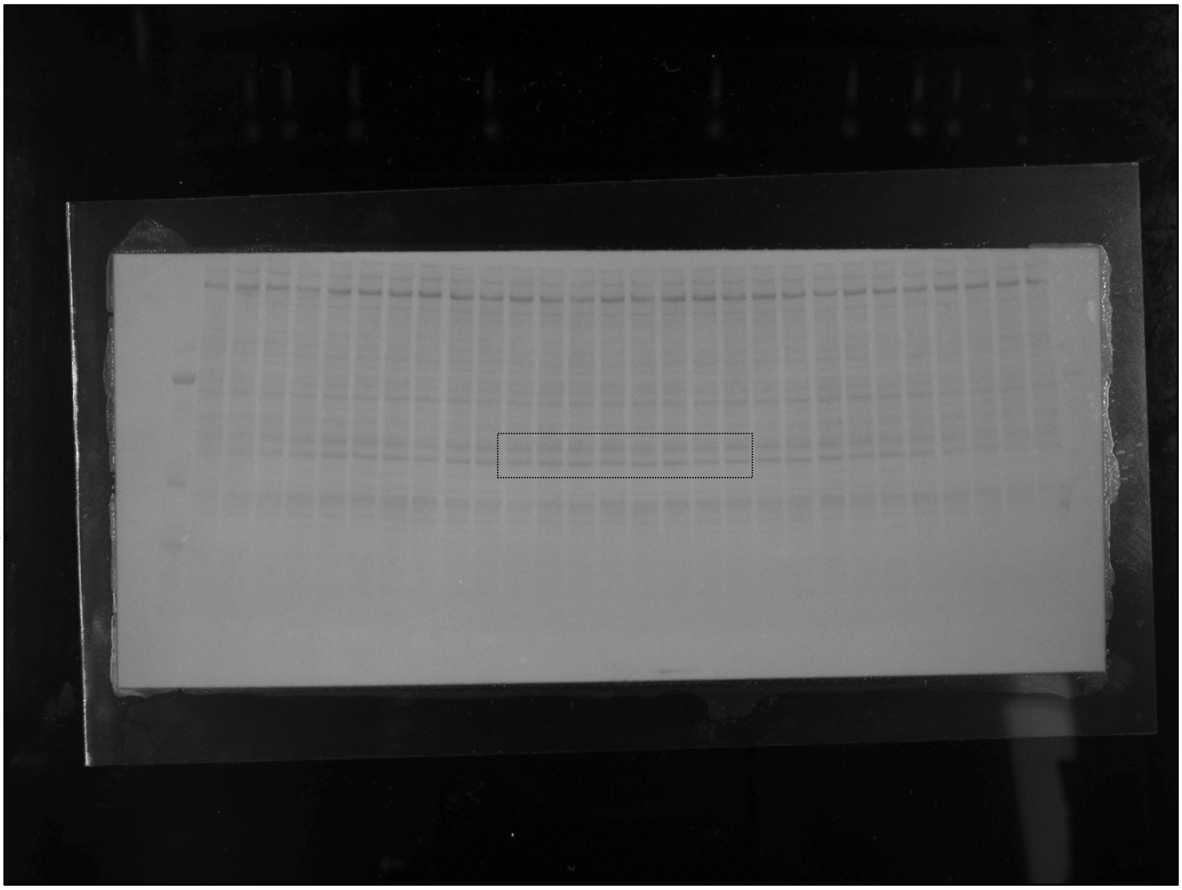

Band images in Fig. 6.

P-p70S6K

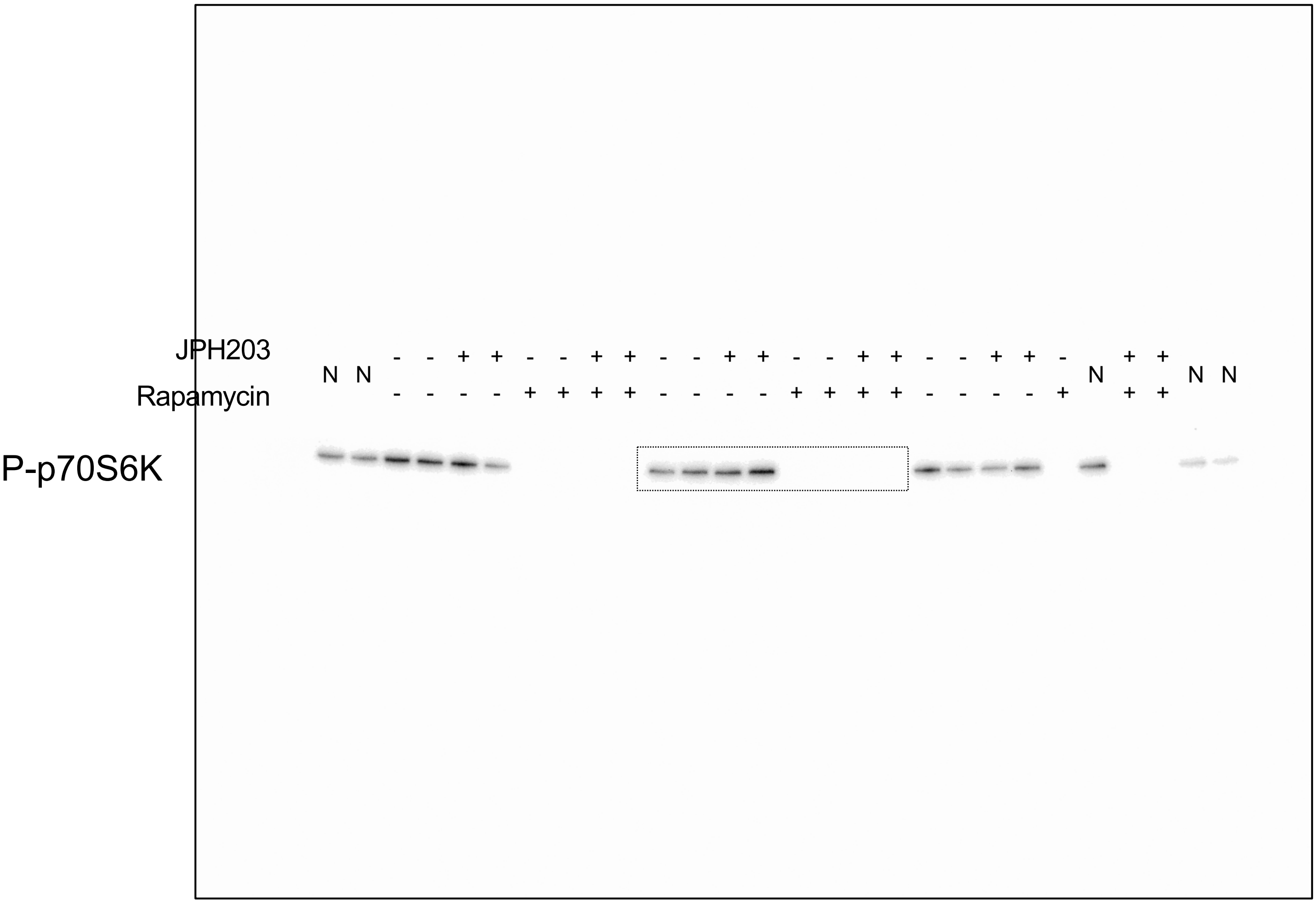

Bright field

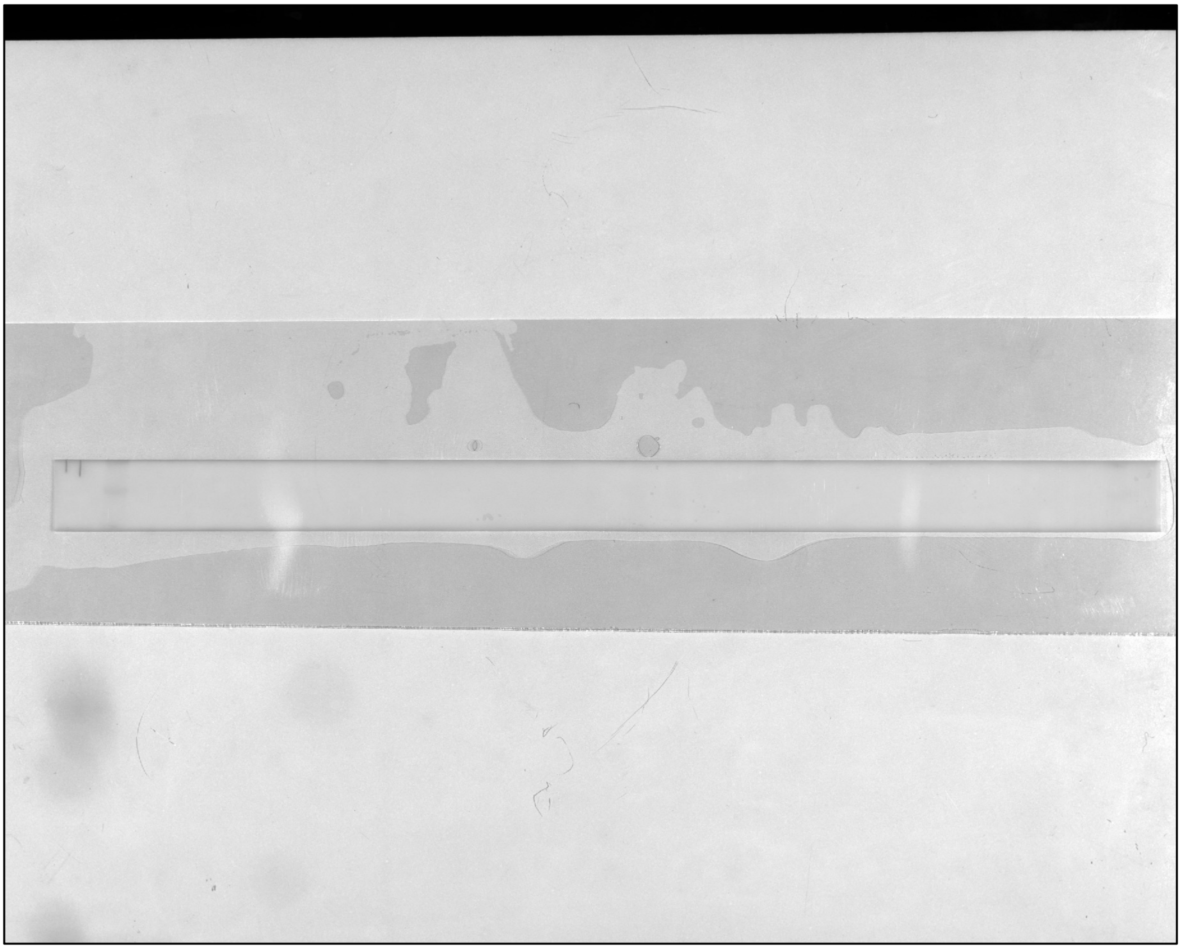

Band images in Fig. 6 (continued).

P-rpS6

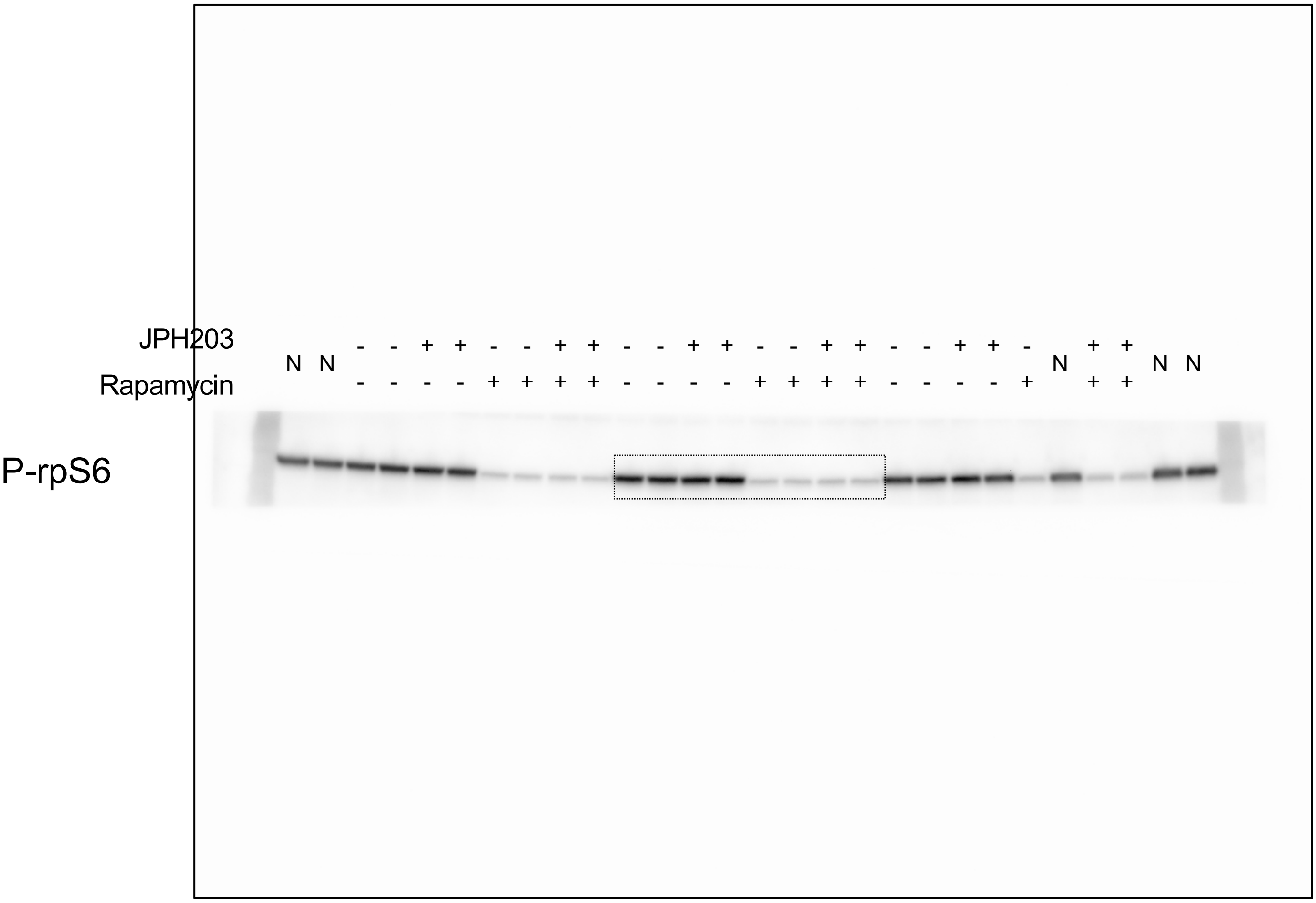

Bright field

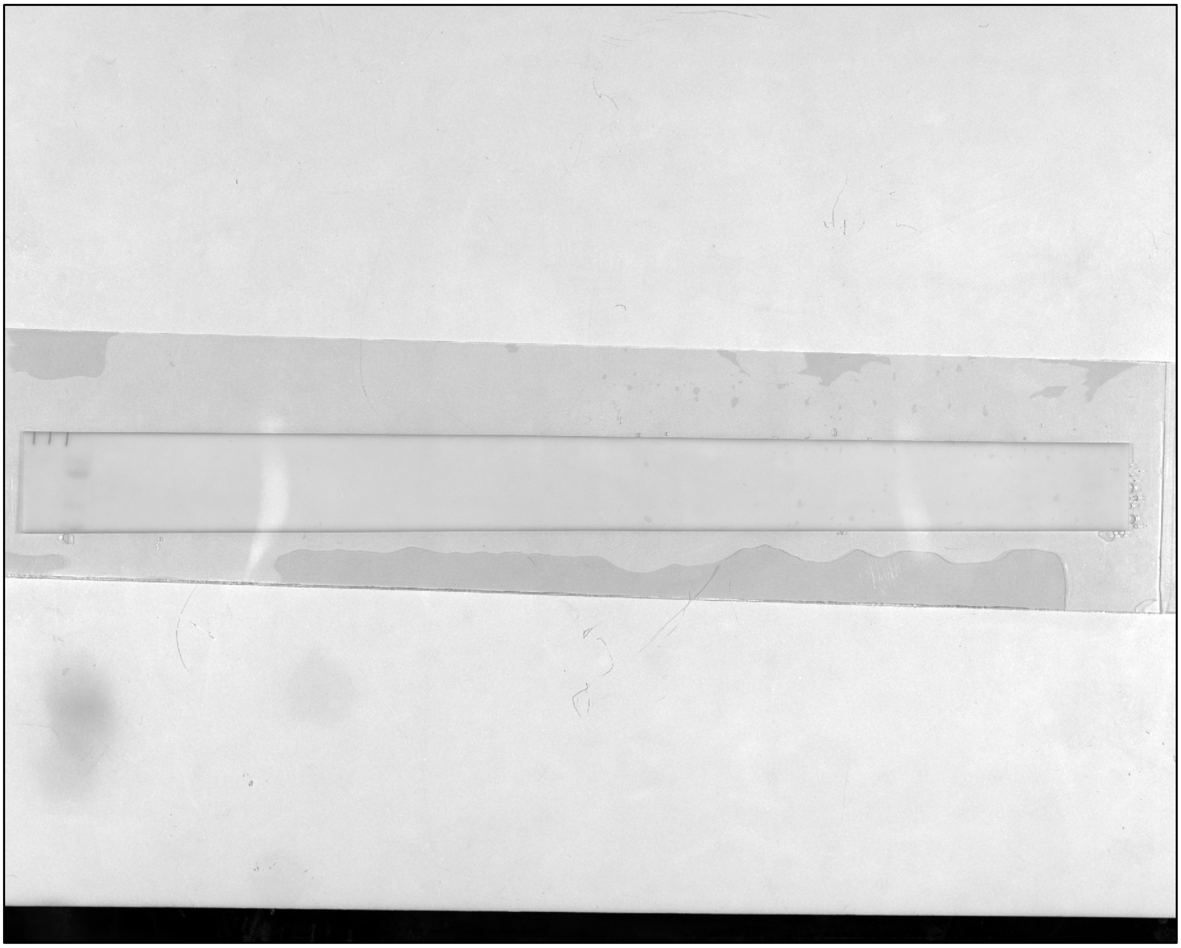

Band images in Fig. 6 (continued).

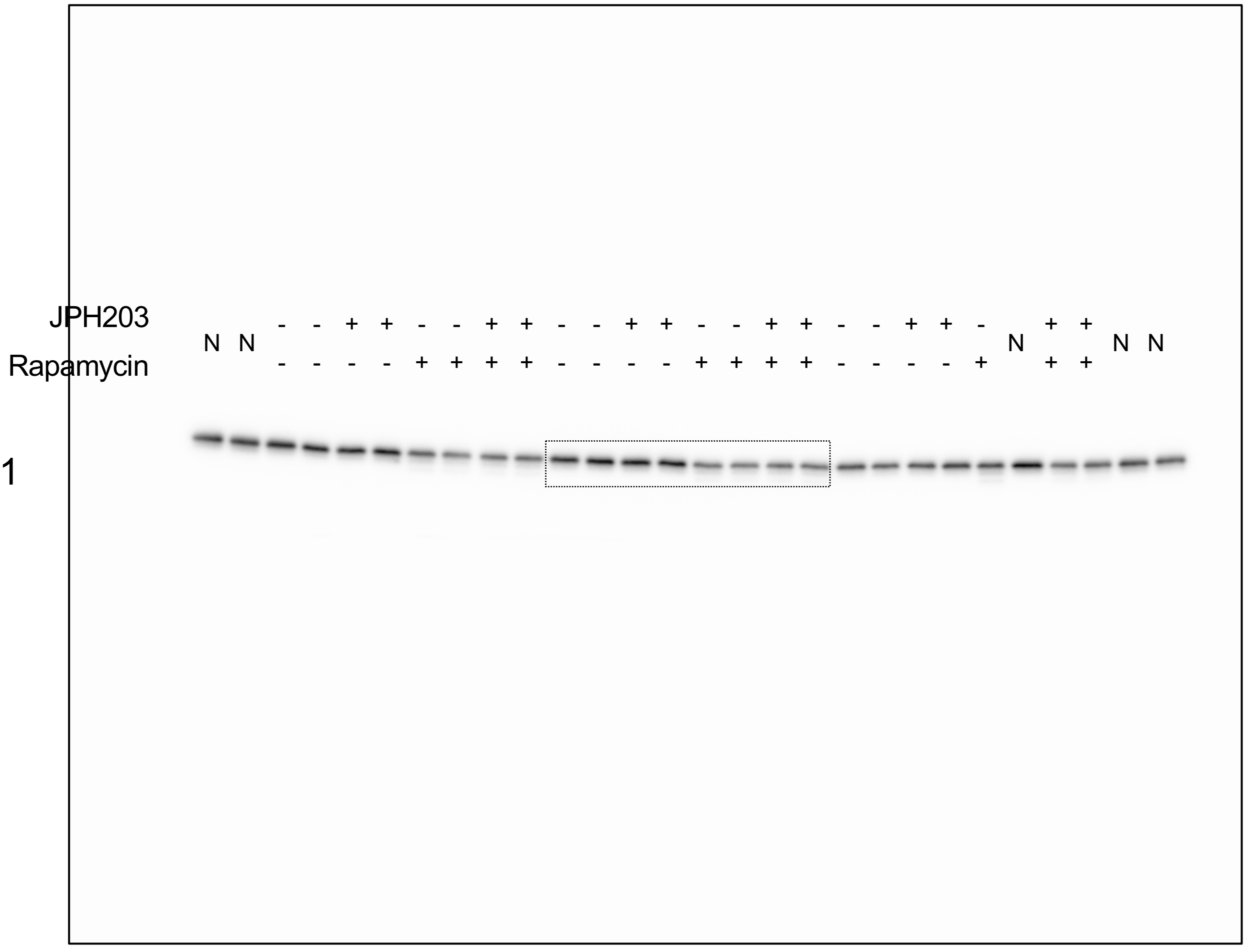

P-4EBP1

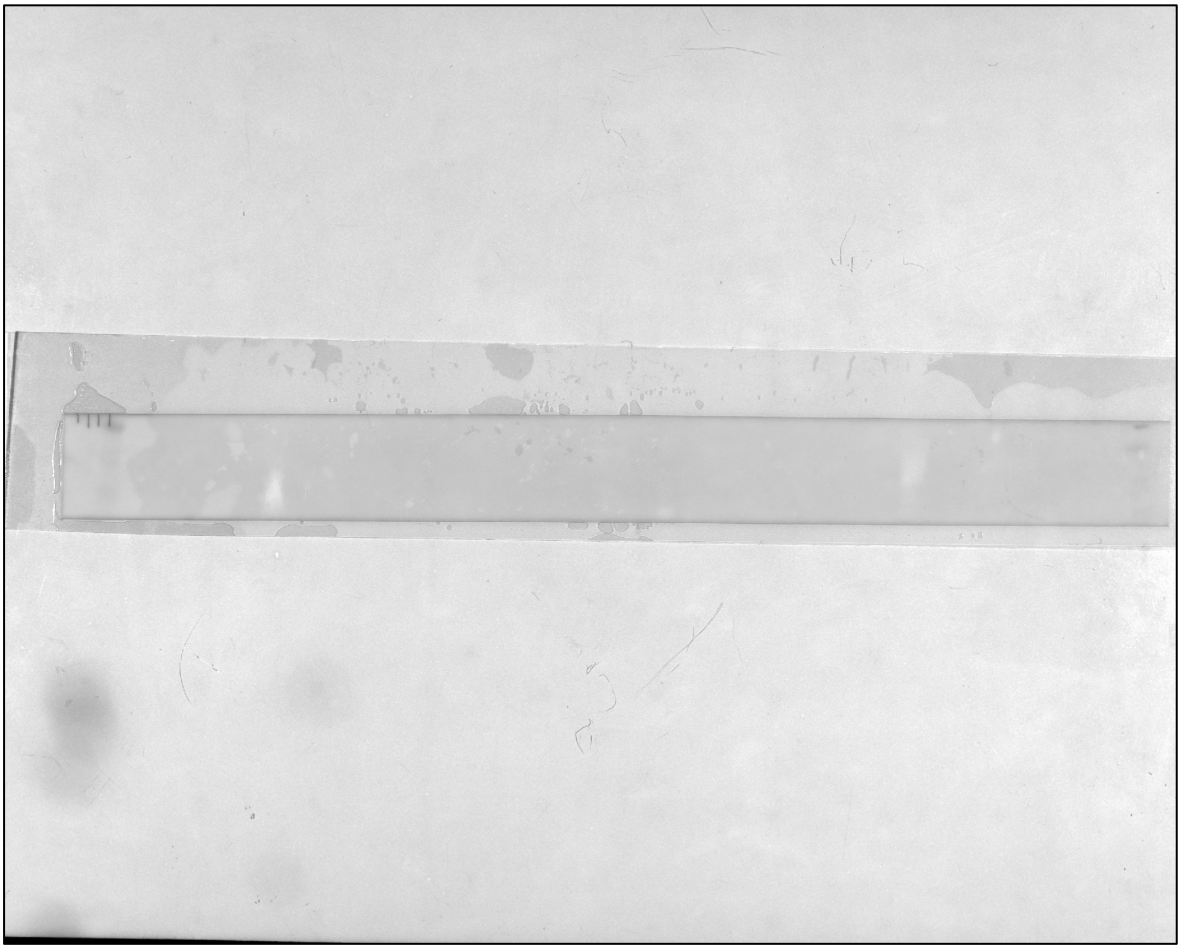

Bright field

Band images in Fig. 6 (continued).

T-p70S6K

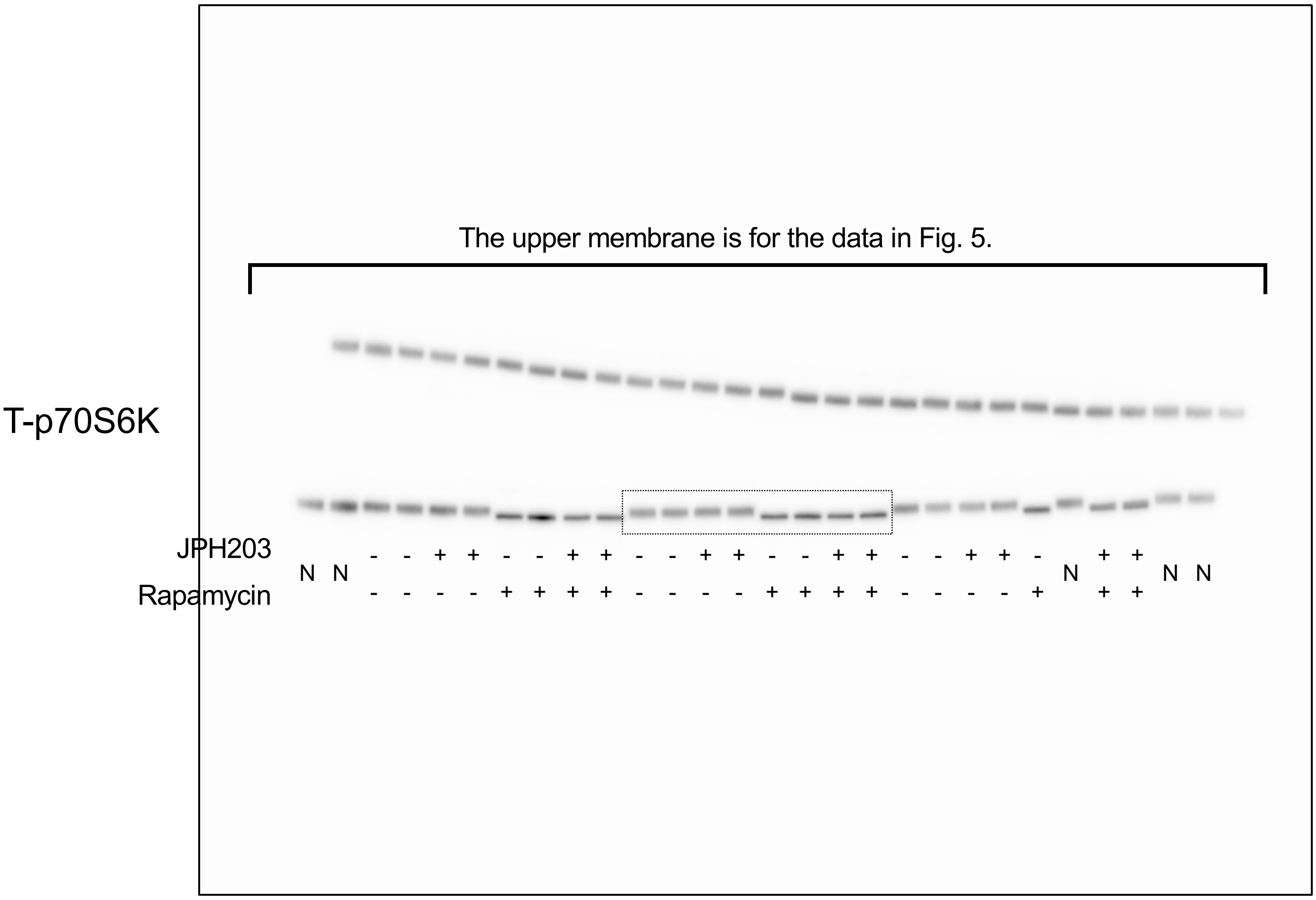

Bright field

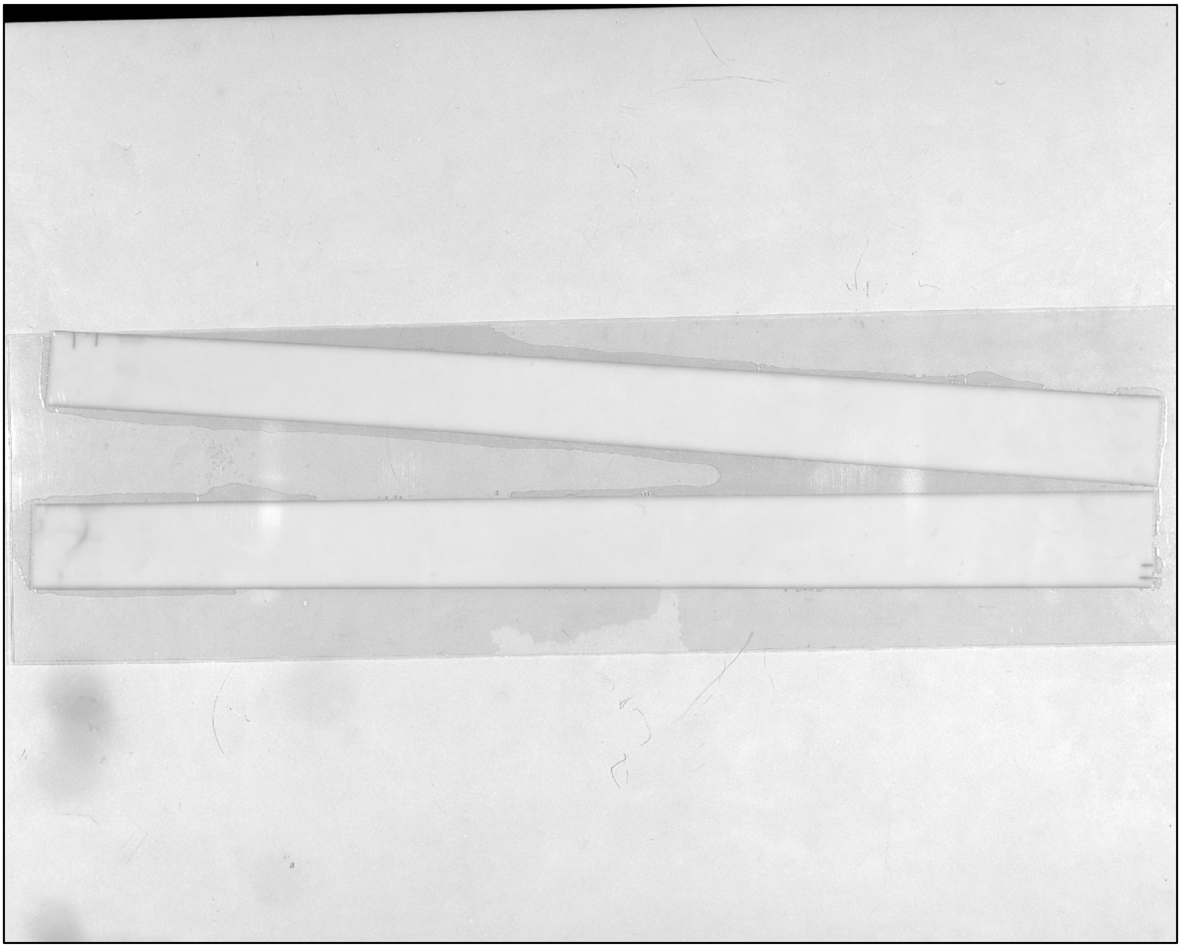

Band images in Fig. 6 (continued).

T-rpS6

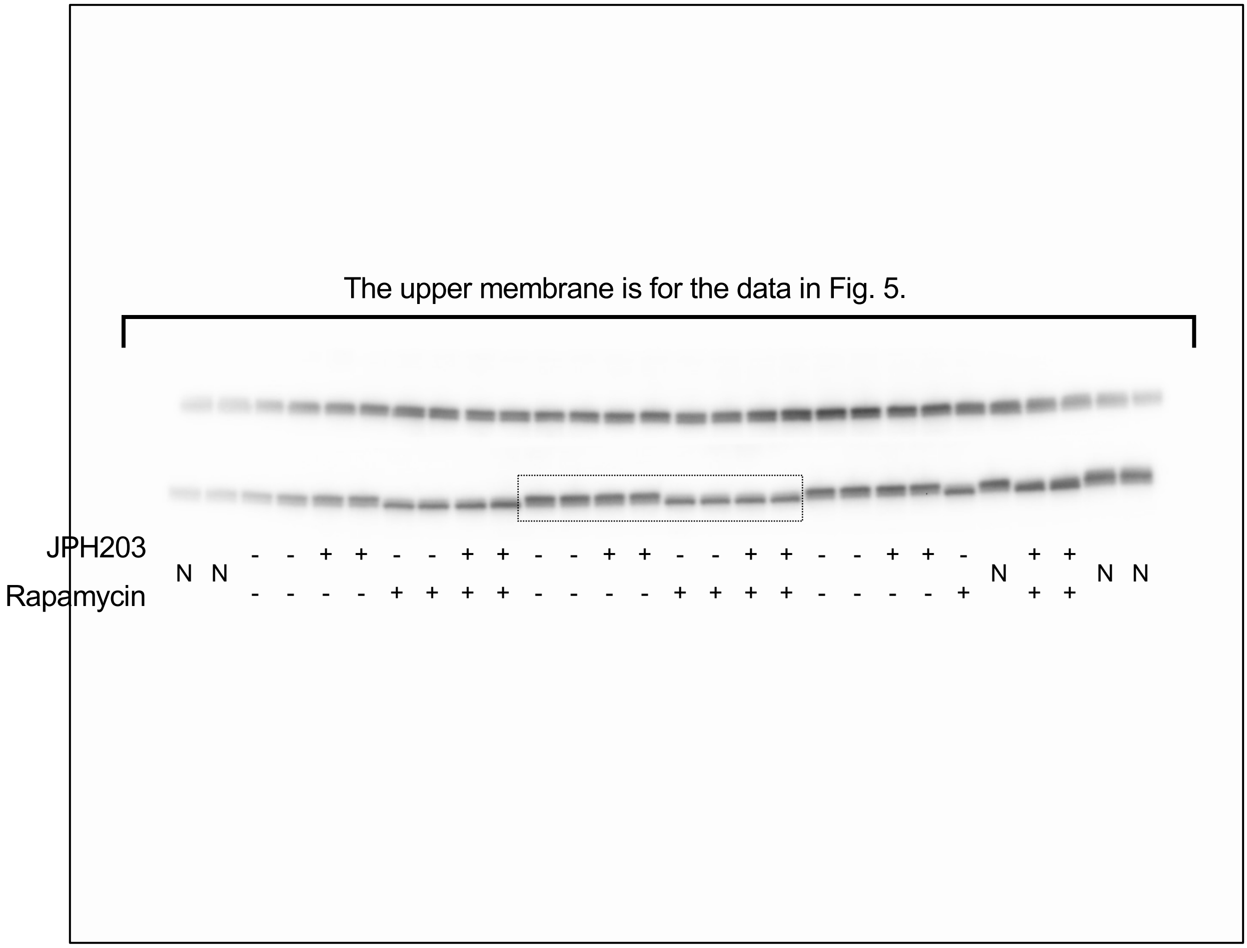

Bright field

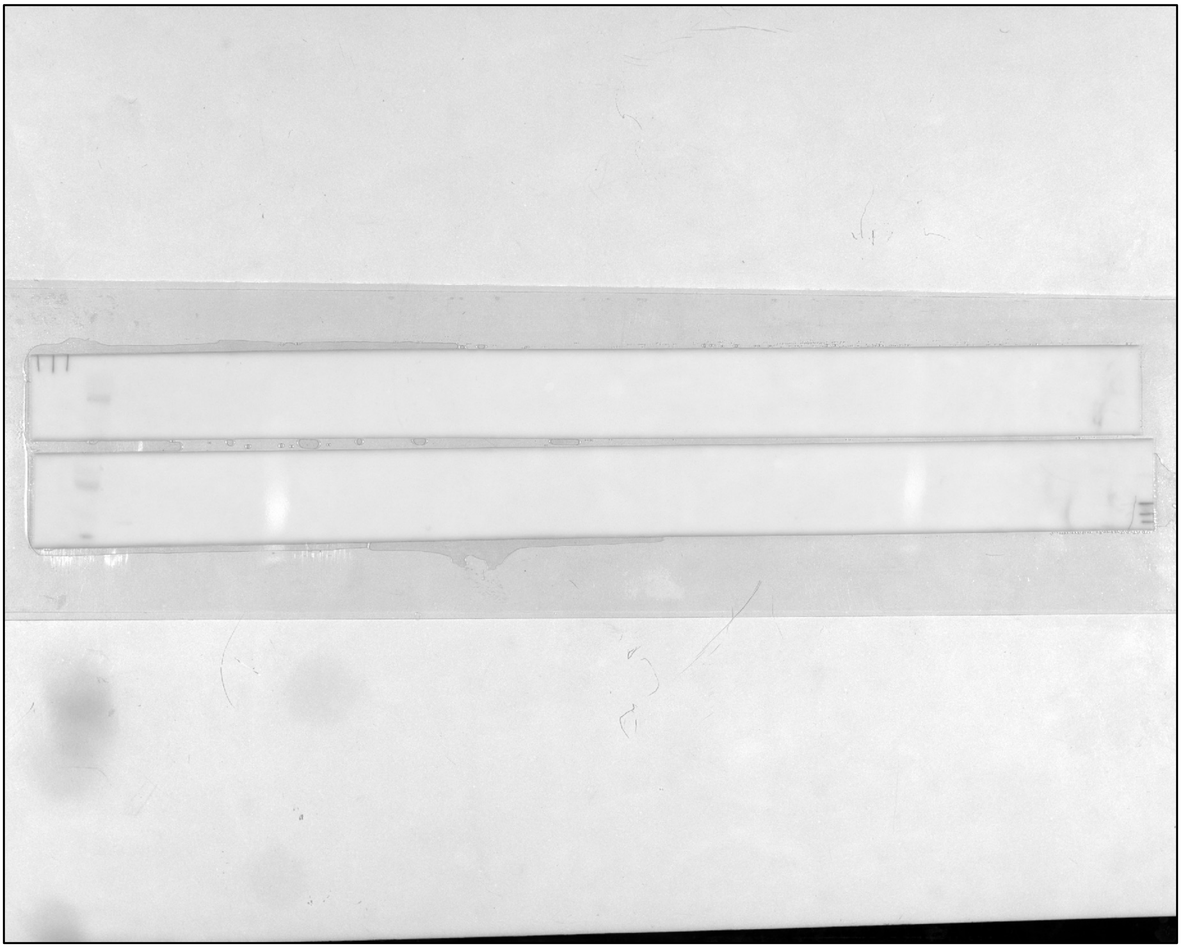

Band images in Fig. 6 (continued).

T-4EBP1

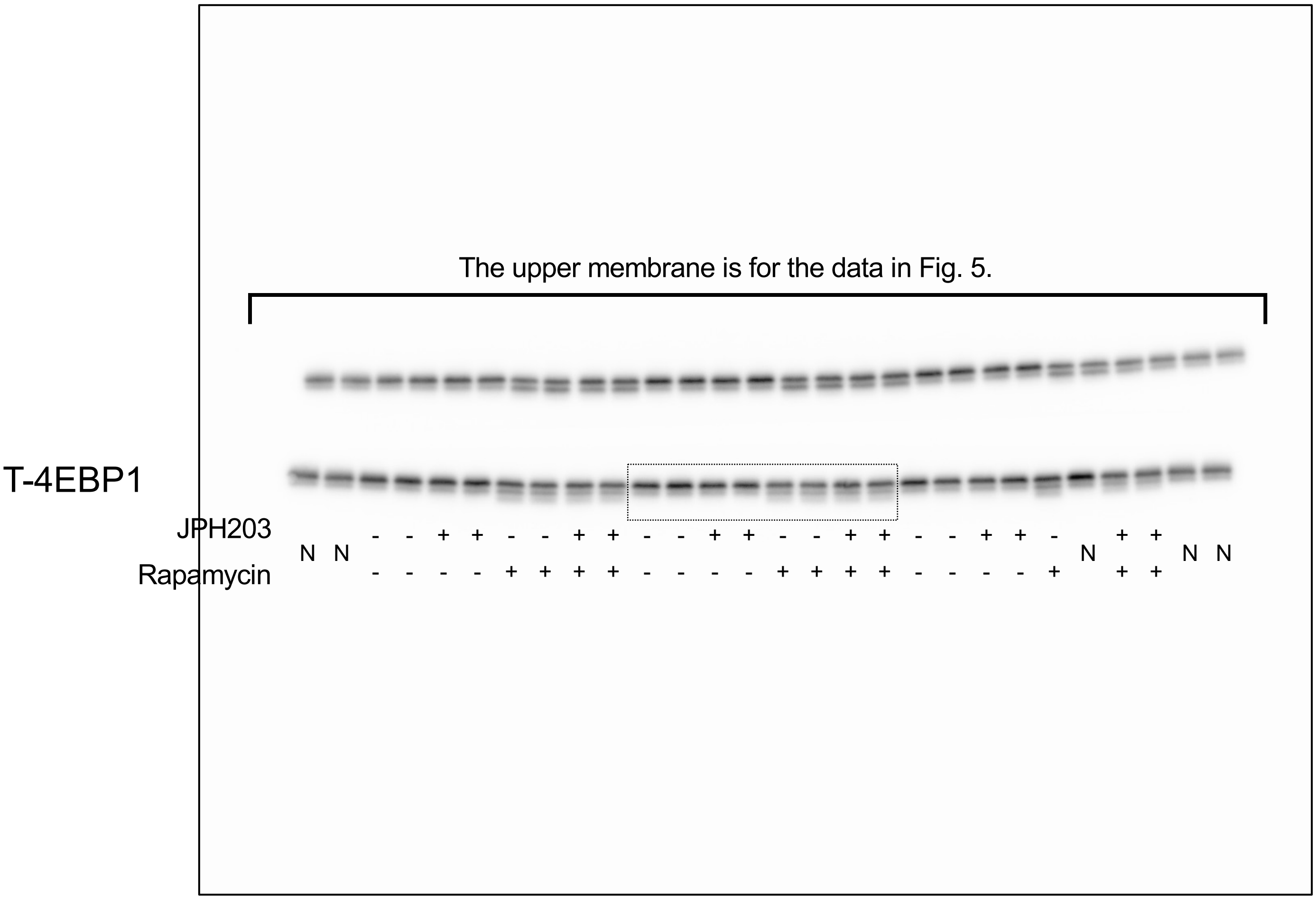

Bright field

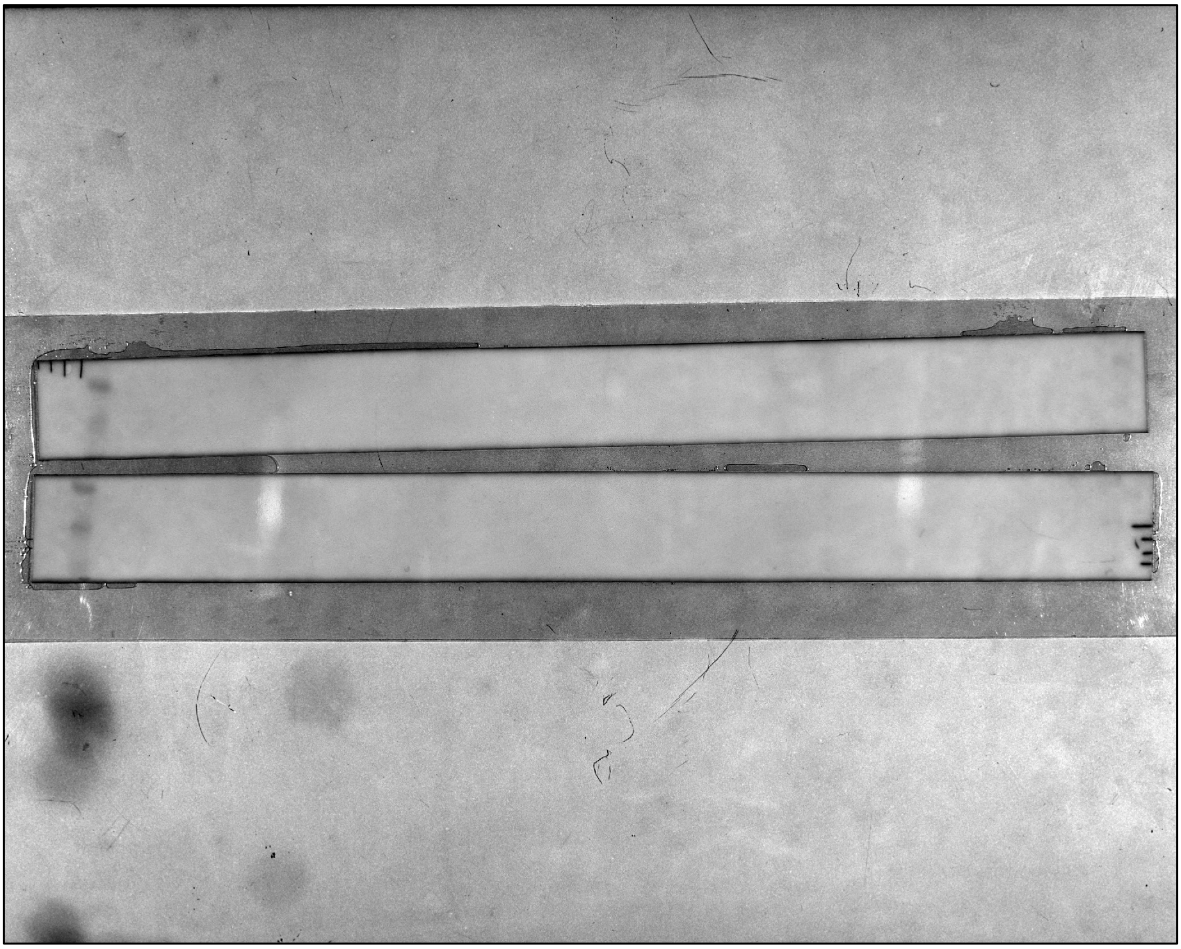

Band images in Fig. 6 (continued).

CBB

For P-p70S6K, P-rpS6, and P-4EBP1.

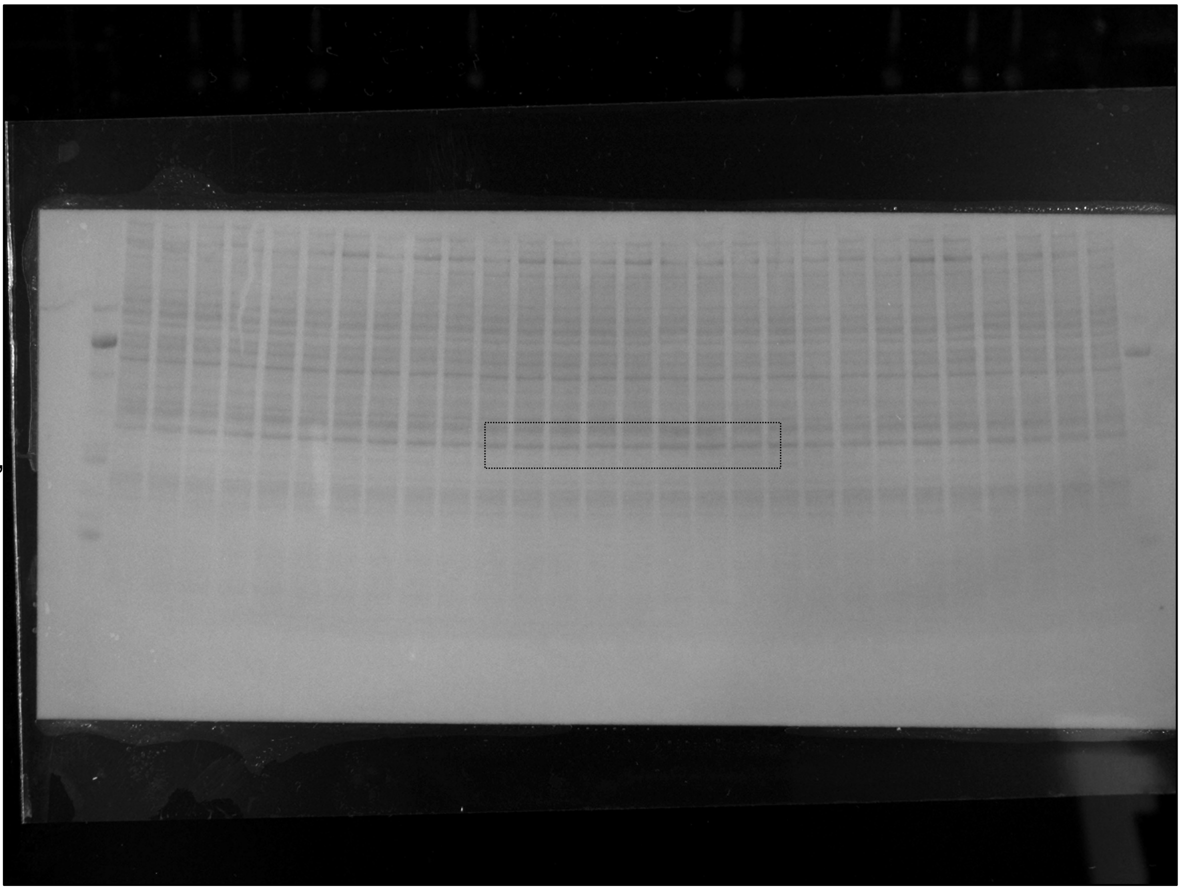

CBB

For T-p70S6K, T-rpS6, and T-4EBP1.

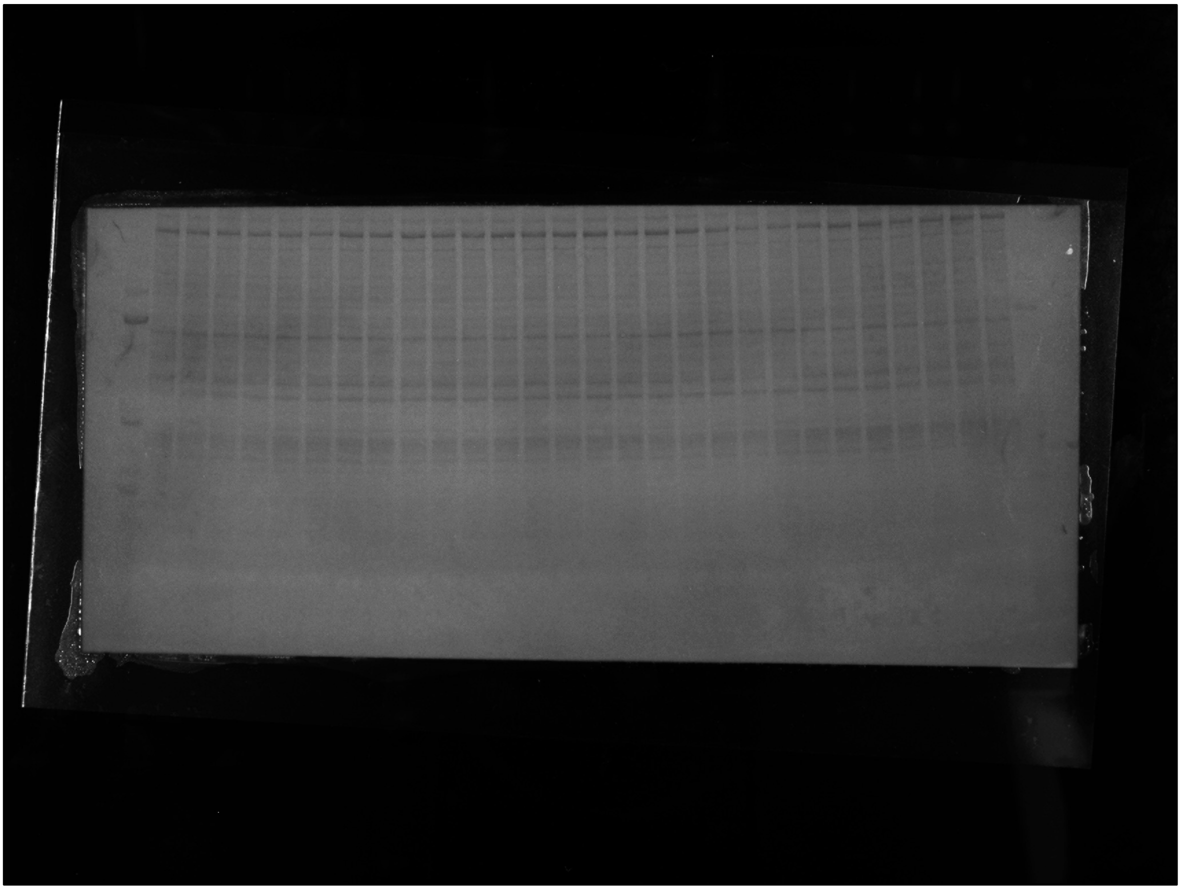

Band images in Fig. 6 (continued).

Puromycin

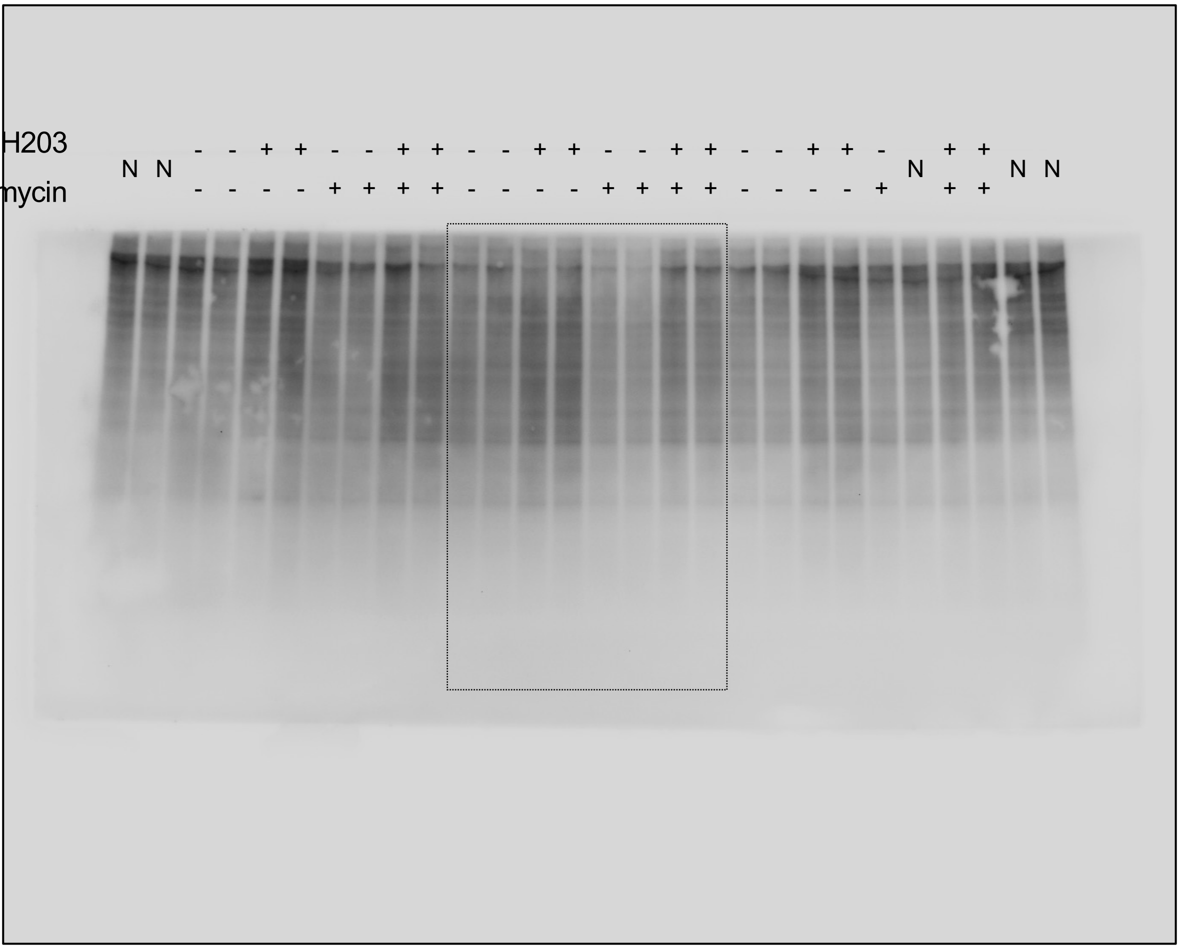

Bright field

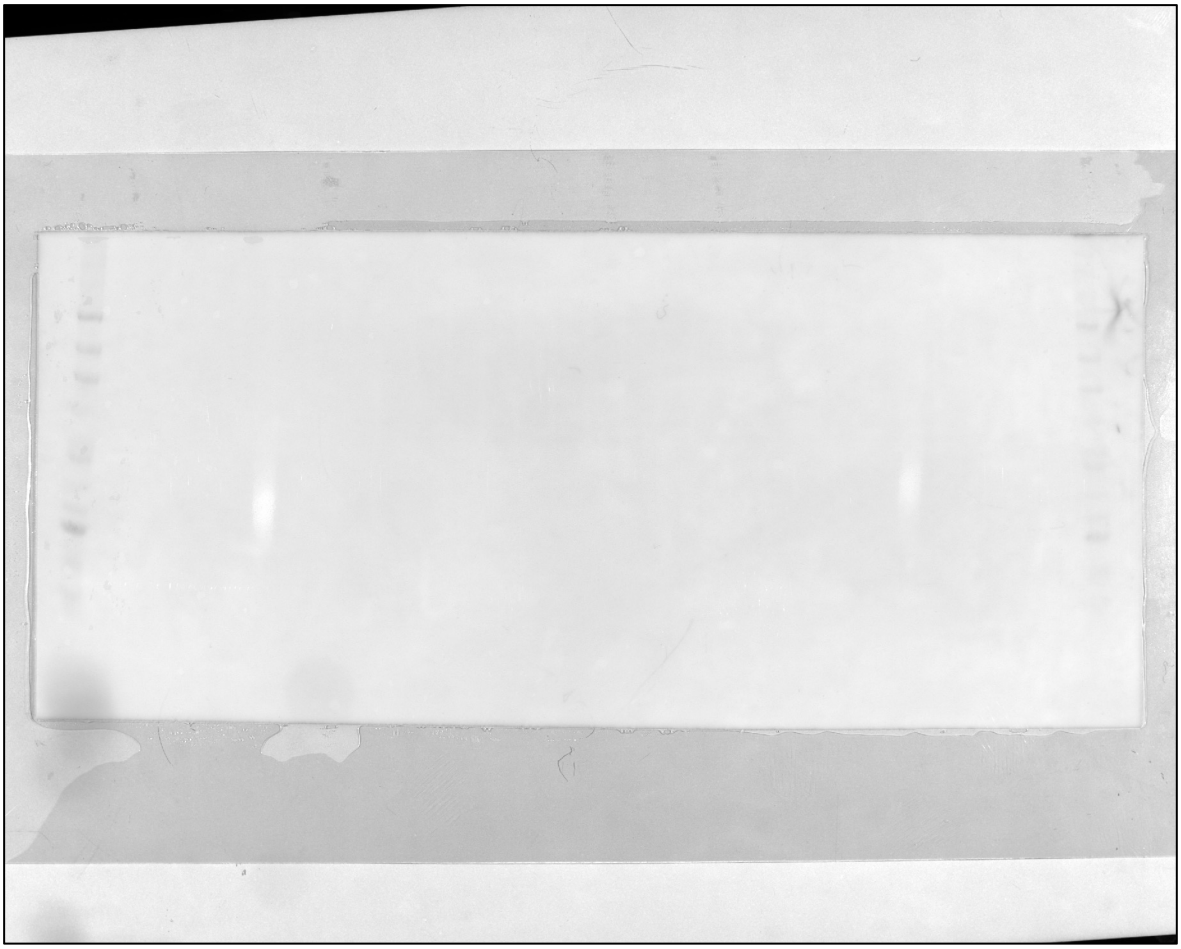

Band images in Fig. 6 (continued).

CBB

For puromycin

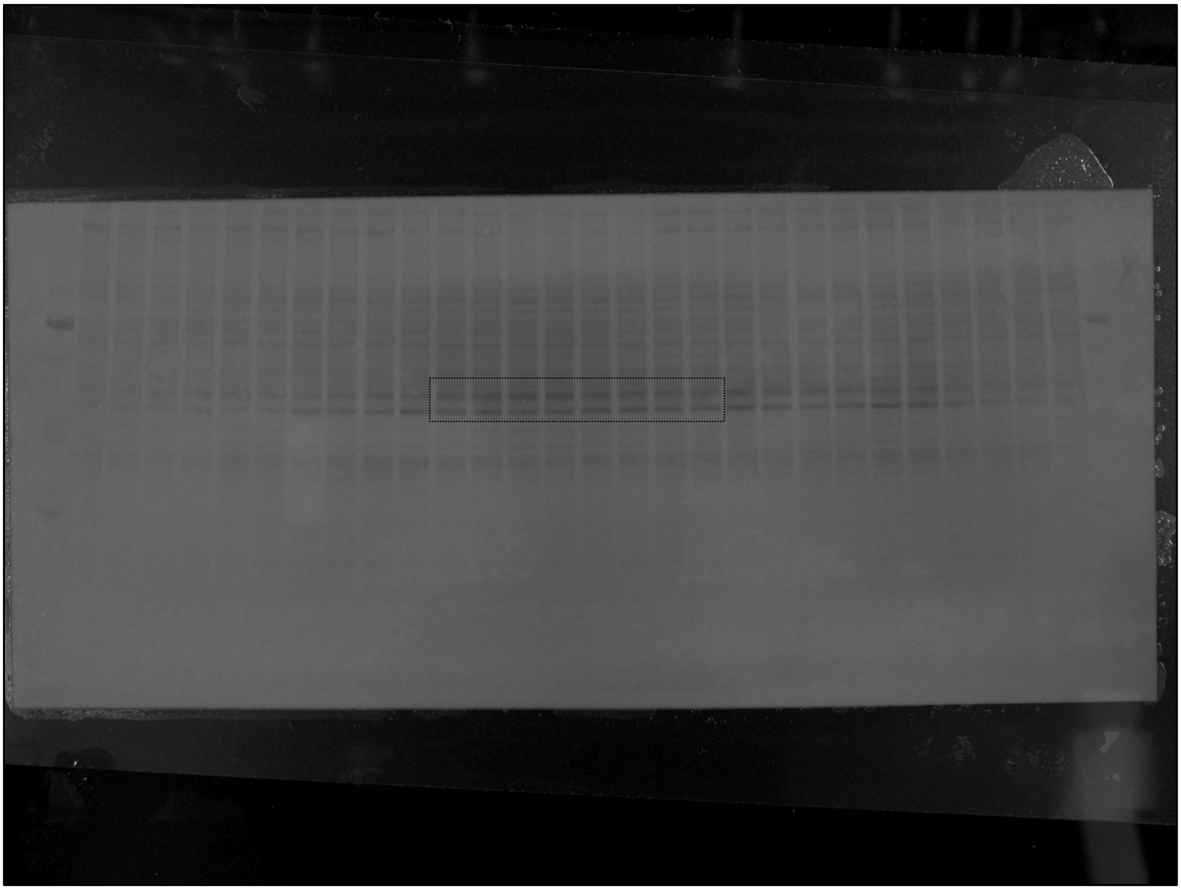

Band images in Fig. 7.

P-p70S6K

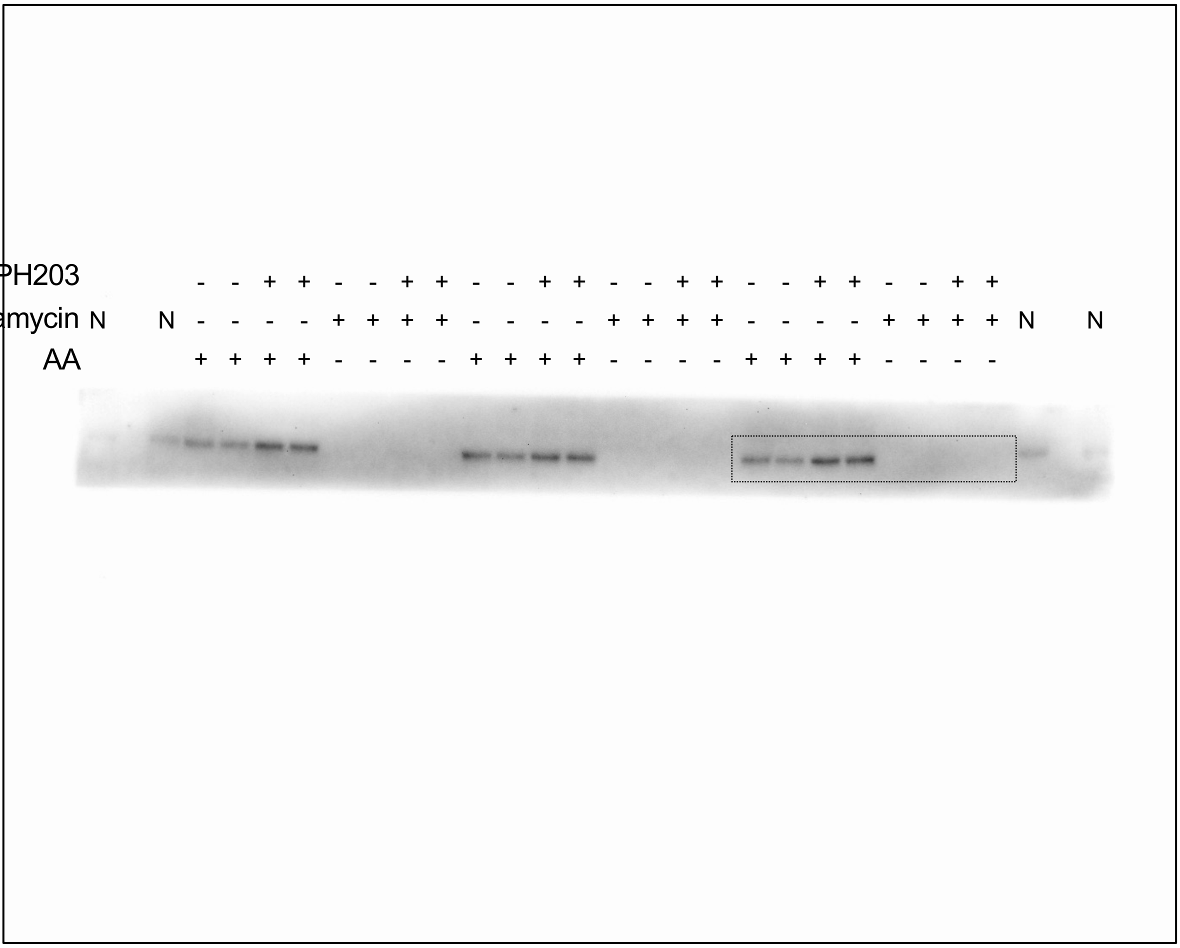

Bright field

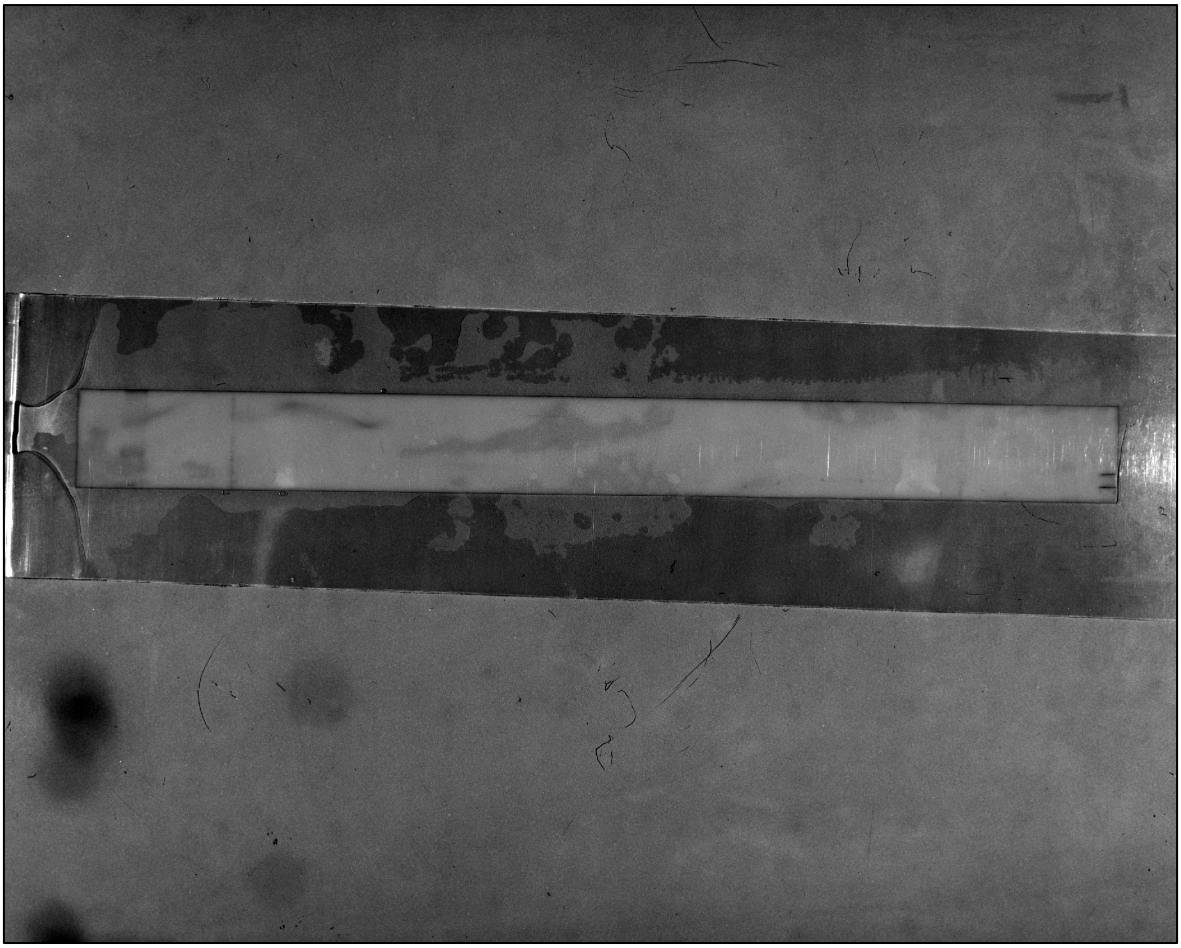

Band images in Fig. 7 (continued).

P-rpS6

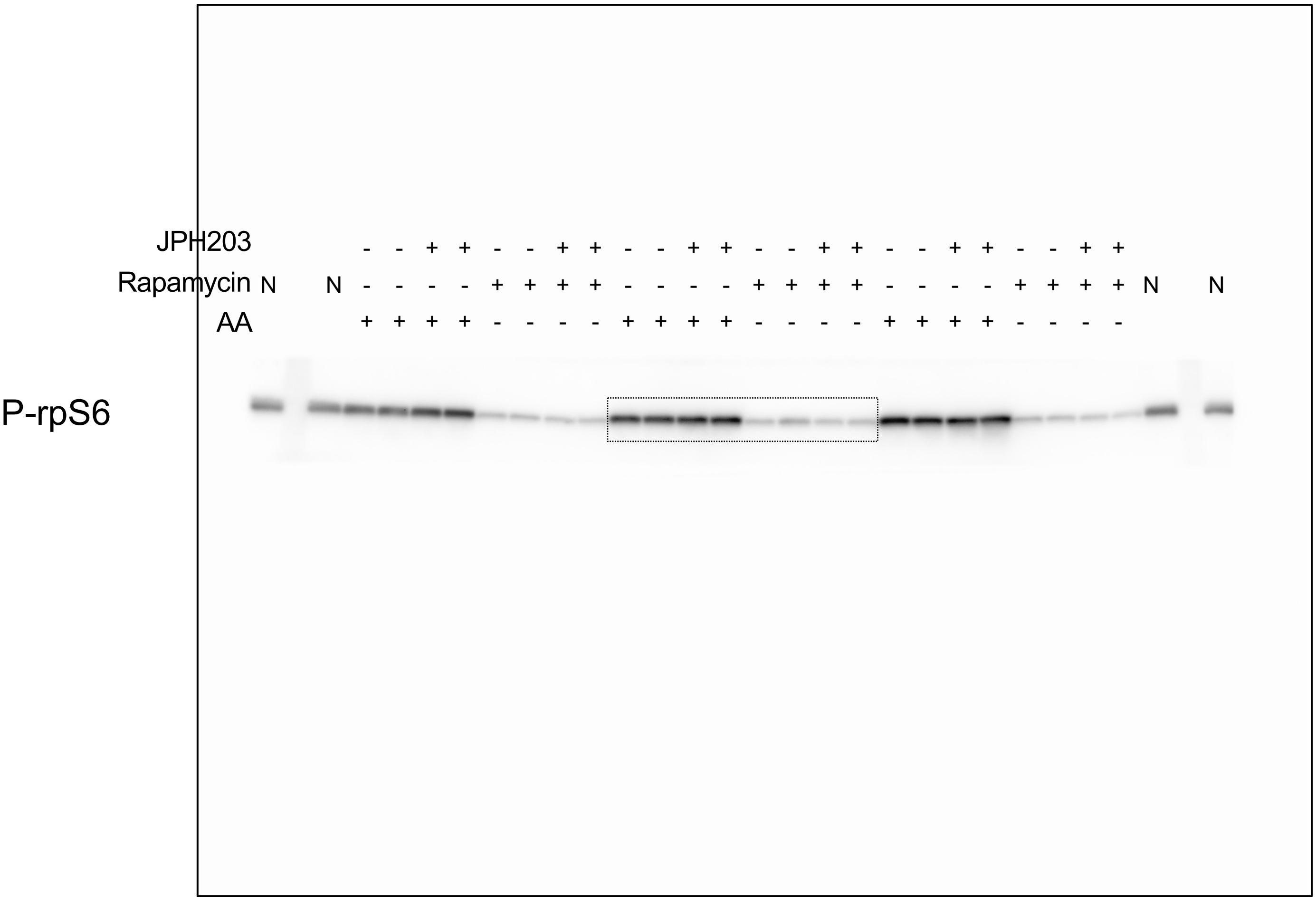

Bright field

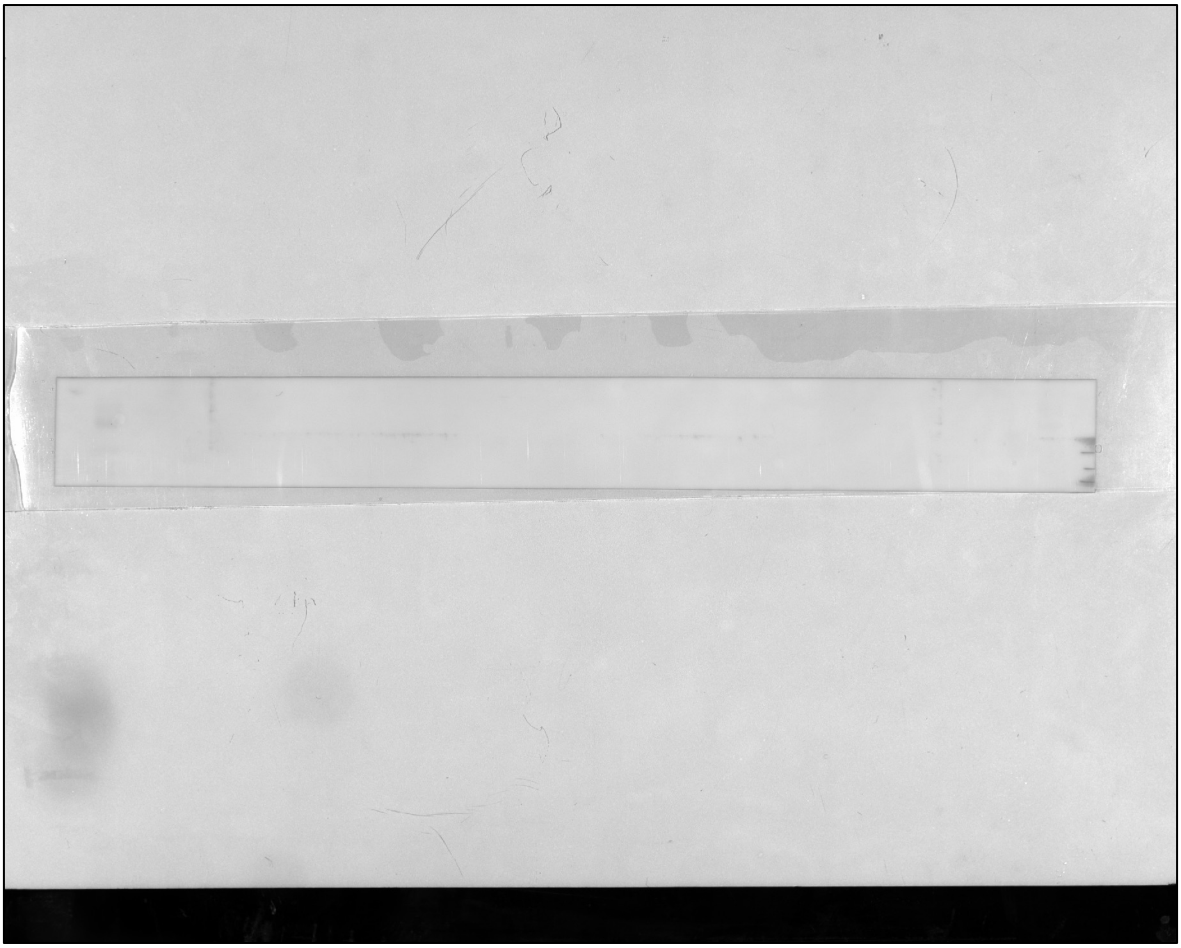

Band images in Fig. 7 (continued).

P-4EBP1

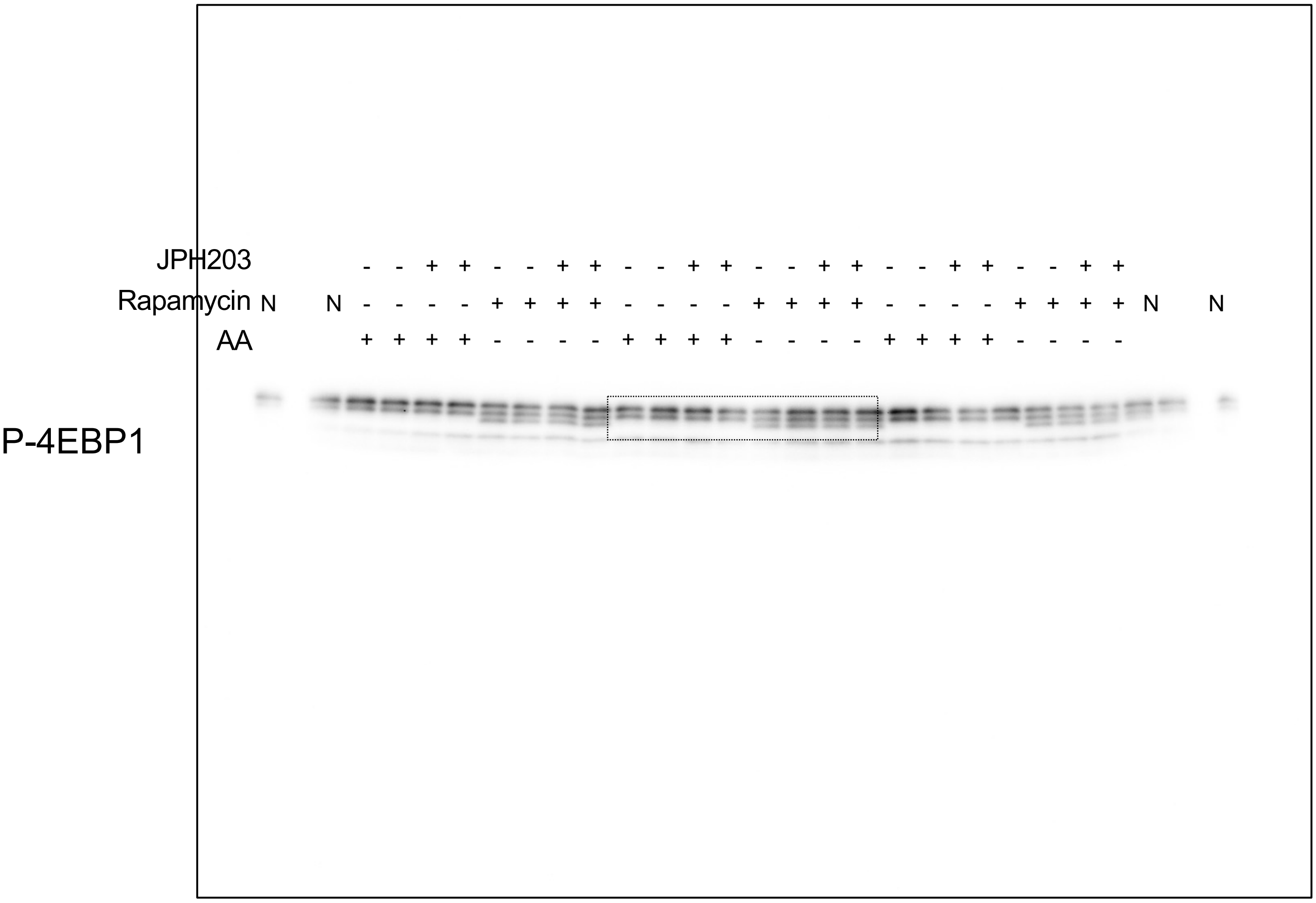

Bright field

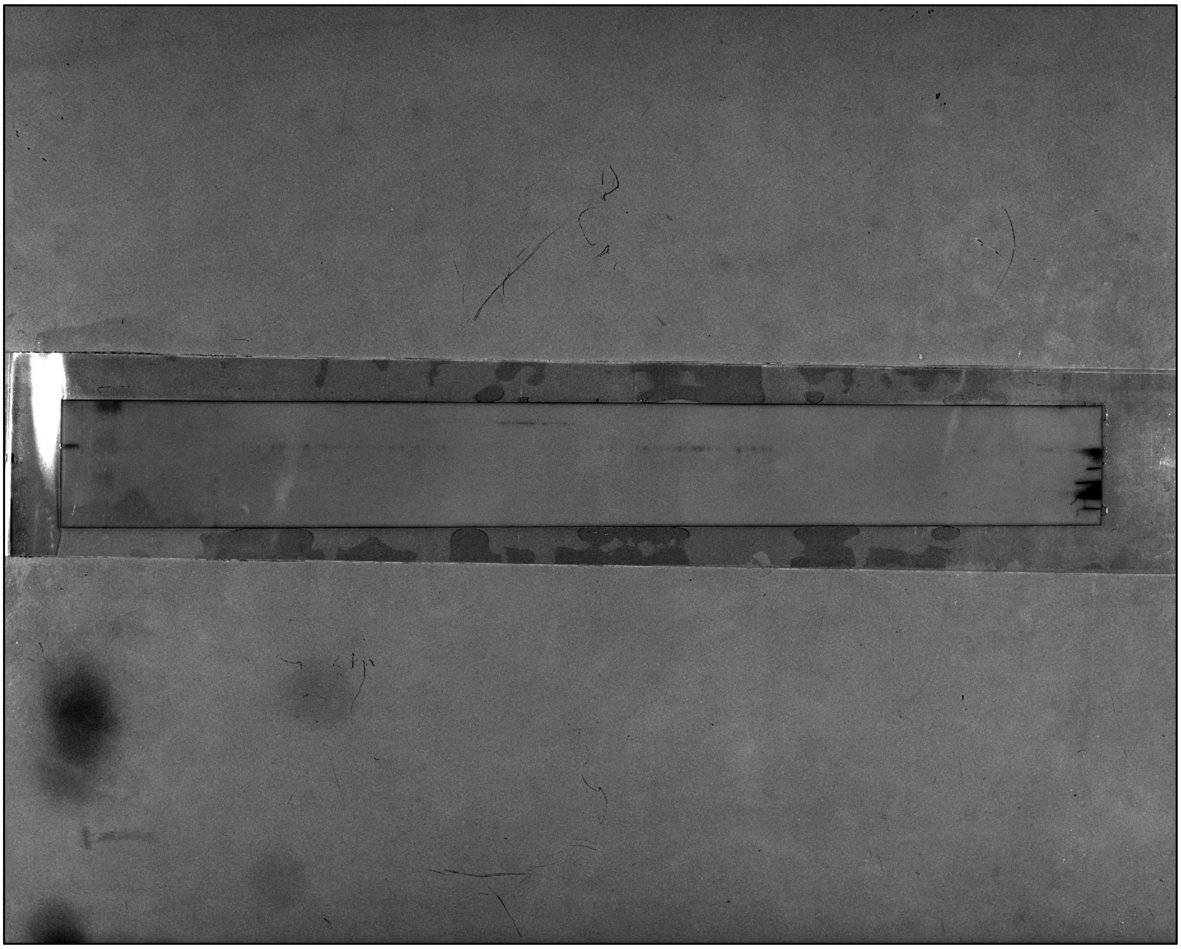

Band images in Fig. 7 (continued).

T-p70S6K

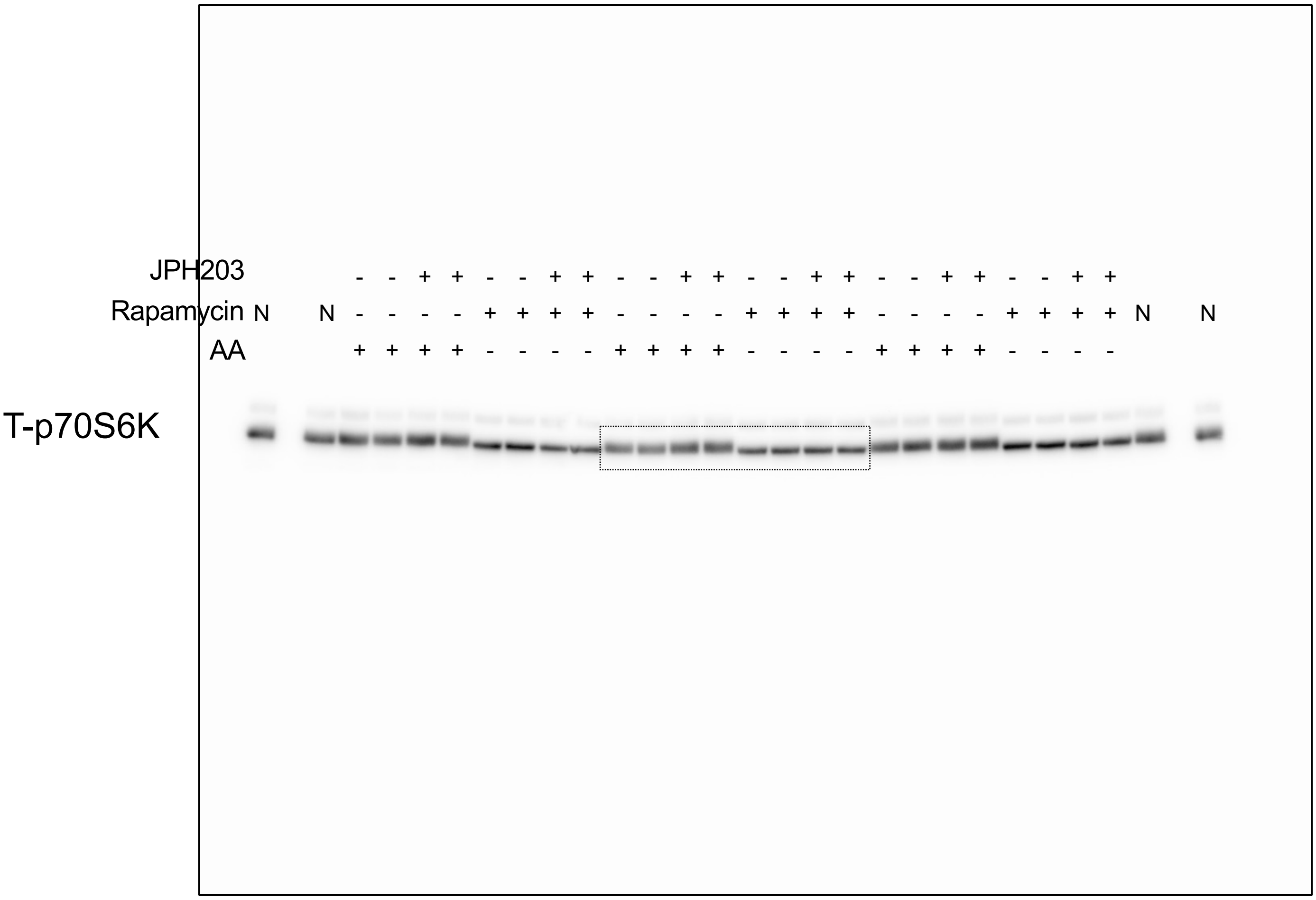

Bright field

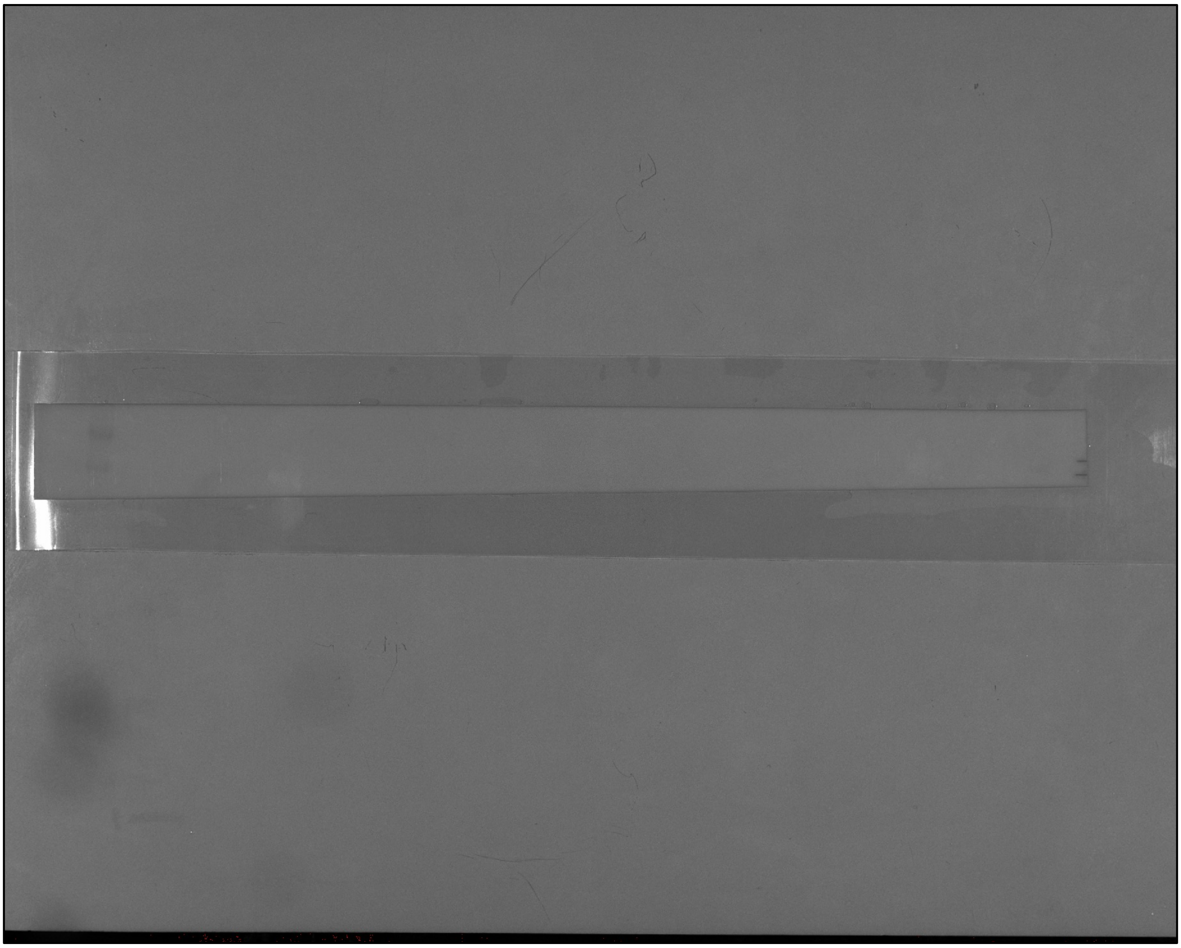

Band images in Fig. 7 (continued).

T-rpS6

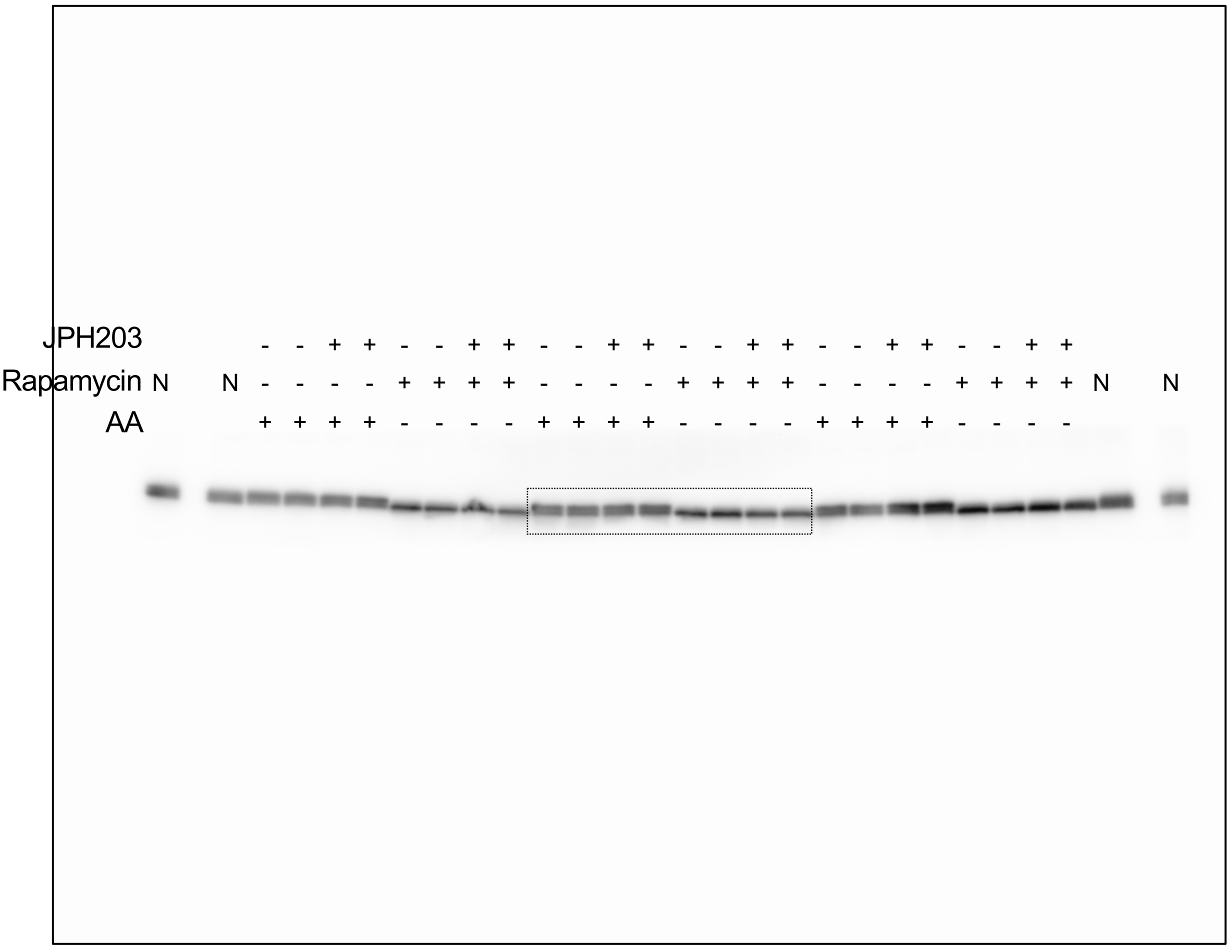

Bright field

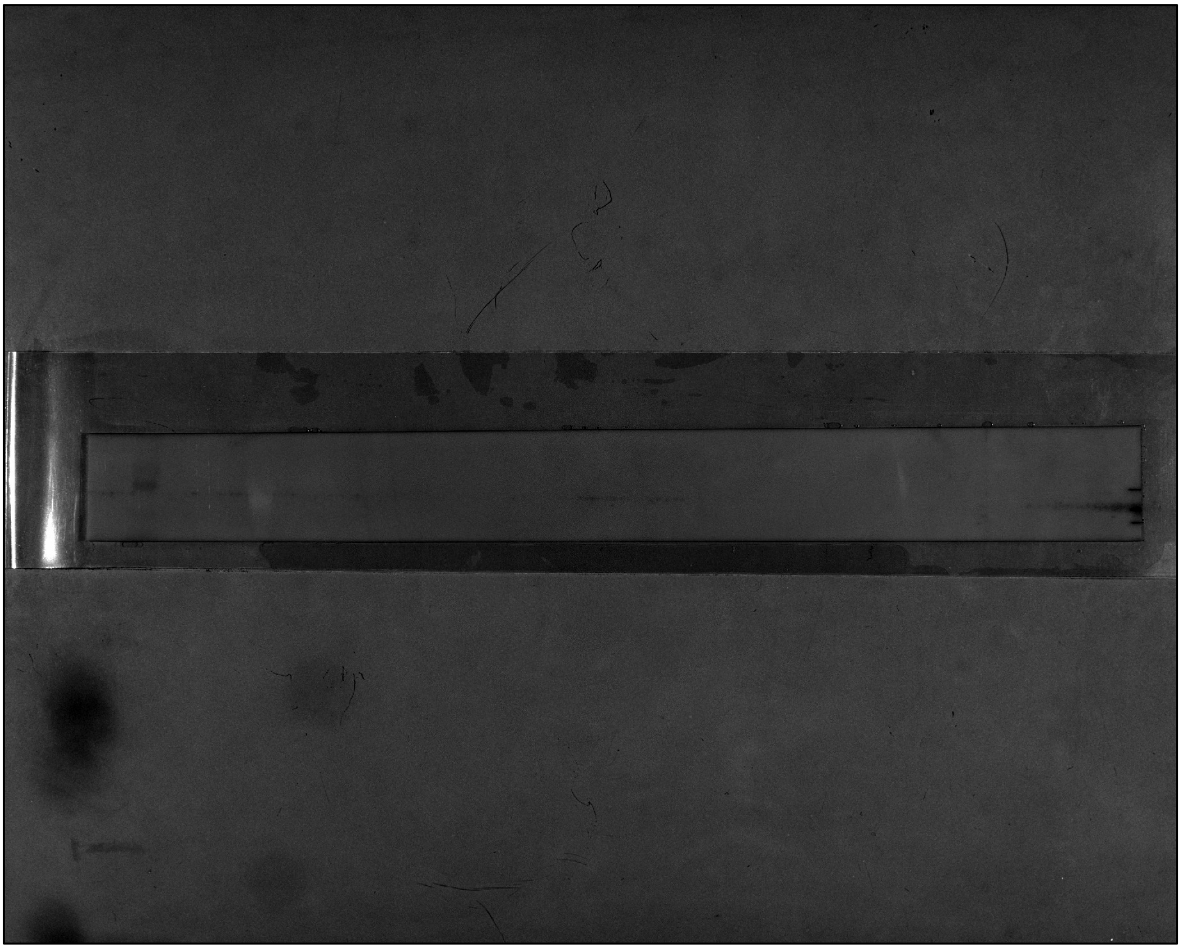

Band images in Fig. 7 (continued).

T-4EBP1

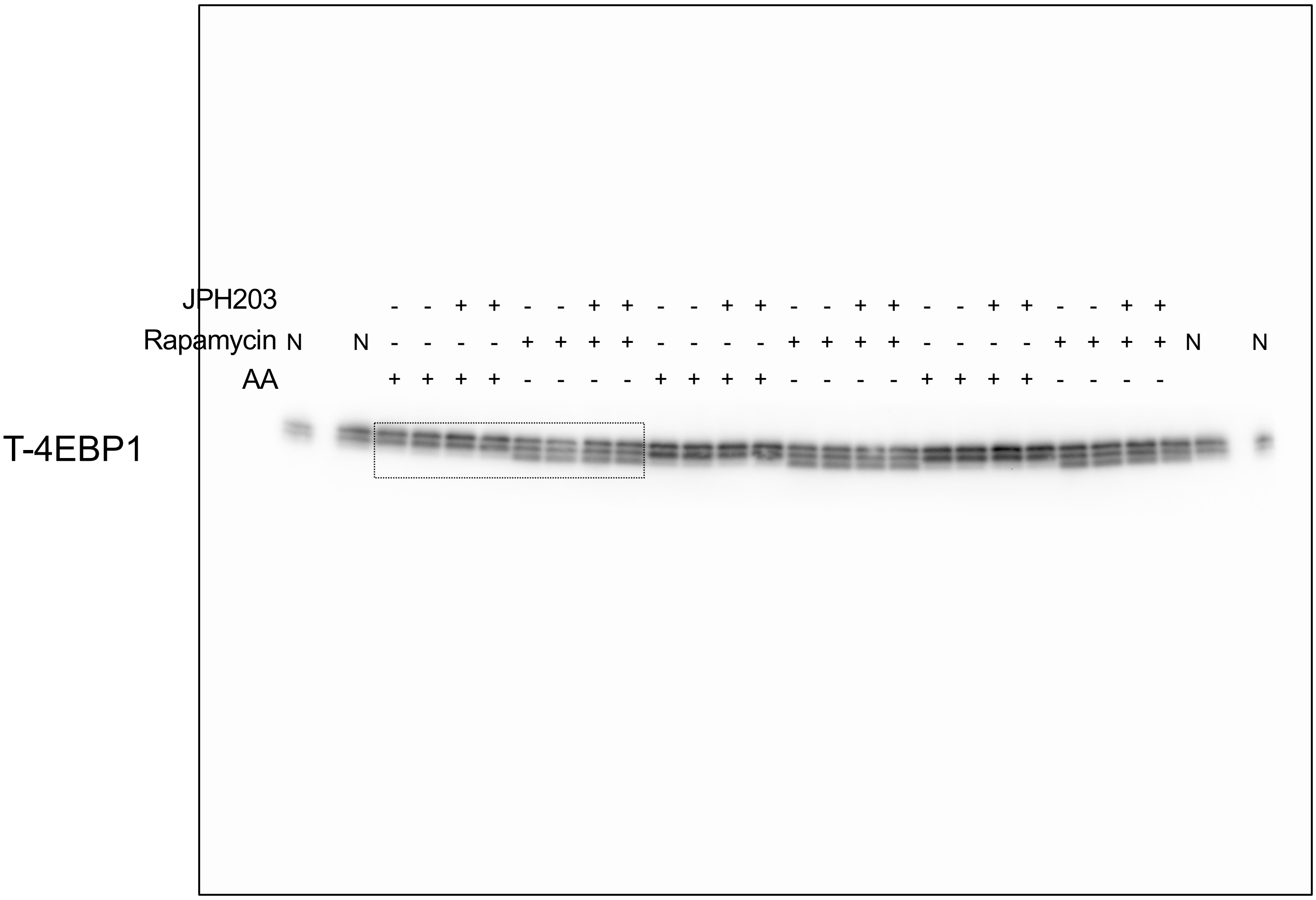

Bright field

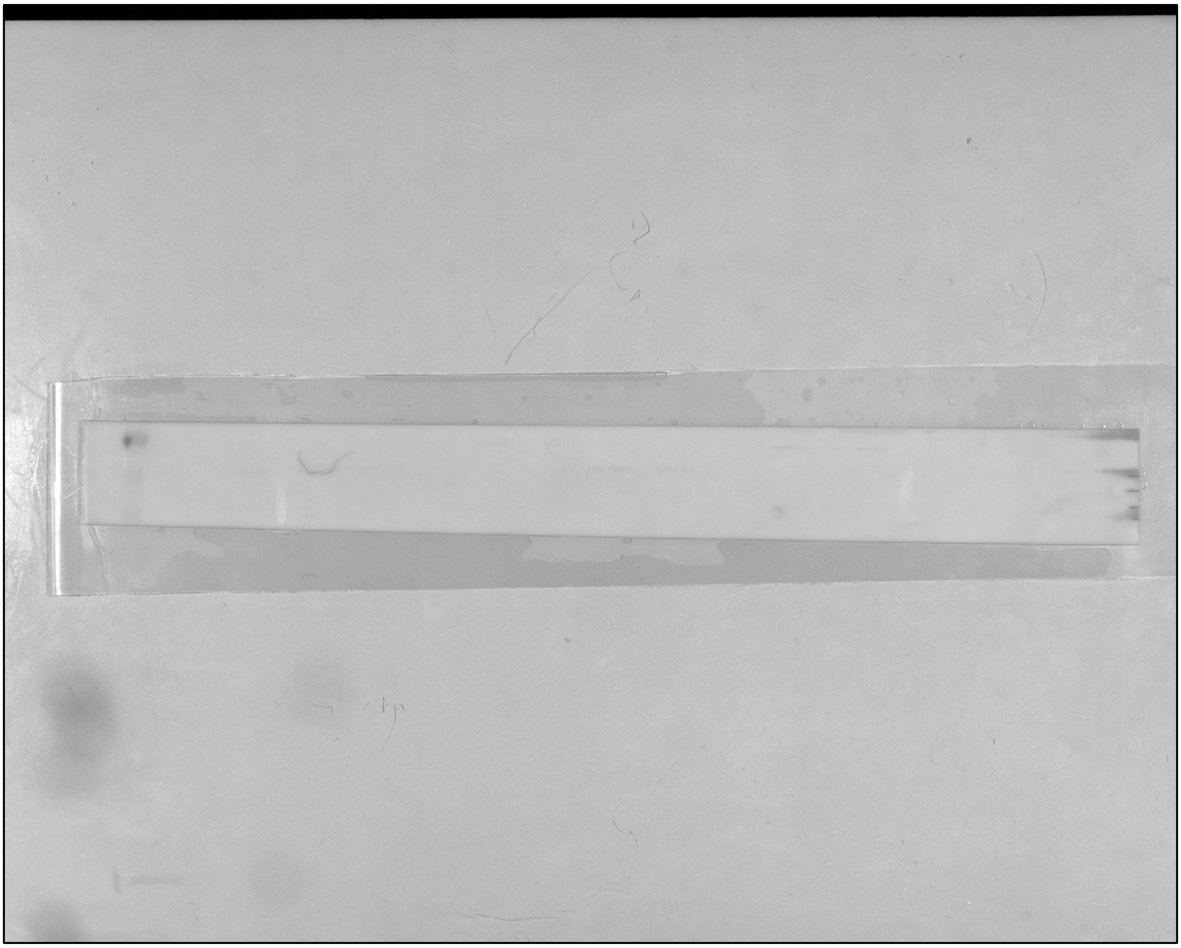

Band images in Fig. 7 (continued).

CBB

For P-p70S6K, P-rpS6, and P-4EBP1.

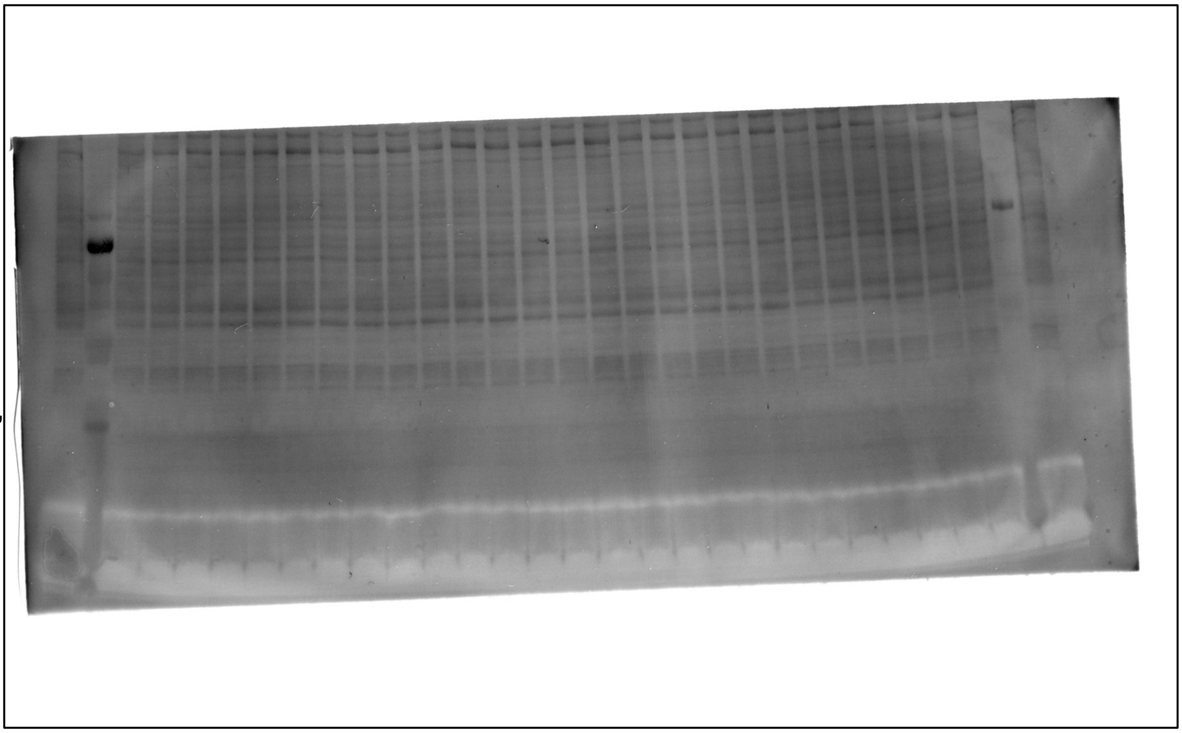

CBB

For T-p70S6K, T-rpS6, and T-4EBP1.

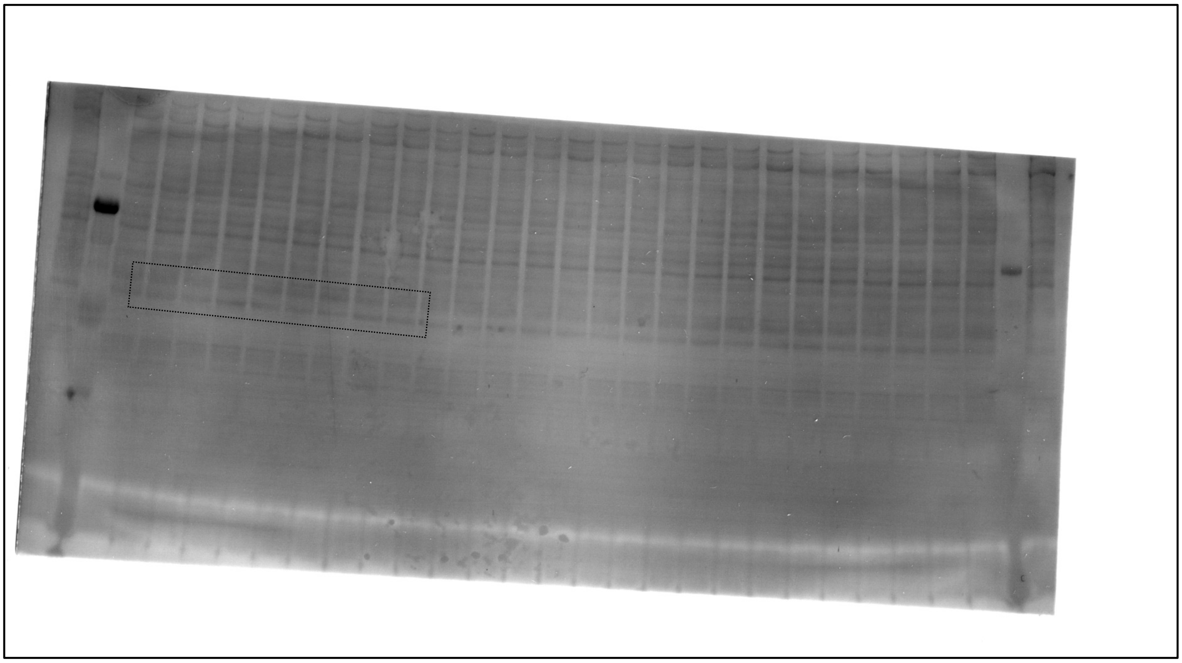

Band images in Fig. 7 (continued).

Puromycin

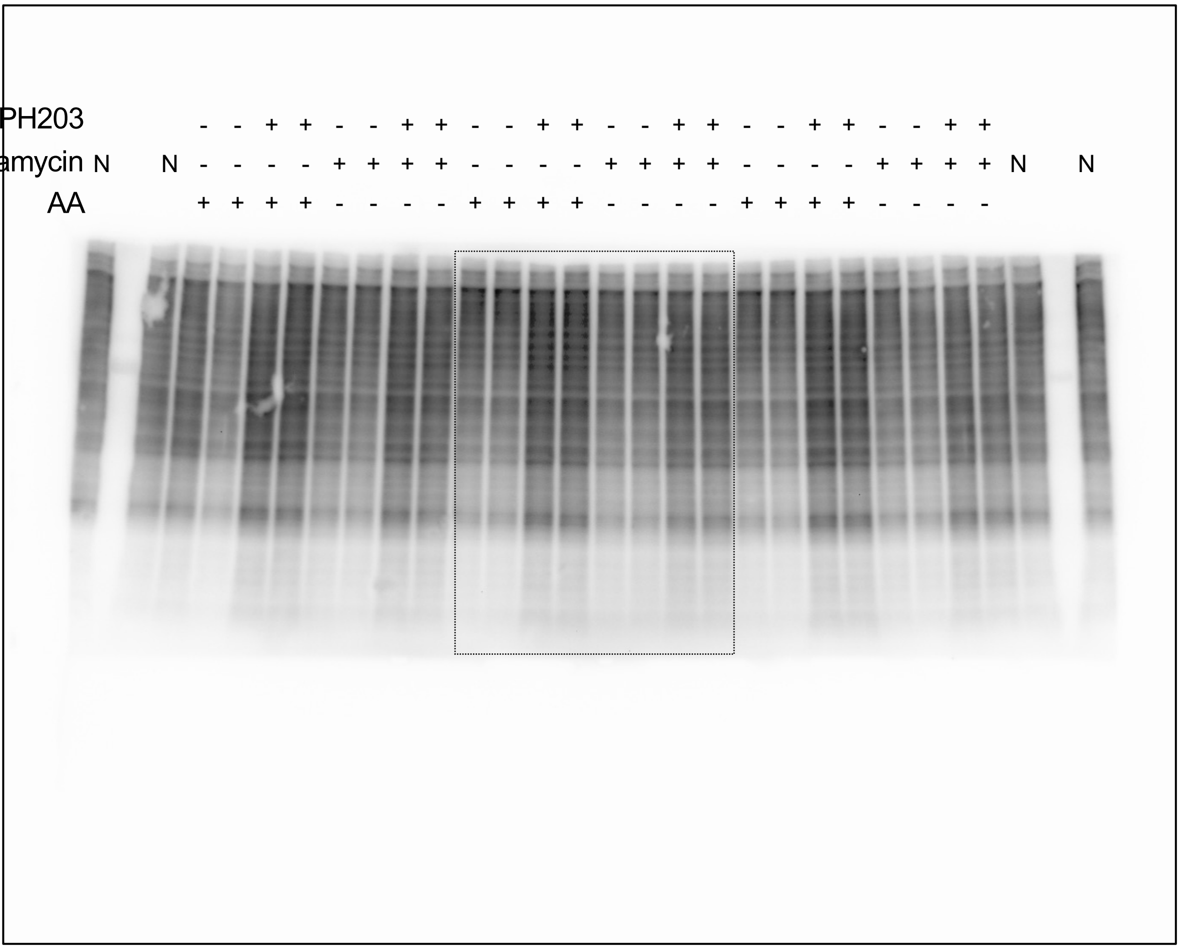

Bright field

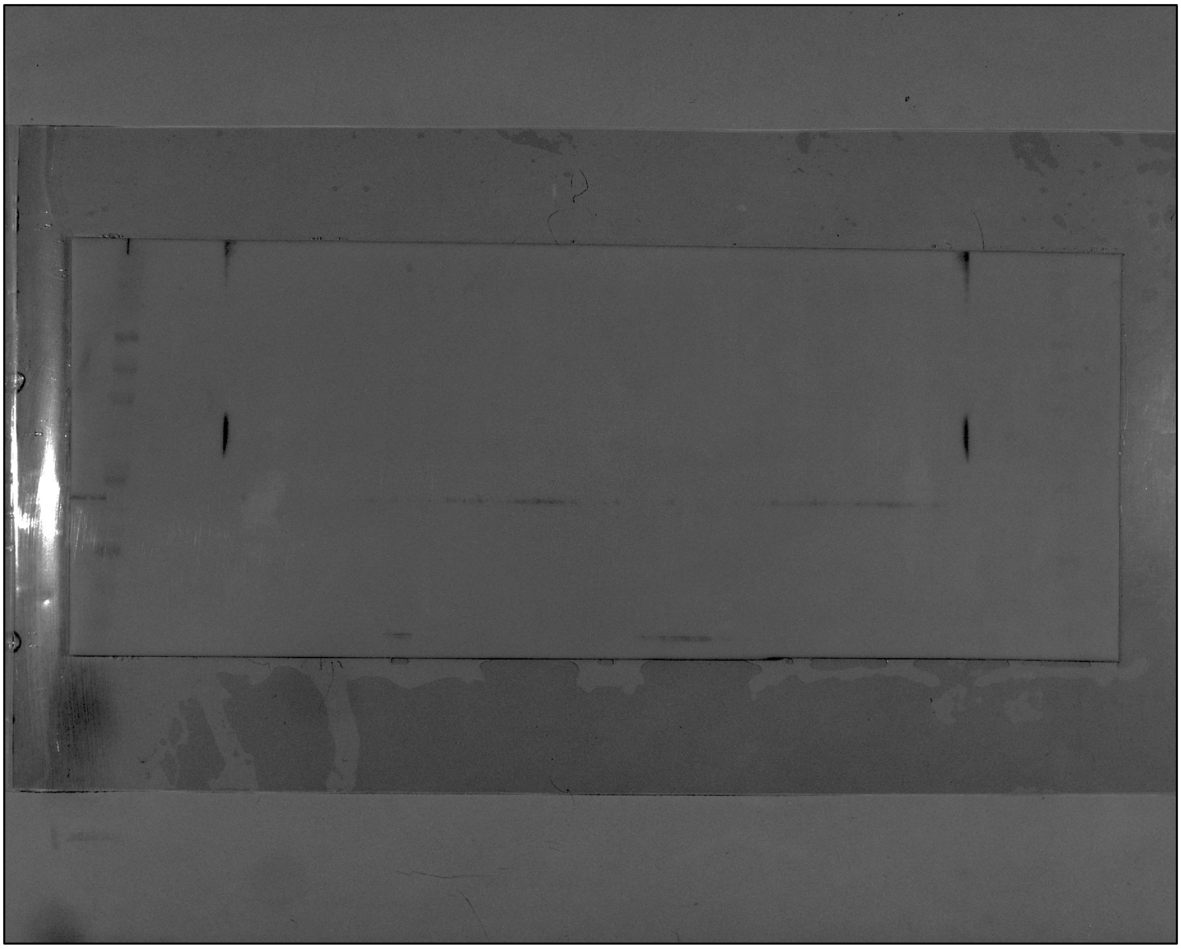

Band images in Fig. 7 (continued).

CBB

For
puromycin

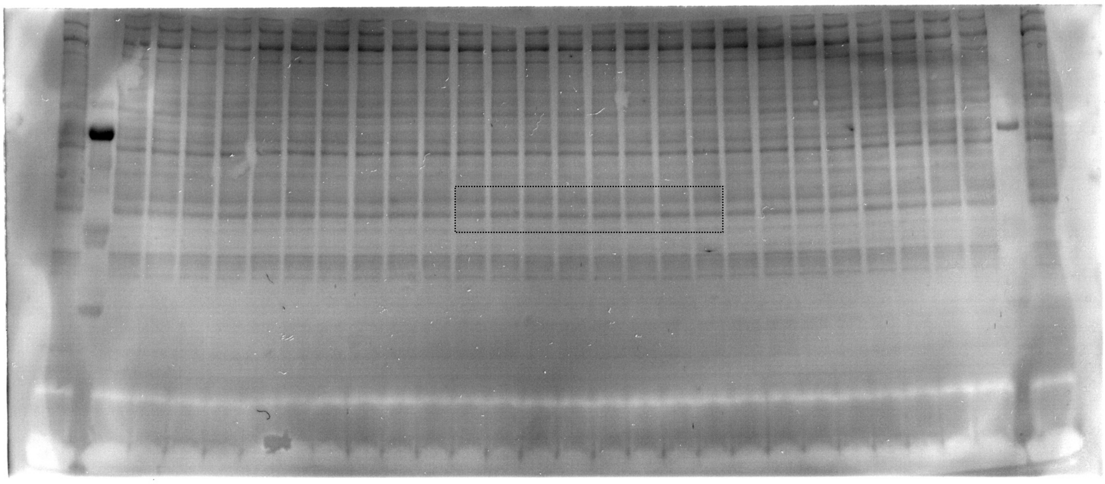

Band images in Fig. 8.

P-Akt

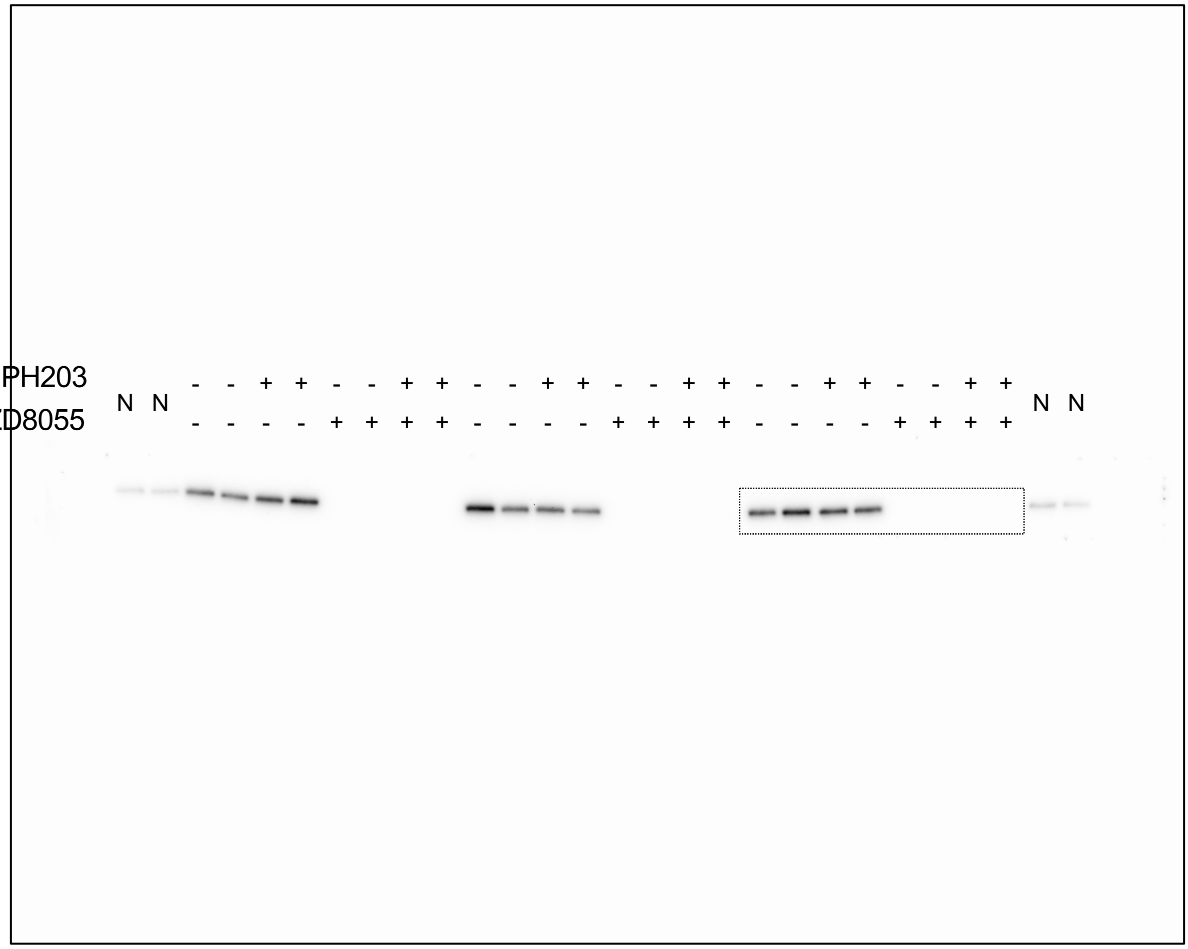

Bright field

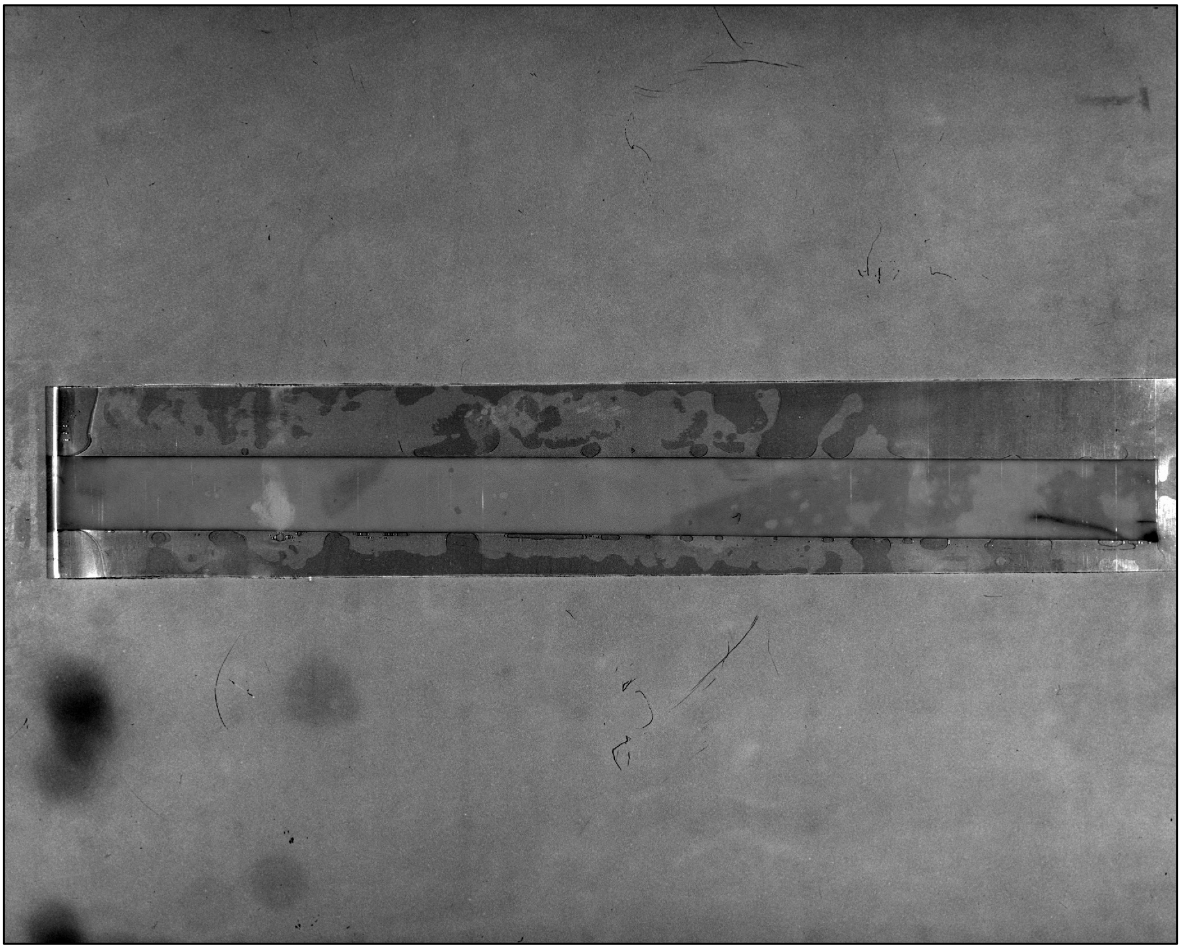

Band images in Fig. 8 (continued).

T-Akt

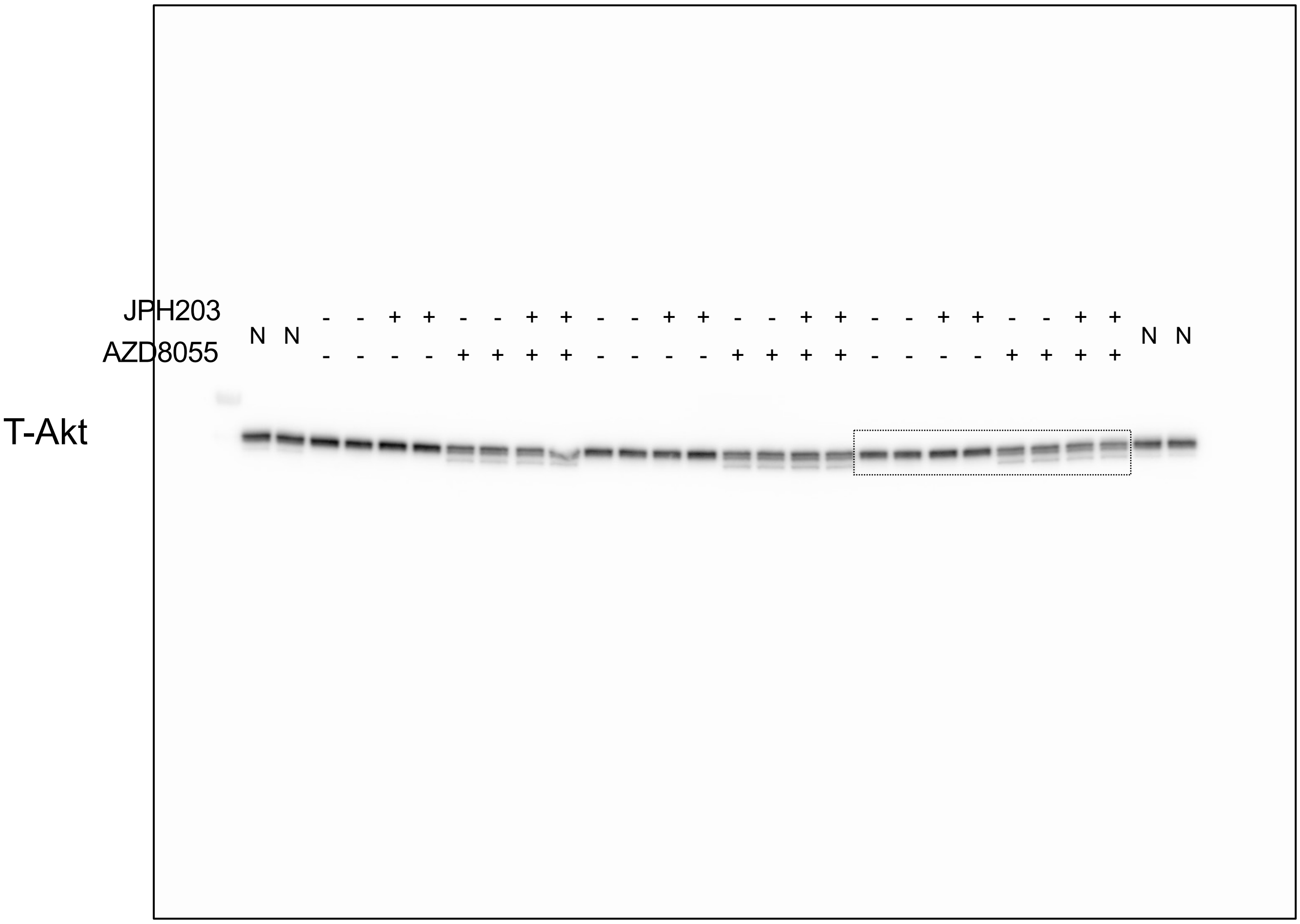

Bright field

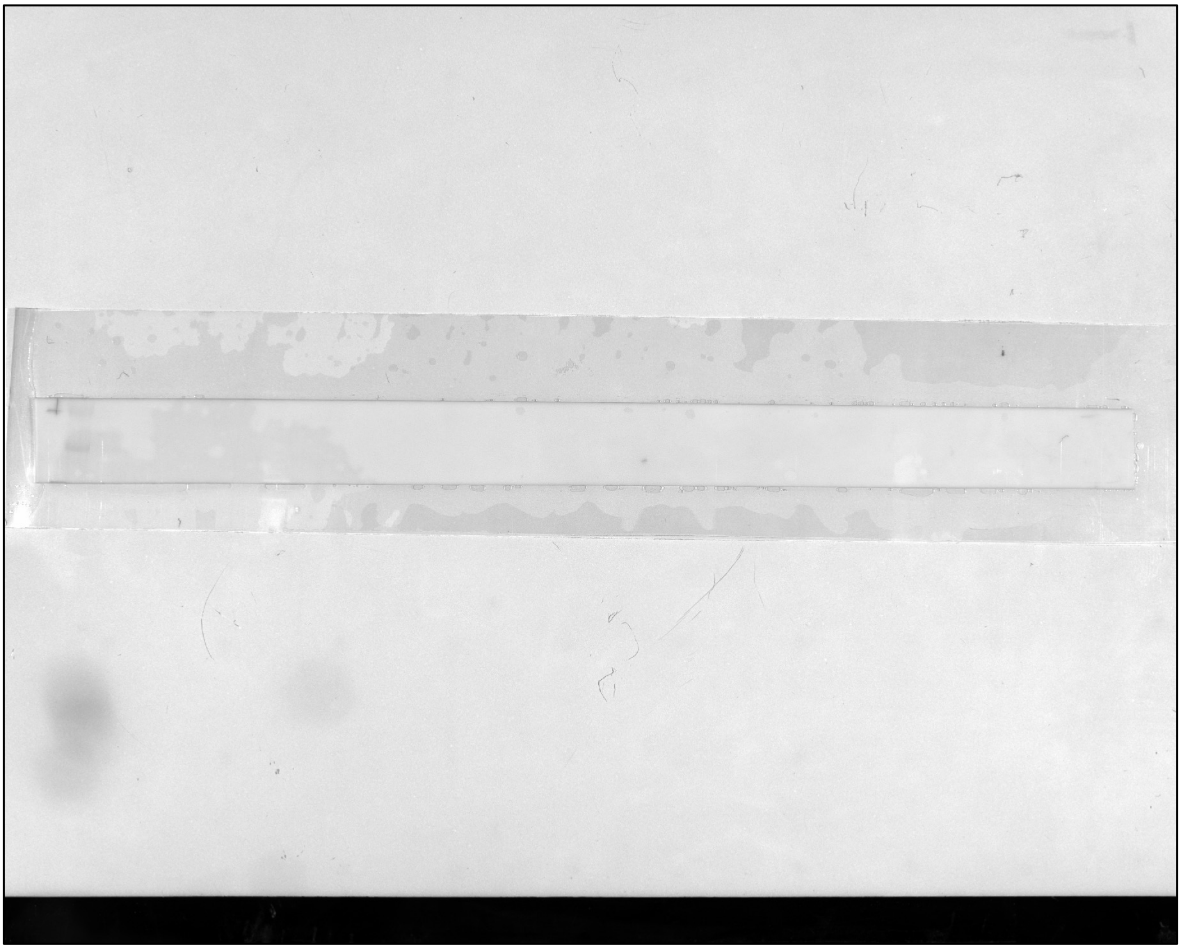

Band images in Fig. 8 (continued).

CBB

For P-Akt

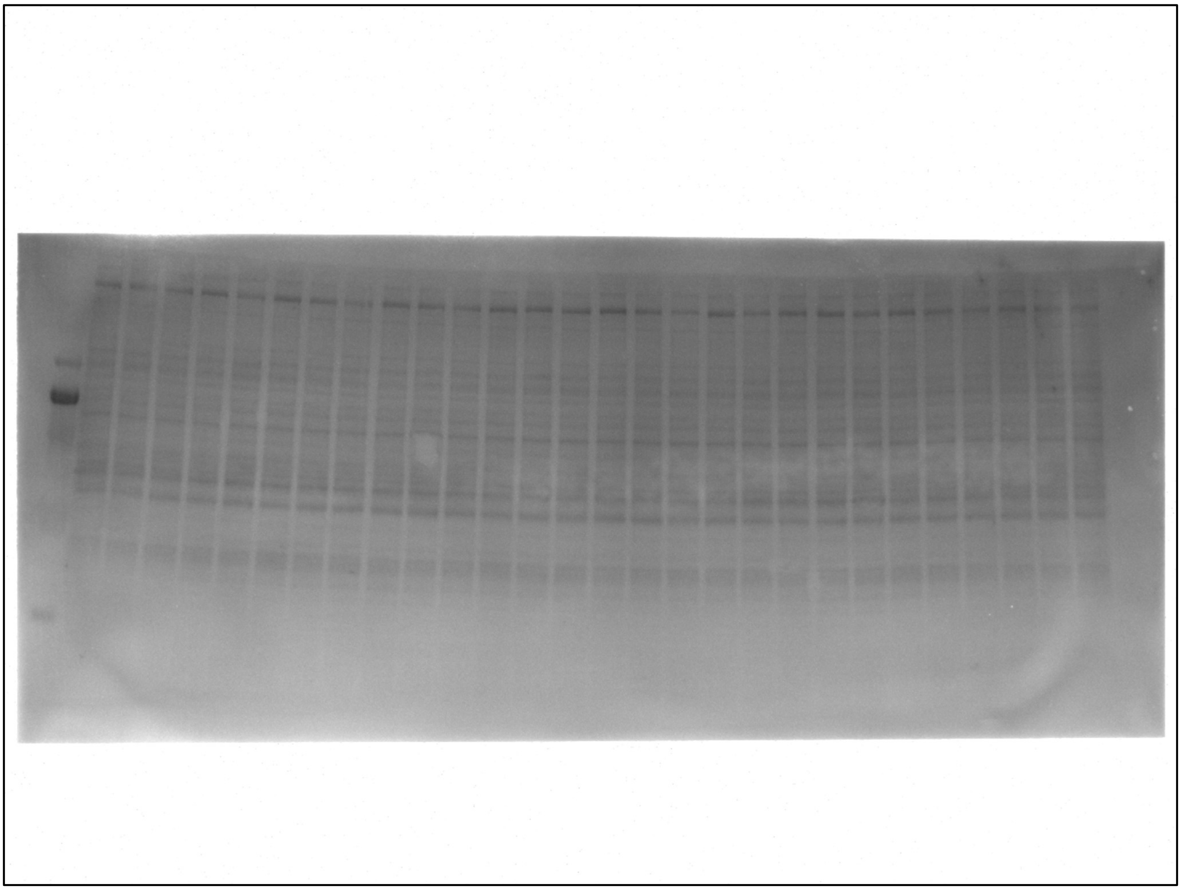

CBB

For T-Akt

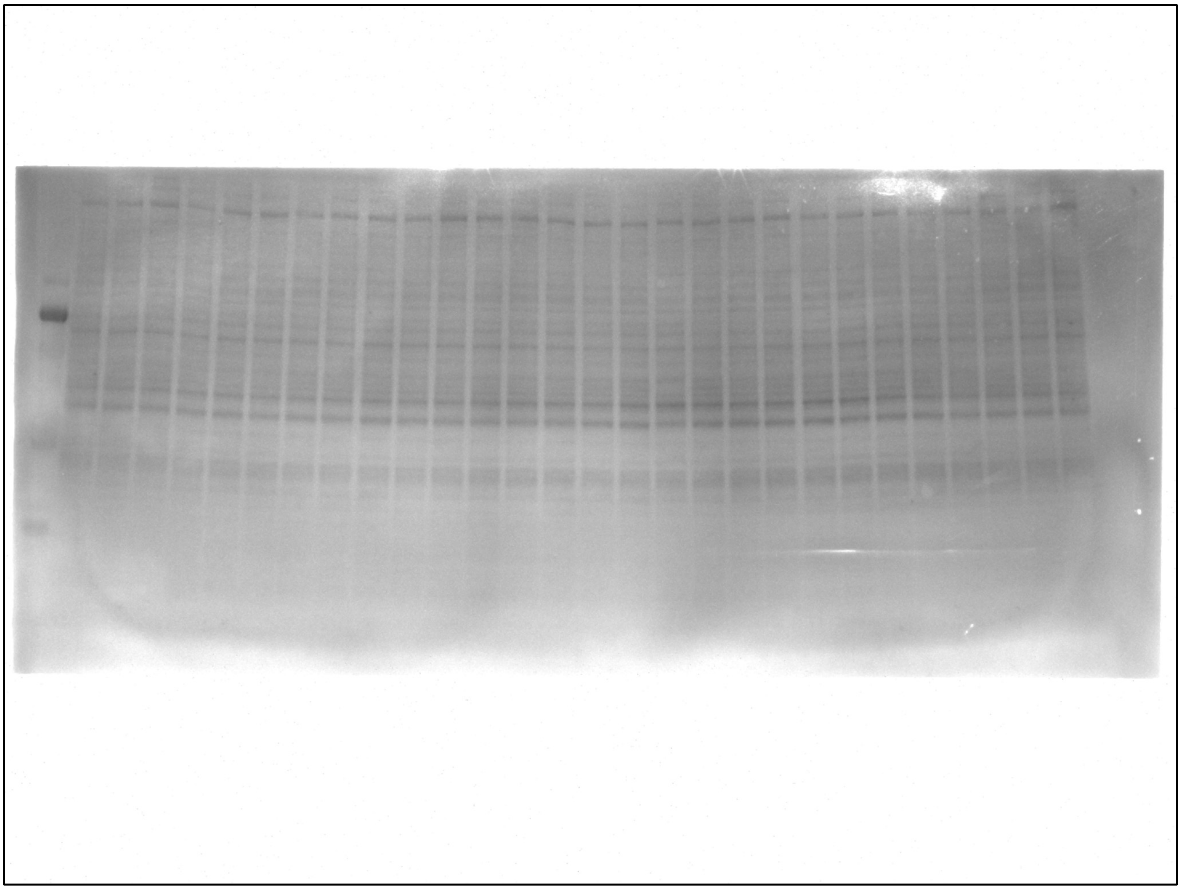

Band images in Fig. 8 (continued).

P-p70S6K

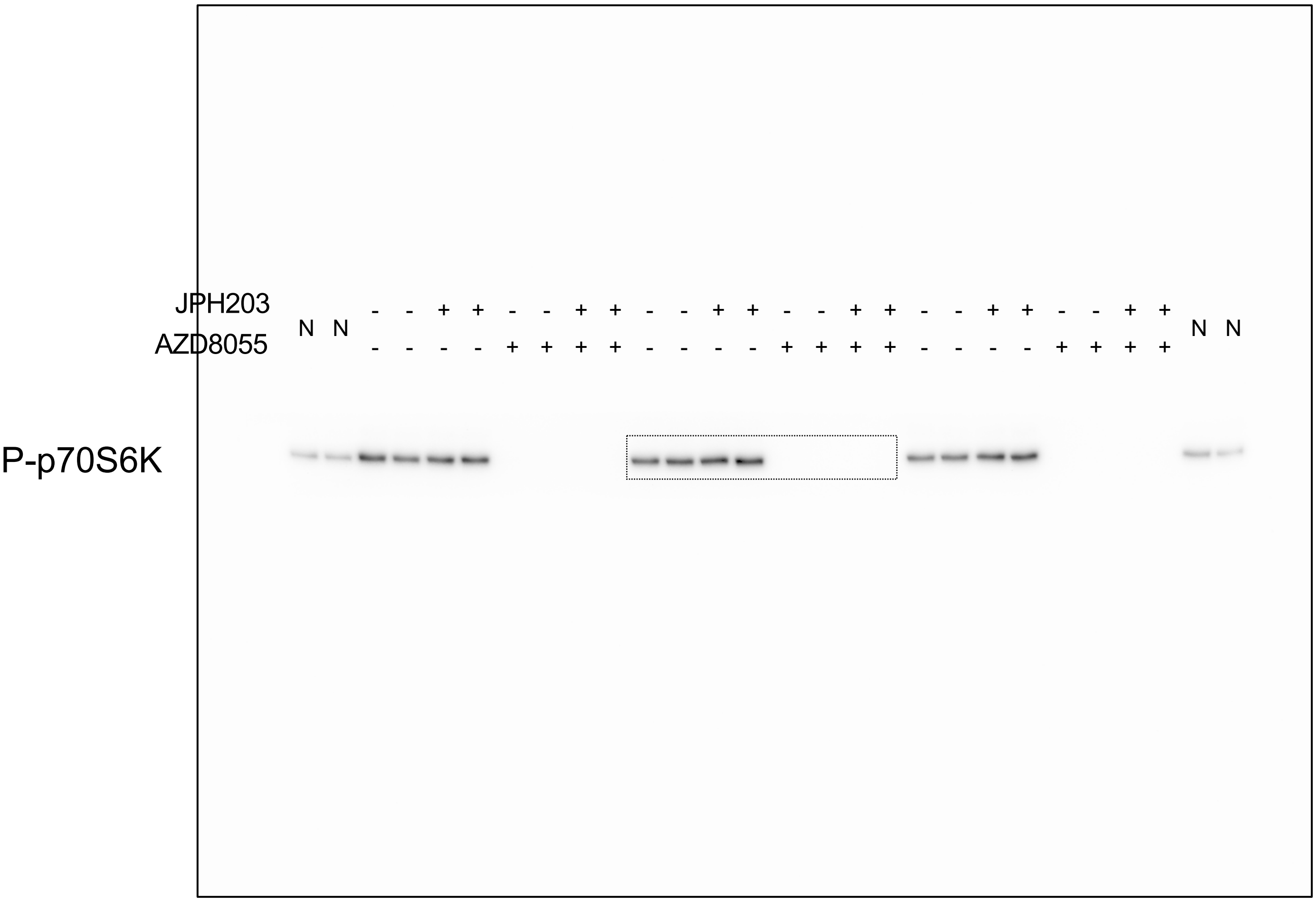

Bright field

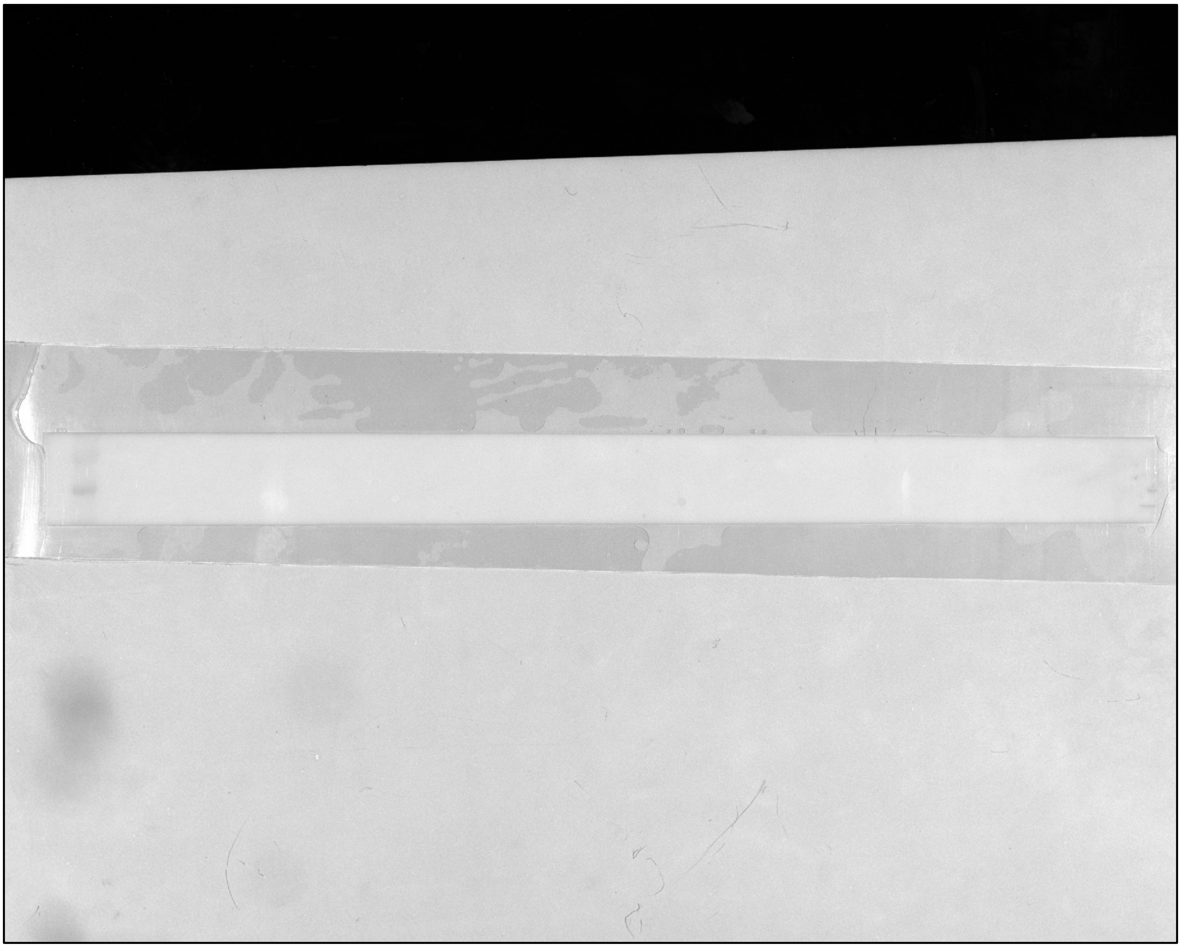

Band images in Fig. 8 (continued).

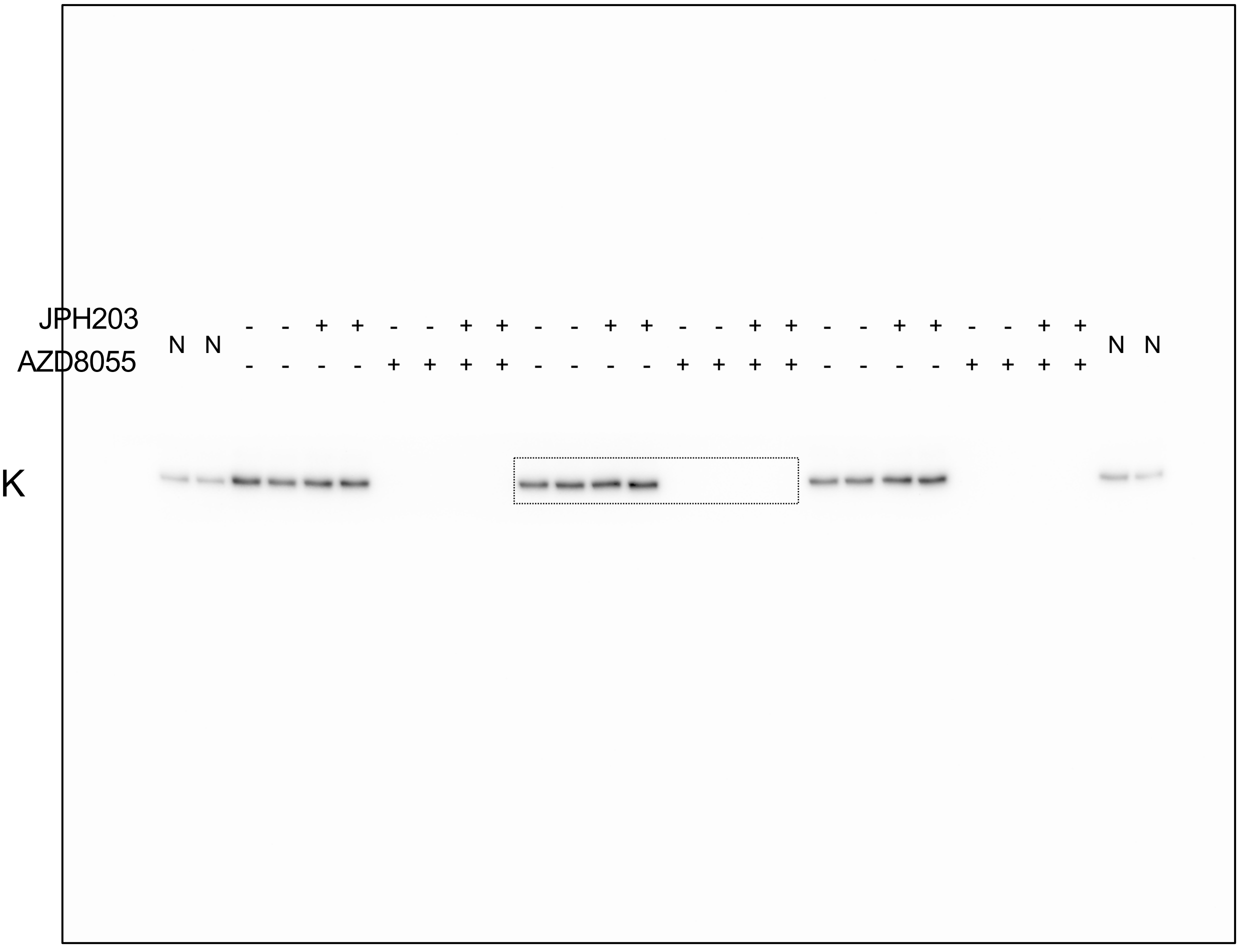

Bright field

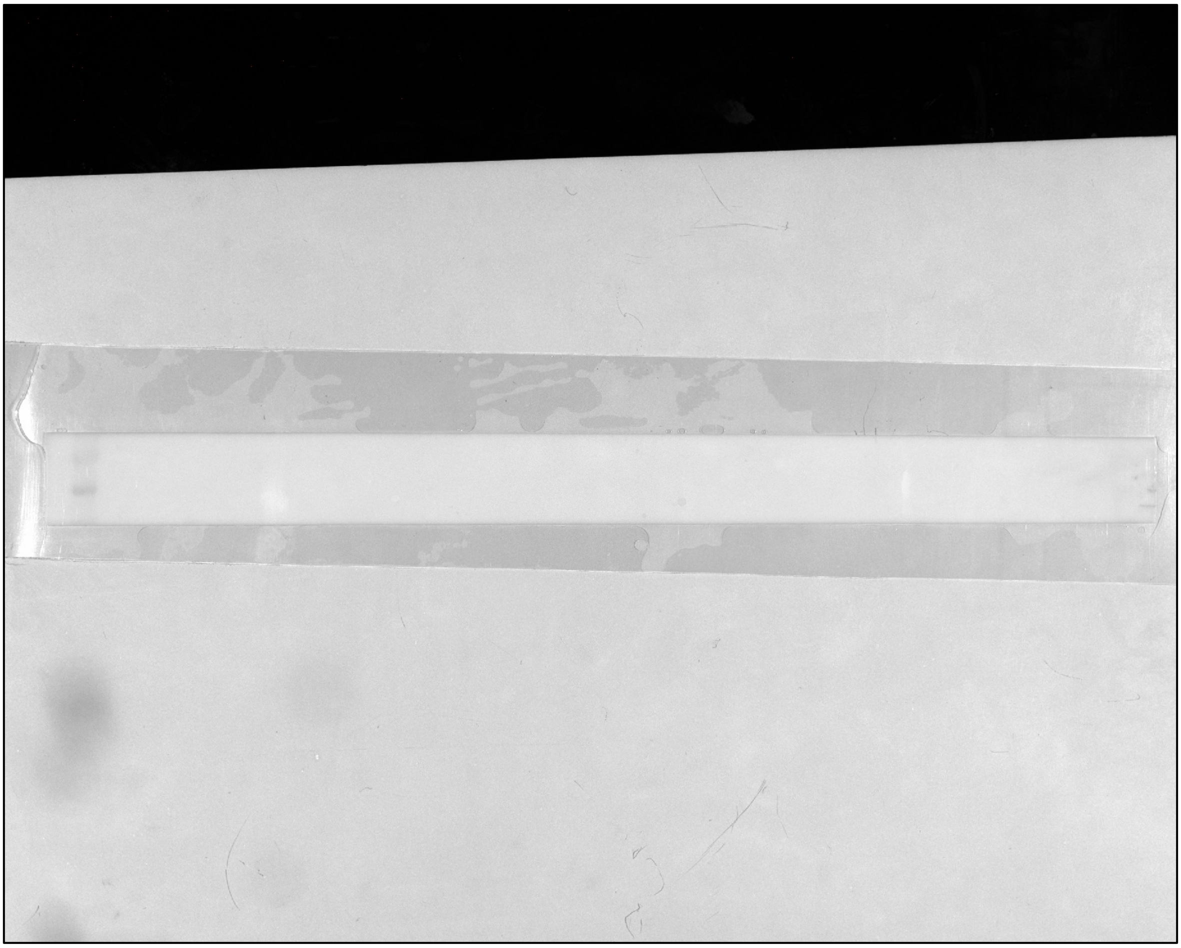

Band images in Fig. 8 (continued).

P-rpS6

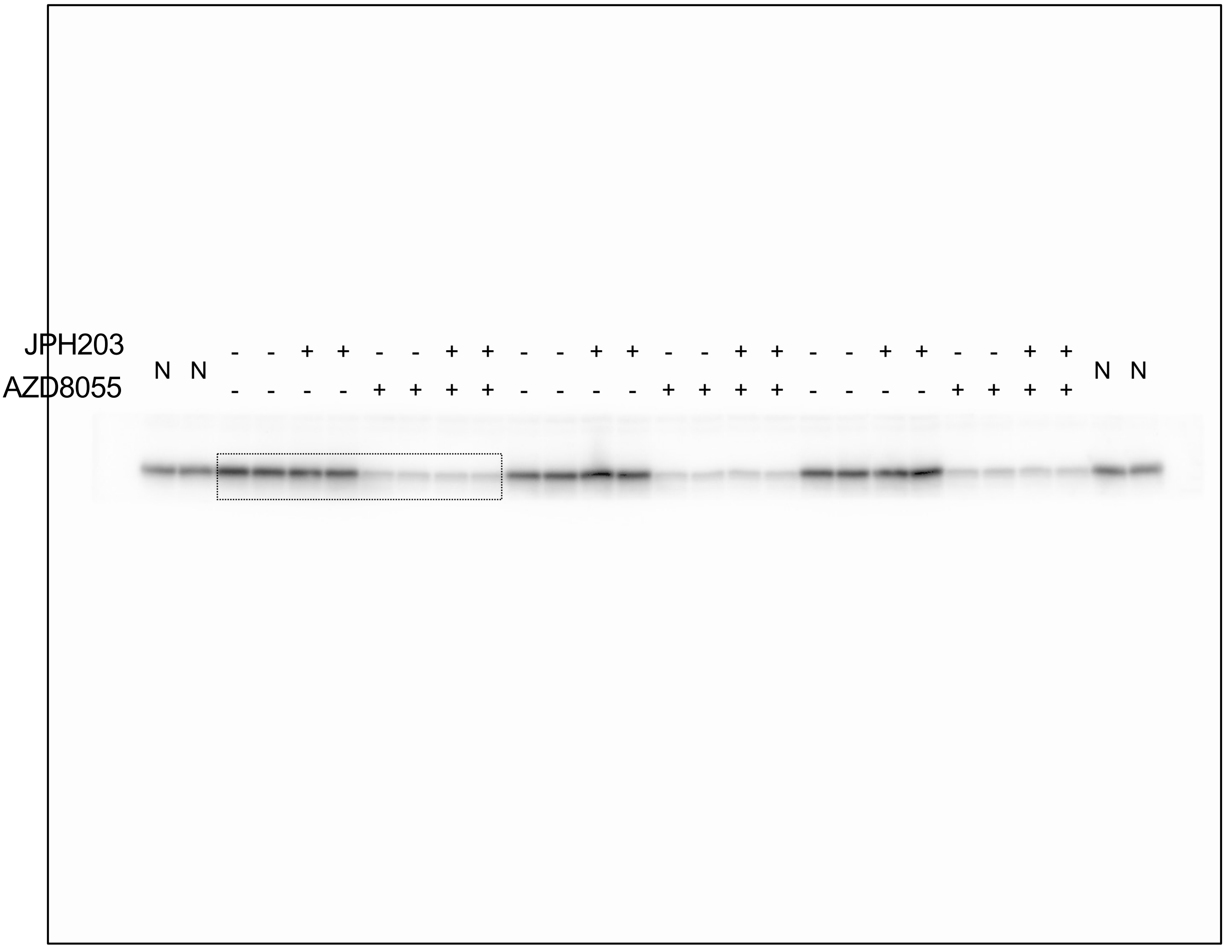

Bright field

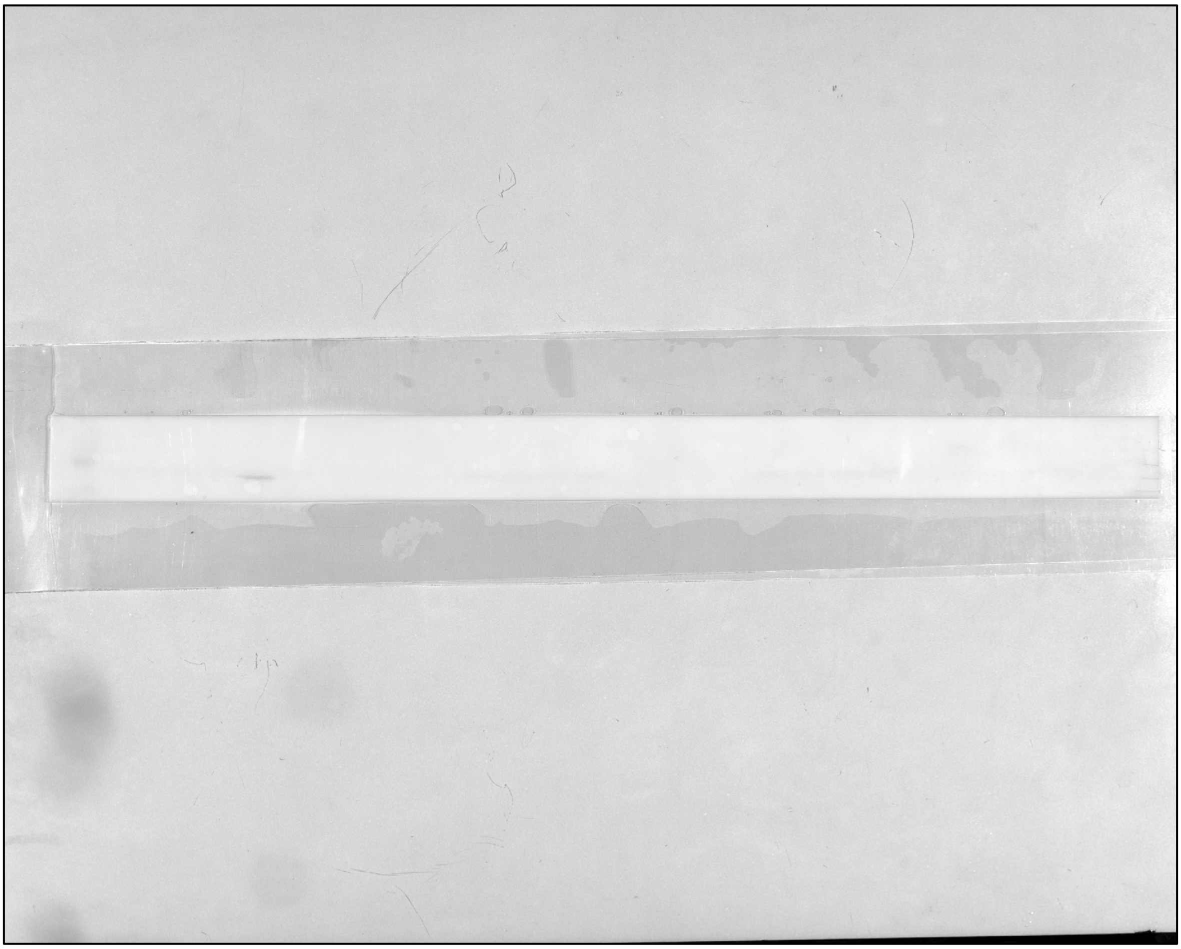

Band images in Fig. 8 (continued).

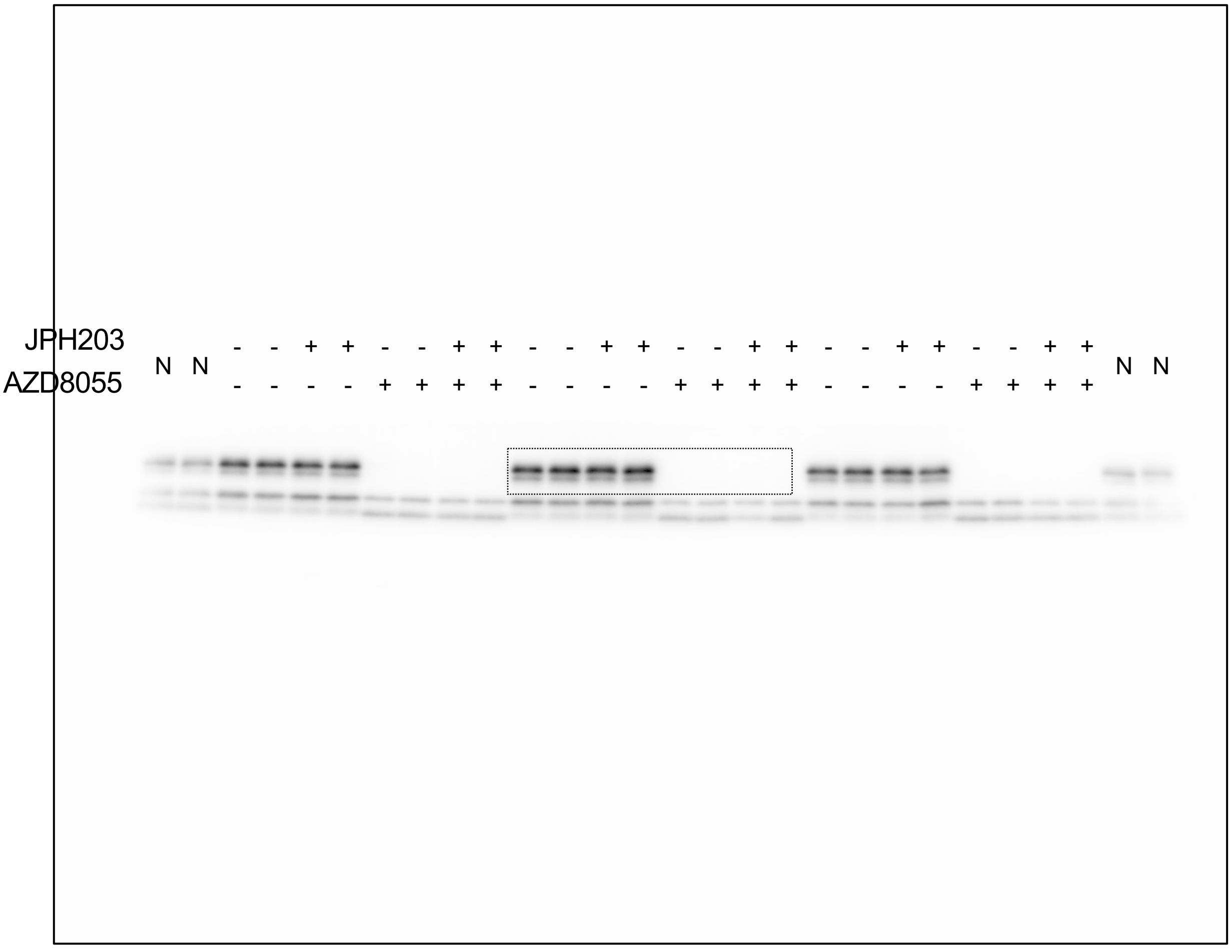

P-4EBP1

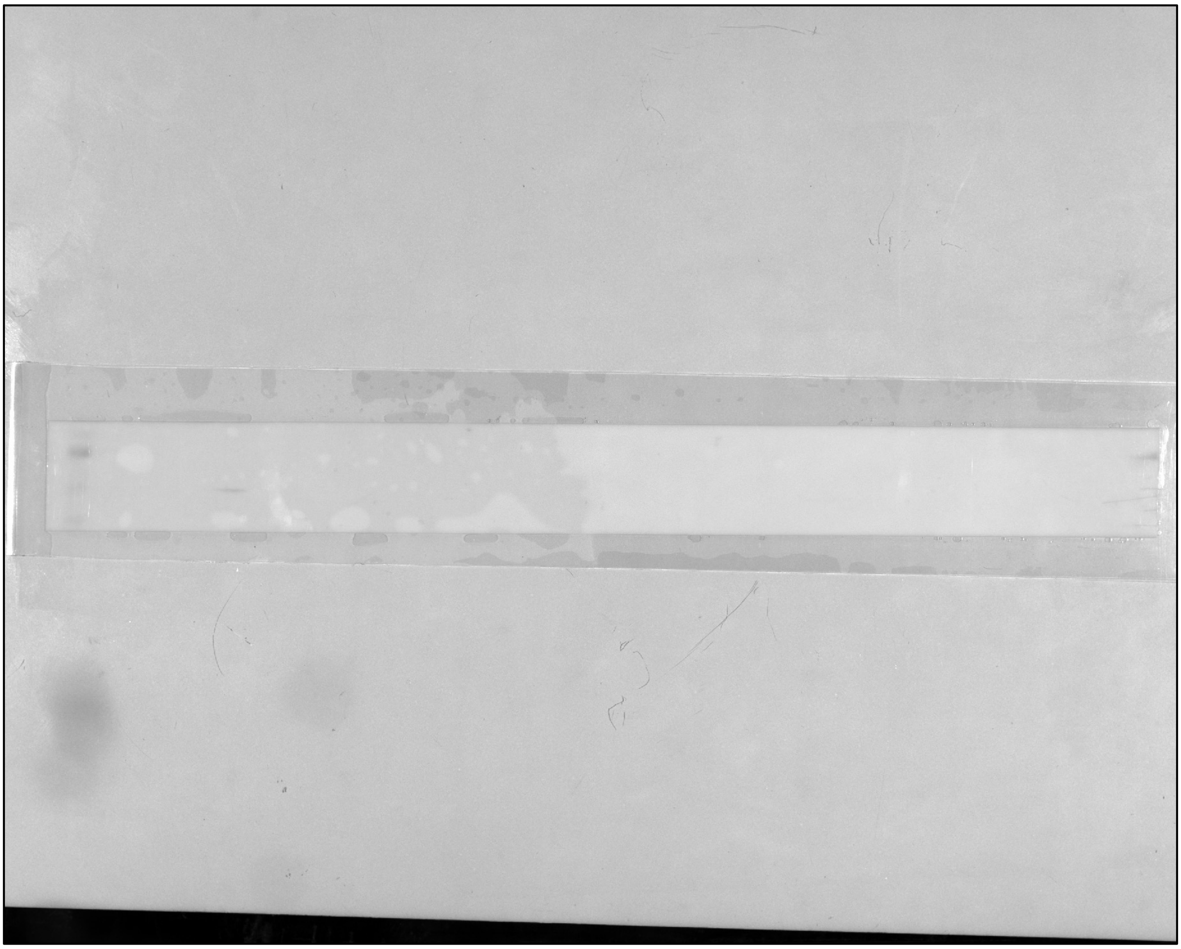

Bright field

Band images in Fig. 8 (continued).

T-p70S6K

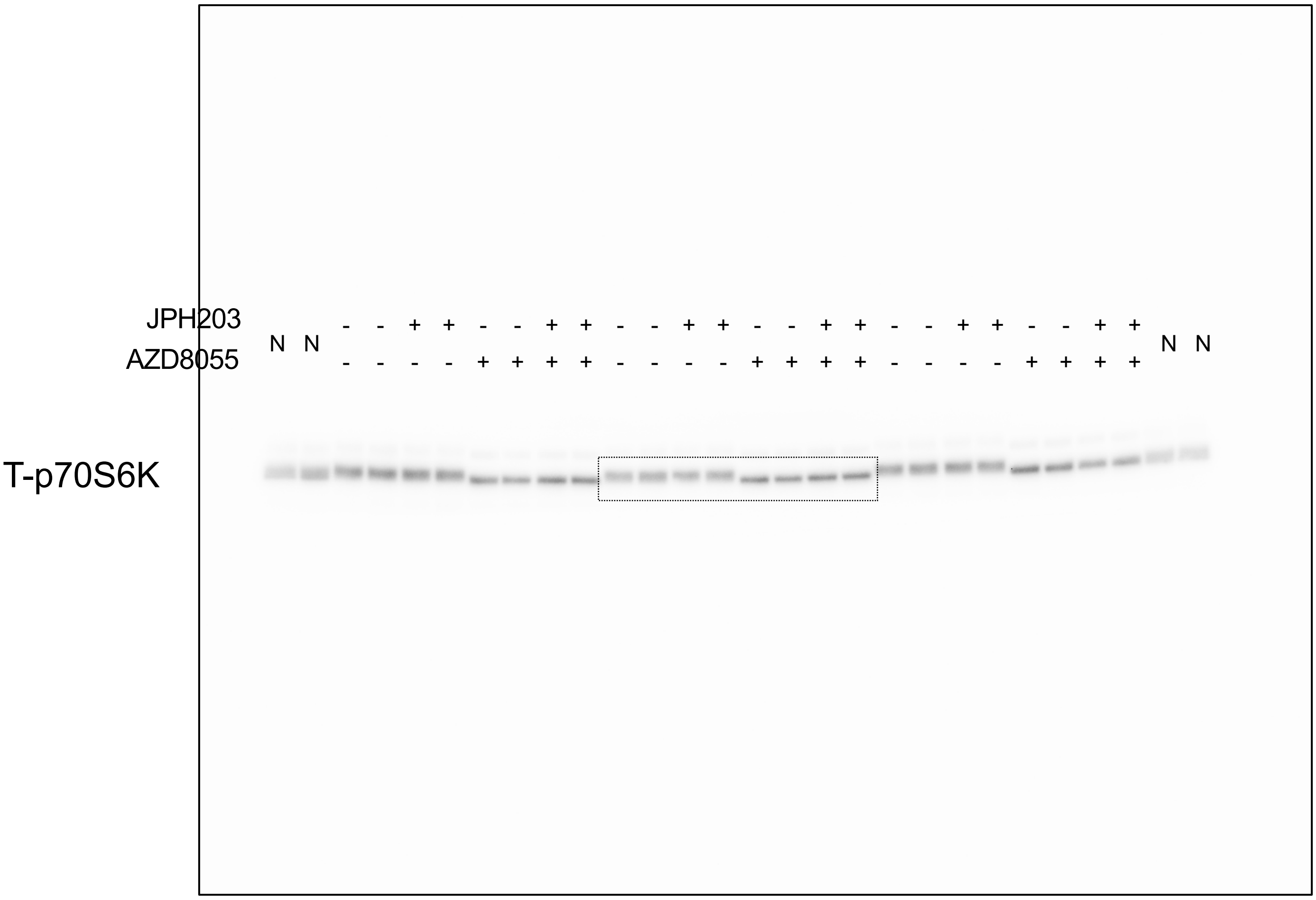

Bright field

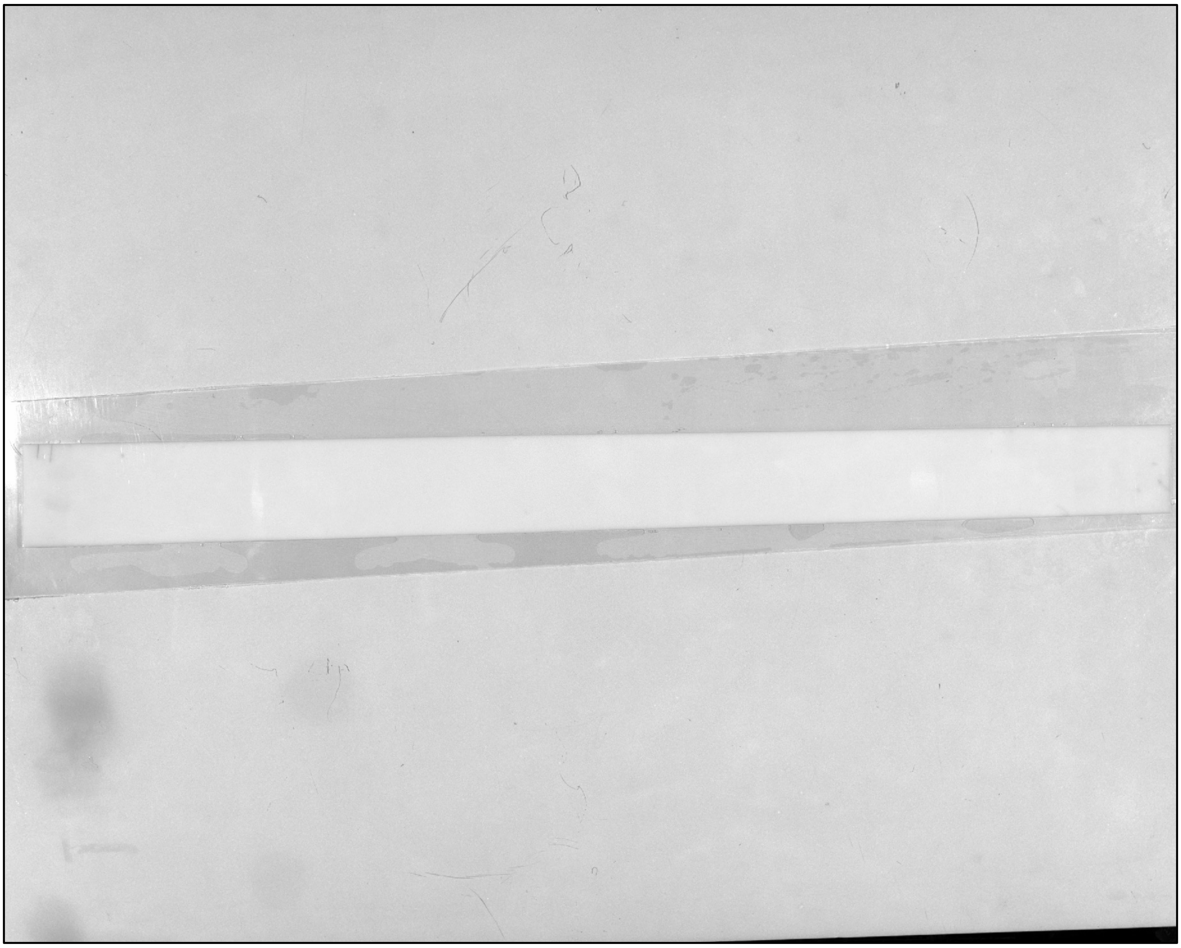

Band images in Fig. 8 (continued).

T-rpS6

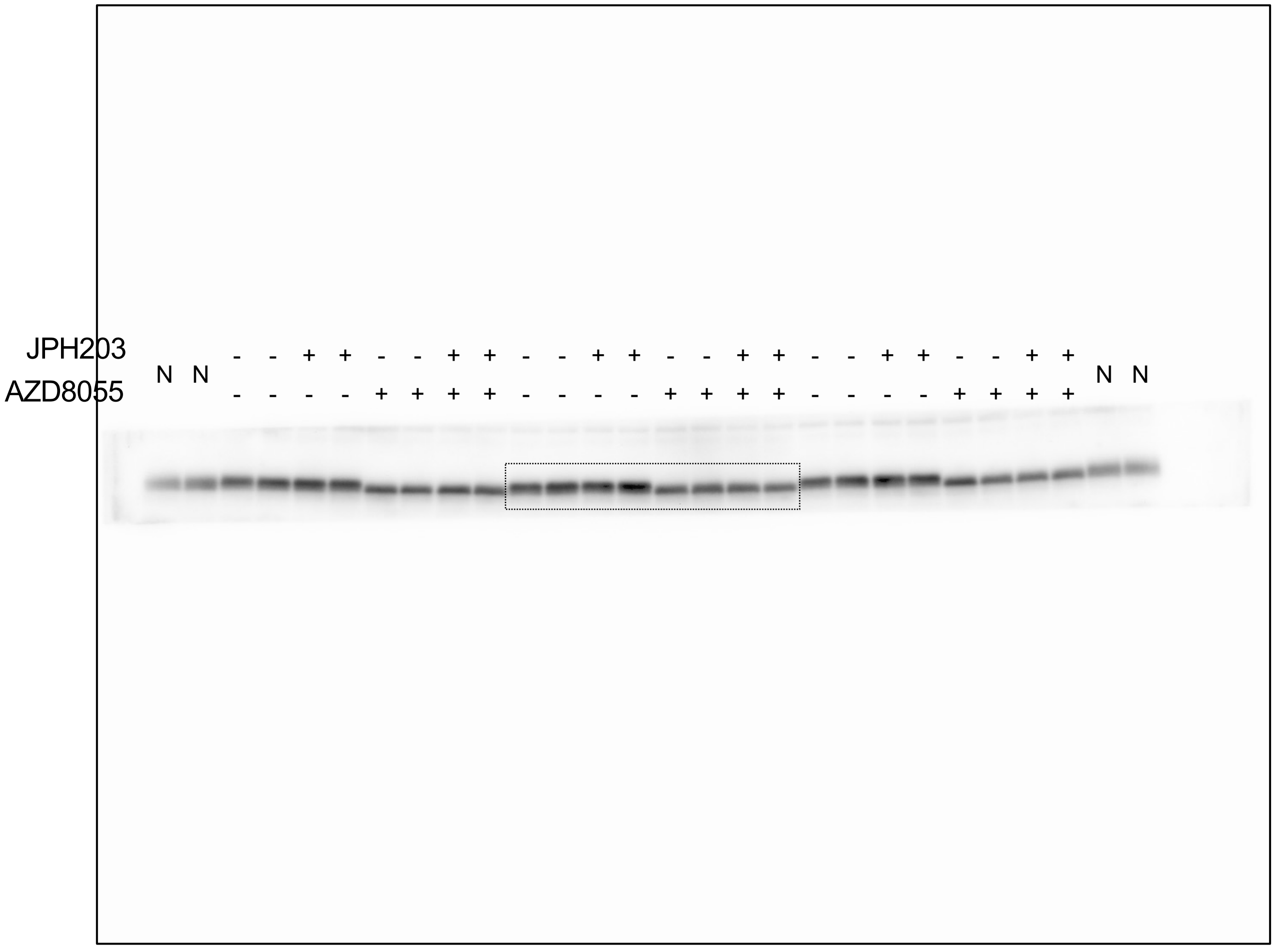

Bright field

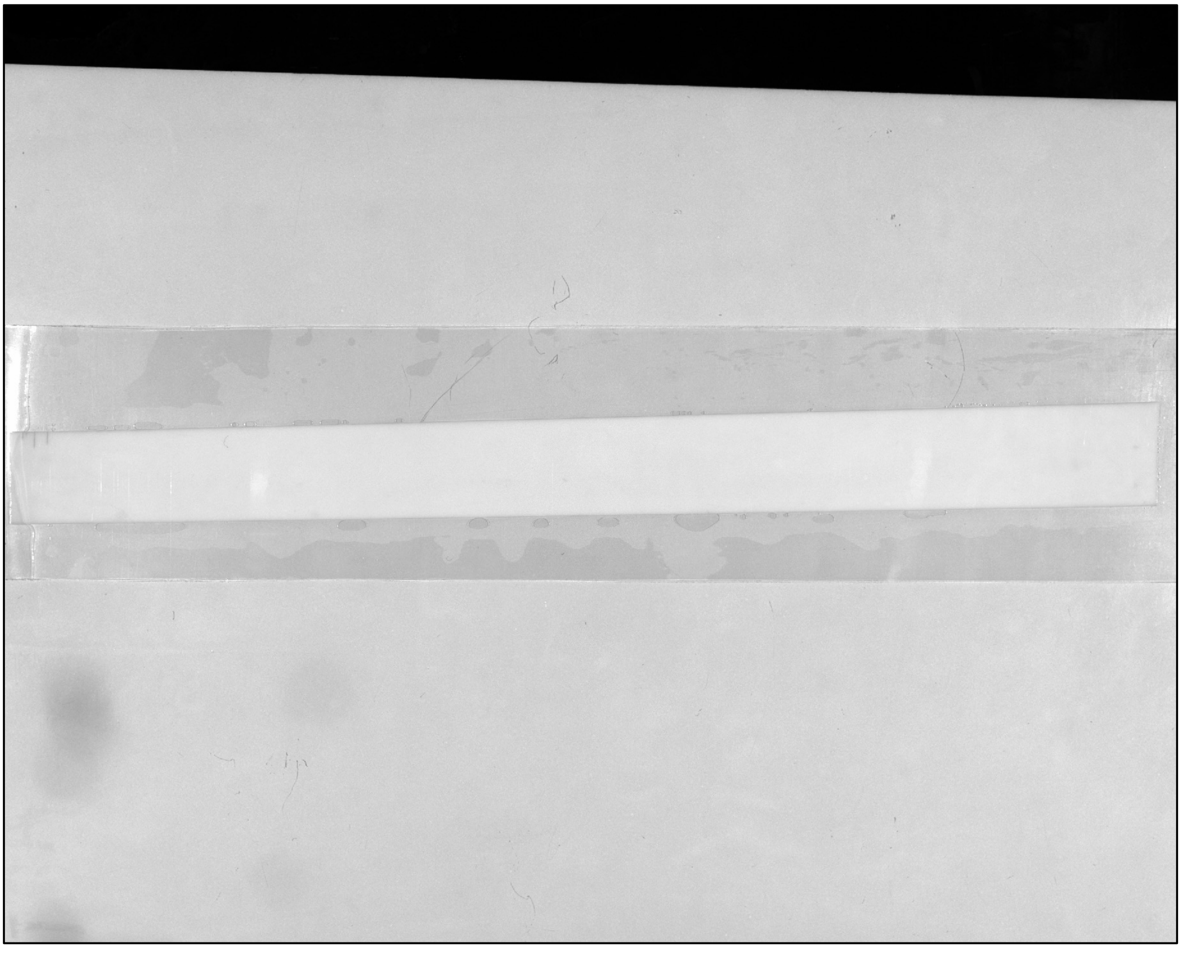

Band images in Fig. 8 (continued).

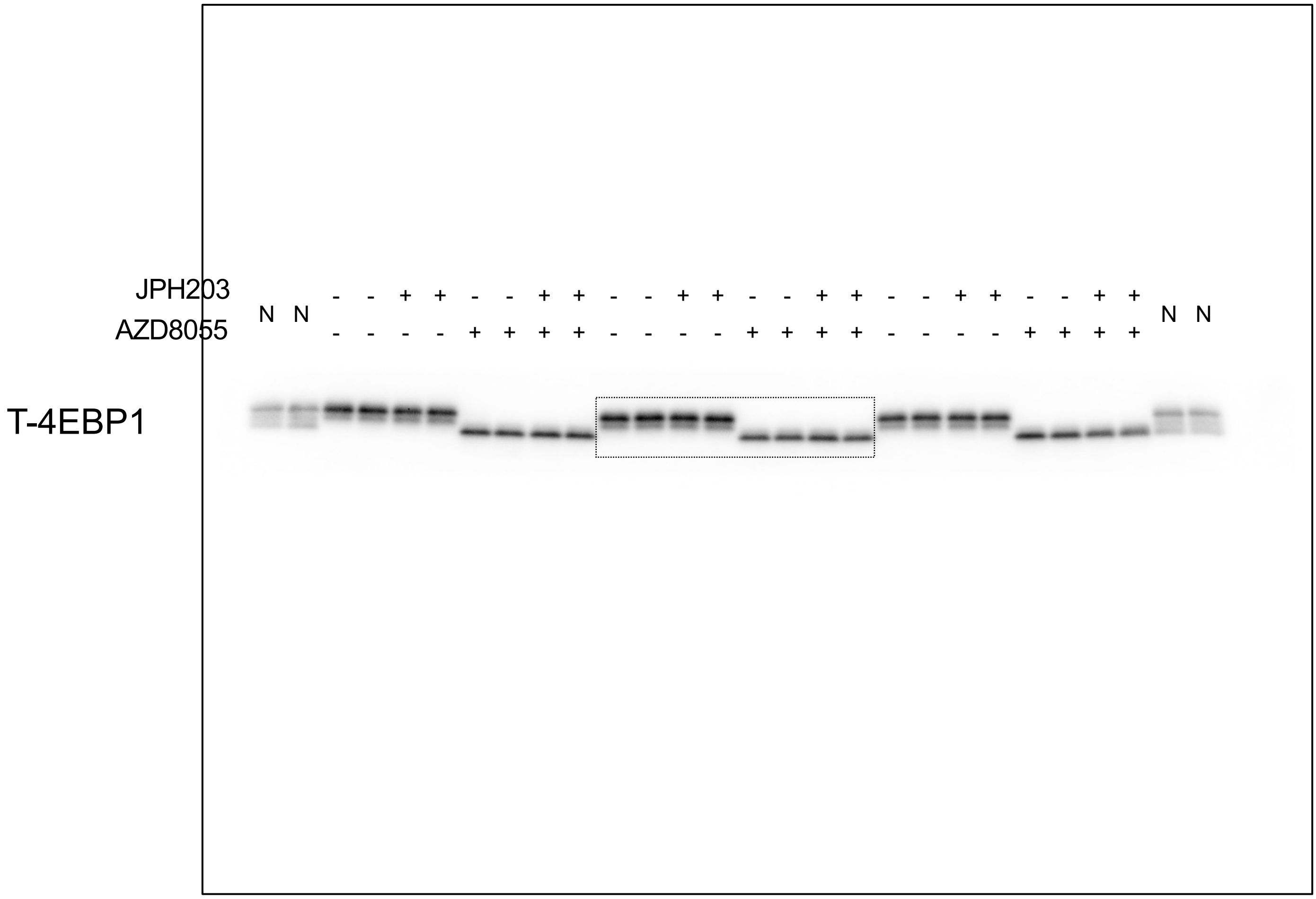

Bright field

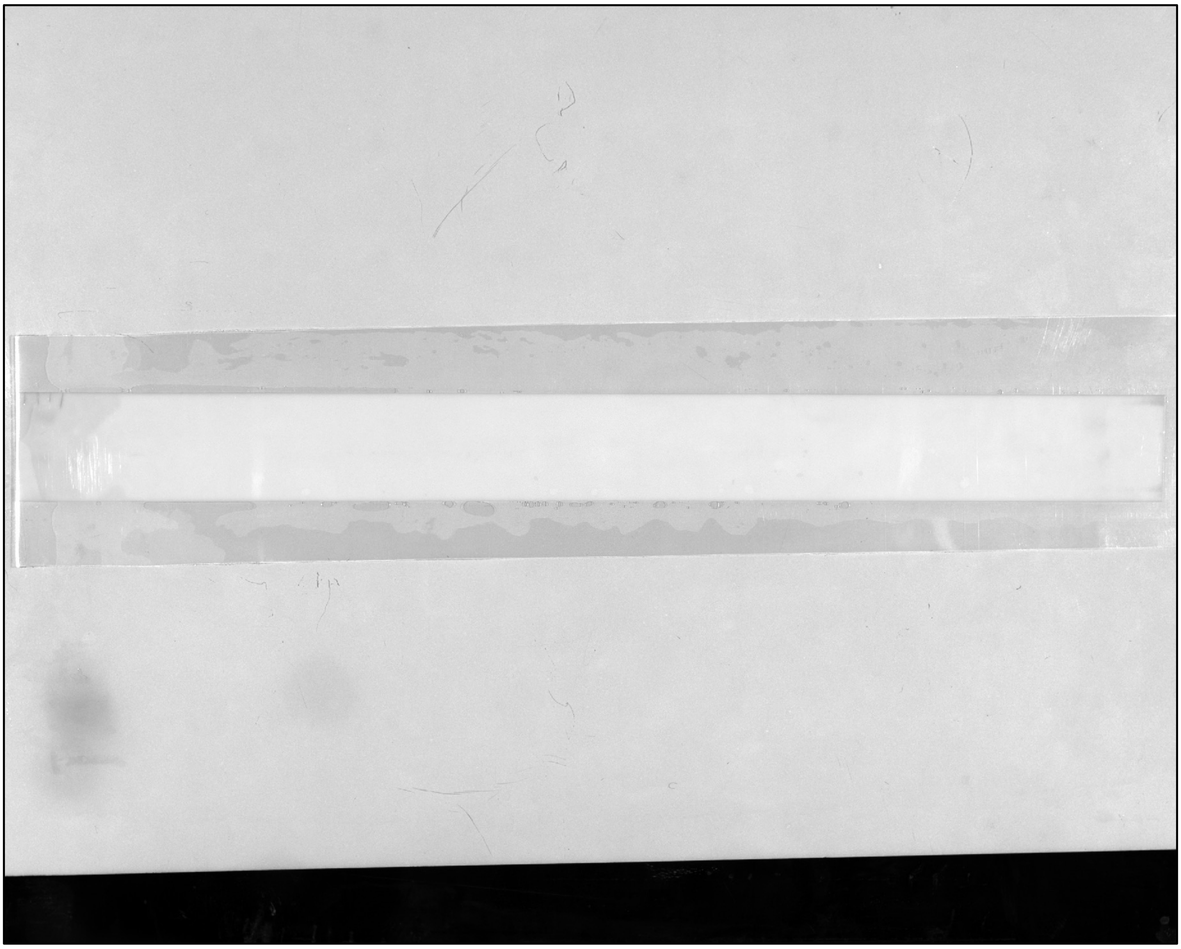

Band images in Fig. 8 (continued).

CBB
For P-p70S6K, P-rpS6, and P-4EBP1.

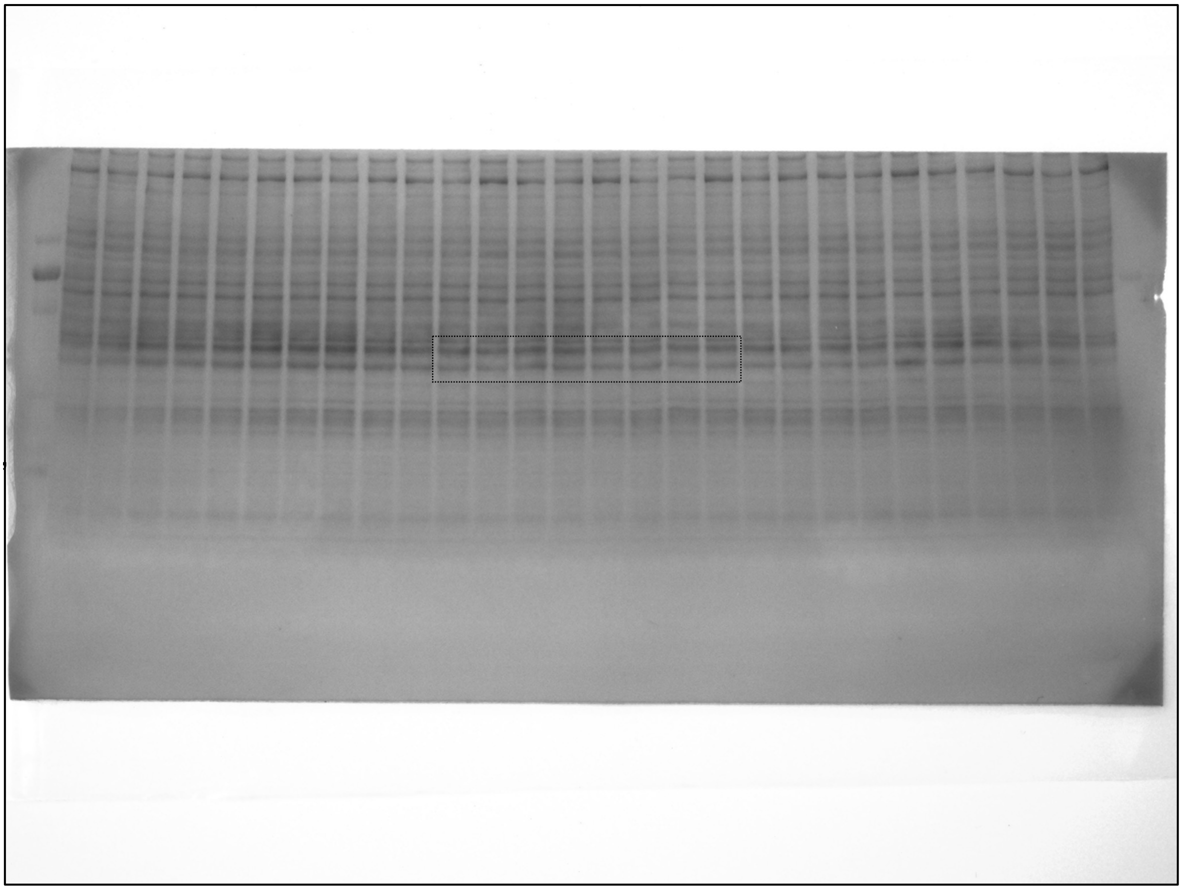

CBB

For T-p70S6K, T-rpS6, and T-4EBP1.

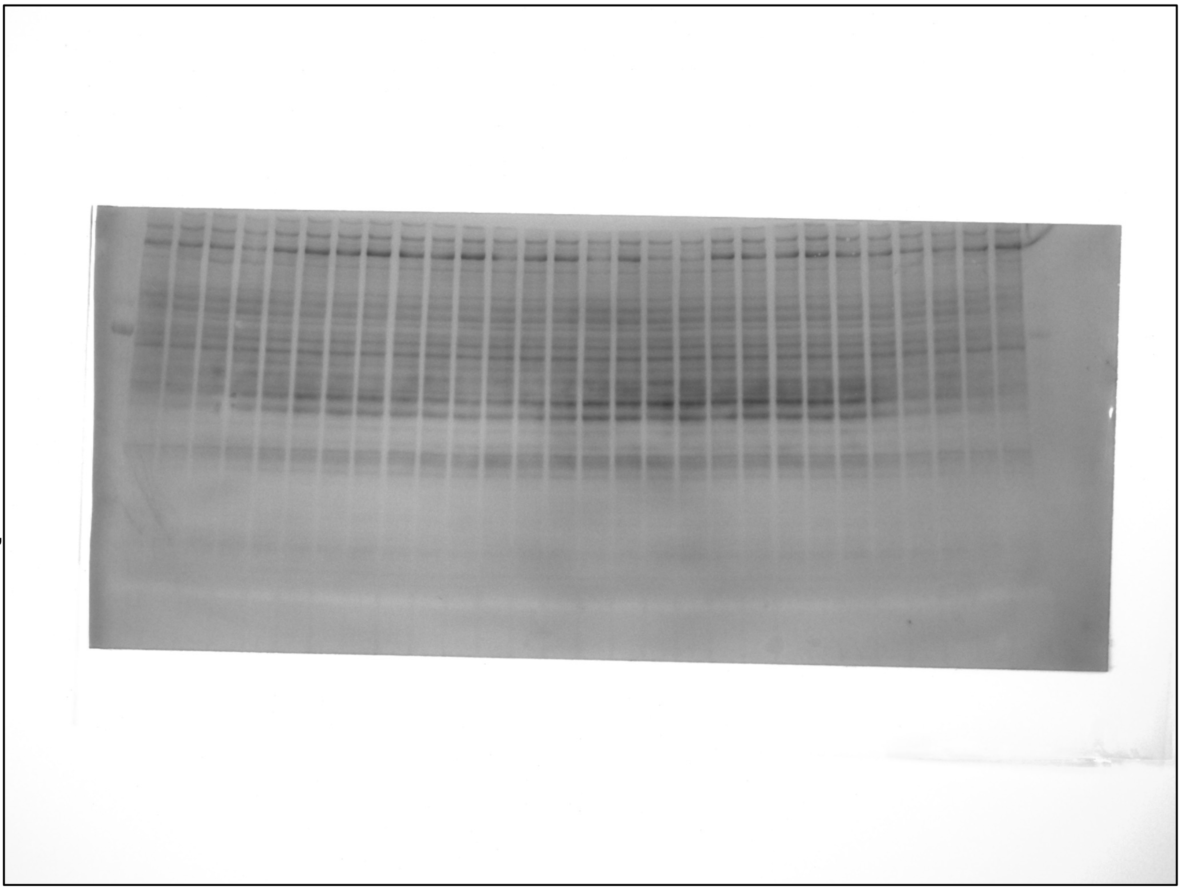

Band images in Fig. 8 (continued).

Puromycin

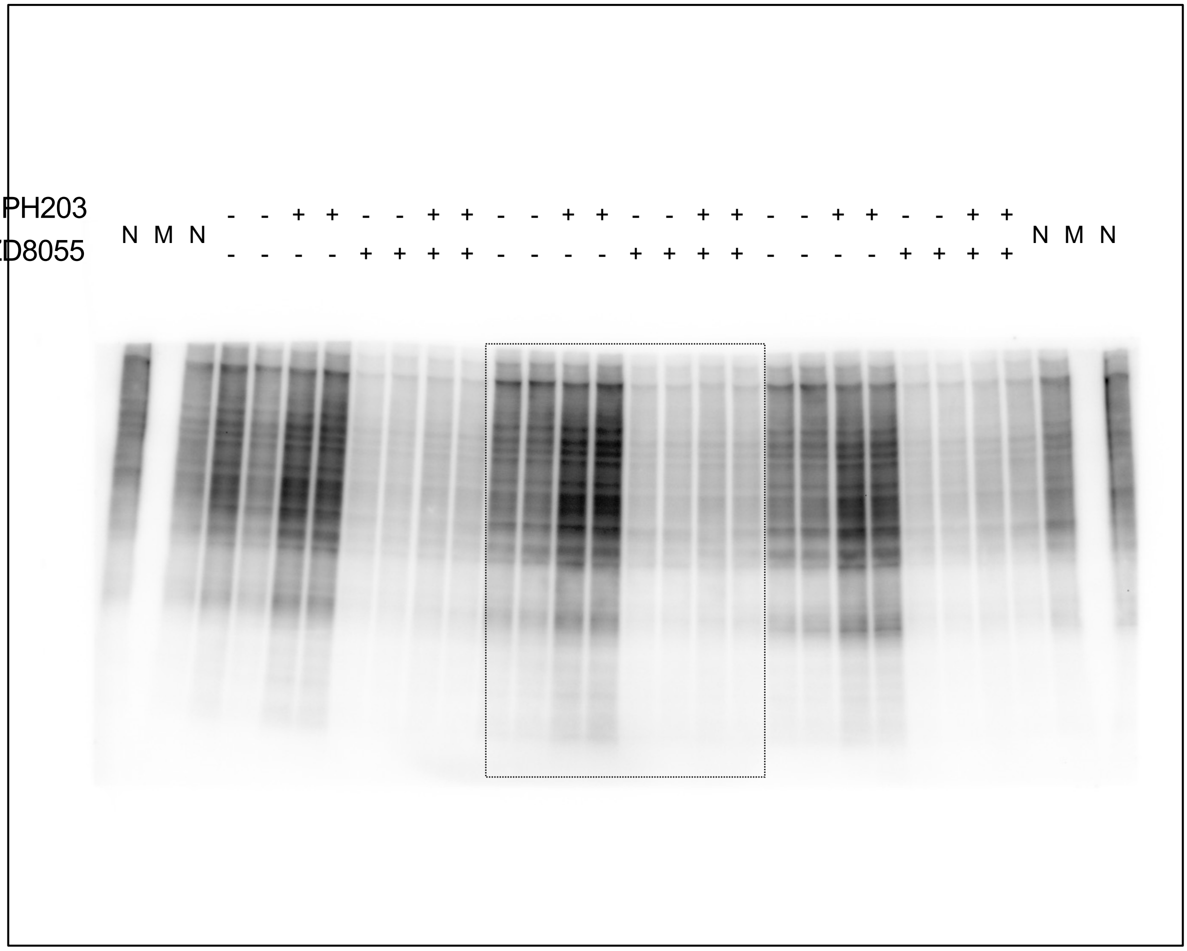

Bright field

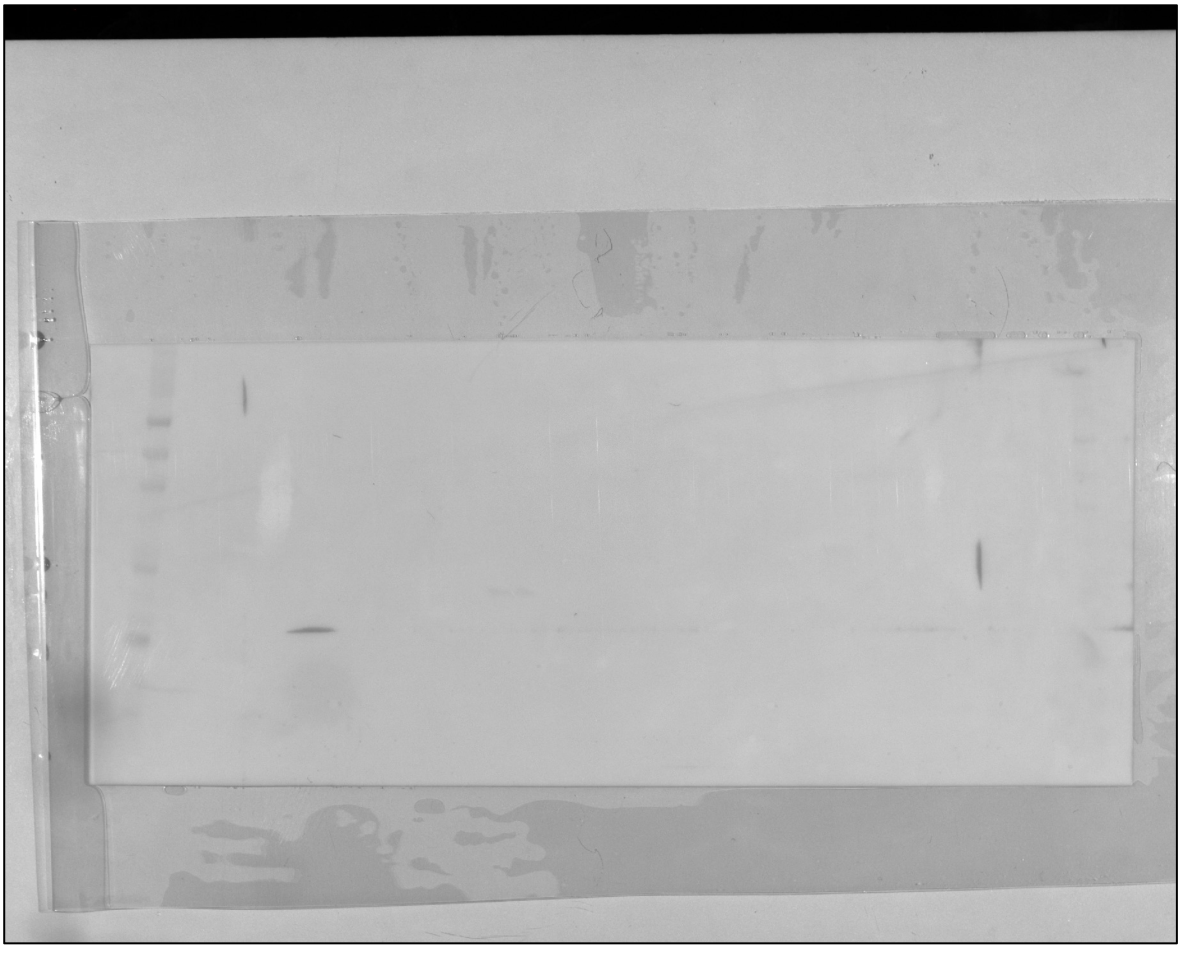

Band images in Fig. 8 (continued).

CBB

For
puromycin

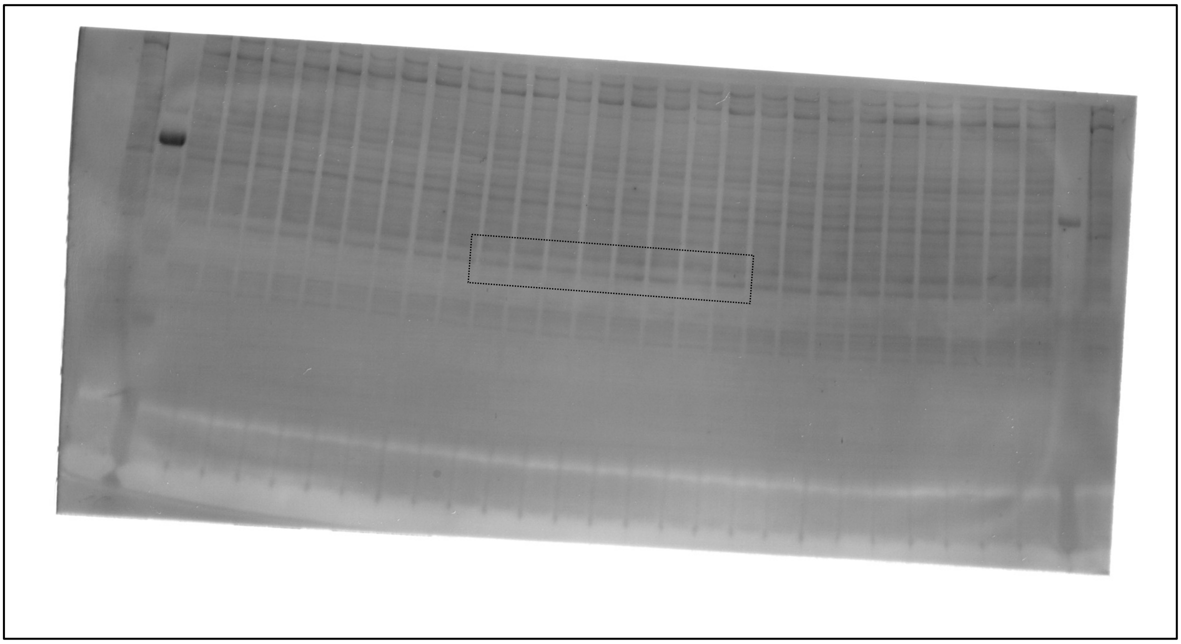

Supplemental information 2

Uncropped images used for amino acid uptake assay. Each images were taken under same condition.

Control

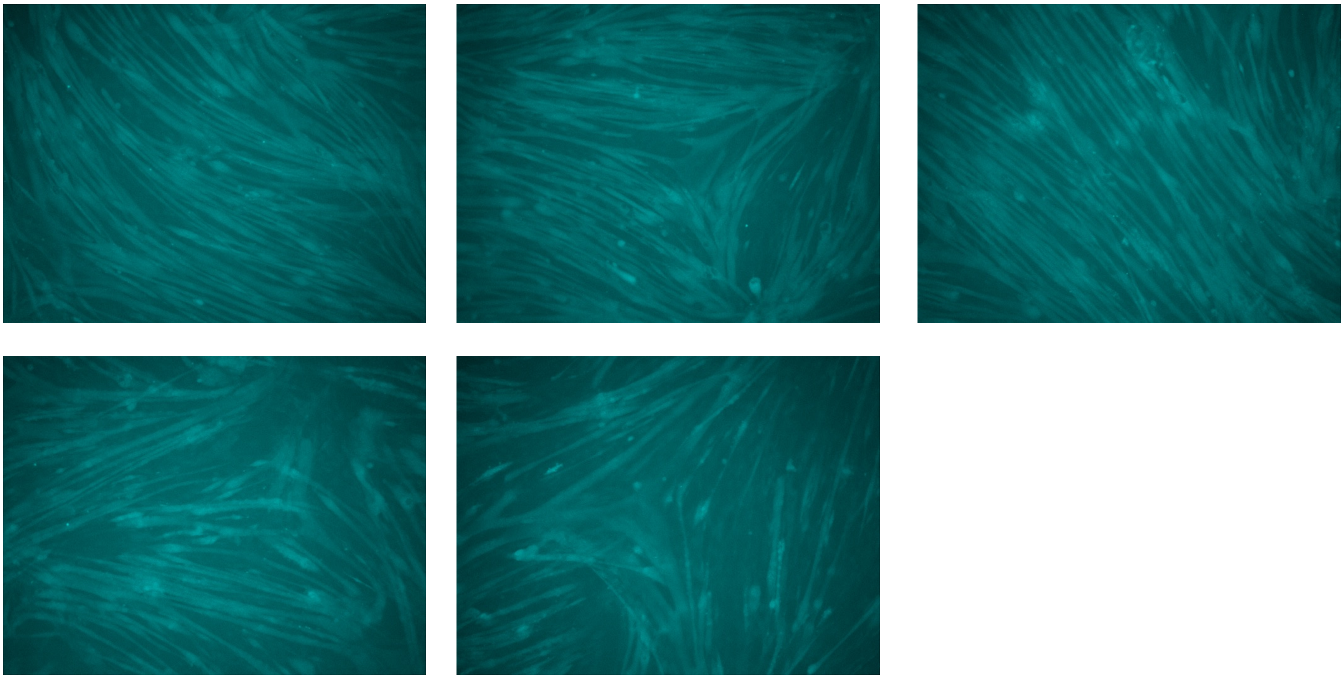

JPH203

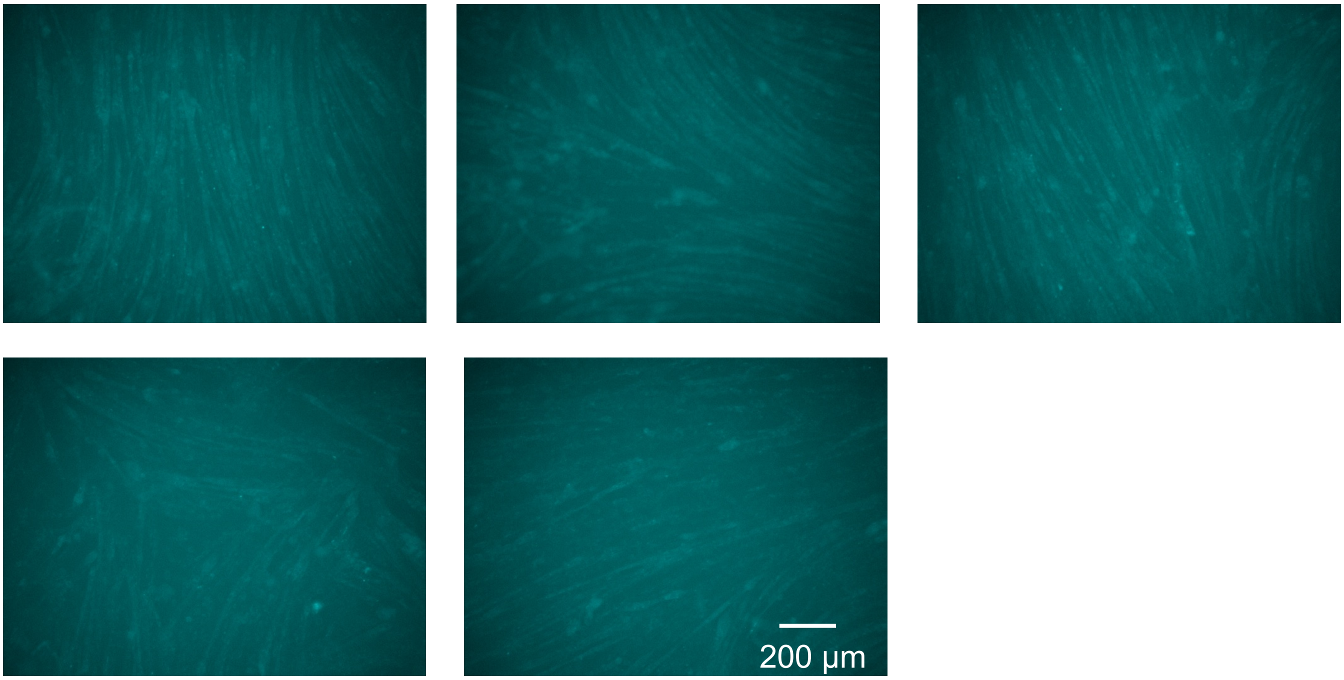

Supplemental information 3

Uncropped images used for measurement of myotube diameter. Each images were taken under same condition.

Control-1

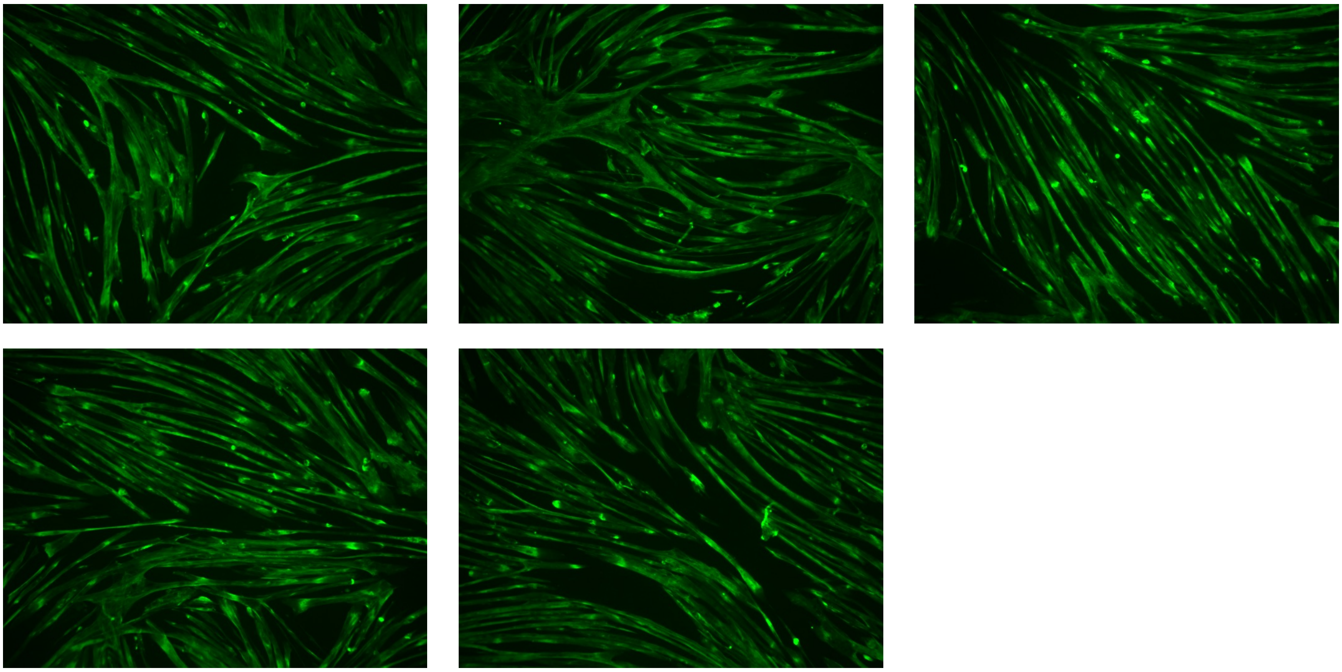

Control-2

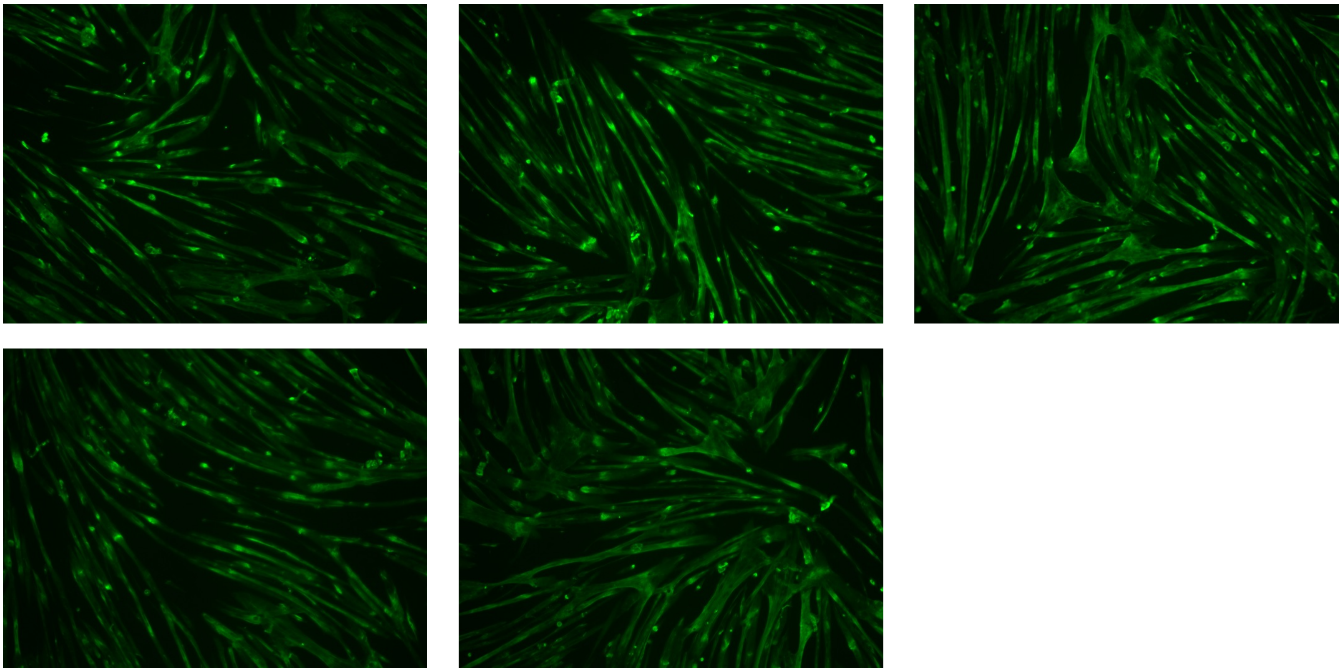

Control-3

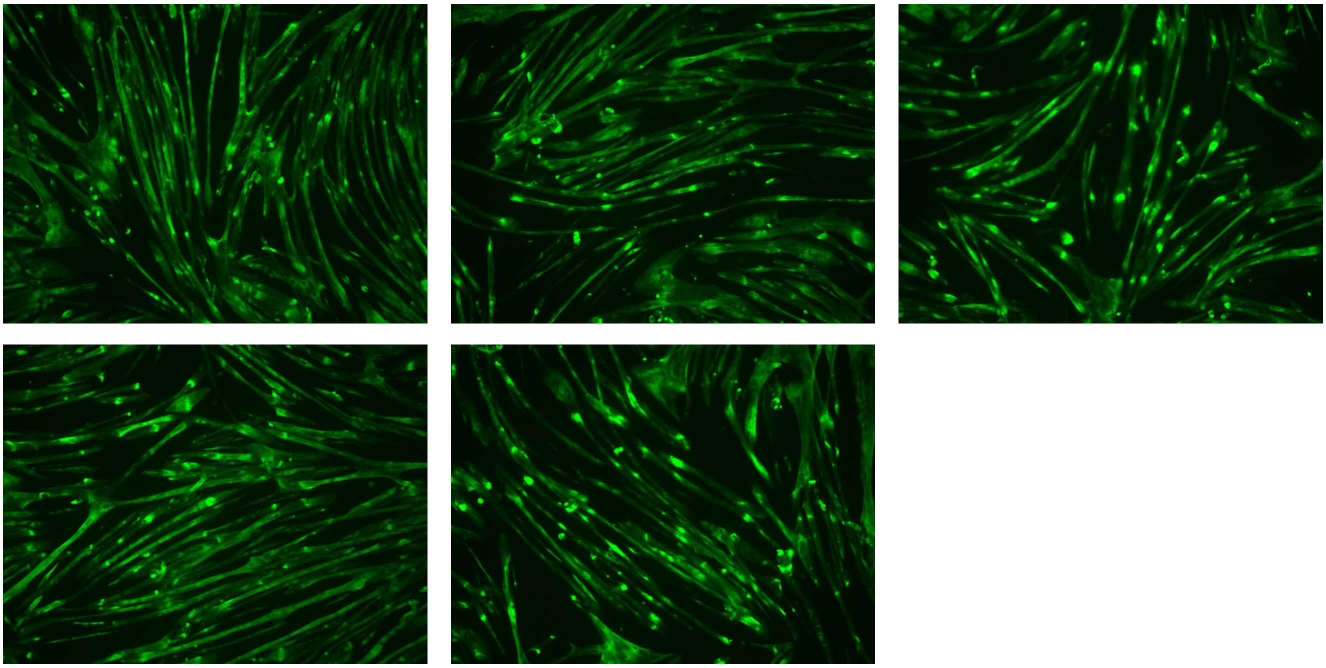

Control-4

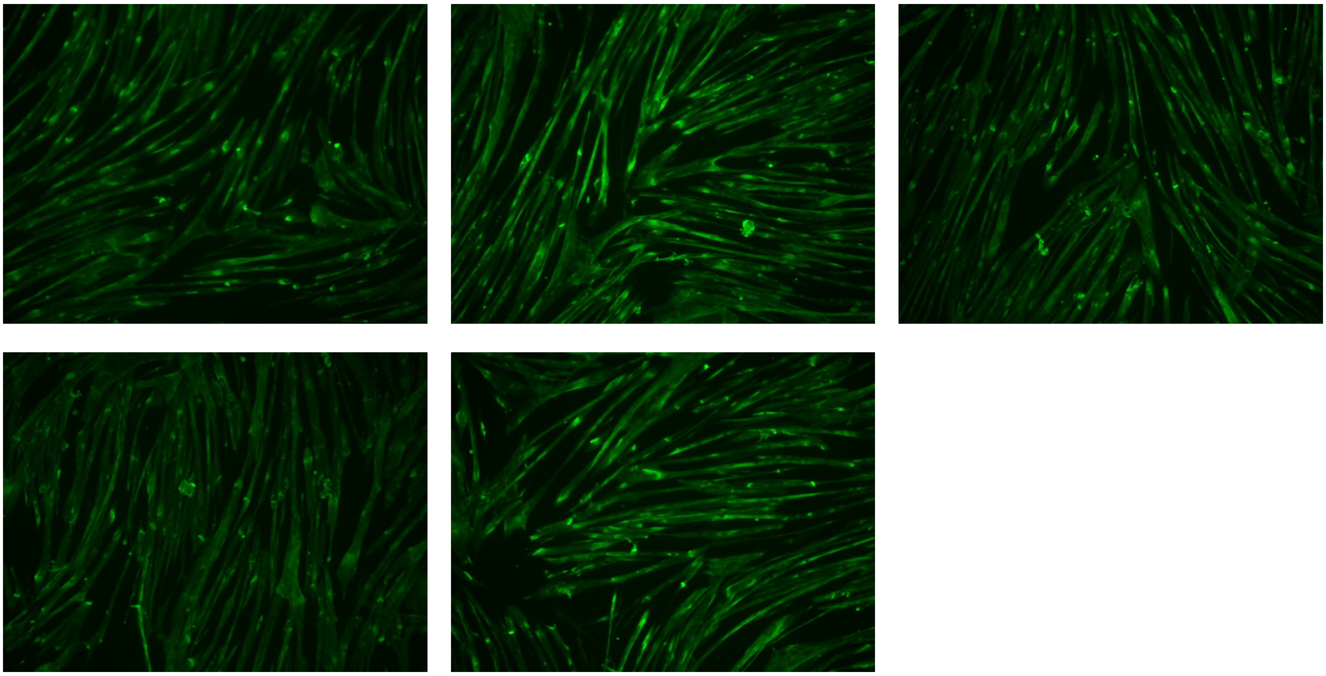

Control-5

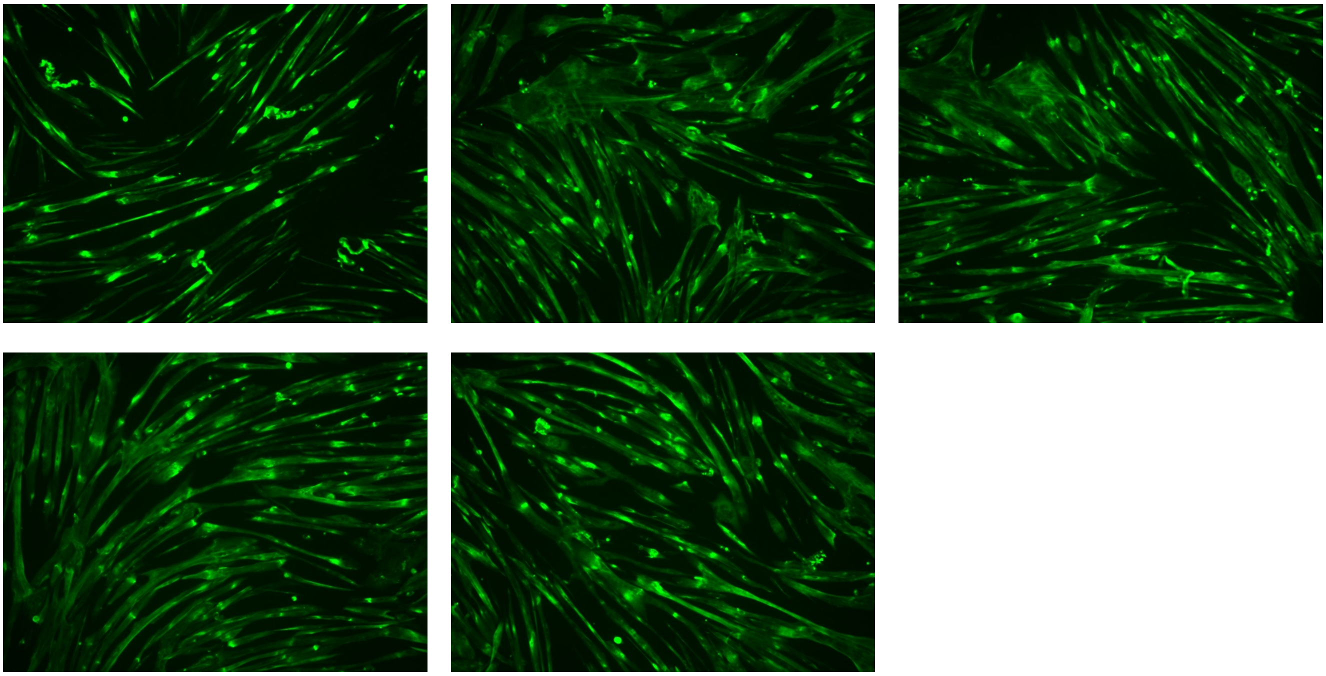

Control-6

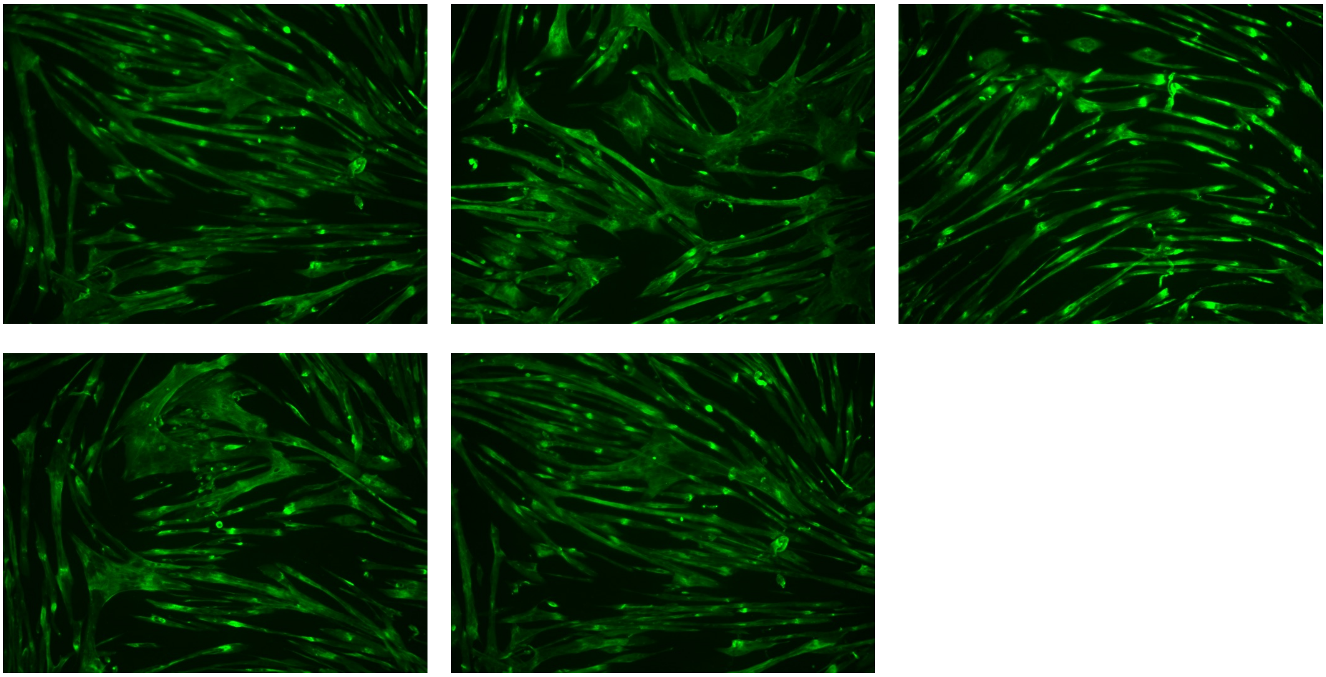

JPH203-1

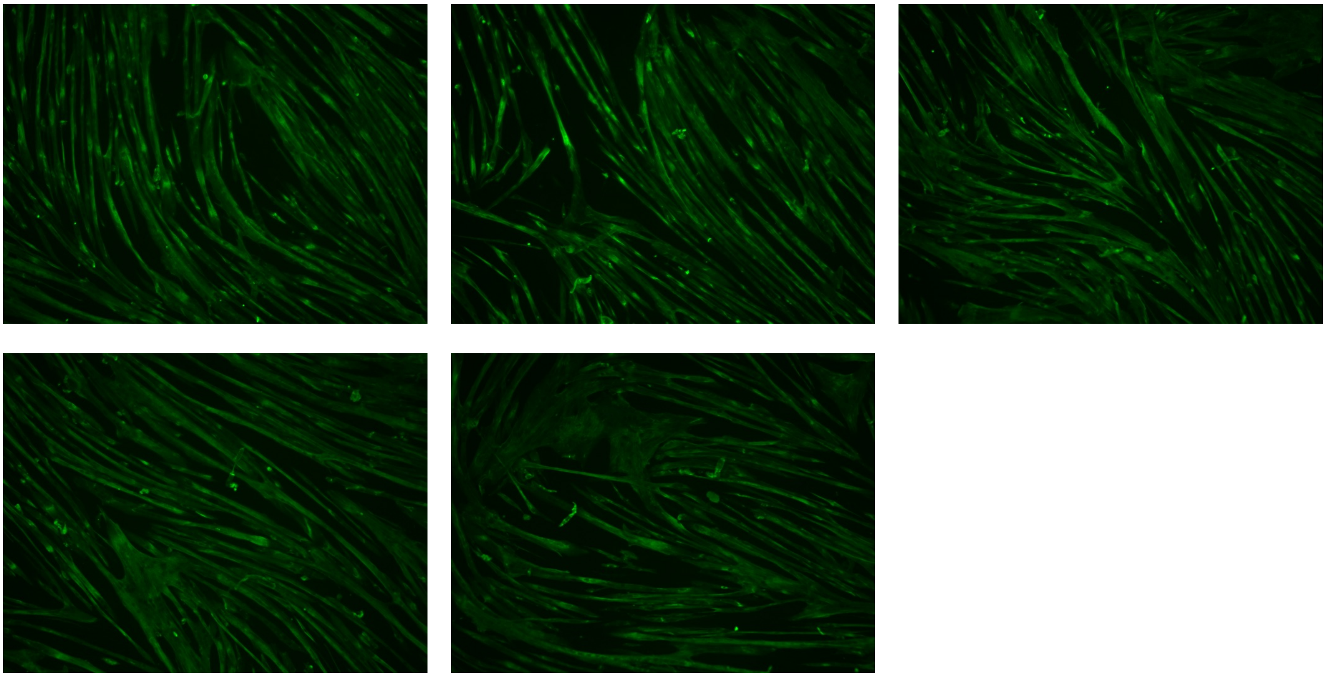

JPH203-2

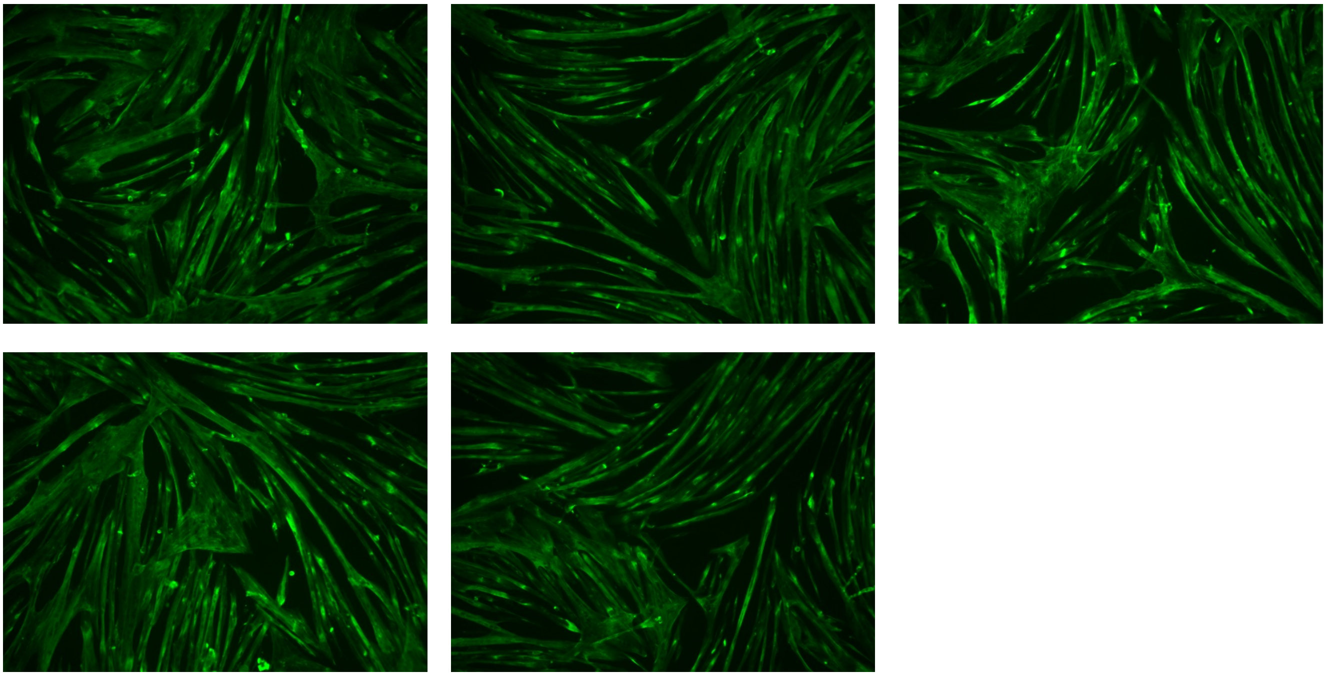

JPH203-3

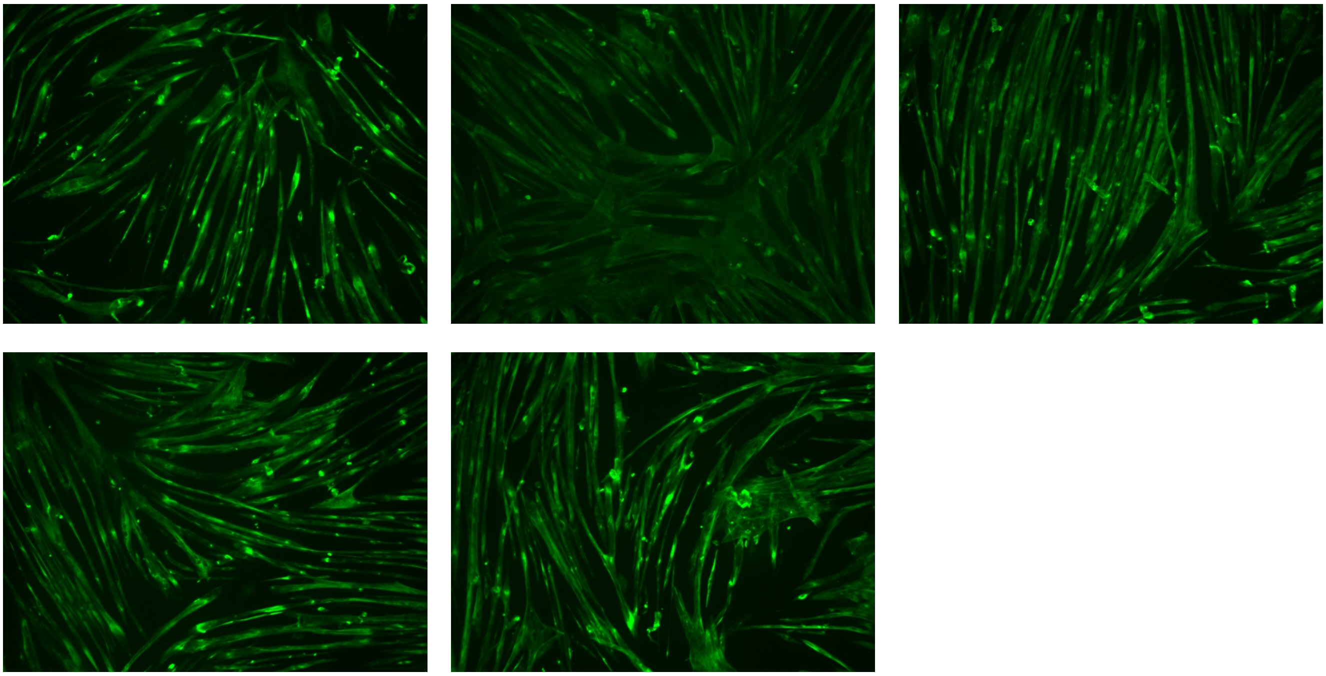

JPH203-4

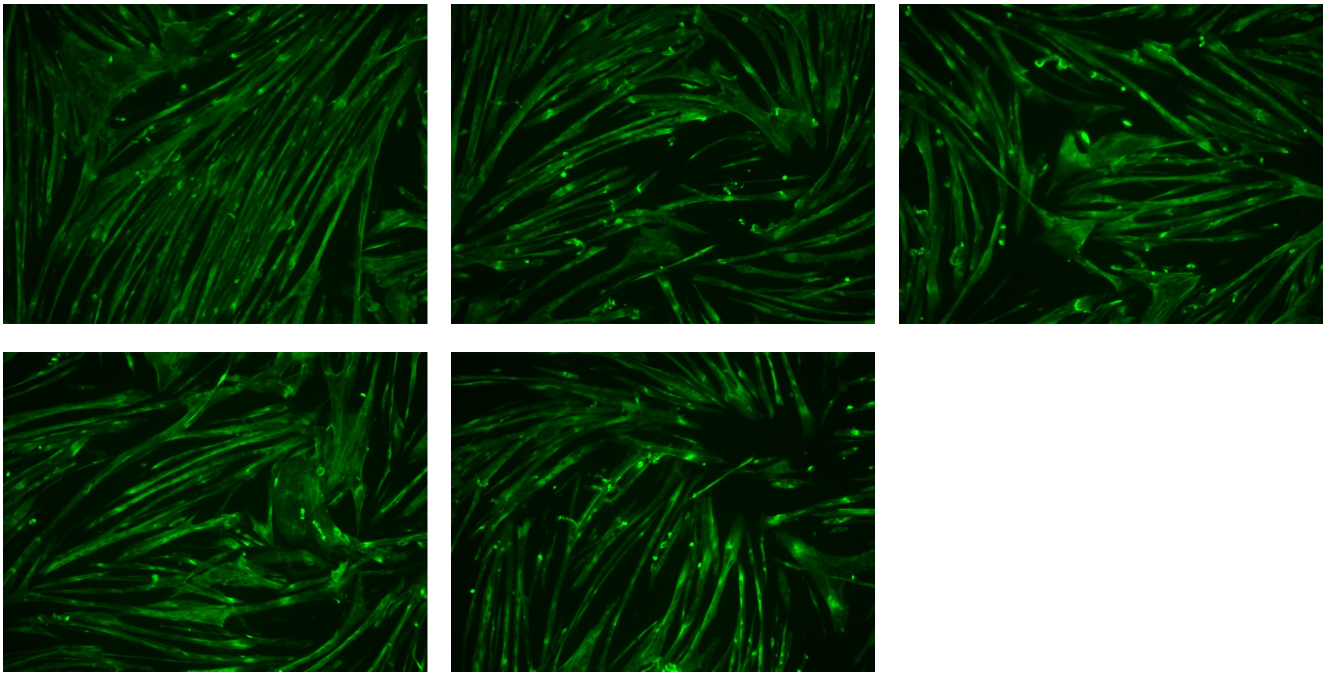

JPH203-5

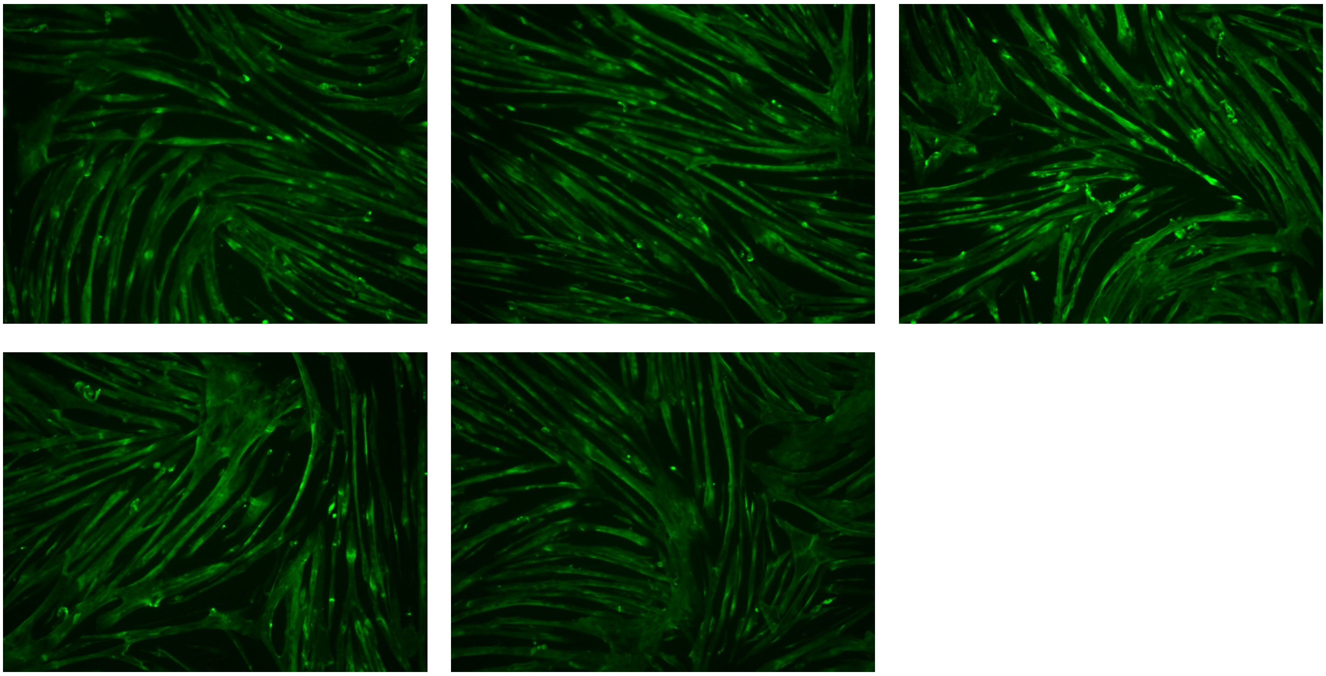

JPH203-6

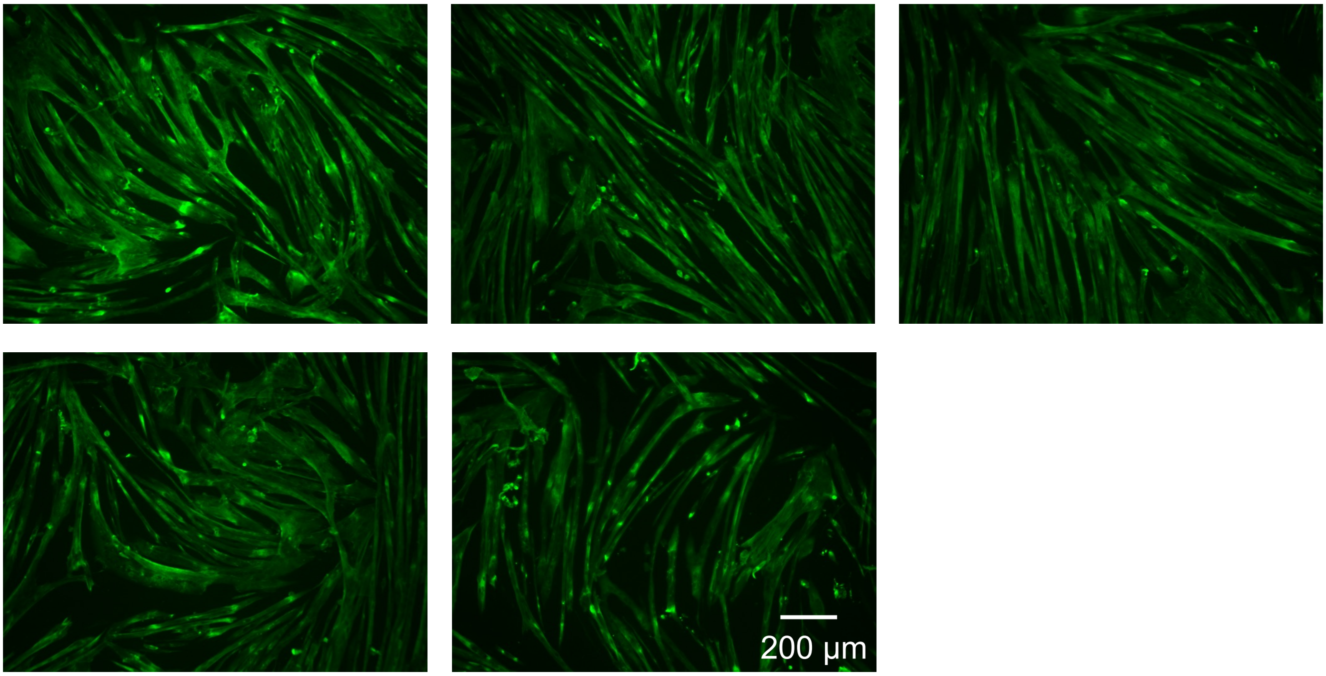

Supplement: Supplementary file 1 — Supplementary Material 1 [file 41598_2025_24534_MOESM1_ESM.pdf]
